# Identification of Arabidopsis Protein Kinases That Harbor Functional Type 1 Peroxisomal Targeting Signals

**DOI:** 10.3389/fcell.2022.745883

**Published:** 2022-02-15

**Authors:** Amr Kataya, Nitija Gautam, Muhammad Jamshed, Douglas G. Muench, Marcus A. Samuel, Jay J. Thelen, Greg B. Moorhead

**Affiliations:** ^1^ Department of Chemistry, Bioscience, and Environmental Engineering, University of Stavanger, Stavanger, Norway; ^2^ Department of Biological Sciences, University of Calgary, Calgary, AB, Canada; ^3^ Christopher S. Bond Life Sciences Center, Department of Biochemistry, University of Missouri, Columbia, MO, United States

**Keywords:** peroxisome, Protein Kinases, peroxisomal targeting signal 1, beta oxidation, kinome

## Abstract

Peroxisomes are eukaryotic specific organelles that perform diverse metabolic functions including fatty acid β-oxidation, reactive species metabolism, photorespiration, and responses to stress. However, the potential regulation of these functions by post-translational modifications, including protein phosphorylation, has had limited study. Recently, we identified and catalogued a large number of peroxisomal phosphorylated proteins, implicating the presence of protein kinases in this organelle. Here, we employed available prediction models coupled with sequence conservation analysis to identify 31 protein kinases from the Arabidopsis kinome (all protein kinases) that contain a putative, non-canonical peroxisomal targeting signal type 1 (PTS1). From this, twelve C-terminal domain-PTS1s were demonstrated to be functional *in vivo*, targeting enhanced yellow fluorescent protein to peroxisomes, increasing the list of presumptive peroxisomal protein kinases to nineteen. Of the twelve protein kinases with functional PTS1s, we obtained full length clones for eight and demonstrated that seven target to peroxisomes *in vivo*. Screening homozygous mutants of the presumptive nineteen protein kinases revealed one candidate (GPK1) that harbors a sugar-dependence phenotype, suggesting it is involved in regulating peroxisomal fatty acid β-oxidation. These results present new opportunities for investigating the regulation of peroxisome functions.

## Introduction

Peroxisomes are eukaryotic specific, single-membraned organelles involved in a host of specialized metabolic events, but are best characterized for their role in fatty acid β-oxidation and photorespiration (Li-Beisson et al., 2013; Kao et al., 2018). Peroxisomes derive from the endoplasmic reticulum (ER), have the ability to bind to and move on the cytoskeleton, to proliferate (Agrawal and Subramani, 2016; Neuhaus et al., 2016; Kao et al., 2018) and in plants were recently discovered to have intralumenal vesicles (Wright and Bartel, 2020). Pre-peroxisomes bud from specific ER domains and become mature peroxisomes through importing nuclear-encoded peroxisomal membrane (PMPs) and matrix proteins. Proteins synthesized in the cytosol enter peroxisomes via short sequence signals named peroxisome targeting signal type 1 (PTS1) and 2 (PTS2) located at protein C-termini and N-termini, respectively. The majority of peroxisomal matrix proteins have PTS1 with a prototype SKL> (this is the single amino acid code and >indicates a C-terminal stop codon), while some harbor PTS2 with RLX_5_HL as a prototype (Gould et al., 1987; Hu et al., 2012; Kao et al., 2018; Kunze, 2020). Bioinformatic prediction tools have been developed primarily for PTS1 identification and have been widely utilized to identify putative peroxisomal candidate proteins (Reumann, 2004; Lingner et al., 2011; Chowdhary et al., 2012; Skoulding et al., 2015; Wang et al., 2017; Reumann and Chowdhary, 2018).

Peroxisome biogenesis, functions (Kao et al., 2018), remodelling (Paudyal et al., 2017; Luo and Zhuang, 2018) and crosstalk with other organelles (Oikawa et al., 2015; Thazar-Poulot et al., 2015; Jaipargas et al., 2016) is expected to be fine-tuned under varying cellular conditions through various post-translational modifications (PTMs) (Sandalio et al., 2019). Yeast protein kinase and phosphatase mutants show a role for protein phosphorylation in peroxisome formation, size, fission, and pexophagy (Saleem et al., 2008; Oeljeklaus et al., 2016). We proposed that phosphorylation could control plant proteins shuttling between peroxisomes and other organelles, possibly under specific conditions (Kataya et al., 2019), similar to yeast glycerol-3-phosphate dehydrogenase 1 (GPD1), which changes its localization from the nucleus and cytosol to peroxisomes by a phosphorylation-dependent event (Jung et al., 2010). A limited number of studies have investigated protein phosphorylation in mammalian, yeast, and Arabidopsis peroxisomes (for a full overview we refer readers to two comprehensive reviews about this topic (Oeljeklaus et al., 2016; Kataya et al., 2019)). Recently, Arabidopsis and *Zea mays* peroxisomal photorespiratory glycolate oxidase activity was found to be modulated by phosphorylation (Jossier et al., 2019) and our recent analysis found more than half of the known peroxisomal proteome to be phosphorylated (Kataya et al., 2019). However, it is not known how phosphorylation controls these proteins or the identity of the protein kinases and phosphatases that regulate them. Over the last 2 decades, four Arabidopsis peroxisomal protein phosphatases were identified (Fukao et al., 2002; Matre et al., 2009; Kataya et al., 2015b; Kataya et al., 2016; Kataya et al., 2019). The number of protein kinases and phosphatases in Arabidopsis is ∼1100 and ∼150, respectively (reviewed in (Uhrig et al., 2013; Lillo et al., 2014)). Only calcium-dependent protein kinase 1 (CPK1) and glyoxysomal protein kinase 1 (GPK1) were reported as peroxisome targeted protein kinases (Dammann et al., 2003; Fukao et al., 2003; Coca and San Segundo, 2010) with no information about their impact on peroxisome function.

In this study, we screened the Arabidopsis kinome for potential PTS1 sequences, studied their conservation and experimentally investigated 31 protein kinases harboring putative PTS1s or PTS1-like tripeptide sequences. We confirmed functional peroxisomal targeting signals that belong to twelve of these protein kinases and studied the subcellular localization for eight of their full-length proteins. Seven additional protein kinases have previously been implicated as peroxisomal or at least harboring functional PTS1s. Here, we have used an array of approaches to confirm or refute these seven as true peroxisomal proteins. The peroxisomal protein kinase types, sequence relationship, and expression were studied, and several homozygous T-DNA insertion lines were isolated. These mutants were tested for sugar dependence phenotypes, implicating a role for one protein kinase in the peroxisomal degradation of fatty acids.

## Materials and Methods

### Gene Cloning for in Planta Expression

The 31 fusion constructs Supplementary Table 1 of the protein kinase peroxisomal targeting domains (PTD) were amplified from enhanced yellow fluorescent protein (EYFP) template using forward primer (CAC​CAT​GGC​AAT​GGT​GAG​CAA​GGG​CGA​GGA​G) and reverse synthesized primers obtaining the sequence that covers each C-terminal decapeptide of each (primer sequences are in Supplementary Table 2). Arabidopsis protein kinases cDNAs were either amplified from isolated RNA or obtained from the ABRC (Columbus, OH, United States) and RIKEN BioResource Center (Ibaraki, Japan), for more details see Supplementary Table 3. The amplified cDNAs were cloned/subcloned into pCAT-EYFP vector (Lingner et al., 2011; Kataya et al., 2016) to create N-terminal protein fusions with EYFP. The subcloning vector has a 35S promoter of cauliflower mosaic virus with a duplicated enhancer region and a 35S polyadenylation site.

### RT-PCR

Total RNA was extracted using RNAeasy Plant Mini Kit (Qiagen, Hilden, Germany), according to the manufacturer’s protocol. First-strand cDNA synthesis was performed using Superscript IV reverse transcriptase (Invitrogen, Carlsbad, CA, United States) in a 20-µl standard reaction mixture containing gene-specific primers. PCR amplification was done using the Expand High FidelityPLUS PCR System (Roche, Mannheim, Germany). Primers for RT-PCR amplifications, and cloning are found in Supplementary Table 3.

### Bioinformatics

Sequencing the recombinant constructs was accomplished by Seqlab (Gottingen, Germany) using their facility of Extended Hotshots reactions and the DNA Core DNA services, the University of Calgary (Calgary, Canada). The general promoters T7, SP6, 35S, and NOS terminator primers were used for sequencing in pGEM-T Easy and EYFP-containing plasmids. Sequence analysis was done using Vector NTI (Invitrogen, Carlsbad, CA, United States) in combination with web-based programs for reversing DNA (http://www.bioinformatics.org/SMS/rev_comp.html) and protein translation (http://us.expasy.org/tools/dna.html).

Sites used for predicting PTS1 scores are PredPlantPTS1, http://ppp.gobics.de/ (Lingner et al., 2011; Reumann et al., 2012) and PPero, https://biocomputer.bio.cuhk.edu.hk/PP/ (Wang et al., 2017). The prediction threshold was reported to be 0.412 and 0.218 for the PWM and RI models, respectively. To further facilitate the prediction, model-specific posterior probabilities were used to quantify the peroxisome targeting probability (Lingner et al., 2011). PPero, a new computational model for predicting PTS1 was developed and reported to improve the PTS1 prediction where the coauthors used and compared their scores with the posterior probability from Lingner et al. and set their prediction threshold to 1.5 (Lingner et al., 2011; Wang et al., 2017). PPero developed a bi-profile Bayesian SVM method to extract and learn position-based amino acid features for both PTS1 motifs and their extended adjacent sequences (beyond 14 aa) in plants (Wang et al., 2017). Phylogenetic relationships were inferred by preferential alignments of the protein sequences obtained from NCBI. This was done using the program MEGAx (Kumar et al., 2018) and vector NTI (Invitrogen, Carlsbad, CA, United States).

### Transformation and Microscopy

For transformation analysis in onion epidermal cells and *Nicotiana tabacum* leaves, plasmids were transiently introduced by a helium-driven particle accelerator (PDS/1000; Bio-Rad, Hercules, CA, United States). The bombarded tissues were incubated for one to 2 days in the dark at room temperature and then observed under the microscope. Peroxisomal markers used were gMDH-CFP (Fulda et al., 2002) that contains 50 N-terminal amino acids (including the PTS2) from Cucumis sativus glyoxysomal malate dehydrogenase linked with cyan fluorescent protein, and RFP::SKL that contains red fluorescent protein fused with the tripeptide Ser-Lys-Leu to have a C-terminal PTS1 (Matre et al., 2009). Epifluorescence and confocal imaging microscopy and image processing were carried out following the procedures described in Kataya et al. (2016), Uhrig et al. (2017), Alberts et al. (2019). Typically, 5 to 10 images were captured for each construct in three independent experiments, with a representative image shown. Figures 3–5 and Supplementary Figures 3–5 are showing localization images, which were captured by confocal and epifluorescence imaging, respectively for the same constructs.

### Plant Growth

Arabidopsis (Col-0) WT and T-DNA lines were obtained from the European Arabidopsis Stock Centre (Nottingham, United Kingdom, see Supplementary Table 4). Homozygous mutant selection was performed by PCR using primers (see Supplementary Table 4 for genotyping primers sequences) for the T-DNA insertion lines recommended at the SALK institute website SIGnAL (http://www.signal.salk.edu/tdnaprimers.2.html). For plant material grown on soil, seeds were sown directly in a regular soil plant mix. Seeds were stratified for 2 days and transferred to standard growth conditions.

### Sugar Dependence Assay

For sugar dependence analysis, seeds of WT Col-0 and mutant were sown on ½ MS medium with vitamins (SIGMA, United States) with or without 1% (w/v) sucrose and stratified in the dark at 4°C for 2 days before being transferred to light for 8 h, and subsequently to darkness at 20°C. Five-day-old seedlings were scanned, and hypocotyl length was measured using ImageJ (Schneider et al., 2012) (http://rsb.info.nih.gov/ij/).

### Accession Numbers

Accession numbers are provided in Supplementary Table 1 and Table 1.

**TABLE 1:** Summary of full-length protein kinases and peroxisomal targeting validation studies. Protein kinase candidates found to harbor functional PTS1s (from this study and previous studies) and those found in a previous peroxisomal proteome are listed. The table is divided into three parts: protein kinases targeted to punctate structures in onion and co-localized with a peroxisomal marker in tobacco; protein kinases targeted partly to peroxisomes in tobacco and remained undetected in onion; non-peroxisomal targeting in both tobacco and onion, and untested protein kinases that harbor functional PTS1. All candidate full-length cDNAs were either subcloned or cloned N-terminally with EYFP from plasmids provided by ABRC or RICKEN or isolated Arabidopsis total RNA (Supplementary Table 3). Only 4 of 12 protein kinase cDNAs were not retrieved.

AGI	Annotation	Acronym (TAIR)	Kinase number	Full-length localization Onion^a^	Full-length localization tobacco^a^	PTD^b^	PTDPeroxisomal localization^c^
Confirmed peroxisomal localization							
AT5G07180.1	Receptor-like kinase; ERECTA-LIKE 2	ERL2	K1	Punctate structures	Peroxisomes	FREDISKSSL	This study
AT5G60300.3	L-type lectin receptor kinase I.9	LECRK-I.9, P2K1, DORN1	K2	Punctate structures, aggregates	Peroxisomes	LFFFLQLARL	This study
AT4G13190.1	Protein kinase superfamily protein	PBL24	K3	Punctate structures, nucleus?	Peroxisomes, nucleus?	ESPRDVYSLL	This study
AT5G49660.1	Leucine-rich repeat transmembrane protein kinase family protein	CEPR1, XIP1	K4	Punctate structures	Peroxisomes	VSDHLTQTRL	This study
AT3G57760.1	Protein kinase superfamily protein	ZRK6	K8	Punctate structures, nucleus?	Peroxisomes	SNNRSQMSSI	This study
AT4G31230.1	Kinase with adenine nucleotide alpha hydrolases-like domain-containing protein	PK2	K13	Cytosol, punctate structures, nucleus?	Peroxisomes, nucleus?, cytosol	TESQTSSPKL	Ma and Reumann, (2008)
AT3G08720.1	Serine/threonine protein kinase 2	ATPK19, ATPK2, PK6	K15	Punctate structures, network-like	Peroxisomes	SFLHRTTSNL	Ma and Reumann, (2008)
AT3G20530.1	Protein kinase superfamily protein	PBL23, PK1	K16	Punctate structures	Peroxisomes	EEEEDERSKL	Ma and Reumann, 2008; Lingner et al., 2011; Wang et al., 2017
Partial peroxisomal localization							
AT2G26830.1	Protein kinase; Embryo defective 1187; Choline/Ethanolamine kinase 4	CEK4, EMB1187	K5	Cytosol, nucleus?	Cytosol, nucleus? partly in peroxisomes	LVTSHLSASL	This study
AT1G76540.1	Cyclin-dependent kinase	CDKB2-1	K6	Cytosol, network-like, nucleus?	Network-like, partly in peroxisomes	FDDLPEKSSL	This study
AT3G17420.1	Glyoxysomal protein kinase 1	GPK1, PK7	K17	Cytosol, little punctate structures	Partially in peroxisomes	DNDITTDAKI	Ma and Reumann, (2008)
AT5G03730.1	Constitutive triple response 1; sugar insensitive 1	CTR1, SIS1	K19	Punctate structures, nucleus?	Partially in peroxisomes	AVPPPNRSDL	Chowdhary et al. (2012)
Non-peroxisomal localization and untested full-length protein kinases							
AT1G29720.1	Transmembrane protein kinase	RFK1	K9	ND	ND	STVENSSSSL	This study
AT5G51560.1	Leucine-rich repeat protein kinase family protein	ND	K10	ND	ND	HELGNCSSCL	This study
AT1G34420.1	Leucine-rich repeat transmembrane protein kinase family protein	ND	K11	ND	ND	KTVLRMLTRL	This study
AT1G74330.1	Protein kinase superfamily protein	ND	K12	ND	ND	KKILLFSSEL	This study
AT3G61960.1	Protein kinase superfamily protein	ATG1A, ATPK4	K7	Cytosol	Cytosol, network-like	SNLQHRRSHL	This study; Ma and Reumann, 2008
AT1G69270.1	Receptor-like protein kinase 1	RPK1, PK5	K14	Cytosol, network-like	Cytosol, network-like	LLKRIQPSRL	Ma and Reumann, 2008; Narsai et al., 2011
AT5G04870.1	Calcium-dependent kinase 1	CPK1	K18	Cytosol, network-like	Cytosol, network-like	EKSFSIALKL	Lingner et al. (2011)

a The results of subcellular localization of EYFP fusions in onion epidermal cells and tobacco, respectively (Figures 3–5; Supplementary Figures 3–6).

b The cloned presumptive PTD (C-terminal decapeptides of each kinase).

c The study where the PTS1/PTD investigation was reported. ND, not determined.

## Results

### Bioinformatic Screening for Putative Peroxisomal Signals in the Arabidopsis Kinome

The updated Arabidopsis peroxisomal proteome harbors approximately 200 unique members (catalogued in Pan and Hu (2018)). However, to date, only two peroxisomal protein kinases have been reported by proteomic and subcellular localization studies. The fragile nature and the low number of peroxisomes in cells have made it difficult to identify low-abundance proteins, some of which may only transiently shuttle into peroxisomes under special circumstances (Ma and Reumann, 2008; Bussell et al., 2013). To determine protein kinase candidates for their peroxisomal localization, the Arabidopsis kinome (Zulawski et al., 2014) was searched for the presence of canonical and non-canonical PTS1s. Our search utilized the prediction tools position-specific weight matrices (PWM) and residue interdependence (RI) models in identifying putative PTS1s (Lingner et al., 2011). We extracted scores for seven unstudied protein kinases (Supplementary Table 1), which are predicted to harbor functional PTS1 tripeptides (SSL>, ARL>, SQM>, SLL>, SEL>, KRL>, SEL>) at their C-termini. Also, using the newly developed model “PPero”, we were able to extract seven additional protein kinases that harbor putative PTS1s (SKD>, 3 with SSL>, SCL>, 2 with SHL>), that were not detected by the PWM or RI models (Supplementary Table 1).

Although these prediction algorithms are able to correctly infer peroxisomal targeting for many plant proteins, it is advised to use caution and verify predictions because in the past several below-threshold examples proved to be positive, and some predicted examples failed to target to peroxisomes (Lingner et al., 2011; Chowdhary et al., 2012; Kataya et al., 2015b; Wang et al., 2017). Taking this into consideration, we expanded our selection to include several candidates located below the threshold of all prediction methods. Thus, we included several candidates harboring non-canonical PTS1s (TRL>, GKL>, SLM>, ASL>, SSL>, ANL>, SEM>), or tripeptides with novel residues at different positions (SIM>, SGM>, RRL>) (Supplementary Table 1). Canonical tripeptides are the dominant signals of high-abundance peroxisomal proteins and are sufficient for peroxisomal targeting, while non-canonical signals are usually found in low-abundance peroxisomal proteins and require target enhancing elements, which are located upstream of the tripeptide and generally fall in the next seven residues N-terminal to the PTS1 (Lingner et al., 2011). We also selected two protein-kinase candidates found harboring KRR>, an unusual tripeptide reported to be a functional PTS1 (Ramirez et al., 2014). Because the Q at position -3 was reported in QRL> (Chowdhary et al., 2012) and KM> recently was reported in a novel PTS1 (PKM>, (Kataya et al., 2020a)), we added a protein kinase ending with QKM> to our list. In total, we gathered 31 protein kinase candidates that harbor putative functional PTS1s.

### Conservation of PTS1-Like Tripeptides in Protein Kinase Orthologs

To further support our selection process, we examined if the candidate PTS1 or the PTS1-like tripeptides are conserved (Supplementary Figure 1). We extracted orthologous sequences using BLAST and performed alignments. Our analysis used two major parameters for finding orthologs: 1) the conservation of predicted putative PTS1 tripeptides, and 2) the presence of divergent, but known PTS1 tripeptides in the ortholog. Out of the 31 candidates, 15 displayed conservation of their putative PTS1s, including 10 with wide conservation in planta (Supplementary Figure 1). For the PWM-selected candidates, the protein kinases AT4G13190.1 and AT3G24790.1 ending with the PTS1 tripeptides SLL> and SQM>, respectively, were found to have conservation of the PTS1 in their orthologs (see PTD3 and PTD15 in Supplementary Figure 1). The PWM and RI predicted candidate, AT5G60300.3 (ARL>), has only two orthologs with the PTS1 tripeptides QRL>, and ASL>, respectively (see PTD2 in Supplementary Figure 1). For the PPero-selected candidates, six had wider conservation of the PTS1 tripeptides (Supplementary Figure 1). For example, the tripeptides SHL> and SCL> were widely conserved and fulfilled parameter 1 in the orthologs of AT3G61960.1 and AT5G51560.1, respectively (see PTD7 and PTD10 in Supplementary Figure 1). The rest of the PPero-selected candidates fulfilled both conservation parameters (see PTD1, PTD9, PTD23, and PTD26 in Supplementary Figure 1). The targeting sequence conservation supports the idea that this group of protein kinases reside in peroxisomes and encouraged us to pursue further studies.

Additional candidates were found to have a conserved PTS1 in their orthologs. For example, AT2G26830.1 (ASL>), AT3G57760.1 (SSI>), and AT1G66880.1 (ASL>) have limited orthologs that are found primarily in the same family/tribe (Brassicaceae/Camelineae). AT2G26830.1 has an ortholog from the genus Capsella (*Capsella rubella*, SSV>) and other two orthologs with SSV> and SSL> (see PTD5 in Supplementary Figure 1). AT3G57760.1 retrieved one ortholog with STI> (see PTD8 in Supplementary Figure 1) from the same genus as Arabidopsis (*Arabidopsis lyrata*). AT1G66880.1 retrieved several orthologs with the PTS1-tripeptide in the genus Arabidopsis and Capsella, in addition to two in *Camelina sativa* with ASL> (see PTD24 in Supplementary Figure 1). This pattern of limited conservation aligns with the evolution of peroxisomal signals such as seen for the PP2A B’θ-SSL> signal, which was found to be limited to the same family/tribe (Brassicaceae/Camelineae) (Kataya et al., 2015b; Booker and Delong, 2017). The candidates: AT5G49660.1 (TRL>), AT1G34420.1 (TRL>), and AT1G76540.1 (SSL>) were found to have a broader conservation of PTS1 (Supplementary Figure 1). These outcomes strengthen the probability that these expanded selected candidates have functional peroxisomal targeting signals.

### Experimental Validation of Protein Kinase PTS1s

#### Peroxisomal Targeting With Protein Kinase C-Terminal Residues

**FIGURE 1:**
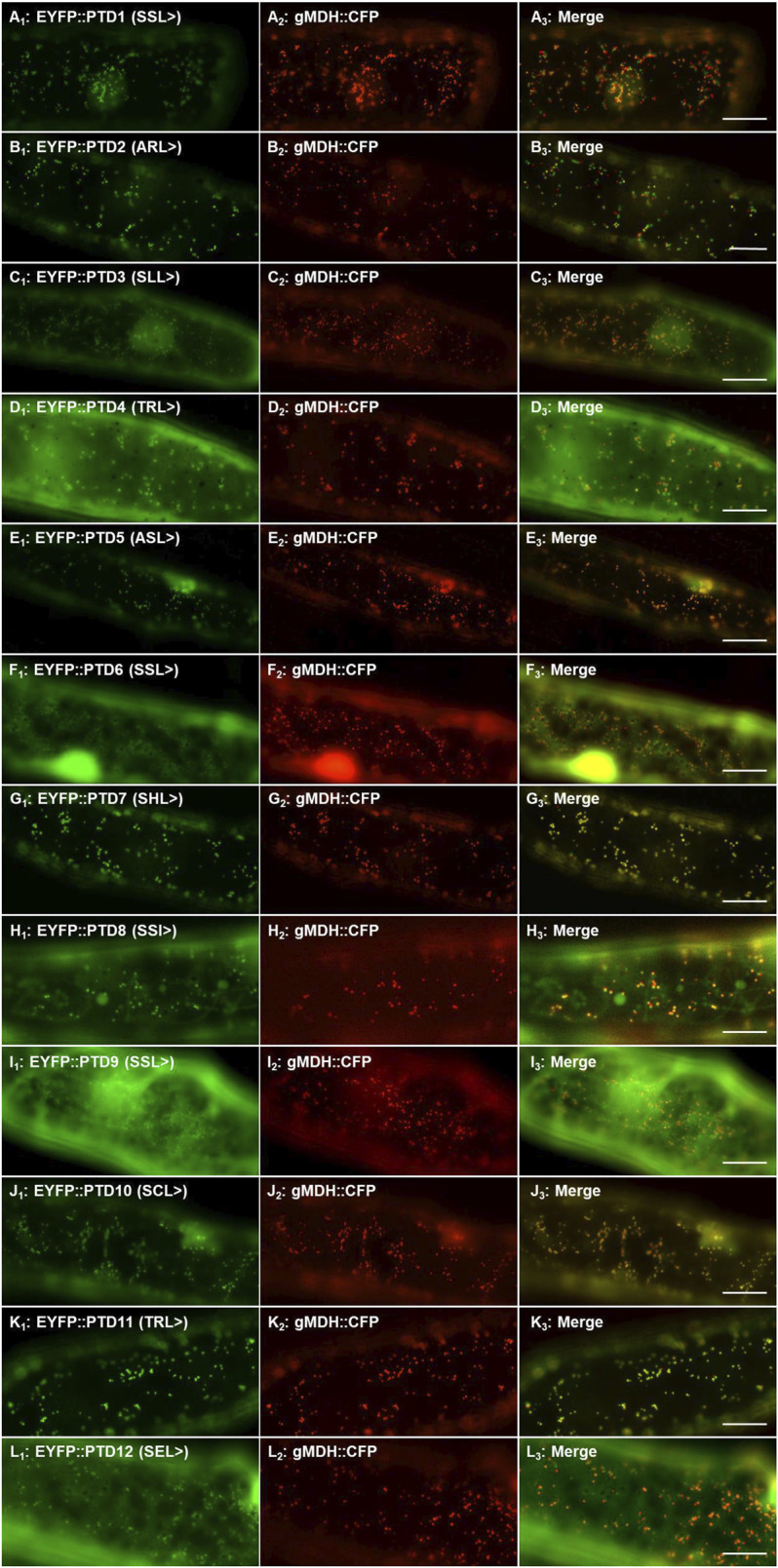
Peroxisomal targeting validation of twelve protein kinase peroxisomal targeting domains (PTD). The ten amino acid PTD of 31 protein kinases were fused to the C-terminus of EYFP and the translated products visualized through transient expression in onion epidermal cells. Twelve PTDs localized EYFP to punctate structures (Supplementary Figure 2) and here the peroxisomal identity of these structures were verified by signal coincidence with the gMDH-CFP reporter (Fulda et al., 2002). PTD number refers to the protein kinase it was derived from (ex. PTD1 = K1) and the C-terminal 3 residues are indicated in brackets. Shown left to right are EYFP signals **(A**_**1**_ **)**, CFP signals **(A**_**2**_ **)** and merge **(A**_**3**_ **)**. The subcellular localization of the extended reporter proteins was investigated through transient expression in onion epidermal cells (after ∼18 h expression at room temperature) upon biolistic bombardment. The cyan fluorescence was converted to red. Representative images (produced by fluorescence microscopy) are shown. Scale bars are 20 μm.

To study the functionality of the protein kinase C-terminal tripeptides as peroxisomal targeting signals, we fused the C-terminal ten residues of the selected 31 protein kinases to EYFP (Supplementary Table 2) and performed subcellular localization. This screening step was performed in onion epidermal cells due to the ease of the bombardment procedure and visualization. Of the 31 fusion proteins, 12 sequences containing either canonical or non-canonical signals were found to target punctate structures that resemble peroxisomes when transformed (Supplementary Figures 2A–L). Having 12 potential positive localizations, the peroxisomal identity of these structures was determined by co-localization studies through co-transformation with PTS2-CFP derived from the peroxisomal protein gMDH (Figures 1A–L). One of these kinase derived constructs (PTD7, SHL>, Figure 1G; Supplementary Figure 1G; from AT3G61960) acted as an experimental positive control because it was shown previously to target to peroxisomes, but was only predicted by PPero (Ma and Reumann, 2008; Wang et al., 2017). The other 19 constructs appeared to remain in the cytosol and lacked any organelle or structure-like targeting (Supplementary Table 1; data not shown). This approach allowed us to identify 11 novel functional peroxisomal protein kinase C-terminal tripeptides (3 with SSL>, ARL>, SSL>, SCL>, 2 with TRL>, ASL>, SEL>, SSI>) (Supplementary Table 1; Figure 1) and confirm 1 previously studied protein kinase sequence that targets peroxisomes. Of the 12, 7 (three for PWM and 4 for PPero) were predicted, while five signals fell below the prediction thresholds, but were selected for study here (Supplementary Table 1; Figure 1). We will refer to them as protein kinases 1-12, or K1-K12.

**FIGURE 2:**
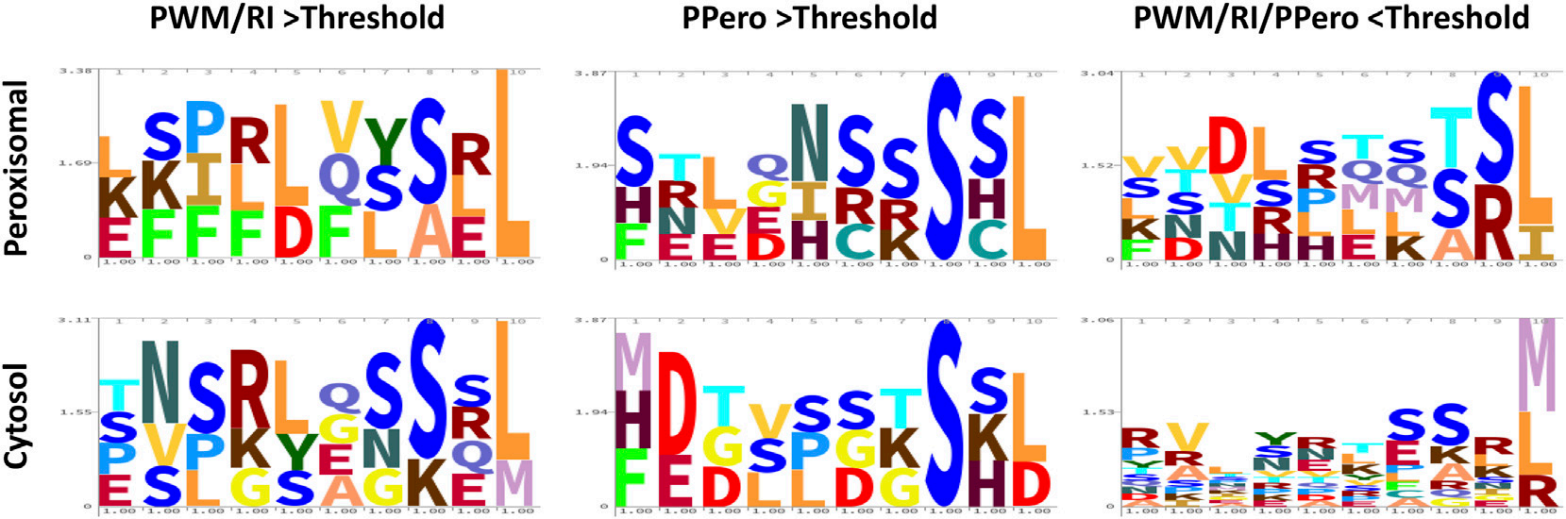
Logo plots for the peroxisomal versus cytosolic localized putative peroxisomal targeting domain (PTD) sequences of the 31 selected protein kinases. The 31 PTD sequences that were fused to EYFP are grouped according to their bioinformatic selection method and their final localization (peroxisome or cytosol). The height of each letter represents the frequency of the corresponding amino acid at each position. Logos were done using https://skylign.org/ (Wheeler et al., 2014). Peroxisomal refers to targeting of EYFP-PTD fusions to labelled peroxisomes *in vivo*, while cytosol refers to the accumulation of EYFP-PTD in the cytosol. Plots are based on the outcome of experiments shown in Figure 1 and Supplementary Figure 2. Details of sequence, scores and localizations are provided in Supplementary Table 1.

The sequence immediately N-terminal to the last three residues of the peroxisomal targeting protein includes the target enhancing or inhibiting elements and can affect targeting both positively and negatively and along with the last three residues is referred to as the PTD, or peroxisomal targeting domain. In general, basic residues, especially R, at positions -4 and -6 and proline (P) at -7 from the C-terminus enhance peroxisomal targeting (Lingner et al., 2011; Chowdhary et al., 2012; Reumann and Chowdhary, 2018). We analyzed the 31-protein kinase PTD sequences and compared the targeted (peroxisomal) versus non-targeted (non-peroxisomal) outcomes (Figure 2). What we did note was the prevalence of hydrophobic residues (V, L, I, F) in the peroxisomal targeted EYFP, in particular F. Also was the apparent negative targeting effect of G which appeared abundantly at positions -4, -5, -7 and -8 in sequences that did not localize to the peroxisome (Figure 2). Although this is a limited number of sequences, this analysis indicates that additional exploration of target elements is warranted, and refinement of prediction tools is important. It is also presumed that PTMs can enhance or block binding to the peroxisomal import receptor. For instance, phosphorylation has been detected in several peroxisomal proteins upstream of the PTS1 tripeptides (Kataya et al., 2019), and S, T, and Y are found in many peroxisomal PTD signals in the protein kinases explored here (Figure 2).

### Subcellular Localization of Full-Length Protein Kinases

**FIGURE 3:**
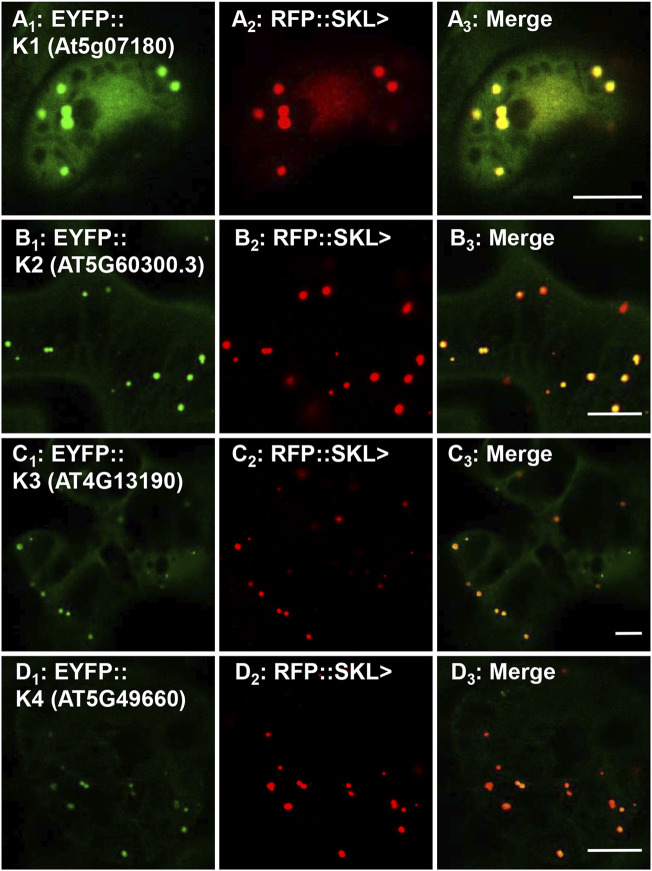
*In vivo* targeting of full-length RLK and RLCKs. *Nicotiana tabacum* cells were transformed with EYFP fusion constructs that were C-terminally fused with Arabidopsis protein kinase full-length cDNAs that are annotated as receptor-like or receptor-like cytosolic kinases. These RLK/RLCKs were shown to have functional PTS1s (Figure 1; Supplementary Table 1) and here their full-length fusion constructs targeted to punctate structures that coincided with RFP::SKL> in peroxisomes. Shown left to right are EYFP-kinase **(A**_**1**_ **)**, RFP::SKL signals **(A**_**2**_ **)**, and merge **(A**_**3**_ **)**. The protein kinase expressed as an EYFP fusion is shown in brackets (accession) and annotated K# as defined in Table 1. Representative images (produced by confocal microscopy) are shown. Scale bars are 5 μm.

**FIGURE 4:**
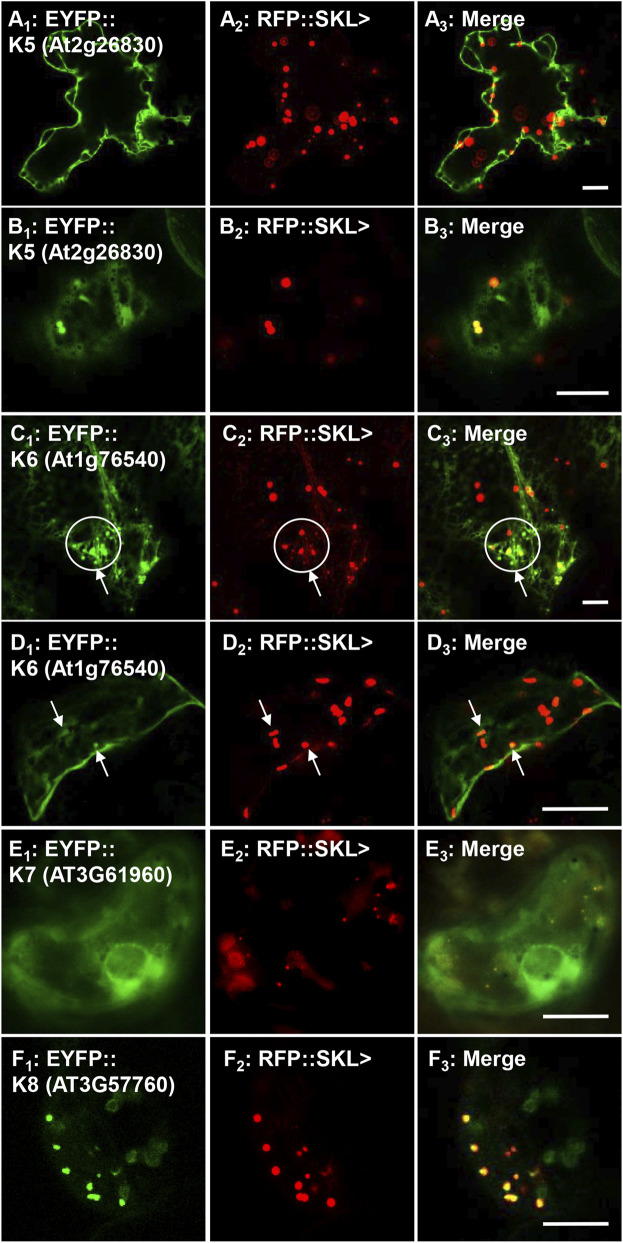
*In vivo* targeting of full-length soluble protein kinases. *Nicotiana tabacum* cells were transformed with EYFP fusion constructs that were C-terminally fused with Arabidopsis protein kinase full-length cDNAs. These protein kinases were shown to have functional PTS1s (Figure 1; Supplementary Table 1) and their full-length fusion constructs were targeted to either cytosol, punctate structures that coincided with RFP::SKL> in peroxisomes, or both. Shown left to right are EYFP-kinase **(A**_**1**_ **)**, RFP::SKL signals **(A**_**2**_ **)**, and merge **(A**_**3**_ **)**. The protein kinase expressed as an EYFP fusion is shown in brackets (accession) and annotated K# as defined in Table 1. Circles and arrows represent co-localized EYFP and RFP signals in peroxisomes in **C**_**1–3**_ and **D**_**1–3**_. Representative images (produced by confocal microscopy, except for E, which was done by epifluorescence microscopy) are shown. Scale bars are 5 μm.

To investigate the peroxisomal targeting of the full-length protein kinases with the newly identified functional peroxisomal signals, we amplified the cDNA from either cDNA-clones or RNA (Supplementary Table 3). Out of 12 candidates harboring functional PTDs, we were able to amplify and fuse eight full-length protein kinases N-terminally with EYFP. The EYFP-kinase fusions were first investigated in onion epidermal cells for their subcellular localization through biolistic transformations. Results indicated that four kinases (K1-K4) were found targeting to punctate structures (Supplementary Figures 3A–D; Table 1), and three (K5-K7) remained primarily in the cytosol (Supplementary Figures 3E–G; Table 1), while K8 targets both punctate structures and a region that is likely the nucleus (Supplementary Figure 3H; Table 1). To confirm or refute the results with onions, we switched to *Nicotiana tabacum* because, like Arabidopsis, tobacco is a dicot and has fewer, larger peroxisomes making co-localization studies easier. Subcellular localization in *Nicotiana tabacum* (Havana) leaves was performed through biolistic transformations in the presence of a peroxisomal marker (RFP-SKL>) using confocal (Figures 3, 4) and epifluorescence microscopy (Supplementary Figures 4, 5) in several independent experiments with similar outcomes. Using this approach, K1-K4 proved to be peroxisomal (Figures 3A–D; Supplementary Figures 4A–D; Table 1). In addition to peroxisomes, K3 (AT4G13190) appeared to target the nucleus as well (Supplementary Figure 4C).

In tobacco leaves the protein kinase K5 (At2g26830; Table 1) fusion targeted primarily to the cytosol and possibly to the nucleus (Figure 4A; Supplementary Figure 5A), but also partially coincided with the RFP-SKL in peroxisomes in different cell types (Figure 4B; Supplementary Figure 5B). The cyclin-dependent kinase, K6 (At1g76540; Table 1) was mostly localized to the cytosol and network-like structures, but also proved to partially target to peroxisomes (Figures 4C,D; Supplementary Figure 5C). Although having a functional and conserved PTS1, K7 (AT3G61960) failed to target to peroxisomes and remained in the cytosol and a network-like structure surrounding the nucleus, which resembles ER (Figure 4E; Supplementary Figure 5D) similar to a previous investigation (Ma and Reumann, 2008). K8 (AT3G57760) targeted to peroxisomes (Figure 4F; Supplementary Figure 5E) consistent with targeting punctate structures in the onion epidermal localization experiments (Supplementary Figure 3H). Our work supports the idea that seven full-length protein kinases can indeed target to peroxisomes and aligns with predicted functional PTDs, and PTS1 tripeptides (Figure 2).

### Re-Examination of Protein Kinases Predicted to Target Peroxisomes

**FIGURE 5:**
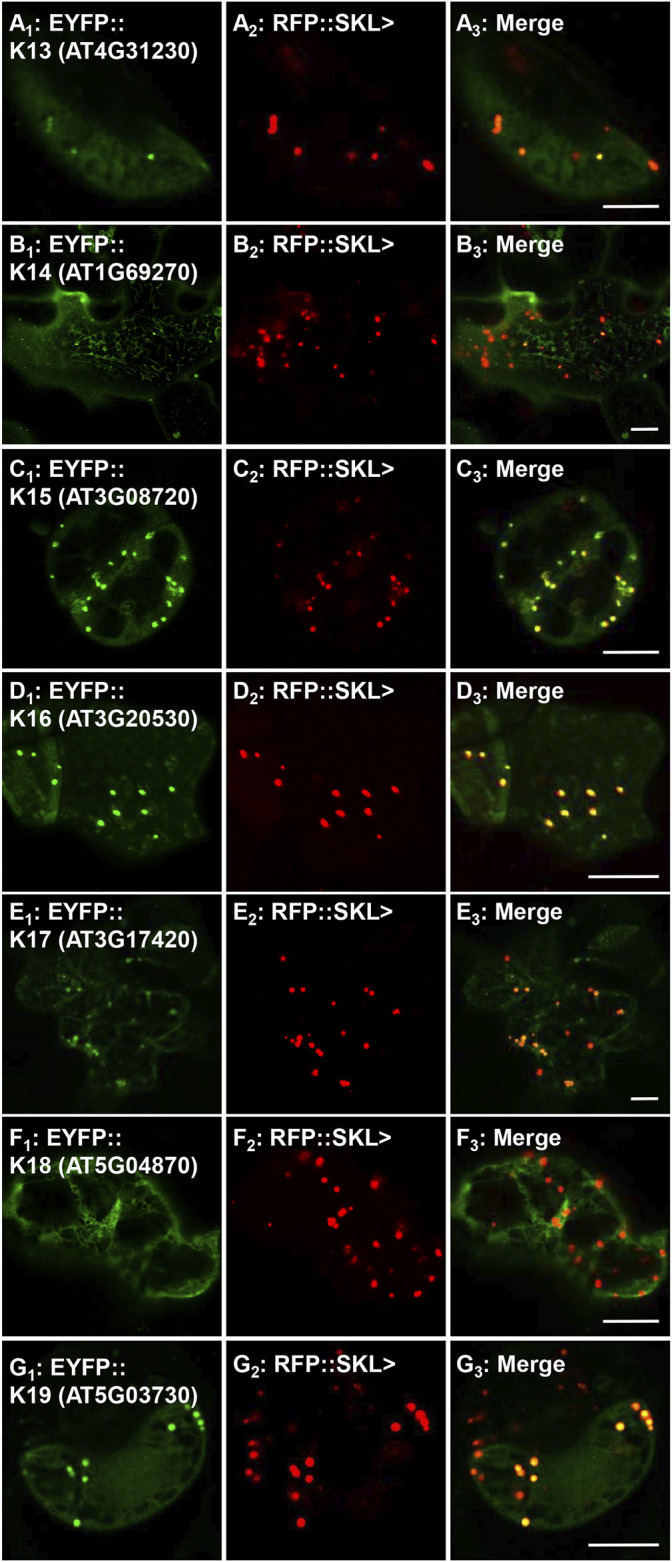
*In vivo* targeting of previously reported peroxisomal protein kinases. *Nicotiana tabacum* cells were transformed with EYFP fusion constructs that were C-terminally fused with Arabidopsis full-length cDNAs (K13-K19). These proteins were previously shown to have functional PTS1s and/or detected in isolated peroxisome proteomes (see Table 1 for details), but their full-length peroxisomal targeting was not previously validated. Shown left to right are EYFP-kinase **(A**_**1**_ **)**, RFP::SKL> signals **(A**_**2**_ **)**, and merge **(A**_**3**_ **)**. The protein kinase expressed as an EYFP fusion is shown in brackets (accession) and annotated K# as defined in Table 1. Representative images (produced by confocal microscopy) are shown. Scale bars are 5 μm.

Previously, three studies have investigated several putative peroxisomal protein kinases (Ma and Reumann, 2008; Lingner et al., 2011; Wang et al., 2017) and for completeness we re-explored targeting for several of these. Using our nomenclature, K13 (AT4G31230, PKL>), K14 (AT1G69270, SRL>), and K15 (AT3G08720.1, SNL>) were previously investigated (Table 1) and experimentally verified to harbor functional PTS1s (Ma and Reumann, 2008; Narsai et al., 2011). The kinase K16 (AT3G20530, SKL>) PTD, although not originally thought to target to peroxisomes (Ma and Reumann, 2008), was later reported to enter peroxisomes with high cytosolic background (Lingner et al., 2011; Wang et al., 2017). Although these protein kinases are also predicted by the updated algorithms (PWM, RI and PPero), in previous studies the full-length proteins were not associated with peroxisomes when investigated in onion epidermal cells, with the lack of any study for full-length K15 (Ma and Reumann, 2008). Due to the continuous improvement in peroxisome detection ability, we decided to re-study these full-length proteins. Interestingly, the fusion for K13 was noted in punctate structures but with a high cytosolic and nuclear background in onion epidermal cells (Supplementary Figure 3I). In tobacco leaves we were able to see different localization patterns for K13: only cytosolic (Supplementary Figure 6A), or nuclear and punctate structures (Supplementary Figure 6B), which coincided with the marker in peroxisomes (Figure 5A; Supplementary Figure 6C). K14 was non-peroxisomal in both plant systems consistent with previous studies (Figure 5B; Supplementary Figure 3J). The fusion protein K15 was detected in punctate structures and network-like structures in onion epidermal cells (Supplementary Figure 3K) and confirmed to localize in peroxisomes in tobacco leaves (Figure 5C; Supplementary Figure 6E). The fusion protein of K16 was detected in punctate structures in onion epidermal cells (Supplementary Figure 3L) and confirmed to be in peroxisomes in tobacco leaves (Figure 5D; Supplementary Figure 6F). In summary, we were able to verify the ability of three additional protein kinases to target to peroxisomes. This update is consistent with the finding of many conserved PTS1s in the orthologs of these protein kinases (Supplementary Figure 7). Results for full-length protein kinases are summarized in Table 1.

### Investigating GPK1, CPK1, and CTR1 Peroxisome Import

GPK1, CPK1 and constitutive triple response 1 (CTR1) have been previously implicated as peroxisomal protein kinases. K17 (GPK1, AT3G17420) was previously identified in the proteome of Arabidopsis glyoxysomes (specialized peroxisomes) (Fukao et al., 2003) and was localized in punctate structures in onion epidermal cells while studying its possible targeting by the predicted PTS1 AKI> (Ma and Reumann, 2008). Our full-length fusion for GPK1 also showed several punctate structures being targeting in onions (Supplementary Figure 3M). Although it did not coincide with RFP-SKL> in peroxisomes in many tobacco leaf cells, we demonstrate peroxisomal targeting in tobacco mesophyll and guard cells (Figure 5E; Supplementary Figure 6G).

K18, calcium-dependent protein kinase 1 (CPK1), was previously reported to target to peroxisomes and lipid bodies in an N-myristoylation dependent-fashion (Dammann et al., 2003; Coca and San Segundo, 2010; Kataya et al., 2019). CPK1 was also found to have a predicted PTS1 (LKL>), which was verified experimentally to target to peroxisomes (Lingner et al., 2011). To get a comprehensive overview about CPK1-peroxisomal import, we fused CPK1 N-terminally with EYFP in order to block peroxisomal localization by N-myrsitoylation, and to study if CPK1 can also target to peroxisomes in a PTS1-dependent manner. The fusion remained in the cytosol and network-like structures in both onions and tobacco (Supplementary Figures 3N, 6H; Figure 5F). This implies that K18, although having a functional PTS1, relies on N-myristoylation for peroxisome membrane targeting.

K19, (CTR1; SDL>) harbors an atypical predicted PTS1 due to the presence of N-terminal target enhancing residues (Chowdhary et al., 2012). The full-length protein was detected in punctate structures in onion epidermal cells (Supplementary Figure 3O), and in peroxisomes in tobacco (Figure 5G). Although peroxisomal targeting was reproducible, many transformed cells displayed a different localization pattern (Supplementary Figure 6I), which raises the question if CTR1, like GPK1 and some other kinases studied here, are targeting peroxisomes under specific cellular conditions, which might affect PTS1 signal exposure and binding to the PEX5 peroxisome import receptor.

### Sequence, Phylogenetic, and Expression Analysis of the Peroxisomal Protein Kinases

The Arabidopsis protein kinases have been catalogued and a recent sequence and phylogenetic analyses divided the protein kinases into so called “soluble”, “receptor-like” (RLK; have a transmembrane and extracellular domain) and “receptor-like but lacking a transmembrane and extracellular domain, therefore likely cytosolic” (RLCK), plus a few non-categorized protein kinases (Zulawski et al., 2014). Mapping K1-K19 onto this tree shows the peroxisomal kinases are distributed broadly in each group (soluble, RLK and RLCK). Table 1 is a list of the 19 peroxisome protein kinases studied here (12 identified in this study plus 7 previously studied). Most of these kinases harbor a non-canonical PTS1 (Figure 2; Supplementary Figure 8). These kinases belong to the subclades/superfamilies LRR clade 1, 2, 3, 4, and 11, RLCK 9, RLCK clade2, L-LPK, mixed clade 1, CDK and AGC (Zulawski et al., 2014). To gain potential insight into the function of each peroxisomal protein kinase we generated a phylogenetic tree with the sequences of K1-K19 plus 86 protein kinases with defined roles (Zulawski et al., 2014). This is presented in Supplementary Figure 9. It is interesting to note that K6 (cyclin-dependent kinase B2/ CDKB2) resides nearest CDKA1 and both are described as regulators of mitosis and RNA polymerase II CTD heptapeptide repeat kinases. K12 groups with CDKF1, which is in fact a CDK activating kinase (CAK) that phosphorylates and activates CDKs, suggesting that K12 may be K6’s (CDKB2) activating kinase in the peroxisome. Two other groupings of interest are K3 and K16 which sit near the protein kinase PBS1 that is involved in PAMP-triggered immunity signaling and defense responses downstream of FLS2 (Rao et al., 2018).

Different factors and conditions are expected to influence protein kinase targeting and the exposure of PTS1 to the PEX5 peroxisome receptor for import into peroxisomes. Therefore, it is of importance to study the PTMs and expression of these kinases to ultimately decipher their roles. In addition to K18 (CPK1), K12 (AT1G74330) has a predicted N-myristoylation site (Zulawski et al., 2014). Several of these protein kinases are phosphorylated at multiple sites with K13 having 22 mapped phospho-sites. Four kinases (K15, K16, K17 and K19/CTR1) are phosphorylated in their activation loops, and several phosphopeptides appear to be tissue specific (Zulawski et al., 2014; Mergner et al., 2020). The transcripts of these kinases are found in most tissues and developmental stages as seen in the public GENEVESTIGATOR (microarray, Supplementary Figures 10, 11), and ATHENA (RNAseq/proteome) databases (Zimmermann et al., 2008; Mergner et al., 2020). In addition, several kinase transcripts are found either induced or suppressed in response to external perturbations such as biotic and abiotic stresses (Supplementary Figure 12). Peroxisomes are now known to be involved in several stress response pathways through the synthesis of a jasmonic acid (JA) precursor via 12-oxophytodienoate reductase isoform 3 (OPR3) and β-oxidation, and salicylic acid (SA) signaling and antifungal defense through the peroxisomal enzyme PEN2 myrosinase (reviewed in (Kataya et al., 2020b)). Several protein kinases (K11, K12, K14, and K15) show higher expression, not only under fungal and bacterial infections, but also when plants are exposed to different elicitors such as FLG22 and GST-NPP1 and to SA (Supplementary Figure 12), suggesting these kinases are involved in peroxisome-dependent defense responses. K13 and K16 gene expression are specifically upregulated upon the treatment of stamens of *opr3* plants with methyl jasmonate and 12-oxophytodienoic acid (Supplementary Figure 12), suggesting a regulatory role for these kinases in jasmonic acid signaling, involving peroxisomes.

### Isolating Kinase Knockout Mutants and Investigating Links to Fatty Acid β-Oxidation

**FIGURE 6:**
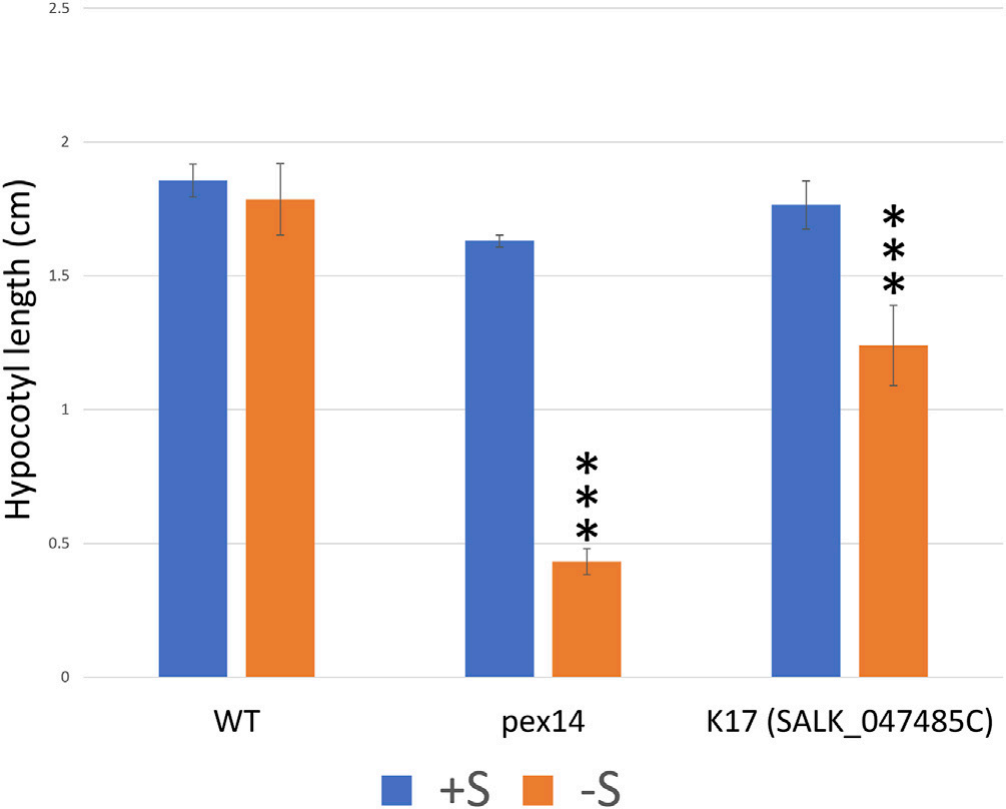
The T-DNA knock-out lines of K17 (GPK1) seedlings show sugar dependency. To investigate the potential role of the peroxisomal protein kinases in fatty acid β-oxidation, we performed a sucrose dependence assay on selected homozygous lines (details of the lines and the results are summarized in Supplementary Table 4). Hypocotyl length (cm) of seedlings grown for 1 day in light followed by 5 days in the dark on one-half-strength Murashige and Skoog (MS) medium with or without 1% sucrose. Only, K17 (SAIL_1250_C08) mutant seedlings show significant sucrose dependence phenotypes when compared to WT (Supplementary Table 4, data not shown). To further confirm the K17 knockout mutant phenotype, an additional T-DNA mutant “K17_SALK_047485” seedling was investigated and displayed a significant sugar dependence phenotype. The *PEX14* mutant control seedlings also show a significant sucrose dependence phenotype when compared to WT. Columns marked with three stars are significantly different from WT at *p* < 0.01 (Student’s *t*-test). The experiments were repeated three times with more than 50 seedlings; error bars represent standard deviation.

Fatty acid β-oxidation is important for seedling development, and growth is halted in mutants deficient in this process, unless exogenous sucrose is provided. As most β-oxidation enzymes are phosphorylated (Kataya et al., 2019) and protein phosphatase 2A (PP2A) was shown to have a potential role in regulating the β-oxidation proteins KAT1 and LACS5 (Kataya et al., 2015b), we were keen to investigate if any of the peroxisomal protein kinases are involved in regulating fatty acid β-oxidation. We genotyped T-DNA insertion lines and isolated homozygous plants for 17 lines (see Supplementary Table 4 for full summary). Subsequently, the homozygous mutants were used to assess hypocotyl elongation defects that could be ameliorated by addition of sucrose. The basis of the screen is germination and growth (measured by hypocotyl elongation) in the dark being dependent on the mobilization of stored triacylglycerol via fatty acid β-oxidation, a peroxisomal metabolic process. Protein kinases that are regulators of β-oxidation in the peroxisome may yield a phenotype in insertional lines. Growth on sucrose will bypass the need for β-oxidation in the dark. In dark conditions, on sucrose-free medium, hypocotyl elongation was retarded in one protein kinase-mutant (K17) relative to sucrose-containing medium and compared to WT (Supplementary Table 4, data not shown). This implicates a potential role for K17 in regulating fatty acid β-oxidation. K17 (GPK1) was identified as peroxisomal (Figure 5E) and was previously identified in the glyoxysome proteome (Fukao et al., 2003). We confirmed the effect of K17 with a second homozygous line (Figure 6; K17 Salk). PEX14 with a nonsense mutation was used as a control because it lacks the ability to import β-oxidation enzymes, and thus requires sucrose to support hypocotyl elongation in the dark (Hayashi et al., 2000; Zhang and Hu, 2010; Kataya et al., 2015a).

CPK1 (K18) is targeted to the membranes of oil bodies and peroxisomes, and previously has been implicated in regulating triacylglycerol degradation in oil bodies, fatty acid β-oxidation in peroxisomes, and possible crosstalk between these organelles during early germination (Dammann et al., 2003; Coca and San Segundo, 2010; Kataya et al., 2019). To test this, we obtained two CPK1 mutants (*cpk1-1* (Salk_007698); *cpk1-2* (Salk_080155)) to examine the effects of sucrose on germination. No significant differences were observed between control and sucrose-containing media, in both CPK1 lines when compared to WT (data not shown). This indicates that CPK1 is likely not involved in regulating early germination.

## Discussion

### Identifying Functional Protein Kinase PTS1 Signals

Over the past 2 decades, a combination of methods (proteomics, prediction algorithms, and subcellular verification tools) has helped identify ∼200 peroxisomal proteins and suggested many new functions for plant peroxisomes, as compiled in recent reviews (Pan and Hu, 2018; Kataya et al., 2019). We have established protein phosphorylation as a regulatory mechanism in Arabidopsis peroxisomes by cataloging over 100 peroxisomal phosphoproteins (Kataya et al., 2019). We identified four peroxisomal protein phosphatases and predict a higher number of peroxisomal protein kinases based on the genomic ratio of protein phosphatases to kinases (Matre et al., 2009; Kataya et al., 2015b; 2016). To initiate exploring the peroxisome kinome we employed three prediction algorithms (PWM, RI, and PPero) and identified a group of putative peroxisomal protein kinases, which harbor non-canonical PTS1s. This approach generated a list of 31 putative peroxisomal protein kinases (Figure 2; Table 1). Although twelve protein kinase-PTDs proved to be functional *in vivo* we cannot eliminate the possibility that the other PTDs target their parent proteins to peroxisomes under unique cellular conditions to promote this shuttling. Consistent with this idea, targeting efficiency varied for all identified functional PTDs harboring non-canonical PTS1s. For example, PTDs 3, 4, 6, 8, 9 and 12 show higher cytosolic than peroxisomal localization implicating a weaker targeting efficiency (Figure 1; Supplementary Figure 2). Targeting strength has been correlated with the ability to bind to the peroxisomal PEX5 receptor and this in turn is controlled by the target inhibiting and enhancing elements located upstream of the PTS1 (Reumann and Chowdhary, 2018). In some cases, the prediction tools did identify functional non-canonical PTS1s and failed to predict others. This emphasizes the need of using multiple approaches to define the putative peroxisomal proteome using a combination of prediction methods, searching for non-canonical PTS1s, and PTS1 conservation in multiple (plant) species. By increasing our knowledge about peroxisomal proteins, the prediction tools could be trained with additional positive datasets, which hopefully could further improve their prediction accuracy.

### Testing Peroxisomal Targeting of Full-Length Protein Kinases

From the 12 newly identified PTS1 signals, we confirmed the ability of 7 full-length protein kinases (K1-K6 and K8) to target to peroxisomes as predicted. We also were able to re-examine a group of additional protein kinases that harbor functional PTS1 tripeptides (K13-16, K18, and K19/CTR1) or were previously detected in the peroxisomal proteome (K17) (Fukao et al., 2003; Ma and Reumann, 2008; Lingner et al., 2011; Wang et al., 2017), from which we were able to provide evidence for five full-length protein kinases (K13, K15-17, and K19/CTR1) having the ability to target to peroxisomes *in vivo*. Although having canonical PTS1s, full-length K13 and K16 were previously reported to reside in the cytosol (Ma and Reumann, 2008). Conversely, we show these protein kinases do target to peroxisomes, although weakly in onion cells, but more pronounced in tobacco cells.

Although having a predicted PTS1, K17 (GPK1) was the only protein kinase that had a non-functional PTS1 but has been found in the glyoxysome proteome (Fukao et al., 2003; Ma and Reumann, 2008). As previously reported, full-length K17/GPK1 targeted to punctate structures in onion epidermal cells (Supplementary Figure 3M) but these failed to coincide with peroxisomes or mitochondria (Ma and Reumann, 2008). Although GPK1 might be localizing to peroxisomes by the mPTS/PEX19 pathway (Sacksteder et al., 2000), it could be localizing to or moving through intermediate vesicles that did not fuse with peroxisomes in onion cells. This is supported by its localization in tobacco, where it was found to target to peroxisomes in some cells but not others. Also, GPK1 has two predicted glycosylation sites that are possibly modified in the ER to be exported in vesicles which could fuse with peroxisomes under specific conditions. This hypothesis fits with glycosylation and ER-to-peroxisome transport currently established for some peroxisomal membrane proteins (PMPs) (Buentzel and Thoms, 2017). For instance, PEX2 and PEX16 from *Yarrowia lipolytica* were reported in the ER-to-peroxisome pathway and are glycosylated (Titorenko and Rachubinski, 1998; Kim and Hettema, 2015). Similarly, Arabidopsis purple acid phosphatase 7 (PAP7) has been shown to follow the same pathway and has a predicted glycosylation site (Kataya et al., 2016). In our study, we also provide evidence that K18 (CPK1) uses N-myristoylation for peroxisomal targeting, rather than its functional PTS1.

Subcellular targeting within the eukaryotic cell is governed by a sophisticated suite of protein-protein interactions specific to the subcellular compartment. It is precisely this specificity that prevents mistargeting of proteins. The flexibility in the protein kinases PTS1 amino acid substitutions likely indicates functional specificity, but we cannot eliminate the possibility that these changes are functionally irrelevant or are an evolutionary artifact. Determining the relevance of these substitutions is the arduous challenge of the plant cell biology community. From our new and re-examined peroxisomal protein kinases, we developed an inventory of 19 protein kinases that are associated with Arabidopsis peroxisomes (Table 1) with some having a canonical PTS1, but with the majority having a non-canonical PTS1 (Supplementary Figure 8). The prevalence of non-canonical and weak targeting PTS1s in the peroxisomal kinases is possibly due to a need for weak binding with PEX5 to prevent over-accumulation or to allow proper folding inside peroxisomes. This idea is supported by catalase, which needs a weak PTS1 for proper targeting, and if changed to a strong PTS1 (SKL>) leads to accumulation of catalase protein aggregates (Williams et al., 2012).

The 19 protein kinases from this study are predicted by SUBA to target plasma membrane, extracellular, cytosol, or nucleus (Hooper et al., 2016). We believe that the protein kinases K1-6, K8, K13, K15-16, and K19 are targeting to peroxisomes, because their C-terminal sequences acts as PTS1 in reporter-domain fusions as shown in this study and previous reports (Ma and Reumann, 2008; Wang et al., 2017) and the full-length proteins were found in peroxisomes. However, in some of these proteins the mechanism of transport is not clear, because kinases K1-4 encode transmembrane domain and kinase K16 is predicted to be extracellular. Previous reporter fusion studies have only shown K1 in the plasma membrane, and K6 and K15 in cytosol and nucleus, while proteomics indicated the presence of K3 in the plasma membrane, and K8 that has no predicted chloroplast signal in the plastid (Hooper et al., 2016). We believe that proteins K1-4 and K16 can become imported into the peroxisomal lumen (despite the predicted transmembrane domains) upon recognition of the PTS1 by PEX5 in the cytosol and the alternative signals could allow transport to different subcellular locations. However, we cannot exclude other possible alternative routes similar to the reports that show proteins harboring transmembrane domains and alternative N-terminal signals are imported into peroxisomes in a PTS1-dependent fashion. For example, purple acid phosphatase 7 that has an N-terminal hydrophobic/transmembrane stretch, targets peroxisomes through the ER in a PTS1-dependent fashion (Kataya et al., 2016). Also, a peroxisomal protein phosphatase 2C has been shown to target mitochondria through its N-terminal mitochondrial signal, this is followed by a transmembrane segment necessary for proteolytic processing in the intermembrane space. It is proposed that the remainder of the protein phosphatase 2C, which has a C-terminal PTS1, is now localized to peroxisomes (Stehlik et al., 2020). Moreover, we cannot exclude that a PTS1-independent mechanisms might be responsible for the transport of those proteins harboring a transmembrane as has been described for GPK1 and might be mediated by the mPTS1/PEX19 pathway. If these 19 kinases are indeed present in peroxisomes, conditionally targeting to peroxisomes, dual- or multi-targeted to cell compartments remains unknown.

### Linking Peroxisomal Protein Kinases to Cell Function

The Arabidopsis peroxisomal protein kinases have previously been grouped into receptor kinases (RLKs), receptor-like cytoplasmic kinases (RLCKs), soluble and unclassified kinases (Supplementary Figure 9). Here, K1-2, 4, 9–11, 13–14 are RLKs, and 16–17 RLCKs (Supplementary Figure 9). Transcripts for two RLKs, K1 and K11, were downregulated and upregulated, respectively, in response to biotic stress and different elicitors (Supplementary Figure 12). K1 and K11 reside in the same clade with several leucine-rich repeat receptor kinases (LRR-RKs) including the pattern recognition receptors EFR and FLS2, which might imply importance in peroxisome signaling and crosstalk with other organelles and pathways (Supplementary Figure 9). This also aligns with the prediction and experimental verification of a number of resistance proteins of the nucleotide-binding site leucine-rich repeat (NB-LRR) protein family targeting peroxisomes, and may play major roles in signaling during innate immunity (Kataya, 2011; Sorhagen et al., 2013). The RLCKs, K3 (PBL23) and K16 (PBL24), group together during phylogenetic analysis (Supplementary Figure 9) and belong to the RLCKVII subfamily (Supplementary Figure 9). Both K3 and K16 are members of the PBS1-like proteins (PBL), which is a group of 47 proteins primarily associated with innate immunity (Lu et al., 2010; Rao et al., 2018). Why both kinases can target to peroxisomes is unclear. Taken together, both RLKs and NB-LRR proteins might constitutively or transiently target to peroxisomes during stress conditions to perform strategic functions needed for the peroxisomal role during innate immunity responses (Kataya, 2011). The peroxisomal POL-like phosphatase 3 is expressed in leaves only after *Pseudomonas syringe* infection (Kataya et al., 2016), implicating an induction mechanism for (at least some) peroxisomal phosphoregulatory proteins during innate immunity.

CPK1, a soluble kinase, has been associated with peroxisomes and is known to have a stress-response role based upon gene induction by fungal elicitors and susceptibility to both biotic and abiotic stresses when knocked out (Coca and San Segundo, 2010). Although CPK1 targeted to peroxisomes and lipid bodies, mutant *cpk1-1* seedlings did not show sugar dependence (Cassin-Ross and Hu, 2014). We produced similar results for both *cpk1-1* and *cpk1-2* and show that CPK1 localization to peroxisomes is PTS1-independent. CPK1’s role in JA-signaling pathways has been proposed because over-expresser plants show higher expression of genes needed for OPDA synthesis (Coca and San Segundo, 2010). Also, the peroxisomal proteins OPR3 and benzoate-CoA ligase, involved in JA and SA synthesis, respectively, are phosphorylated (Kataya et al., 2019). Therefore, it will be intriguing to investigate OPR3 and/or benzoate-CoA ligase phosphorylation status in *CPK1* mutants or the other peroxisomal kinases (K13 and K16) mutants.

We demonstrate that protein kinase K19/CTR1 targets to peroxisomes (Figure 5G). We speculate that placing the fluorophore on the N-terminus to expose the C-terminal PTD, may have blocked ER localization, but also allowed recognition of the PTD by the peroxisome import machinery. The CTR1 PTD may normally be masked letting the enzyme reside in the ER, then under certain conditions, it is exposed to allow peroxisome import. Indeed, CTR1 was suggested to be quarantined in peroxisomes under stress, which could be an alternative strategy for the cell to regulate stress responses (Sorhagen et al., 2013; Kataya et al., 2020b). K6 (CDKB2-1), a soluble cyclin-dependent protein kinase, partially targets peroxisomes and is annotated to be involved in the regulation of cell division (Gutierrez, 2009). Peroxisomes are known to also divide and increase in number under stress. Mitogen-Activated Protein Kinase17 (MPK17) was shown to affect peroxisomal proliferation under abiotic stress (Frick and Strader, 2018) and emphasizes the presence of a phosphorylation-dependent mechanism controlling peroxisome proliferation. Future studies could use our results to investigate if K6 is one of the regulators of this process.

Our results demonstrate that many more protein kinases can target the peroxisome than previously thought and that more variant, but functional PTD sequences exist. This leads us to speculate that the protein kinases studied here, perhaps additional protein kinases and even other proteins could reside in the peroxisome (or be excluded) depending on cellular conditions, cell or tissue types. Perhaps this is not surprising given the abundance of protein phosphorylation in the peroxisome, the growing list of peroxisome functions and the dynamic nature of this organelle (Kataya et al., 2016).

### GPK1 (K17) Is Associated With Fatty Acid β-Oxidation

The reduced ability of K17/GPK1 mutant line to elongate their hypocotyls in the dark unless provided with sucrose implicates K17/GPK1 protein kinase as a regulator of fatty acid β-oxidation. Phosphorylation of glyoxosomal proteins was reported biochemically to occur in the presence of various metal ions and ATP (Fukao et al., 2003), and the majority of fatty acid β-oxidation and glyoxylate cycle proteins are phosphorylated (Kataya et al., 2019). The seedlings of the protein phosphatase knockouts *pp2a b′θ* and *pll3-7* have shown a sugar-dependence phenotype during early germination with a mild effect on auxin metabolism for PP2A (Kataya et al., 2015b, 2016; Kataya et al., 2019). Here, we also show a sugar-dependence phenotype for K17/GPK1 mutant seedlings implying a possible function of this protein kinase in fatty acid β-oxidation. Fukao et al. (2003), have shown K17/GPK1 is a peripheral membrane protein (with a kinase domain facing the peroxisome lumen) and phosphorylates an as yet unidentified 40 kDa peroxisomal protein. In the same study, they confirmed KAT1 to be phosphorylated, and this was recently detected in the mutant seedlings of *pp2a-b′θ*, but not WT (Fukao et al., 2003; Kataya et al., 2015b). K17/GPK1 is phosphorylated (Mergner et al., 2020), has autophosphorylation activity (Nemoto et al., 2011), and was found physically to interact with E3 ubiquitin-protein ligase SINAT3 in a Y2H screen (Arabidopsis Interactome Mapping, 2011). K17/GPK1 expression is upregulated during early germination like most fatty acid β-oxidation and glyoxylate cycle enzymes (Supplementary Figure 12). Notably, the K17/GPK1 mutant phenotype was not particularly strong compared with other mutants disrupting β-oxidation processes, including PEX14 (Figure 6F). Our view is that knockout of an enzyme in the β-oxidation process should give a dramatic effect (as expected), but a loss of an enzyme that covalently modifies one or several proteins in the process of β-oxidation would likely give a less pronounced phenotype for a variety of reasons. Firstly, if a protein kinase phosphorylates one or several proteins, we do not know the impact or effect until the biochemistry can be done. Secondly, if phosphorylation directly affects activity, it is likely that this is not a complete on/off switch for enzyme activity, and thus we would expect a less dramatic phenotype than knocking out a β-oxidation enzyme. It will be interesting in the future to decipher the role of K17/GPK1 in β-oxidation.

## Data Availability

The datasets presented in this study can be found in online repositories. The names of the repository/repositories and accession number(s) can be found in the article/Supplementary Material.

## References

[B1] Agrawal G. Subramani S. (2016). De Novo peroxisome Biogenesis: Evolving Concepts and Conundrums. Biochim. Biophys. Acta (Bba) - Mol. Cel Res. 1863, 892–901. 10.1016/j.bbamcr.2015.09.014 PMC4791208 26381541

[B2] Alberts M. E. Chua G. Muench D. G. (2019). Exposure to Naphthenic Acids and the Acid Extractable Organic Fraction from Oil Sands Process-Affected Water Alters the Subcellular Structure and Dynamics of Plant Cells. Sci. Total Environ. 651, 2830–2844. 10.1016/j.scitotenv.2018.10.181 30463136

[B3] Arabidopsis Interactome Mapping C. (2011). Evidence for Network Evolution in an Arabidopsis Interactome Map. Science 333, 601–607. 10.1126/science.1203877 21798944 PMC3170756

[B4] Booker M. A. Delong A. (2017). Atypical Protein Phosphatase 2A Gene Families Do Not Expand via Paleopolyploidization. Plant Physiol. 173, 1283–1300. 10.1104/pp.16.01768 28034953 PMC5291013

[B5] Buentzel J. Thoms S. (2017). The Use of Glycosylation Tags as Reporters for Protein Entry into the Endoplasmic Reticulum in Yeast and Mammalian Cells. in Peroxisomes: Methods and Protocols. Editor Schrader M. (New York, NY: Springer New York), 221–232. 10.1007/978-1-4939-6937-1_21 28409466

[B6] Bussell J. D. Behrens C. Ecke W. Eubel H. (2013). Arabidopsis Peroxisome Proteomics. Front. Plant Sci. 4, 101. 10.3389/fpls.2013.00101 23630535 PMC3633942

[B69] Cassin-Ross G. Hu J. (2014). Systematic Phenotypic Screen of Arabidopsis Peroxisomal Mutants Identifies Proteins Involved in β-Oxidation. Plant Physiol. 166, 1546–1559. 10.1104/pp.114.250183 25253886 PMC4226370

[B7] Chowdhary G. Kataya A. R. Lingner T. Reumann S. (2012). Non-canonical Peroxisome Targeting Signals: Identification of Novel PTS1 Tripeptides and Characterization of Enhancer Elements by Computational Permutation Analysis. BMC Plant Biol. 12, 142. 10.1186/1471-2229-12-142 22882975 PMC3487989

[B8] Coca M. San Segundo B. (2010). AtCPK1 Calcium-dependent Protein Kinase Mediates Pathogen Resistance in Arabidopsis. Plant J. 63, 526–540. 10.1111/j.1365-313x.2010.04255.x 20497373

[B9] Dammann C. Ichida A. Hong B. Romanowsky S. M. Hrabak E. M. Harmon A. C. (2003). Subcellular Targeting of Nine Calcium-dependent Protein Kinase Isoforms from Arabidopsis. Plant Physiol. 132, 1840–1848. 10.1104/pp.103.020008 12913141 PMC181270

[B10] Frick E. M. Strader L. C. (2018). Kinase MPK17 and the Peroxisome Division Factor PMD1 Influence Salt-Induced Peroxisome Proliferation. Plant Physiol. 176, 340–351. 10.1104/pp.17.01019 28931630 PMC5761782

[B11] Fukao Y. Hayashi M. Hara-Nishimura I. Nishimura M. (2003). Novel Glyoxysomal Protein Kinase, GPK1, Identified by Proteomic Analysis of Glyoxysomes in Etiolated Cotyledons of *Arabidopsis thaliana* . Plant Cel Physiol 44, 1002–1012. 10.1093/pcp/pcg145 14581625

[B12] Fukao Y. Hayashi M. Nishimura M. (2002). Proteomic Analysis of Leaf Peroxisomal Proteins in Greening Cotyledons of *Arabidopsis thaliana* . Plant Cel Physiol 43, 689–696. 10.1093/pcp/pcf101 12154131

[B13] Fulda M. Shockey J. Werber M. Wolter F. P. Heinz E. (2002). Two Long-Chain Acyl-CoA Synthetases fromArabidopsis Thalianainvolved in Peroxisomal Fatty Acid β-oxidation. Plant J. 32, 93–103. 10.1046/j.1365-313x.2002.01405.x 12366803

[B14] Gould S. G. Keller G. A. Subramani S. (1987). Identification of a Peroxisomal Targeting Signal at the Carboxy Terminus of Firefly Luciferase. J. Cel Biol 105, 2923–2931. 10.1083/jcb.105.6.2923 PMC2114716 3480287

[B15] Gutierrez C. (2009). The Arabidopsis Cell Division Cycle. The Arabidopsis Book 7, e0120. 10.1199/tab.0120 22303246 PMC3243301

[B16] Hayashi M. Nito K. Toriyama-Kato K. Kondo M. Yamaya T. Nishimura M. (2000). AtPex14p Maintains Peroxisomal Functions by Determining Protein Targeting to Three Kinds of Plant Peroxisomes. EMBO J. 19, 5701–5710. 10.1093/emboj/19.21.5701 11060021 PMC305803

[B17] Hooper C. M. Castleden I. R. Tanz S. K. Aryamanesh N. Millar A. H. (2016). SUBA4: the Interactive Data Analysis centre for Arabidopsis Subcellular Protein Locations. Nucleic Acids Res. 45, D1064–D1074. 10.1093/nar/gkw1041 27899614 PMC5210537

[B18] Hu J. Baker A. Bartel B. Linka N. Mullen R. T. Reumann S. (2012). Plant Peroxisomes: Biogenesis and Function. Plant Cell 24, 2279–2303. 10.1105/tpc.112.096586 22669882 PMC3406917

[B19] Jaipargas E. A. Mathur N. Bou Daher F. Wasteneys G. O. Mathur J. (2016). High Light Intensity Leads to Increased Peroxule-Mitochondria Interactions in Plants. Front Cel Dev Biol 4, 6. 10.3389/fcell.2016.00006 PMC4740372 26870732

[B20] Jossier M. Liu Y. Massot S. Hodges M. (2019). Enzymatic Properties of Recombinant Phospho-Mimetic Photorespiratory Glycolate Oxidases from *Arabidopsis thaliana* and Zea mays. Plants (Basel) 9. 10.3390/plants9010027 PMC7020226 31878154

[B21] Jung S. Marelli M. Rachubinski R. A. Goodlett D. R. Aitchison J. D. (2010). Dynamic Changes in the Subcellular Distribution of Gpd1p in Response to Cell Stress. J. Biol. Chem. 285, 6739–6749. 10.1074/jbc.m109.058552 20026609 PMC2825468

[B22] Kao Y.-T. Gonzalez K. L. Bartel B. (2018). Peroxisome Function, Biogenesis, and Dynamics in Plants. Plant Physiol. 176, 162–177. 10.1104/pp.17.01050 29021223 PMC5761812

[B23] Kataya A. R. A. (2011). in Identification of Peroxisome-Targeted Proteins Implicated in Plant Innate Immunity in *Arabidopsis thaliana* . Editor Reumann S. (Norway): University of Stavanger).

[B24] Kataya A. R. A. Elshobaky A. Heidari B. Dugassa N.-F. Thelen J. J. Lillo C. (2020a). Multi-targeted Trehalose-6-Phosphate Phosphatase I Harbors a Novel Peroxisomal Targeting Signal 1 and Is Essential for Flowering and Development. Planta 251, 98. 10.1007/s00425-020-03389-z 32306103 PMC7214503

[B25] Kataya A. R. A. Heidari B. Hagen L. Kommedal R. Slupphaug G. Lillo C. (2015a). Protein Phosphatase 2A Holoenzyme Is Targeted to Peroxisomes by Piggybacking and Positively Affects Peroxisomal β-Oxidation. Plant Physiol. 167, 493–506. 10.1104/pp.114.254409 25489022 PMC4326747

[B26] Kataya A. R. A. Hu J. Muench D. G. Moorhead G. B. (2020b). “Plant Peroxisomal Protein Kinases Implicated in Stress‐Related Responses,” in Protein Kinases and Stress Signaling in Plants, 501–517. 10.1002/9781119541578.ch22

[B27] Kataya A. R. A. Muench D. G. Moorhead G. B. (2019). A Framework to Investigate Peroxisomal Protein Phosphorylation in Arabidopsis. Trends Plant Sci. 24, 366–381. 10.1016/j.tplants.2018.12.002 30683463

[B28] Kataya A. R. A. Schei E. Lillo C. (2015b). MAP Kinase Phosphatase 1 Harbors a Novel PTS1 and Is Targeted to Peroxisomes Following Stress Treatments. J. Plant Physiol. 179, 12–20. 10.1016/j.jplph.2015.03.002 25817413

[B29] Kataya A. R. A. Schei E. Lillo C. (2016). Towards Understanding Peroxisomal Phosphoregulation in *Arabidopsis thaliana* . Planta 243, 699–717. 10.1007/s00425-015-2439-5 26649560

[B30] Kim P. K. Hettema E. H. (2015). Multiple Pathways for Protein Transport to Peroxisomes. J. Mol. Biol. 427, 1176–1190. 10.1016/j.jmb.2015.02.005 25681696 PMC4726662

[B31] Kumar S. Stecher G. Li M. Knyaz C. Tamura K. (2018). MEGA X: Molecular Evolutionary Genetics Analysis across Computing Platforms. Mol. Biol. Evol. 35, 1547–1549. 10.1093/molbev/msy096 29722887 PMC5967553

[B32] Kunze M. (2020). The Type-2 Peroxisomal Targeting Signal. Biochim. Biophys. Acta (Bba) - Mol. Cel Res. 1867, 118609. 10.1016/j.bbamcr.2019.118609 31751594

[B33] Li-Beisson Y. Shorrosh B. Beisson F. Andersson M. X. Arondel V. Bates P. D. (2013). Acyl-lipid Metabolism. The Arabidopsis Book 11, e0161. 10.1199/tab.0161 23505340 PMC3563272

[B34] Lillo C. Kataya A. R. A. Heidari B. Creighton M. T. Nemie‐feyissa D. Ginbot Z. (2014). Protein Phosphatases PP 2A, PP 4 and PP 6: Mediators and Regulators in Development and Responses to Environmental Cues. Plant Cel Environ 37, 2631–2648. 10.1111/pce.12364 24810976

[B35] Lingner T. Kataya A. R. Antonicelli G. E. Benichou A. Nilssen K. Chen X. Y. (2011). Identification of Novel Plant Peroxisomal Targeting Signals by a Combination of Machine Learning Methods and *In Vivo* Subcellular Targeting Analyses. Plant Cell. 10.1105/tpc.111.084095 PMC3101550 21487095

[B71] Lu D. Wu S. Gao X. Zhang Y. Shan L. He P. A. (2010). A Receptor-Like Cytoplasmic Kinase, BIK1, Associates with a Flagellin Receptor Complex to Initiate Plant Innate Immunity. Proc. Natl. Acad. Sci. U.S.A. 107, 496–501. 10.1073/pnas.0909705107 20018686 PMC2806711

[B36] Luo M. Zhuang X. (2018). Review: Selective Degradation of Peroxisome by Autophagy in Plants: Mechanisms, Functions, and Perspectives. Plant Sci. 274, 485–491. 10.1016/j.plantsci.2018.06.026 30080638

[B37] Ma C. Reumann S. (2008). Improved Prediction of Peroxisomal PTS1 Proteins from Genome Sequences Based on Experimental Subcellular Targeting Analyses as Exemplified for Protein Kinases from Arabidopsis. J. Exp. Bot. 59, 3767–3779. 10.1093/jxb/ern221 18836189

[B38] Matre P. Meyer C. Lillo C. (2009). Diversity in Subcellular Targeting of the PP2A B′η Subfamily Members. Planta 230, 935–945. 10.1007/s00425-009-0998-z 19672620

[B39] Mergner J. Frejno M. List M. Papacek M. Chen X. Chaudhary A. (2020). Mass-spectrometry-based Draft of the Arabidopsis Proteome. Nature 579, 409–414. 10.1038/s41586-020-2094-2 32188942

[B40] Narsai R. Law S. R. Carrie C. Xu L. Whelan J. (2011). In-Depth Temporal Transcriptome Profiling Reveals a Crucial Developmental Switch with Roles for RNA Processing and Organelle Metabolism that Are Essential for Germination in Arabidopsis. Plant Physiol. 157, 1342–1362. 10.1104/pp.111.183129 21908688 PMC3252162

[B41] Nemoto K. Seto T. Takahashi H. Nozawa A. Seki M. Shinozaki K. (2011). Autophosphorylation Profiling of Arabidopsis Protein Kinases Using the Cell-free System. Phytochemistry 72, 1136–1144. 10.1016/j.phytochem.2011.02.029 21477822

[B42] Neuhaus A. Eggeling C. Erdmann R. Schliebs W. (2016). Why Do Peroxisomes Associate with the Cytoskeleton? Biochim. Biophys. Acta (Bba) - Mol. Cel Res. 1863, 1019–1026. 10.1016/j.bbamcr.2015.11.022 26616035

[B43] Oeljeklaus S. Schummer A. Mastalski T. Platta H. W. Warscheid B. (2016). Regulation of Peroxisome Dynamics by Phosphorylation. Biochim. Biophys. Acta (Bba) - Mol. Cel Res. 1863, 1027–1037. 10.1016/j.bbamcr.2015.12.022 26775584

[B44] Oikawa K. Matsunaga S. Mano S. Kondo M. Yamada K. Hayashi M. (2015). Physical Interaction between Peroxisomes and Chloroplasts Elucidated by *In Situ* Laser Analysis. Nat. Plants 1, 15035. 10.1038/nplants.2015.35 27247035

[B45] Pan R. Hu J. (2018). Proteome of Plant Peroxisomes. Subcell Biochem. 89, 3–45. 10.1007/978-981-13-2233-4_1 30378017

[B46] Paudyal R. Roychoudhry S. Lloyd J. P. (2017). Functions and Remodelling of Plant Peroxisomes. in eLS (Chichester: John Wiley & Sons). 10.1002/9780470015902.a9780470001677

[B47] Ramirez R. A. Espinoza B. Kwok E. Y. (2014). Identification of Two Novel Type 1 Peroxisomal Targeting Signals in *Arabidopsis thaliana* . Acta Histochem. 116, 1307–1312. 10.1016/j.acthis.2014.08.001 25183666 PMC4262709

[B48] Rao S. Zhou Z. Miao P. Bi G. Hu M. Wu Y. (2018). Roles of Receptor-like Cytoplasmic Kinase VII Members in Pattern-Triggered Immune Signaling. Plant Physiol. 177, 1679–1690. 10.1104/pp.18.00486 29907700 PMC6084675

[B49] Reumann S. Buchwald D. Lingner T. (2012). PredPlantPTS1: A Web Server for the Prediction of Plant Peroxisomal Proteins. Front. Plant Sci. 3, 194. 10.3389/fpls.2012.00194 22969783 PMC3427985

[B50] Reumann S. Chowdhary G. (2018). Prediction of Peroxisomal Matrix Proteins in Plants. Subcell Biochem. 89, 125–138. 10.1007/978-981-13-2233-4_5 30378021

[B51] Reumann S. (2004). Specification of the Peroxisome Targeting Signals Type 1 and Type 2 of Plant Peroxisomes by Bioinformatics Analyses. Plant Physiol. 135, 783–800. 10.1104/pp.103.035584 15208424 PMC514115

[B52] Sacksteder K. A. Jones J. M. South S. T. Li X. Liu Y. Gould S. J. (2000). PEX19 Binds Multiple Peroxisomal Membrane Proteins, Is Predominantly Cytoplasmic, and Is Required for Peroxisome Membrane Synthesis. J. Cel Biol 148, 931–944. 10.1083/jcb.148.5.931 PMC2174547 10704444

[B53] Saleem R. A. Knoblach B. Mast F. D. Smith J. J. Boyle J. Dobson C. M. (2008). Genome-wide Analysis of Signaling Networks Regulating Fatty Acid-Induced Gene Expression and Organelle Biogenesis. J. Cel Biol. 181, 281–292. 10.1083/jcb.200710009 PMC2315675 18426976

[B54] Sandalio L. M. Gotor C. Romero L. C. Romero-Puertas M. C. (2019). Multilevel Regulation of Peroxisomal Proteome by Post-Translational Modifications. Int. J. Mol. Sci. 20. 10.3390/ijms20194881 PMC6801620 31581473

[B70] Schneider C. A. Rasband W. S. Eliceiri K. W. (2012). NIH Image to ImageJ: 25 Years of Image Analysis. Nat. Methods. 9, 671–675. 22930834 10.1038/nmeth.2089 PMC5554542

[B55] Skoulding N. S. Chowdhary G. Deus M. J. Baker A. Reumann S. Warriner S. L. (2015). Experimental Validation of Plant Peroxisomal Targeting Prediction Algorithms by Systematic Comparison of *In Vivo* Import Efficiency and *In Vitro* PTS1 Binding Affinity. J. Mol. Biol. 427, 1085–1101. 10.1016/j.jmb.2014.12.003 25498386

[B56] Sørhagen K. Laxa M. Peterhänsel C. Reumann S. (2013). The Emerging Role of Photorespiration and Non-photorespiratory Peroxisomal Metabolism in Pathogen Defence. Plant Biol. (Stuttg) 15, 723–736. 10.1111/j.1438-8677.2012.00723.x 23506300

[B57] Stehlik T. Kremp M. Kahnt J. Bölker M. Freitag J. (2020). Peroxisomal Targeting of a Protein Phosphatase Type 2C via Mitochondrial Transit. Nat. Commun. 11, 2355. 10.1038/s41467-020-16146-3 32398688 PMC7217942

[B58] Thazar-Poulot N. Miquel M. Fobis-Loisy I. Gaude T. (2015). Peroxisome Extensions Deliver the Arabidopsis SDP1 Lipase to Oil Bodies. Proc. Natl. Acad. Sci. USA 112, 4158–4163. 10.1073/pnas.1403322112 25775518 PMC4386359

[B59] Titorenko V. I. Rachubinski R. A. (1998). Mutants of the Yeast Yarrowia Lipolytica Defective in Protein Exit from the Endoplasmic Reticulum Are Also Defective in Peroxisome Biogenesis. Mol. Cel Biol 18, 2789–2803. 10.1128/mcb.18.5.2789 PMC110658 9566898

[B60] Uhrig R. G. Labandera A.-M. Moorhead G. B. (2013). Arabidopsis PPP Family of Serine/threonine Protein Phosphatases: many Targets but Few Engines. Trends Plant Sci. 18, 505–513. 10.1016/j.tplants.2013.05.004 23790269

[B61] Uhrig R. G. Labandera A.-M. Tang L.-Y. Sieben N. A. Goudreault M. Yeung E. (2017). Activation of Mitochondrial Protein Phosphatase SLP2 by MIA40 Regulates Seed Germination. Plant Physiol. 173, 956–969. 10.1104/pp.16.01641 27923987 PMC5291043

[B62] Wang J. Wang Y. Gao C. Jiang L. Guo D. (2017). PPero, a Computational Model for Plant PTS1 Type Peroxisomal Protein Prediction. PLoS One 12, e0168912. 10.1371/journal.pone.0168912 28045983 PMC5207514

[B63] Wheeler T. J. Clements J. Finn R. D. (2014). Skylign: a Tool for Creating Informative, Interactive Logos Representing Sequence Alignments and Profile Hidden Markov Models. BMC Bioinformatics 15, 7. 10.1186/1471-2105-15-7 24410852 PMC3893531

[B64] Williams C. Bener Aksam E. Gunkel K. Veenhuis M. Van Der Klei I. J. (2012). The Relevance of the Non-canonical PTS1 of Peroxisomal Catalase. Biochim. Biophys. Acta (Bba) - Mol. Cel Res. 1823, 1133–1141. 10.1016/j.bbamcr.2012.04.006 22546606

[B65] Wright Z. J. Bartel B. (2020). Peroxisomes Form Intralumenal Vesicles with Roles in Fatty Acid Catabolism and Protein Compartmentalization in Arabidopsis. Nat. Commun. 11, 6221. 10.1038/s41467-020-20099-y 33277488 PMC7718247

[B66] Zhang X. Hu J. (2010). The Arabidopsis Chloroplast Division Protein DYNAMIN-RELATED PROTEIN5B Also Mediates Peroxisome Division. Plant Cell 22, 431–442. 10.1105/tpc.109.071324 20179140 PMC2845408

[B67] Zimmermann P. Laule O. Schmitz J. Hruz T. Bleuler S. Gruissem W. (2008). Genevestigator Transcriptome Meta-Analysis and Biomarker Search Using rice and Barley Gene Expression Databases. Mol. Plant 1, 851–857. 10.1093/mp/ssn048 19825587

[B68] Zulawski M. Schulze G. Braginets R. Hartmann S. Schulze W. X. (2014). The Arabidopsis Kinome: Phylogeny and Evolutionary Insights into Functional Diversification. BMC Genomics 15, 548. 10.1186/1471-2164-15-548 24984858 PMC4112214

