# Supplementary material for: Identification of Arabidopsis Protein Kinases That Harbor Functional Type 1 Peroxisomal Targeting Signals
Source: Front Cell Dev Biol. 2022 Feb 15;10:745883. doi: 10.3389/fcell.2022.745883 (PMC8886021; doi:10.3389/fcell.2022.745883)
Supplement: Supplementary file 1 [file DataSheet2.PDF]

**Supplemental Figure 7 Multiple sequence alignments and phylograms for previously studied protein kinases harboring PTS1-like sequence conservation.**

The full-length kinase sequences extracted from TAIR were subjected to protein BLAST and the orthologs harboring PTS1 were extracted and used for alignments. The C-terminal PTS1 tripeptides are highlighted in bold red in each alignment and on phylograms (see attached table of contents and attached linked pages). AlignX (Vector NTI Advance, Invitrogen) was used to conduct and manage multiple sequence alignment projects based on the Clustal W algorithm (Nucleic Acid Research, 22 (22): 4673-4680, 1994). Color background: yellow, identical aa; blue, conservative aa; white, weakly similar aa; green, block of similar aa. The phylograms were generated by the AlignX program (Vector NTI Advance, Invitrogen) using the Neighbor Joining method (NJ) (Saitou and Nei, 1987). These distances are related to the degree of divergence between the sequences. PTD: Peroxisomal targeting domains

## Contents


K13 _AT4G31230.1 ..... 2

K14_ AT1G69270.1 ..... 5

K15_AT3G08720.1 ..... 7

K16_ AT3G20530.1 ..... 8

K17_ AT3G17420.1 ..... 9

K18_ AT5G04870.1 ..... 12

K19_ AT5G03730.1 ..... 17

# K13 _AT4G31230.1

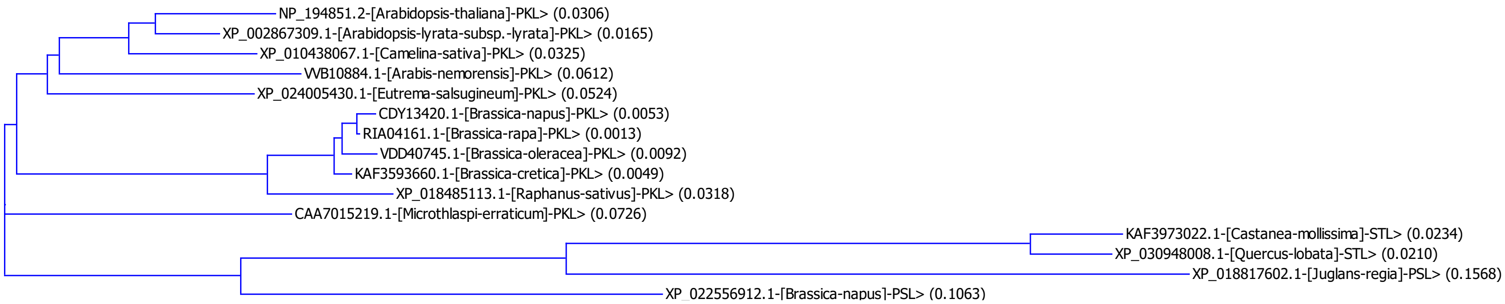


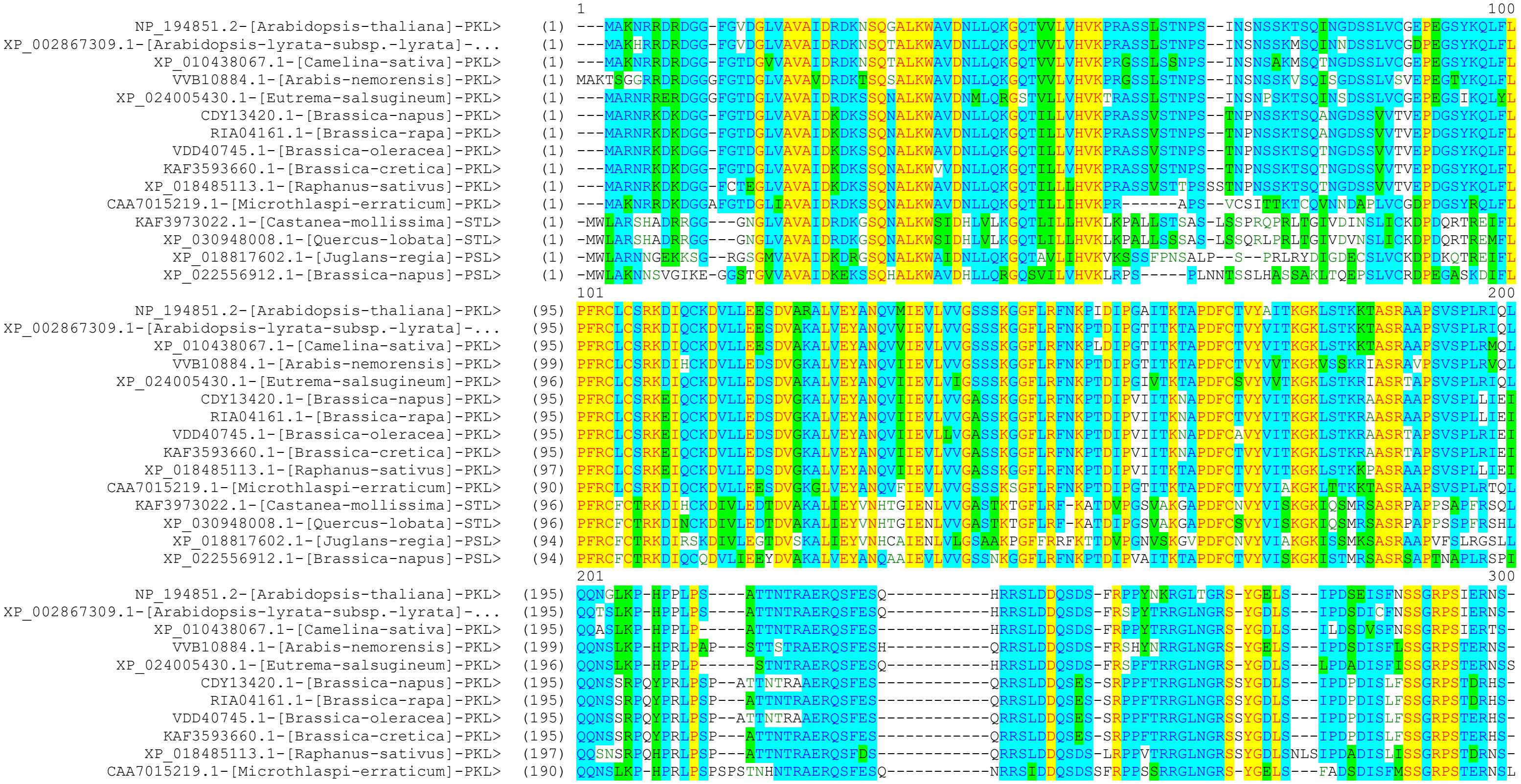

```
KAF3973022.1-[Castanea-mollissima]-STL>                (195) INQSSIKSDSWDPNLYQSTTPKKVTEKPPVDLF-------------HRMQDETDI-MRSPFSRKSYNGKS-YGDLT---LPDTDISFISSARPSIDHMF-
XP_030948008.1-[Quercus-lobata]-STL>                   (195) INQSSIKSDAWDPNLHQSTTPKKGTEKPPVDLF-------------HRMQDDTDI-MRSPFSRKGYDGKS-YGELT---LPDSDISFVSSARPSIDQMF-
XP_018817602.1-[Juglans-regia]-PSL>                    (194) NQGSIKS-DTAEPQLIDFAATVKGAERLQSELP------------RRSLHDETDL-IRSPLPRKGLNWKS-YGELP---LLDADISFVSSGRPSIDRVF-
XP_022556912.1-[Brassica-napus]-PSL>                   (194) QPPSLRP-PQPVPS----TSNNMRADRQSFESQRRSMEDRRSVEDRRSVEDQSDS-FRSPFTRRG-YGRS-YGDLS---VPESDISFVSSGRPSIDRMF-
                                                             301                                                                                            400
NP_194851.2-[Arabidopsis-thaliana]-PKL>                (273) PSLYDNSDPNRTPP--RLSNFSDVDYCSFE--SMTFGRRSMDLSSPT---AFSTGSFENERFSSASQGGD-DVEAEMRRLKLELKQTMEMYSTACKEALT
XP_002867309.1-[Arabidopsis-lyrata-subsp.-lyrata]-...  (273) PSLYDNSDPNRTPP--RLSNFSDIDYGSFE--SMTFGRRSMDISSPT---AFSTGSFENERFSSASQGGD-DVEAEMRRLKLELKQTMEMYSTACKEALT
XP_010438067.1-[Camelina-sativa]-PKL>                  (271) LSLYDNQDPNRTPP--RLSNFSDVDYGSFE--SMTIGRRSMDISSPN---AFSTGSFENERFSSASQGVD-DVEAEMRRLKLELKQTMEMYSTACKEALT
VVB10884.1-[Arabis-nemorensis]-PKL>                    (278) LSLYDNSDPNRTPP--RLSNFSDTDFGSFE--SMTYGRRSMDISSPT---AFSTGSFENERFSSASQGGD-DVEAEMRRLRLELKQTMDMYSTACKEALT
XP_024005430.1-[Eutrema-salsugineum]-PKL>              (273) TSLYDNYDPNRTPPP-RLSNFSDIDSGSFE--SMTNGRRSMDISSPT---AFSTGSFETDRFSSASQVADIDVEAEMRRLKLELKQTMEMYSTACKEALT
CDY13420.1-[Brassica-napus]-PKL>                       (276) LSLFDNSDQNRTPPP-RLSNFSDIDNGSFE--SMNYGQGSMDISSPP---ASSRDSFENERFSSAEPGGE-DVEAEMRRLKLELKQTMEMYSSACKEALT
RIA04161.1-[Brassica-rapa]-PKL>                        (275) LSLFDNSDQNRTPPP-RLSNFSDIDNGSFE--SMNYGQGSMDISSPP---ASSRDSFENERFSSAEPGGE-DVEAEMRRLKLELKQTMEMYSSACKEALT
VDD40745.1-[Brassica-oleracea]-PKL>                    (276) LSLFDNSDQNRTPPP-RLSNFSDIDNGSFE--SMNYGQGSMDISSPP---ASSRDSFENERFSSASPGGE-DVEAEMRRLKLELKQTMEMYSSACKEALT
KAF3593660.1-[Brassica-cretica]-PKL>                   (275) LSLFDNSDQNRTPPP-RLSNFSDIDNGSFE--SMTYGQGSMDISSPP---ASSRDSFENERFSSASPGGE-DVEAEMRRLKLELKQTMEMYSSACKEALT
XP_018485113.1-[Raphanus-sativus]-PKL>                 (280) LSLFDNADQNRTPPP-RLSNFSDIDRGSFE--SITYGQGTLDISSPP---ASSRDSFENERFSSASPRGE-DVEAEMRRLKLELKQTMEMYSSACKEALT
CAA7015219.1-[Microthlaspi-erraticum]-PKL>             (274) SSLYDNYDSNRTPPPPRLSNFSDVDYGSFE--SMSYGRKSMDINSPT---AFSIGSFDNERSSSASQGGDGDVEAEMRRLKLELKQTMEMYSTACKEALT
KAF3973022.1-[Castanea-mollissima]-STL>                (276) -SVYDNLDIGSTPS--RMSFTSTEVDMKENFDIMQIGPKSLEIGSPPP--EFSALSPESDRLSNSSQTAE-EVEAEMRRLKLELKQTMEMYSTACKEALT
XP_030948008.1-[Quercus-lobata]-STL>                   (276) -TVYDNLDIGSTPS--RMSLTSTEVDMKENFDIMQIGRKSLEIGSPPP--EFSNLSQENDRLSNSAQTAE-DVETEMRRLKLELKQTMEMYSTACKEALT
XP_018817602.1-[Juglans-regia]-PSL>                    (275) --HYDHMNEIGHRTPSRLSHISERDMINPSFEPLYFGRKSVDIASPPPESLAFSSESDQDRLSGELQSTEGALEDEMRRLKLELKQTMEMYSTACKEALT
XP_022556912.1-[Brassica-napus]-PSL>                   (282) PNINDMNDPNRTPP--RLSNFSDMDYGPNFESSSNYGRRSVDVNSPT---DLSSGSFENERFSSASAMDD--VESEMRRLKLELKQTMEMYSTACKEALT
                                                             401                                                                                            500
NP_194851.2-[Arabidopsis-thaliana]-PKL>                (365) AKHKATELQRWKLAEERKFEEAKLAEEAALAIAEKEKAKSKAAMEAAEAAQRIADIESRKRVDAETKALKESEARTKAVNALA-KDVRYRKYSIEEIEDA
XP_002867309.1-[Arabidopsis-lyrata-subsp.-lyrata]-...  (365) AKHKATELQRWKLAEERKFEEAKLAEEAALAIAEKEKAKSKAAMEAAEAAQRIADIESRKRVDAEMKALKESEARTKAVNALANSDVRYRKYSIEEIEDA
XP_010438067.1-[Camelina-sativa]-PKL>                  (363) AKHKATELQRWKLEEERKFEEAKIAEEAALAIAEKEKAKSKAAMEAAEAAQRIAEIESRKRVNAEMKALKESEARTKAVHALAKSEVRYRKYSIEEIEDA
VVB10884.1-[Arabis-nemorensis]-PKL>                    (370) AKQKATELQRWKLEEERKFEEAKLAEEAALAIVEKEKAKSKAAMEAAEAAKRIAEIESRKRIDAEMKALKESEEKTKAVNALAHSDVRYRKYSIEEIEDA
XP_024005430.1-[Eutrema-salsugineum]-PKL>              (367) AKQKATELQRWKMEEERKLEEAKNAEEAALAIVEKEKAKSKAAIEA---AQRIAEIESRKRISAEMKALMESEEKTKAVNALAHADVRYRKYSIEEIEDA
CDY13420.1-[Brassica-napus]-PKL>                       (369) SKRKATELQRWKLEEERKYEEAKQAEEAALAIAEKEKAKSKAALEAAEAAQRIAEMESKKRISAEMKALKETEEKKKAVDALANADVRYRKYSIEEIEDA
RIA04161.1-[Brassica-rapa]-PKL>                        (368) SKRKATELQRWKLEEERKYEEAKQAEEAALAIAEKEKAKSKAALEAAEAAQRIAEMESKKRISAEMKALKETEEKKKAVDALANADVRYRKYSIEEIEDA
VDD40745.1-[Brassica-oleracea]-PKL>                    (369) SKRKATELQRWKLEEERKYEEAKQAEEAALAIAEKEKAKSKAALEAAEAAQRIAEMESKKRISAEMKALKETEEKRKAVDALANADVRYRKYSIEEIEDA
KAF3593660.1-[Brassica-cretica]-PKL>                   (368) SKRKATELQRWKLEEERKYEEAKQAEEAALAIAEKEKAKSKAALEAAEAAQRIAEMESKKRISAEMKALKETEEKRKAVDALANADVRYRKYSIEEIEDA
XP_018485113.1-[Raphanus-sativus]-PKL>                 (373) SKRKASELQRWKLEEERKYEEAKLAEEAALAIAEKEKAKSKAALEAAEAAQRIAEMESKKRISAEMKVLKETEEKKKAVDALAHADVRYRKYTIEEIEDA
CAA7015219.1-[Microthlaspi-erraticum]-PKL>             (369) AKQKATELQRWKLEEERKFEEAKHAEEAALATVEKERAKSKAAIEAAEAAQRIAEIESKKRINAEMKALKESEEKKKAVDALAHSDIRYRKYTIEEIEDA
KAF3973022.1-[Castanea-mollissima]-STL>                (370) AKQKAVELQRWKLEEERRLQEAQLAEEAALALAEKEKAKSRAAIEAAEAAKRIAELEGQKRINAELKVLQESEEKRKVLDALAQSDIRYRRYSIEEIEAA
XP_030948008.1-[Quercus-lobata]-STL>                   (370) AKQKAVELQRWKLEEERRLQEARLAEEAALALAEKEKAKSRAAIEAAEAAKRIAELEGQKRINAEMKVLQESEEKRKVLDALAQSDIRYRRYSIEEIEAA
XP_018817602.1-[Juglans-regia]-PSL>                    (373) AKQMAVELQRWKLEEVRRLEDAQLAEEAALATVEKEKAKSRAAIEAAEAAKRIAELEAQKRINAEMKALRETEEKRKAFDALAHSDGRCRRYTIDEIEVA
XP_022556912.1-[Brassica-napus]-PSL>                   (375) AKQKATELQRWKLEEERKLEEARLAEEAALAIAEKEKARSKAAMEAAEAAQRIAELESKKRVNAEMKALKESEEKTKALTALANNDVRYRKYSIEEIESA
                                                             501                                                                                            600
NP_194851.2-[Arabidopsis-thaliana]-PKL>                (464) TEFFDDKYKIGEGSYGPVYKCYLDHTPVAVKALRPDAAQGRSQFQKEVEVLCSIRHPNMVLLLGACPECGCLVYEFMANGSLEDRLFRQGDSPALSWQTR
XP_002867309.1-[Arabidopsis-lyrata-subsp.-lyrata]-...  (465) TEFFDEKYKIGEGGYGPVYKCYLDHTPVAVKALRPDAAQGRSQFQQEVEVLSSIRHPNMVLLLGACPECGCLVYEFMANGSLEDRLFREGDSPPLSWQTR
XP_010438067.1-[Camelina-sativa]-PKL>                  (463) TEFFDEKYKIGEGGYGPVYKCYLDHTPVAVKALRPDAAQGRSQFQQEVEVLSSMRHPNMVLLLGVCPECGCLVYEFMANGSLEDRLFRQGDSPALSWQTR
VVB10884.1-[Arabis-nemorensis]-PKL>                    (470) TEFFDDKYKIGEGGYGPFYKCYLDNTPVAVKTLHPDASQGMSQFQQEVEVLSCMRHPNMVLLLGACPECGCLVYEFMSNGSLEDRLFRLGDSPPLSWQTR
XP_024005430.1-[Eutrema-salsugineum]-PKL>              (464) TDYFDEKYKIGEGGYGPVYKCYLDHTPVAVKALRPDATQGRSQFQKEVEVLSCMRHPNMVLLLGACPECGCLVYEFMANGSLEDRLFRQGNTPPLSWQTR
CDY13420.1-[Brassica-napus]-PKL>                       (469) TEYFDEKYKIGEGGYGPVYKCYLDQTPVAVKALHTDAAQGRSQFKQEVEVLSCMRHPHMVLLLGACPEGGCLVYEFMSNGSLEDRLFQQGDSPPLSWQTR
RIA04161.1-[Brassica-rapa]-PKL>                        (468) TEYFDEKYKIGEGGYGPVYKCYLDQTPVAVKALHTDAAQGRSQFKQEVEVLSCMRHPHMVLLLGACPEGGCLVYEFMSNGSLEDRLFQQGDSPPLSWQTR
VDD40745.1-[Brassica-oleracea]-PKL>                    (469) TEYFDEKYKIGGGGYGPVYKCYLDQTPVAVKALHTDAAQGRSQFKQEVEVLSCMRHPHMVLLLGACPEGGCLVYEFMSNGSLEDRLFQQGDSPPLSWQTR
KAF3593660.1-[Brassica-cretica]-PKL>                   (468) TEYFDEKYKIGGGGYGPVYKCYLDQTPVAVKALHTDAAQGRSQFKQEVEVLSCMRHPHMVLLLGACPEGGCLVYEFMSNGSLEDRLFQQGDSPPLSWQTR
XP_018485113.1-[Raphanus-sativus]-PKL>                 (473) TEYFDEKYKIGEGGYGPVYKCYLDQTPVAVKALHPDAAQGMSQFKQEVEVLSCMRHPHMVLLLGACSEGGCLVYEFMSNGSLEDRLFQQGDSPPLSWQTR
CAA7015219.1-[Microthlaspi-erraticum]-PKL>             (469) TEYFDDKYKIGEGSYGPVYKCYLDHTPVAVKVLRPDATQGRSQFQQEVEVLSCMRHPNMVLLLGACPECGCLVYEYMANGSLEDRLFRQGDSPPLSWQTR
KAF3973022.1-[Castanea-mollissima]-STL>                (470) TDFFTESRKIGEGGYGPVYKCYLDHTPVAIKVLRPDAAHGRSQFLQEVEVLSCIRHPNMVLLLGACPEYGCLVYEFMANGSLDDRLFCRGGSPPLSWQLR
XP_030948008.1-[Quercus-lobata]-STL>                   (470) TDFFTESRKIGEGGYGPVYKCYLDHTPVAIKVLRPDAAHGRSQFLQEVEVLSCIRHPNMVLLLGACPEYGCLVYEFMANGSLDDRLFCRGGSPPLSWQLR
XP_018817602.1-[Juglans-regia]-PSL>                    (473) TEFFRVSRKIGEGGYGPVYKCYLDHTPVAIKVLRPDAAQGRSQFQQEVEVLSCIRHPNMVLLLGACPEYGCLVYEYMANGSLEDRLFCRGNTRPLSWQKR
XP_022556912.1-[Brassica-napus]-PSL>                   (475) TEFFAEKYKIGEGGYGPVYKCYLDHTPVAVKVLRADAAQGRSQFQQEVEVLSSIRHPNMVLLLGACPECGCLVYEFMANGSLEDRLFRLGNSPPLSWQMR
                                                             601                                                                                            700
NP_194851.2-[Arabidopsis-thaliana]-PKL>                (564) FRIAAEIGTVLLFLHQTKPEPLVHRDLKPANILLDRNFVSKLADVGLARLVPPSVANTVTQYHMTSTAGTFCYIDPEYQQTGMLGVKSDIYSLGIMFLQL
XP_002867309.1-[Arabidopsis-lyrata-subsp.-lyrata]-...  (565) FRIAAEIGTVLLFLHQTKPEPLVHRDLKPANILLDRNFVSKVADVGLARLVPPSVANTVTQYRMTSTAGTFCYIDPEYQQTGMLGVKSDIYSLGIMFLQL
XP_010438067.1-[Camelina-sativa]-PKL>                  (563) FRIAAEIGTVLLFLHQTKPEPLVHRDLKPANILLDRNFVSKVADVGLARLVPASVANTVTQYRMTSTAGTFCYIDPEYQQTGMLGVKSDIYSLGIMFLQL
VVB10884.1-[Arabis-nemorensis]-PKL>                    (570) FRIAAEIGTVLLYLHQTKPEPLVHRDLKPANILLDRNFVSKVADVGLARLVPPSVANTVTQYRMTATAGTFCYIDPEYQQTGMLGVKSDIYSLGIIFLQL
XP_024005430.1-[Eutrema-salsugineum]-PKL>              (564) FRIAAEIGTVLLFLHQTKPEPLVHRDLKPANILLDRNFVSKLADVGLARLVPPSVANTVTQYRMTSTAGTFCYIDPEYQQTGMLGVKSDIYSLGIMFLQL
CDY13420.1-[Brassica-napus]-PKL>                       (569) FRIAAEIGTVLLYLHQTKPEPLVHRDLKPANILLDRNFVSKVADVGLARLVPPSVANTVTQYRMTSTAGTFCYIDPEYQQTGMLGVKSDIYSLGIMYLQM
RIA04161.1-[Brassica-rapa]-PKL>                        (568) FRIAAEIGTVLLYLHQTKPEPLVHRDLKPANILLDRNFVSKVADVGLARLVPPSVANTVTQYRMTSTAGTFCYIDPEYQQTGMLGVKSDIYSLGIMYLQM
VDD40745.1-[Brassica-oleracea]-PKL>                    (569) FRIAAEIGTVLLYLHQTKPEPLVHRDLKPANILLDRNFVSKVADVGLARLVPPSVANTVTQYRMTSTAGTFCYIDPEYQQTGMLGVKSDIYSLGIMYLQM
KAF3593660.1-[Brassica-cretica]-PKL>                   (568) FRIAAEIGTVLLYLHQTKPEPLVHRDLKPANILLDRNFVSKVADVGLARLVPPSVANTVTQYRMTSTAGTFCYIDPEYQQTGMLGVKSDIYSLGIMYLQM
XP_018485113.1-[Raphanus-sativus]-PKL>                 (573) FRIAAEIGTVLLYLHQTKPEPLVHRDLTPANILLDRNFVSKVADVGLARLVPPSVANTVTQYRMTSTAGTFCYIDPEYQQTGMLGVKSDIYSLGIMFLQM
CAA7015219.1-[Microthlaspi-erraticum]-PKL>             (569) FRIAAEIGTVLLFLHQTKPEPLVHRDLKPANILLDRNFVSKVADVGLARLVPPSVANTVTQYRMTSTAGTFCYIDPEYQQTGMLGVKSDIYSLGIMFLQL
KAF3973022.1-[Castanea-mollissima]-STL>                (570) FRIAAEIGTGLLFLHQSKPEPLVHRDLKPGNILLDRNFVSKISDVGLARLVPPSLADTVTQYRMTSTAGTFCYIDPEYQQTGMLGVKSDIYSIGIMFLQL
XP_030948008.1-[Quercus-lobata]-STL>                   (570) FRIAAEIGTGLLFLHQSKPEPLVHRDLKPGNILLDRNFVSKISDVGLARLVPPSLADTVTQYRMTSTAGTFCYIDPEYQQTGMLGVKSDIYSIGIMFLQL
XP_018817602.1-[Juglans-regia]-PSL>                    (573) FKIAAEVATGLLFLHQAKPEPLVHRDLKPANILLDSNYVSKISDVGLARLVPPSVADNMTQYRMTSTAGTFCYIDPEYQQTGMLGVKSDIYSIGIMFLQM
XP_022556912.1-[Brassica-napus]-PSL>                   (575) FRIAAEIGTGLLFLHQAKPEPLVHRDLKPGNILLDRNFVSKISDVGLARLVPPTVADTVTQYRMTSTAGTFCYIDPEYQQTGMLGVKSDIYSLGIMFLQL
```

```
                                                                701                                                                                               800
             NP_194851.2-[Arabidopsis-thaliana]-PKL>   (664) ITGKPPMGLTHYVERALEKGNLKDLLDPAVSDWPVEDTTEFAKLALKCAEIRRKDRPDLSKVILPELNRLRVLAEESTQAAVVINSSGPT--PTESQTSS
XP_002867309.1-[Arabidopsis-lyrata-subsp.-lyrata]-...  (665) ITGKPPMGLTHYVERALEKGNLKDLLDPAVSDWPVEDTTEFAKLALKCAEIRRKDRPDLSKVILPELNRLRVLAEESTKTAVVINSPGPT--PTESQSSS
           XP_010438067.1-[Camelina-sativa]-PKL>       (663) ITAKPPMGLTHYVGRALEKGNLKDLLDPAVSDWPLEDTTEFAKLALKCAEIRRKDRPDLSKVILPELNRLRVLAEESTQSAVVINSPGPT--PTVSQTSS
             VVB10884.1-[Arabis-nemorensis]-PKL>       (670) ITGKPPMGLTHYIEKALEKGNLKDLLDPAVSDWPVKDTTEFAKLALKCAEIRRKDRPDLSKVILPELNRLRELAEESTQSAVVINSPGPT--PTDSQNSS
       XP_024005430.1-[Eutrema-salsugineum]-PKL>       (664) ITGKPPMGLTHYVERALEKGNLKDLLDPAVSDWPVEDTTEFAKLALKCAEIRRKDRPDLSKVILPELNRLRTLAEESTQSAVVINSPGPTSSPTDSQTSS
               CDY13420.1-[Brassica-napus]-PKL>        (669) ITGKPPMGLTRYIESALEKGNLKDVLDPSVSDWPVEDTTEFAKLALKCAEIRRKDRPDLSKIILPELNRLRALAEESSRSAVVINSPGPS--PTVSQTSS
                RIA04161.1-[Brassica-rapa]-PKL>        (668) ITGKPPMGLTRYIESALEKGNLKDVLDPSVSDWPVEDTTEFAKLALKCAEIRRKDRPDLSKIILPELNRLRALAEESSRSAVVINSPGPS--PTVSQTSS
             VDD40745.1-[Brassica-oleracea]-PKL>       (669) ITGKPPMGLTRYIESALEKGNLKDVLDPSVSDWPVEDTTEFAKLALKCAEIRRKDRPDLSKVILPELNRLRALAEESSRSAVVINSPGPS--PTVSQTSS
            KAF3593660.1-[Brassica-cretica]-PKL>       (668) ITGKPPMGLTRYIESALEKGNLKDVLDPSVSDWPVEDTTEFAKLALKCAEIRRKDRPDLSKVILPELNRLRTLAEESSRSAVVINSPGPS--PTVSQTSS
         XP_018485113.1-[Raphanus-sativus]-PKL>        (673) ITGKPPMGLTRYIESALEKGNLKDVLDPSVSDWPVEDTTEFAKLALKCAEIRRKDRPDLSKVILPELNRLRALGEESSQSAVVINSPGPS--PTVSQTSS
    CAA7015219.1-[Microthlaspi-erraticum]-PKL>         (669) ITGKAPMGLTHYVEKALEKGNFKGLLDPSVSDWPVEDTTEFAKLALKCAEIRRKDRPDLSKVILPELNRLRALAES-TQSAVVINSSGPT--PCESQTSS
       KAF3973022.1-[Castanea-mollissima]-STL>         (670) ITAKPPMGLTHHVERAIEKGTFTQLLDPAVTDWPVEETLCFAKLALKCSELRRKDRPDLGKDILPELNRLRAIAEETMHPTLMFSSPSLS--PAHSQVSL
          XP_030948008.1-[Quercus-lobata]-STL>         (670) ITAKPPMGLTHHVERAIEKGTLTQLLDPAVTDWPVEETLCFAKLALKCSELRRKDRPDLGKDILPELNRLRAIAEETMHPTLMFSSPSLS--PAHSQVSL
           XP_018817602.1-[Juglans-regia]-PSL>         (673) ITAKPPMGLTHLVERAIETGTFSEMLDPLVLDWPAEEAMCFAKLALKCAELRRKDRPDLCKDILPELNRLRVLAEETMYPTPMNGSPGSS--PDRSQVSL
          XP_022556912.1-[Brassica-napus]-PSL>         (675) ITAKPPMGLTHYVERALEKGTLSDLLDPAVTDWPVEDTAEFAKLALQCAELRRKDRPDLAKVILPELNRLRTLADESQLX--SLNSPGPS----------
                                                                801                 821
             NP_194851.2-[Arabidopsis-thaliana]-PKL>   (762) PKL------------------
XP_002867309.1-[Arabidopsis-lyrata-subsp.-lyrata]-...  (763) PKL------------------
           XP_010438067.1-[Camelina-sativa]-PKL>       (761) PKL------------------
             VVB10884.1-[Arabis-nemorensis]-PKL>       (768) PKL------------------
       XP_024005430.1-[Eutrema-salsugineum]-PKL>       (764) PKL------------------
               CDY13420.1-[Brassica-napus]-PKL>        (767) PKL------------------
                RIA04161.1-[Brassica-rapa]-PKL>        (766) PKL------------------
             VDD40745.1-[Brassica-oleracea]-PKL>       (767) PKL------------------
            KAF3593660.1-[Brassica-cretica]-PKL>       (766) PKL------------------
         XP_018485113.1-[Raphanus-sativus]-PKL>        (771) PKL------------------
    CAA7015219.1-[Microthlaspi-erraticum]-PKL>         (766) PKL------------------
       KAF3973022.1-[Castanea-mollissima]-STL>         (768) QLDEDLLDEHSDLSSKLSSTL
          XP_030948008.1-[Quercus-lobata]-STL>         (768) QLDEDLPHEHSDLSSKYSSTL
           XP_018817602.1-[Juglans-regia]-PSL>         (771) QLEGDLPHTQSGESSRTPSL-
          XP_022556912.1-[Brassica-napus]-PSL>         (763) PSL------------------
```

# K14_ AT1G69270.1

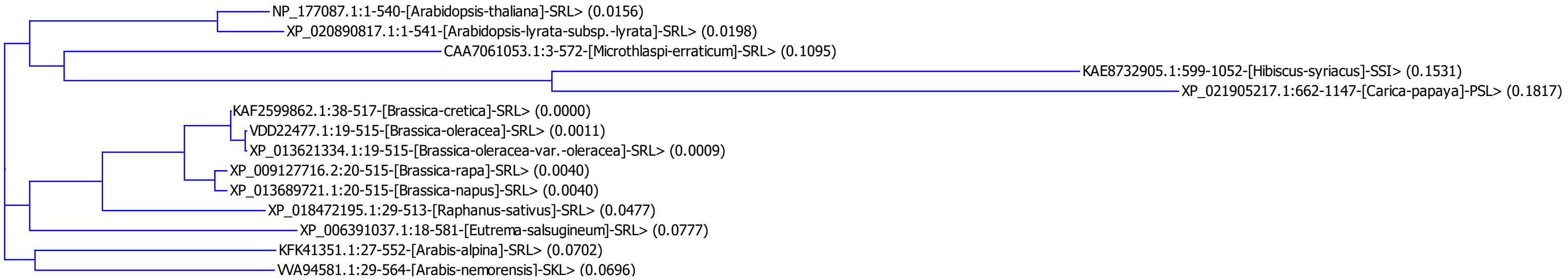


```
                                                             1                                                                                                  100
  NP_177087.1:1-540-[Arabidopsis-thaliana]-SRL>      (1) MKLLGLVFLLFNLFMFSFSRKLLTESGGGLHDEAALLKLKSSFLDPNGVLSSWVSDSSSNHCSWYGVSCNSDSRVVSLILRGCDELEGSGVLHLPDLSSC
XP_020890817.1:1-541-[Arabidopsis-lyrata-subsp.-ly...  (1) MKILGLVFLLFNLFVFSFSRKLLTESG--LHDEAVLLKLKSSFFDPNGVLSSWVSDSSSNHCSWYGVSCNSDLRVVSLILRGCDELEGSGVLHFPDLSSC
   CAA7061053.1:3-572-[Microthlaspi-erraticum]-SRL>  (1) -KVILALFLVLNLFAFSFSRKLFSESGGSHDDEAALLELKSSFSDPNGVLSSWNPDSS-NHCSWFGVSCNSDSRVVSLILRGCDEASR------LHFSAS
     KAE8732905.1:599-1052-[Hibiscus-syriacus]-SSI>  (1) -----------------------------------------------------------SDNQIEGVIPQSFRDLKSLVLLDLS----------------
       XP_021905217.1:662-1147-[Carica-papaya]-PSL>  (1) ---------------------------------------------------------SLSGNNLTGPIPTSFGQLQSLEVLELS----------------
        KAF2599862.1:38-517-[Brassica-cretica]-SRL>  (1) --------------------------------------LKTSFSDPHGVLSSWDPDIS-NHCSWFGVSCNSDLRVVSLIL---D----------------
          VDD22477.1:19-515-[Brassica-oleracea]-SRL>  (1) ----------------SSSRKLKP--F---HDATVLLELKTSFSDPHGVLSRWDPNIS-NHCSWFGVSCNSDLRVVSLIL---D----------------
XP_013621334.1:19-515-[Brassica-oleracea-var.-oler...  (1) ----------------SSSRKLKP--F---HDATVLLELKSSFSDPHGVLSRWDPNIS-NHCSWFGVSCNSDLRVVSLIL---D----------------
           XP_009127716.2:20-515-[Brassica-rapa]-SRL>  (1) ----------------SSSRKLKP--F---HDATVLLELKTSFSDPHGVLSSWDPNIS-NHCSWFGVSCNSDLRVVSLTL---D----------------
          XP_013689721.1:20-515-[Brassica-napus]-SRL>  (1) ----------------SSSRKLKP--F---HDATVLLELKTSFSDPHGVLSSWDPNIS-NHCSWFGVSCNSDLRVVSLTL---D----------------
        XP_018472195.1:29-513-[Raphanus-sativus]-SRL>  (1) ------------------------------HDATVLLELKTSFSDPHGVLSSWDPDISSNHCSWFGVSCNSDLRVVSLTL---DG---------------
     XP_006391037.1:18-581-[Eutrema-salsugineum]-SRL>  (1) ------------------SFSRKLFSENAFHDETVLLELKSSFSDPNGVLSSWDPNSS-NHCSWFGVSCNFDSRVVSLILRGCDEPQGS-----LHLPDS
             KFK41351.1:27-552-[Arabis-alpina]-SRL>  (1) -------------------------------DEDVLLKLKSSFIDPNGVLSSWDPNSS-NYCSWFGVSCNSDSRVVSLILRGCDDLKG--SLHFSDSSSC
           VVA94581.1:29-564-[Arabis-nemorensis]-SKL>  (1) -------------------------------DEAVLLKLKSSFSDPNGVLSSWNPDSS-NHCSWFGVSCNSDSRVVCLILRGCDDDGFEG----SSLHFP
                                                         101                                                                                                200
  NP_177087.1:1-540-[Arabidopsis-thaliana]-SRL>    (101) SSSKRRLGGVISPVVGDLSEIRVLSLSFNDLRGEIPKEIWGLEKLEILDLKGNNFIGGIRVVD-------------------------------------
XP_020890817.1:1-541-[Arabidopsis-lyrata-subsp.-ly... (99) SSSRRRLGGVISPVVGDLSEIRVLSLSFNDLRGEIPKEIWGLEKLEILDLKGNSFIGGIRVLDGFSR---------------------------------
   CAA7061053.1:3-572-[Microthlaspi-erraticum]-SRL> (93) SPCSSKLGGEISPAVGDLSEIRVLSLPFNDLVGQIPKKIRNLEKLEVLDLQGNSFTGEFNRIRVSNRGSDEKPN---------------LSIRRLLAKFR
     KAE8732905.1:599-1052-[Hibiscus-syriacus]-SSI> (26) ---GNKLTGPVPEGLSQLKYLKHLSLGSNNLSGAVPSSFGRFRSLKESEHSSDSLSVQTDDVT-------------------------------------
       XP_021905217.1:662-1147-[Carica-papaya]-PSL> (28) ---SNSLSGEIPDELVNLKNLTVLLLNNNKLSGQIPSGLANVTTLSTFNVSFNNLSGSLPNDDLMKCSSILGN---------------------------
        KAF2599862.1:38-517-[Brassica-cretica]-SRL> (43) ---ETRLGGEISPVVGSLSEIRVLSLAFNNLGGQVPKEIWGLKRLEILDLQGNDFRVQRFSRD-------------------------------------
          VDD22477.1:19-515-[Brassica-oleracea]-SRL> (60) ---ETRLGGEISPVVGSLSEIRVLSLAFNNLGGQVPKEIWGLKRLEILDLQGNDFRVQRFSRD-------------------------------------
XP_013621334.1:19-515-[Brassica-oleracea-var.-oler... (60) ---ETRLGGEISPVVGSLSEIRVLSLAFNNLGGQVPKEIWGLKRLEILDLQGNDFRVQRFSRD-------------------------------------
           XP_009127716.2:20-515-[Brassica-rapa]-SRL> (60) ---ETRLGGEISPVVGSLSEIRVLSLAFNNLGGQVPKEIWGLKRLEFLDLQGNDFRVQSFSRN-------------------------------------
          XP_013689721.1:20-515-[Brassica-napus]-SRL> (60) ---ETRLGGEISPVVGSLSEIRVLSLAFNNLGGQVPKEIWGLKRLEFLDLQGNDFRVQRFSRN-------------------------------------
        XP_018472195.1:29-513-[Raphanus-sativus]-SRL> (53) ----TRLGGEISPAVGSLSEIRVLSLAFNNLGGQVPNEIWGLKKLKSLDLQGNDDLRVPRFSPN------------------------------------
     XP_006391037.1:18-581-[Eutrema-salsugineum]-SRL> (77) SSCSSRLGGEISPVVGNLSEIRVLSLAFNDLGGQIPEEIWGLEKLEVLDLQGNTFTGESHGNRVLNQGLNRISKDLNPVDEKPKLWIQRFLANFGKSLKV
             KFK41351.1:27-552-[Arabis-alpina]-SRL> (67) S--RRRLGGQISPVVGFLSEIRVLSLAFNDLEGEIPKEIWGLKKLEVLDLQGNNFIEEISGIRVLIKRFLVDP--------------------------L
           VVA94581.1:29-564-[Arabis-nemorensis]-SKL> (65) SCSRSRLGGQISPVVGDLSEIRVLSLPFNDLVGQIPKEIWGLKRLQVLDLQGNNFIGQSSGIRVWIQRFLANFGKSWKKV-----------------IDP
                                                         201                                                                                                300
  NP_177087.1:1-540-[Arabidopsis-thaliana]-SRL>    (164) ---------NVVLRKLMSF---EDEDEIGPSSADDDSPGKSGLYPIEIASIVSASVIVFVLLVLVILFIYTRKWKRNSQVQVDEIKEIKVFVDI----GI
XP_020890817.1:1-541-[Arabidopsis-lyrata-subsp.-ly... (166) ---------NVVLRKLMSF---EDEDEIGPSSADDS-PGKPGLYPIEIASIVSASVIVFVLLVLVLLFLYTKKWKRNSQIQVDEIKEIKVFVDI----GI
   CAA7061053.1:3-572-[Microthlaspi-erraticum]-SRL> (178) KSLKVDPFSSNAARKLMSFNEEDEEEEASPSADHSSPSGKTKLYPIEIASIISASIIVFVLLVLVFFFVYSRKWKRNSQIQVAETKELKVFVDSSDSNSI
     KAE8732905.1:599-1052-[Hibiscus-syriacus]-SSI> (86) ------------------AN--TEDSQNAEAPPSGNTTGNNSLDPIEIASIASASAIVSVLLVLVILFFYTRKWVPKSRVQVSESREITVFVDIG----V
       XP_021905217.1:662-1147-[Carica-papaya]-PSL> (98) PYLRPCHVFSLTVPSSDPQGRVGDPQNYAASPLTTAESGKGGFNSIEIASIASASAIVSVLLALIVLFFYTRKWNPKSKIMGTVKKEVTVFTDIG----V
        KAF2599862.1:38-517-[Brassica-cretica]-SRL> (103) -----------AVRKLMSHNGEEEEDSENEASPSSDSSDKTGLYPIEIASIVSASVIVFVLLVLVVLFIYTRKGKRNSQVQVVEPKEIKVFVDN----GI
          VDD22477.1:19-515-[Brassica-oleracea]-SRL> (120) -----------AVRKLMSHNGEEEEDSENEASPSSDSSDKTGLYPIEIASIVSASVIVFVLLVLVVLFIYTRKGKRNSQVQVVEPKEIKVFVDN----GI
XP_013621334.1:19-515-[Brassica-oleracea-var.-oler... (120) -----------AVRKLMSHNGEEEEDSENEASPSSDSSDKTGLYPIEIASIVSASVIVFVLLVLVVLFIYTRKGKRNSQVQVVEPKEIKVFVDN----GI
           XP_009127716.2:20-515-[Brassica-rapa]-SRL> (120) -----------AVRKLMSHNG-EEEEEENEASSTSDSSDKTGLYPIEIASIVSASVIVFVLLVLVVLFIYTRKGKRNSQVQVVEPKEIKVFVDN----GV
          XP_013689721.1:20-515-[Brassica-napus]-SRL> (120) -----------AVRKLMSHNG-EEEEEENEESPSSDSSDKTGLYPIEIASIVSASVIVFVLLVLVVLFIYTRKGKRNSQVQVVEPKEIKVFVDN----GV
        XP_018472195.1:29-513-[Raphanus-sativus]-SRL> (113) -----------AVRKLTSLE--EGEDQEESPTTSSD---KTGLYPIEIASIVSASVIVFVLLVLVVLFIYTRKRKRNSQVQVIEPKEIKVFVDT----GV
     XP_006391037.1:18-581-[Eutrema-salsugineum]-SRL> (177) SRFSSFSGNAVRGIELRKLMSFNEEEDGVTPNSDES-PGKTGLYPIEIASIVSASVIVFVLLVLVVLFFYTRKWKRNSQIQVAETKEIKVFVDI----GL
             KFK41351.1:27-552-[Arabis-alpina]-SRL> (139) KISRFSGFSGNAVLGIRKLLSVADEGEEEVSTSSEDVR-KNGLYPIEIASIVSASVIVFVLIVLVLLFLYTRKWKRNSQVQVDVPKEIKVFVDI----GV
           VVA94581.1:29-564-[Arabis-nemorensis]-SKL> (148) LKISGFSGNAVHGIKLRKLMSVNDEDDEVSPPSSSSSSGKNGLYPIEIASIVSASVIVFVLIVLVALFLYTKKWKRNSQIQVDEPKEIKVFVDI----GI
                                                         301                                                                                                400
  NP_177087.1:1-540-[Arabidopsis-thaliana]-SRL>    (248) PLTYEIIVRATGYFSNSNCIGHGGFGSTYKAEVSPTNVFAVKRLSVGRFQGDQQFHAEISALEMVRHPNLVMLIGYHASETEMFLIYNYLSGGNLQDFIK
XP_020890817.1:1-541-[Arabidopsis-lyrata-subsp.-ly... (249) PLTYEIIVRATGYFSNSNCIGHGGFGSTYKAEVSPTNVFAVKRLSVGRFQGDQQFHAEISALEMVRHPNLVMLIGYHASETEMFLIYNYLSGGNLQDFIK
   CAA7061053.1:3-572-[Microthlaspi-erraticum]-SRL> (278) PLTYEIIVRATNYFSNSNCIGHGGFGSTYKAEVTPNHVYAVKRLSVGRFQGDQQFHAEISALETVKHPNLVVLIGYHASETEMFLIYNYLSGGNLEDFIK
```

```
KAE8732905.1:599-1052-[Hibiscus-syriacus]-SSI>          (162) PLTYETIVQATGNFSAGKRIGNGGFGATYVAEVAPETLVAVKKLAVGRFQGIQQFHAEVKTLETMRHPNLVTLIGYHASESEMFLLYNYLPGGNLENFIK
XP_021905217.1:662-1147-[Carica-papaya]-PSL>            (194) ALTYDNVVRATGNFNASNCIGNGGFGATYKAEISPGVLVAIKRLSIGRFQGVQQFHAEIKTLGRLRHPNLVTLIGYHASEAEMFLIYNYLPGGNLENFIQ
KAF2599862.1:38-517-[Brassica-cretica]-SRL>             (188) PLTYESIVRATGYFCNSNCIGHGGFGSTYKAVVSPDNVFAVKRLSIGRFQGDQQFHAEISALEMVRHPNLVMLIGYHASETEMFLIYNYLSGGNLEDFIK
VDD22477.1:19-515-[Brassica-oleracea]-SRL>              (205) PLTYESIVRATGYFCNSNCIGHGGFGSTYKAVVSPDNVFAVKRLSIGRFQGDQQFHAEISALEMVRHPNLVMLIGYHASETEMFLIYNYLSGGNLEDFIK
XP_013621334.1:19-515-[Brassica-oleracea-var.-oler...   (205) PLTYESIVRATGYFCNSNCIGHGGFGSTYKAVVSPDNVFAVKRLSIGRFQGDQQFHAEISALEMVRHPNLVMLIGYHASETEMFLIYNYLSGGNLEDFIK
XP_009127716.2:20-515-[Brassica-rapa]-SRL>              (204) PLTYESIVRATGYFSNSNCIGHGGFGSTYKAEVSPNNVFAVKRLSIGRFQGDQQFHAEISALEMVRHPNLVMLIGYHASETEMFLIYNYLSGGNLEDFIK
XP_013689721.1:20-515-[Brassica-napus]-SRL>             (204) PLTYESIVRATGYFSNSNCIGHGGFGSTYKAEVSPNNVFAVKRLSIGRFQGDQQFHAEISALEMVRHPNLVMLIGYHASETEMFLIYNYLSGGNLEDFIK
XP_018472195.1:29-513-[Raphanus-sativus]-SRL>           (193) PLTYESVVRATGYFSNSNCIGHGGFGSTYRAEVSPNTVFAVKRLSVGRFQGDQQFHAEISALEMVRHPNLVMLVGYHASETEMFLIYNYLSGGNLEDFIK
XP_006391037.1:18-581-[Eutrema-salsugineum]-SRL>        (272) PLTYESIVRATGYFSNSNCIGHGGFGSTYKAEVSPNNVLAVKRLSVGRFQGDQQFHAEISALEMVRHPNLVMLIGYHASETEMFLIYNYLSGGNLEDFIK
KFK41351.1:27-552-[Arabis-alpina]-SRL>                  (234) PLTYESIVRATGYFSNSNCIGHGGFGSTYKAEVSPDNVFAVKRLSVGRFQGDQQFHAEISALEMVRHPNLVMLIGYHASESEMFLIYNYLSGGNLEDFIK
VVA94581.1:29-564-[Arabis-nemorensis]-SKL>              (244) PLTYESIVRATGYFSNSNCIGHGGFGSTYKAEVSPNNVFAVKRLSVGRFQGDQQFHAEILALEMVRHPNLVMLVGYHASESEMFLIYNYLSGGNLEDFIK
                                                              401                                                                                                 500
NP_177087.1:1-540-[Arabidopsis-thaliana]-SRL>           (348) ERSKAAIEWKVLHKIALDVARALSYLHEQCSPKVLHRDIKPSNILLDNNYNAYLSDFGLSKLLGTSQSHVTTGVAGTFGYVAPEYAMTCRVSEKADVYSY
XP_020890817.1:1-541-[Arabidopsis-lyrata-subsp.-ly...   (349) ERSKAAIEWKVLHKIALDVARALAYLHEQCSPKVLHRDIKPSNILLDNNYNAYLSDFGLSKLLGTSQSHVTTGVAGTFGYVAPEYAMTCRVSEKADVYSY
CAA7061053.1:3-572-[Microthlaspi-erraticum]-SRL>        (378) ERSKPALEWKVLHKIALDVARALAYLHEQCSPKVLHRDIKPSNILLDSKYNAYLSDFGLSKLLGTSQSHVTTGVAGTFGYVAPEYAMTCRVSEKADVYSY
KAE8732905.1:599-1052-[Hibiscus-syriacus]-SSI>          (262) ERSTRAVDWKIIHKIALDIAHALAYLHDECTPKVLHRDVKPSNILLDNDCNAYLSDFGLSRLLGSSETHATTGVAGTFGYVAPEYAMTCRVSEKADVYSY
XP_021905217.1:662-1147-[Carica-papaya]-PSL>            (294) ERSTRAIDWRILHKIALDIARALAYLHDQCVPRVLHRDVKPSNILLDDDYNAYLSDFGLARLLGTSETHATTGVAGTFGYVAPEYAMTCRVSDKADVYSY
KAF2599862.1:38-517-[Brassica-cretica]-SRL>             (288) ERSKSALDWKVLHKIALDVARALAYLHEQCSPKVLHRDIKPSNILLDNNYNAYLSDFGLSKLLGASQSHVTTGVAGTFGYVAPEYAMTCRVSEKADVYSY
VDD22477.1:19-515-[Brassica-oleracea]-SRL>              (305) ERSKSALDWKVLHKIALDVARALAYLHEQCSPKVLHRDIKPSNILLDNNYNAYLSDFGLSKLLGASQSHVTTGVAGTFGYVAPEYAMTCRVSEKADVYSY
XP_013621334.1:19-515-[Brassica-oleracea-var.-oler...   (305) ERSKSALDWKVLHKIALDVARALAYLHEQCSPKVLHRDIKPSNILLDNNYNAYLSDFGLSKLLGASQSHVTTGVAGTFGYVAPEYAMTCRVSEKADVYSY
XP_009127716.2:20-515-[Brassica-rapa]-SRL>              (304) ERSKPALDWKILHKIALDVARALAYLHEQCSPKVLHRDIKPSNILLDNNYNAYLSDFGLSKLLGASQSHVTTGVAGTFGYVAPEYAMTCRVSEKADVYSY
XP_013689721.1:20-515-[Brassica-napus]-SRL>             (304) ERSKPALDWKILHKIALDVARALAYLHEQCSPKVLHRDIKPSNILLDNNYNAYLSDFGLSKLLGASQSHVTTGVAGTFGYVAPEYAMTCRVSEKADVYSY
XP_018472195.1:29-513-[Raphanus-sativus]-SRL>           (293) ERSKPALDWKILHKIALDVARALAYLHEQCSPKVLHRDIKPSNILLDEGYNASLSDFGLSKLLGASQSHVTTGVAGTFGYVAPEYAMTCRVSEKADVYSY
XP_006391037.1:18-581-[Eutrema-salsugineum]-SRL>        (372) ERSKPALEWKILHKIALDVARALAYLHEHCSPKVLHRDIKPSNILLDNNFNAYLSDFGLSKLLGTSQSHVTTGVAGTFGYVAPEYAMTCRVSEKADVYSY
KFK41351.1:27-552-[Arabis-alpina]-SRL>                  (334) ERSKSALEWKILHKIALDVARALAYLHEQCSPKVLHRDIKPSNILLDNNYNAYLSDFGLSKLLGTSQSHVTTGVAGTFGYVAPEYAMTCRVSEKADVYSY
VVA94581.1:29-564-[Arabis-nemorensis]-SKL>              (344) ERSKSALEWKILHKIALDVARALAYLHEQCSPKVLHRDIKPSNILLDDNNNAYLSDFGLSKLLGTSQSHVTTGVAGTFGYVAPEYAMTCRVSEKADVYSY
                                                              501                                                                                         593
NP_177087.1:1-540-[Arabidopsis-thaliana]-SRL>           (448) GIVLLELISDKRALDPSFSSHENGFNIVSWAHMMLSQGKAKEVFTTGLWETGPPDDLVEVLHLALKCTVDSLSIRPTMKQAVRLLKRIQPSRL
XP_020890817.1:1-541-[Arabidopsis-lyrata-subsp.-ly...   (449) GIVLLELISDKRALDPSFSSHENGFNIVSWAHMMLSQGKAKDVFTKGLWETSPPDDLVEVLHLALKCTVDSLSIRPTMKQAVRLLKRIQPSRL
CAA7061053.1:3-572-[Microthlaspi-erraticum]-SRL>        (478) GIVVLELISDKRALDPSFSSHENGFNIVSWAHMMLSQGKAKDVFTNGLWESGPQDDLIEVLHLALKCTVDSLSIRPTMKQAVKLLKRIQPSRL
KAE8732905.1:599-1052-[Hibiscus-syriacus]-SSI>          (362) GIVLLELMSDKKAFDPSFSSHANGFNIVSWACMLLRQGQAKDVFTTRLWETGPRDYLVELLHLAITCTVDSLTNRPTMSRVVRRLKRIQPSSI
XP_021905217.1:662-1147-[Carica-papaya]-PSL>            (394) GVVLLELLSDKKALDPSFSSYGNGFNIVQWACMLLRQGRAKEFFTAGLWDAGPHDDLVEVLHLAVVCTVDSLSTRPTMKQVVRRLKQLQPPSL
KAF2599862.1:38-517-[Brassica-cretica]-SRL>             (388) GIVILELISDKRALDPSFSSHENGFNIVSWAHMMLSQGKAKEVFTKGLWETGPQDDLVEVLHLALKCTVDSLSIRPTMKQAVKQLKRIHPSRL
VDD22477.1:19-515-[Brassica-oleracea]-SRL>              (405) GIVILELISDKRALDPSFSSHENGFNIVSWAHMMLSQGKAKEVFTKGLWETGPQDDLVEVLHLALKCTVDSLSIRPTMKQAVKQLKRIHPSRL
XP_013621334.1:19-515-[Brassica-oleracea-var.-oler...   (405) GIVILELISDKRALDPSFSSHENGFNIVSWAHMMLSQGKAKEVFTKGLWETGPQDDLVEVLHLALKCTVDSLSIRPTMKQAVKQLKRIHPSRL
XP_009127716.2:20-515-[Brassica-rapa]-SRL>              (404) GIVILELISDKRALDPSFSSHENGFNIVSWAHMMLSQGKAKEVFTKGLWETGPQDDLVEVLHLALKCTVDSLSIRPTMKQAVKQLKRIHPSRL
XP_013689721.1:20-515-[Brassica-napus]-SRL>             (404) GIVILELISDKRALDPSFSSHENGFNIVSWAHMMLSQGKAKEVFTKGLWETGPQDDLVEVLHLALKCTVDSLSIRPTMKQAVKQLKRIHPSRL
XP_018472195.1:29-513-[Raphanus-sativus]-SRL>           (393) GIVILELISDKRALDPSFSSHENGFNIVSWAHMMLSQGKAKEVFTKGLWENGPQDDLVEVLHLALKCTVDSLSIRPTMKQAVKQLKRIHPSRL
XP_006391037.1:18-581-[Eutrema-salsugineum]-SRL>        (472) GIVILELISDKRALDPSFSSHENGFNIVSWAHMMLSQGKAKEVFTKGLWETGPQDDLVEVLHLALKCTVDSLSIRPTMKQAVKLLKRIQPSRL
KFK41351.1:27-552-[Arabis-alpina]-SRL>                  (434) GIVILELISDKKALDPSFSSHENGFNIVSWAHMMLSKGKAKEVFTAGLWESGPQDDLIEVLHLALKCTVDSLSIRPTMKHAVKLLKRIQPSRL
VVA94581.1:29-564-[Arabis-nemorensis]-SKL>              (444) GIVILELISDKRALDPSFSSHENGFNIVSWAHMMLSKGKAKEVFTAGLWETGPQDDLVEVLHLALKCTVDSLSIRPTMKQAVKLLKRIQPSKL
```

# K15_AT3G08720.1

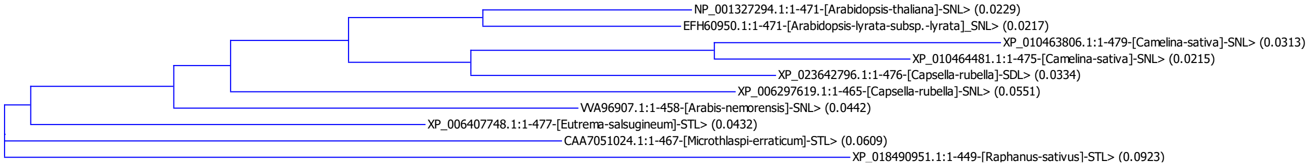


```
                                                                 1                                                                                                  100
   NP_001327294.1:1-471-[Arabidopsis-thaliana]-SNL>        (1)   MVSSQC-SVANKNQTGKPFQKHLSLSISPPKSVLGDNLELQFSDVFGPMPEANS-EEACDVAYDEPAVVYSRSHSLVGPSLVVSHSLKMNKLTLRETEDS
EFH60950.1:1-471-[Arabidopsis-lyrata-subsp.-lyrata...      (1)   MVSSQC-PVANKNQTGKPFQKQLYLSISPPKSVLNDNLELEFSDVFGPMPEANS-GEASDVAYDEPAVVYSRSHSLVGPSLVVSHSLKLNKLTLRETEDS
     XP_010463806.1:1-479-[Camelina-sativa]-SNL>           (1)   MVSSPCPVVANKNLTGKPFQKHLYLSISPPKPAFNDNLELDFSDVFGPMPEANSSGDPSAVLCDEPSVVYSRSHSLVGPSSVVSLSLNLNKLTLLETKNS
     XP_010464481.1:1-475-[Camelina-sativa]-SNL>           (1)   MVSSQCPVVANTNLTGKPFQKHLYLSISPPKPVFNDNLELDFSDVFGPMPETNSSEDPSDVLCDEPAVVYSRSHSLVGPSSVVNLSLNLNKLTLRETKNS
    XP_023642796.1:1-476-[Capsella-rubella]-SDL>           (1)   MVSSQC-PVANNNLTGKPLQKQLYLSITPPKSVFSDNLELDFSDVFGPMPEANSSGEASDVLLDEPAVVYSRSHSLVGPSSLDSHSLKLTKLTLRETKDS
    XP_006297619.1:1-465-[Capsella-rubella]-SNL>           (1)   MVSSQR-PVPNKIQ--K----QQYLSISSSDSVLKDDVELEFSDVFGPLPEEVN-----DIVYDEPAVVYSRSHSLVGPSSLDSHSLKLTKLTLRETKDS
       VVA96907.1:1-458-[Arabis-nemorensis]-SNL>           (1)   MVSSHC-PVSDKN---K---KQLYLSISPSNSVMKDSVELDFSDVFGPLPEEAS-----DVVFDEPAVVYSRSHSLVGPSLLSSHSFKLNKLTLRETEDS
  XP_006407748.1:1-477-[Eutrema-salsugineum]-STL>          (1)   MVSSECSGVANKNLTGKAFHKQLCLTITPPHAVLKDDVELEFSDVFGPLPETNNSGEDGDVVYDEPAVVYSRSHSLVGPSSIGGHSFKLNKLTLRETEDS
CAA7051024.1:1-467-[Microthlaspi-erraticum]-STL>           (1)   MVSSDC-LVADKS--GKLFQKQLCLSITPPSSVLNENVELDFSDVFGPLPEEEAS----DAAYDEPAVVYSRSHSLVGPSVIGCQSFKLNKLTLRETSDS
     XP_018490951.1:1-449-[Raphanus-sativus]-STL>          (1)   MVSSEC-PVANKNLTGK----RMFLTITPPQAVLKESLELDFSDVFGPLPEDHP--TDVAAFDEGPAVIHSRSHSLVGPSLINSHSFKLSSKLTLECLEG
                                                                 101                                                                                                200
   NP_001327294.1:1-471-[Arabidopsis-thaliana]-SNL>       (99)   VDLVE--CVEGESIKENDEFSGN-DDTDSEKSPE----EVSGVVGIEDFEVLKVVGQGAFGKVYQVRKKDTSEIYAMKVMRKDKIVEKNHAEYMKAERDI
EFH60950.1:1-471-[Arabidopsis-lyrata-subsp.-lyrata...     (99)   VDLVE--CVEGESIKENDEFFGN-DDTDSDKALE----EVSGVVGIEDFEVLKVVGQGAFGKVYQVRKKDTSEIYAMKVMRKDKIVEKNHAEYMKAERDI
     XP_010463806.1:1-479-[Camelina-sativa]-SNL>         (101)   IDLVEQECLESEAIKEIDEFSGN-DDTDSDKSLDGELGEVSGVVGIEDFEVLKVVGQGAFGKVYQVRKKDTSEIYAMKVMRKDKIVEKNHAEYMKAERDI
     XP_010464481.1:1-475-[Camelina-sativa]-SNL>         (101)   IDLVE-----EECLEKNDEFSGNDEDTDSDKSPEGELGEVSGVVGIEDFEVLKVVGQGAFGKVYQVRKKDTSEIFAMKVMRKDKIVEKNHAEYMKAERDI
    XP_023642796.1:1-476-[Capsella-rubella]-SDL>         (100)   IDLVE--CLEGESLKENDEFSGN-EDSDSDKTPEGDLGEVSGVVGIEDFEILKVVGQGAFGKVYQVRKKDTSEIYAMKVMRKDKIVEKNHAEYMKAERDI
    XP_006297619.1:1-465-[Capsella-rubella]-SNL>          (89)   IDLVE--CLEGELLKENDEFSGN-DDSDSDKVPKGDLAKVSGVVGIDDFEVMKVVGKGAFGKVYQVRKKETSEIYAMKVMRKDQIMEKNHAEYMKAERDI
       VVA96907.1:1-458-[Arabis-nemorensis]-SNL>          (89)   VDLVECLE---------GESSGN-EDTDSEKAAEGDLVKVSGVVGIEDFEVMKVVGKGAFGKVYQVRKKETSEIYAMKVMRKDHIMEKNHAEYMKAERDI
  XP_006407748.1:1-477-[Eutrema-salsugineum]-STL>        (101)   IELLE--CLEGESLKESDEISGN-DDTDNEKSPEGDVVKVSGVVGIEDFEVLKVVGQGAFGKVYQVRKKETSEIYAMKVMRKDKIMEKNHAEYMKAERDI
CAA7051024.1:1-467-[Microthlaspi-erraticum]-STL>          (94)   VDLVEC-----ESLKENDELSGS-DDTDNEKAPEGDLVKVSGVVGIDDFEVLKVLGQGAFGKVYQVRKKDTYEIYAMKVMRKDKIMEKNHAEYMKAERDI
     XP_018490951.1:1-449-[Raphanus-sativus]-STL>         (94)   ESSCN-------------------EDTETEKPKK-----VSGGVGIEDFEVLKVVGQGAFGKVYQVRKKETCEIYAMKVMRKDKIMEKNHAEYMKAERDI
                                                                 201                                                                                                300
   NP_001327294.1:1-471-[Arabidopsis-thaliana]-SNL>      (192)   LTKIDHPFIVQLKYSFQTKYRLYLVLDFINGGHLFFQLYHQGLFREDLARVYTAEIVSAVSHLHEKGIMHRDLKPENILMDVDGHVMLTDFGLAKEFEEN
EFH60950.1:1-471-[Arabidopsis-lyrata-subsp.-lyrata...    (192)   LTKIDHPFIVQLKYSFQTKYRLYLVLDFINGGHLFFQLYHQGLFREDLARVYTAEIVSAVSHLHEKGIMHRDLKPENILMDVDGHVMLTDFGLAKEFEEN
     XP_010463806.1:1-479-[Camelina-sativa]-SNL>         (200)   LTKIDHPFIVQLKYSFQTKYRLYLVLDFINGGHLFFQLYHQGLFREDLARVYTAEIVSAVSHLHEKGIMHRDLKPENILMDVDGHVMLTDFGLAKEFEEN
     XP_010464481.1:1-475-[Camelina-sativa]-SNL>         (196)   LTKIDHPFIVQLKYSFQTKYRLYLVLDFINGGHLFFQLYHQGLFREDLARAYTAEIVSAVSHLHEKGIMHRDLKPENILMDVDGHVMLTDFGLAKEFEEN
    XP_023642796.1:1-476-[Capsella-rubella]-SDL>         (197)   LTKIDHPFIVQLKYSFQTKYRLYLVLDFINGGHLFFQLYHQGLFREDLARVYTAEIVSAVSHLHEKGIMHRDLKPENILMDVDGHVMLTDFGLAKEFEEN
    XP_006297619.1:1-465-[Capsella-rubella]-SNL>         (186)   LTKIDHPFIVQLKYSFQTKYRLYLVLDFINGGHLFFQLYHQGLFREDLARVYTAEIVSAVSHLHEKGIMHRDLKPENILMDTDGHVMLTDFGLAKEFEEN
       VVA96907.1:1-458-[Arabis-nemorensis]-SNL>         (179)   LTKIDHPFIVQLKYSFQTKYRLYLVLDFINGGHLFFQLYHQGLFREDLARVYTAEIVSAVSHLHEKGIMHRDLKPENILMDADGHVMLTDFGLAKEFEEN
  XP_006407748.1:1-477-[Eutrema-salsugineum]-STL>        (198)   LTKTDHPFIVQLKYSFQTKYRLYLVLDFINGGHLFFQLYHQGLFREDLARVYTAEIVSAVSHLHENGIMHRDLKPENILMDTDGHVMLTDFGLAKEFEEN
CAA7051024.1:1-467-[Microthlaspi-erraticum]-STL>         (188)   LTKIDHPFIVQLNYSFQTKYRLYLVLDFINGGHLFFQLYHQGLFREDLARVYTAEIVSALSHLHEKGIMHRDLKPENILMDVDGHVMLTDFGLAKEFDEN
     XP_018490951.1:1-449-[Raphanus-sativus]-STL>        (170)   LTKIDHPFIVQLKYSFQTKYRLYLVLDFINGGHLFFQLYHQGLFREELARVYTAEIVSAVSHLHENGIMHRDLKPENILMDTDGHVMLTDFGLAKEFDEN
                                                                 301                                                                                                400
   NP_001327294.1:1-471-[Arabidopsis-thaliana]-SNL>      (292)   TRSNSMCGTTEYMAPEIVRGKGHDKAADWWSVGILLYEMLTGKPPFLGSKGKIQQKIVKDKIKLPQFLSNEAHALLKGLLQKEPERRLGSGPSGAEEIKK
EFH60950.1:1-471-[Arabidopsis-lyrata-subsp.-lyrata...    (292)   TRSNSMCGTTEYMAPEIIHGKGHDKAADWWSVGILLYEMLTGKPPFLGSKGKIQQKIVKDKIKLPQFLSNEAHALLKGLLQKEPERRLGSGPSGADEIKQ
     XP_010463806.1:1-479-[Camelina-sativa]-SNL>         (300)   TRSNSMCGTTEYMAPEIVRGKGHNKAADWWSVGILLYEMLTGKPPFVGSKGTIQQRIVKEKIKLPKFLSNEAHALLKGLLAKEPERRLGSGPTGAEEIKK
     XP_010464481.1:1-475-[Camelina-sativa]-SNL>         (296)   TRSNSMCGTTEYMAPEIVRGKGHDKAADWWSVGILLYEMLTGKPPFVGSKGKIQQSIVKEKIKLPKFLSNEAHALLKGLLAKEPERRLGSGPTGAEEIKK
    XP_023642796.1:1-476-[Capsella-rubella]-SDL>         (297)   TRSNSMCGTTEYMAPEIVRGKGHDKAADWWSVGILLYEMLTGKPPFVGGKGMIQQKIVKDKIKLPKFLSNEAHALLKGLLQKEPGRRLGSGPTGAEEIKQ
    XP_006297619.1:1-465-[Capsella-rubella]-SNL>         (286)   TRSNSMCGTTEYMAPEIVRGKGHDKAADWWSVGILLYEMLTGKPPFMGSKGKIQQKIVKDKIKLPQFLSNEAHAMLKGLLQKEPERRLGSGPTGAEEIKQ
       VVA96907.1:1-458-[Arabis-nemorensis]-SNL>         (279)   TRSNSMCGTTEYMAPEIVRGKGHDKAADWWSVGILLYEMLTGKPPFLGGKGKIQQKIVKDKIKLPQFLSNEAHALLKGLLQKEPERRLGSGPSGAEEIKE
  XP_006407748.1:1-477-[Eutrema-salsugineum]-STL>        (298)   TRSNSMCGTTEYMAPEIVRGKGHDKAADWWSVGILLYEMLTGKPPFLGSKGKIQQKIVKDKIKLPQFLSTEAHALLKGLLQKEPERRLGSGPSGAEEIKE
CAA7051024.1:1-467-[Microthlaspi-erraticum]-STL>         (288)   KRSNSMCGTTEYMAPEIVRGKGHDKAADWWSVGILLYEMLTGKPPFLGSKGKIQQKIVKDKIKLPQFLSSEAHALLKGLLQKEPERRLGSGPSGAEEIKK
     XP_018490951.1:1-449-[Raphanus-sativus]-STL>        (270)   KRSNSMCGTTEYMAPEIVRGKGHDKAADWWSVGILLYEMLTGKPPFLGSKGKIQQKIVKDKIKLPPFLSNEAHALLKGLLQKEPERRLGSGPSGAEEIKG
                                                                 401                                                                            480
   NP_001327294.1:1-471-[Arabidopsis-thaliana]-SNL>      (392)   HKWFKAINWKKLEAREVQPSFKPAVSGRQCIANFDKCWTDMSVLDSPASSPNSDAKANPFTNFTYVRPPHSFLHRTTSNL
EFH60950.1:1-471-[Arabidopsis-lyrata-subsp.-lyrata...    (392)   HKWFKVINWKKLEAREVQPSFKPAVSGRQCIANFDKCWTEMSVLDSPASSPNSDPKANPFSNFTYVRPPPSFLHRTTSNL
     XP_010463806.1:1-479-[Camelina-sativa]-SNL>         (400)   HKWFRGINWKKLEAREVQPSFKPEVSGRQCIANFDKCWTEMSVFDSPASSPSSDPKANPFTNFTYVRPPPSFLHRSTSNL
     XP_010464481.1:1-475-[Camelina-sativa]-SNL>         (396)   HKWFKGINWKKLEAREVQPSFKPEVSGRQCIANFDKCWTEMSVLDSPASSPSSDPKANPFTNFTYVRPPPSFLHRSTSNL
    XP_023642796.1:1-476-[Capsella-rubella]-SDL>         (397)   HKWFKGINWKKLEAREVQPSFKPEVSGRHCIANFDKCWTEMSVLDSPASSPSSDPKANPFTNFTYVRPPPSFLHRSTSDL
    XP_006297619.1:1-465-[Capsella-rubella]-SNL>         (386)   HKWFKGINWKKLEAREVMPSFKPEVSGRQCIANFDKCWTEMSVLDSPASSPSSDPKANPFSNFTYVRPPPSFLHRSTSNL
       VVA96907.1:1-458-[Arabis-nemorensis]-SNL>         (379)   HKWFKGINWKKLEAREVKPSFRPEVSGRQCIANFDKCWTDMSVLDSPASSPNSDPKANPFSNFTYVRPPPSFLNQTTSNL
  XP_006407748.1:1-477-[Eutrema-salsugineum]-STL>        (398)   HKWFKGMNWKKLEAREVKPSFKPEVSGRQCIANFDKCWTEMSVLDSPASSPSSDPKANPFTNFTYVRPPPSFLQQSTSTL
CAA7051024.1:1-467-[Microthlaspi-erraticum]-STL>         (388)   HKWFKGMNWKKVEAREVKPSFKPAVLGIQCIANFDKCWTDMSVLDSPASSPSSDPKANPFTNFTYVRPPASFLQRTTSTL
     XP_018490951.1:1-449-[Raphanus-sativus]-STL>        (370)   HKWFKGMNWKKVEAREVKPSFKPEVSGRQCIANFDKCWTDMSVLDSPASSPSSDPEANPFTNFTYVRPPHSFLNQTTSTL
```

# K16_ AT3G20530.1

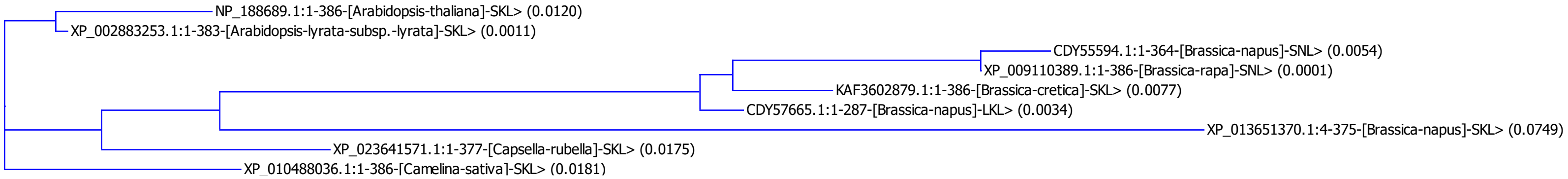


```
                                                         1                                                                                                 100
      NP_188689.1:1-386-[Arabidopsis-thaliana]-SKL>    (1) MKINCLFCCMSHRRFNRRSSSRQSIKDCIDAKNNITTFDNISFKTDSSRRRYISEEIAKLGKGNISAHIFTFRELCVATKNFNPDNQLGEGGFGRVYKGQ
XP_002883253.1:1-383-[Arabidopsis-lyrata-subsp.-ly...    (1) MKINCLFCCMSERRFTRRSSSRQSIKDCIDAKNNITTFDNISFKTDSSRRRYISEEIAKLGKGNISAHIFTFRELCVATKNFNPDNQLGEGGFGRVYKGH
           CDY55594.1:1-364-[Brassica-napus]-SNL>    (1) MKLNCLFCCMSERRFTRRSSSIPSIKDCIHANNNLSRFDNISFKTDSSRRRYISEEIAKLGKGNISAHIFTFRELRVATKNFNPENQLGEGGFGRVYKGH
         XP_009110389.1:1-386-[Brassica-rapa]-SNL>    (1) MKLNCFFCCMSERRFTRRSSSIPSIKDCIHANNNLSRFDNISFKTDSSRRRYISEEIAKLGKGNISAHIFTFRELRVATKNFNPENQLGEGGFGRVYKGH
       KAF3602879.1:1-386-[Brassica-cretica]-SKL>    (1) MKMNCLFCCMSERRFTRRSSSRPSIKDCINANNNLSRFDNISFKTDSSRRRYISEEIAKLGKGNISAQIFTFRELCVATKNFNPENQLGEGGFGRVYKGH
           CDY57665.1:1-287-[Brassica-napus]-LKL>    (1) ---------------------------------------------------------------------------------------------------H
       XP_013651370.1:4-375-[Brassica-napus]-SKL>    (1) ----CLFCCMSERRFTRRSSSRQS----------IKEFDNISFKTDSSRRRYIYEEITKLGRRNISAQIFTFQDLSVATRNFDPENQLGEGGFGRVYKGY
    XP_023641571.1:1-377-[Capsella-rubella]-SKL>    (1) MKINCLLCCMSERRFTRRSSSRQSIKDCIDAKNNITTFDNISFKTDSSRRRYISEEIAKLGKGNISAHIFTFRELCAATKNFNPDNQLGEGGFGRVYKGH
     XP_010488036.1:1-386-[Camelina-sativa]-SKL>    (1) MKINCLFCCMSEKRFTRRSSSRQSIKDCIDAKNNITTFENISFKTDSSRRKYIAEEIAKLGKGNISAHIFTFRELCVATKNFNPDNQLGEGGFGRVYKGH
                                                         101                                                                                               200
      NP_188689.1:1-386-[Arabidopsis-thaliana]-SKL>  (101) IETPEQVVAVKQLDRNGYQGNREFLVEVMMLSLLHHQNLVNLVGYCADGDQRILVYEYMQNGSLEDHLLELARNKKKPLDWDTRMKVAAGAARGLEYLHE
XP_002883253.1:1-383-[Arabidopsis-lyrata-subsp.-ly...  (101) IETPEQVVAVKQLDRNGYQGNREFLVEVMMLSLLHHQNLVNLVGYCADGDQRILVYEYMQNGSLEDHLLELARNKKKPLDWDTRMKVAAGAARGLEYLHE
           CDY55594.1:1-364-[Brassica-napus]-SNL>  (101) IETPEKVVAVKQLDRNGYQGNREFLVEVMMLSLLHHQNLVNLVGYCADGDQRILVYEYMQNGSLEDHLLELVRNKKKPLDWDTRMKVAAGAARGLEYLHE
         XP_009110389.1:1-386-[Brassica-rapa]-SNL>  (101) IETPEKVVAVKQLDRNGYQGNREFLVEVMMLSLLHHQNLVNLVGYCADGDQRILVYEYMQNGSLEDHLLELVRNKKKPLDWDTRMKVAAGAARGLEYLHE
       KAF3602879.1:1-386-[Brassica-cretica]-SKL>  (101) IETPEKVVAVKQLDRNGYQGNREFLVEVMMLSLLHHQNLVNLVGYCADGDQRILVYEYMQNGSLEDHLLELVRNKKKPLDWDTKMKVAAGAARGLEYLHE
           CDY57665.1:1-287-[Brassica-napus]-LKL>    (2) IETPEKVVAVKQLDRNGYQGNREFLVEVMMLSLLHHQNLVNLVGYCADGDQRILVYEYMQNGSLEDHLLELVRNKKKPLDWDTRMKVAAGAASGLEYLHE
       XP_013651370.1:4-375-[Brassica-napus]-SKL>   (87) IETNDKVVAVKQLDRNGYQGNREFLVEVMMLGLLHHQNLVNLVGYCADGDQRILVYEYMQNGSLEDHLLELVRNKKKPLDWDTRMKVAAGAARGLEYLHE
    XP_023641571.1:1-377-[Capsella-rubella]-SKL>  (101) IETPEKVVAVKQLDRNGYQGNREFLVEVMMLSLLHHQNLVNLVGYCADGDQRILVYEYMQNGSLEDHLLELTRNKKKPLDWDTRMKIAAGAARGLEYLHE
     XP_010488036.1:1-386-[Camelina-sativa]-SKL>  (101) IETPEKVVAVKQLDRNGYQGNREFLVEVMMLSLLHHQNLVNLVGYCADGDQRILVYEYMQNGSLEDHLLELVRNKKKPLDWDTRMKVAAGAARGLEYLHE
                                                         201                                                                                               300
      NP_188689.1:1-386-[Arabidopsis-thaliana]-SKL>  (201) TADPPVIYRDFKASNILLDEEFNPKLSDFGLAKVGPTGGETHVSTRVMGTYGYCAPEYALTGQLTVKSDVYSFGVVFLEMITGRRVIDTTKPTEEQNLVT
XP_002883253.1:1-383-[Arabidopsis-lyrata-subsp.-ly...  (201) TADPPVIYRDFKASNILLDEEFNPKLSDFGLAKVGPTGGETHVSTRVMGTYGYCAPEYALTGQLTVKSDVYSFGVVFLEMITGRRVIDTTKPTQEQNLVT
           CDY55594.1:1-364-[Brassica-napus]-SNL>  (201) TADPPVIYRDFKASNILLDEEFNPKLSDFGLAKVGPTGGETHVSTRVMGTFG----------------------VVFLEMITGRRVIDTKKPTQEQNLVT
         XP_009110389.1:1-386-[Brassica-rapa]-SNL>  (201) TADPPVIYRDFKASNILLDEEFNPKLSDFGLAKVGPTGGETHVSTRVMGTYGYCAPEYALTGQLTVKSDVYSFGVVFLEMITGRRVIDTKKPTQEQNLVT
       KAF3602879.1:1-386-[Brassica-cretica]-SKL>  (201) TADPPVIYRDFKASNILLDEEFNPKLSDFGLAKVGPTGGETHVSTRVMGTYGYCAPEYALTGQLTVKSDVYSFGVVFLEMITGRRVIDTKKPTQEQNLVT
           CDY57665.1:1-287-[Brassica-napus]-LKL>  (102) TADPPVIYRDFKASNILLDEEFNPKLSDFGLAKVGPTGGETHVSTRVMGTYGYCAPEYALTGQLTVKSDVYSFGVVFLEMITGRRVIDTKKPTREQNLVT
       XP_013651370.1:4-375-[Brassica-napus]-SKL>  (187) TADPPVIYRDFKTSNILLDEEFNPKLSDFGLAKVGPTGGETHVSTRVMGTYGYCAPEYALTGQLTVKSDVYSFGVVFLEMITGRRVIDPTKPTQEENLVT
    XP_023641571.1:1-377-[Capsella-rubella]-SKL>  (201) TADPPVIYRDFKASNILLDEEFNPKLSDFGLAKVGPTGGETHVSTRVMGTYGYCAPEYALTGQLTVKSDVYSFGVVFLEMITGRRVIDTTKSTEEQNLVT
     XP_010488036.1:1-386-[Camelina-sativa]-SKL>  (201) TADPPVIYRDFKASNILLDEEFNPKLSDFGLAKVGPTGGETHVSTRVMGTYGYCAPEYALTGQLTVKSDVYSFGVVFLEMITGRRVIDTTKPTEEQNLVT
                                                         301                                                                                386
      NP_188689.1:1-386-[Arabidopsis-thaliana]-SKL>  (301) WASPLFKDRRKFTLMADPLLEGKYPIKGLYQALAVAAMCLQEEAATRPMMSDVVTALEYLAVTKTEEDGQTVEGEEEEEEDERSKL
XP_002883253.1:1-383-[Arabidopsis-lyrata-subsp.-ly...  (301) WASPLFKDRRKFTLMADPLLEGKYPIKGLYQALAVAAMCLQEEAATRPMMSDVVTALEYLAMTKTEEDGQTVE---EEEEDERSKL
           CDY55594.1:1-364-[Brassica-napus]-SNL>  (279) WASPLFKDKRKFTLMADPLLEGKYPTKGLYQALAVAAMCLQEEAETRPTMSDVVTALEYLGVTKVEENNETDNNNKEGGEDERSNL
         XP_009110389.1:1-386-[Brassica-rapa]-SNL>  (301) WASPLFKDKRKFTLMADPLLEGKYPTKGLYQALAVAAMCLQEEAETRPTMSDVVTALEYLGVTKVEENNETDNNNKEGGEDERSNL
       KAF3602879.1:1-386-[Brassica-cretica]-SKL>  (301) WASPLFKDKRKFTLMADPLLEGKYPIKGLYQALAVAAMCLQEEAETRPTMSDVVTALEYLGVTKVEENNETDNNKKEEGEDERSKL
           CDY57665.1:1-287-[Brassica-napus]-LKL>  (202) WASPLFKDKRKFTLMADPLLEGKYPIKGLYQALAVAAMCLQEEAETRPTMSDVVTALEYLGVTKVEENNETDNNKKEEGEDERLKL
       XP_013651370.1:4-375-[Brassica-napus]-SKL>  (287) WASPLFKDRRKFTLMADPLLEGKYPIIGLYQALAVAAMCLQEEAATRPMMSDVVSALEYLAMTKDKVDAEAVEDKDREEGEEKSKL
    XP_023641571.1:1-377-[Capsella-rubella]-SKL>  (301) WASPLFKDRRKFTLMADPLLEGKYPIKGLYQALAVAAMCLQEEAATRPMMSDVVTALEYLAMKTEEDG---------EEEDERSKL
     XP_010488036.1:1-386-[Camelina-sativa]-SKL>  (301) WASPLFKDRRKFTLMVDPLLEGKYPIKGLYQALAVAAMCLQEEAATRPMMSDVVTALEYLAMTKTEEDGQTVEEEEEQEEDERSKL
```

# K17_ AT3G17420.1

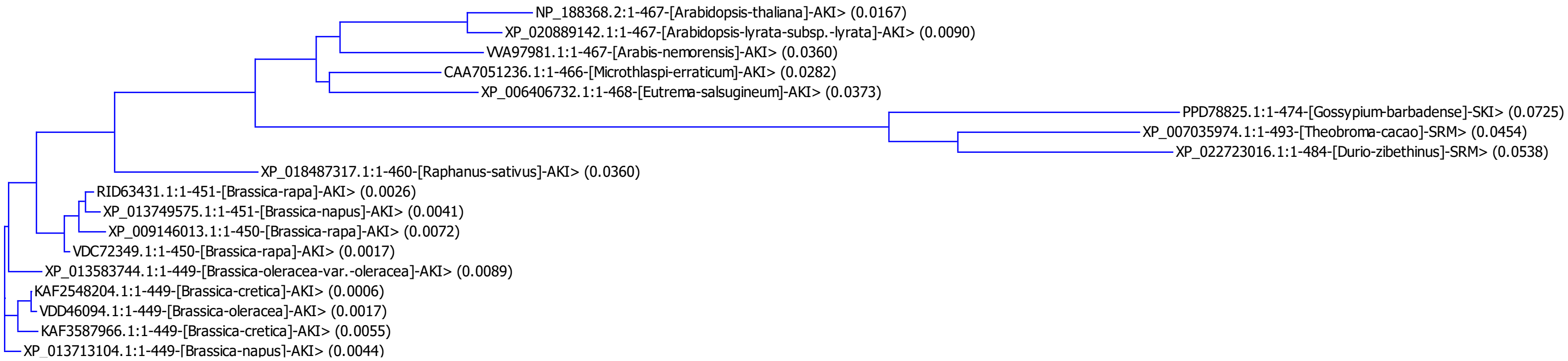


```
                                                              1                                                                                                  100
NP_188368.2:1-467-[Arabidopsis-thaliana]-AKI>           (1)   MTSQLKRTLTKRYGVLELWEIIVIALFAAFIVILVLSVWLSFRKKSKRSNATTLPVTQS-PRFTEEIKEISVDHGSSNNNG-------TSYQTLDEKF--
XP_020889142.1:1-467-[Arabidopsis-lyrata-subsp.-ly...   (1)   MASQLKRTLTKRYGVLELWEIIVIALFAVFIVILVISVWLSFRKKSKRSNATTLPVTQS-PRFTEEIKEISVDHVSSNNNG-------TSYPTLDEKF--
VVA97981.1:1-467-[Arabis-nemorensis]-AKI>               (1)   MASELKRTLTKRYGALQLWEIIVIAIFAAFIVILAISVWLSFRKKSRRSRFTQLPATQS-PRFPEEIKEISVDHVTSSNNG-------SSHHTLDEKFG-
CAA7051236.1:1-466-[Microthlaspi-erraticum]-AKI>        (1)   MASELKQTLTKRYGALELWEIIVIALFAAFIVILAISVWLSFRKKSRRSNFIQLPVTQSSPRLPEEIKEINVDHVSSNNNNNNGTTTTTSHQTLDEKFS-
XP_006406732.1:1-468-[Eutrema-salsugineum]-AKI>         (1)   MASELKQTLTKRYGALQLWEIIVIALFAAFIVILGISVWLSFRKKSRRPNLTQLPVAQS-PRLPEEIKEISVDHVSSNSNG-------TCHQTLDEKFGE
PPD78825.1:1-474-[Gossypium-barbadense]-SKI>            (1)   MASDLNKTLSKTYIGLQLWVLIVICLGIVFLVILGISLWLSFRKKTGRHK--DILPSRQAPYVSEEIKEIGVDEVSANNGS---------PNTLNYKFSD
XP_007035974.1:1-493-[Theobroma-cacao]-SRM>             (1)   MASDLNKSLSKTYIGLQLWVLIVIGLGVVFLVILGISLWLSFRKKSRRAN--DILPVRQAPYVSEEIKEIRVDQVSANSGG---------LNTLNDKFSD
XP_022723016.1:1-484-[Durio-zibethinus]-SRM>            (1)   MASDLNRTLSKTYISLQLWVLIVIGLGVVFLVILGISLWLSFRKKSRRPN--GMLPVRQALYVSEEIKEIRVDQVSANNAC---------LNTLNNKFSD
XP_018487317.1:1-460-[Raphanus-sativus]-AKI>            (1)   MASELKQTLTKKYGALELWEIIVIALFASFILILAVSVWLSFRKKSKRSNFIQLLPITETPRHQDEIKDISVDHVSSNNNVTP-------HQALDDKFG-
RID63431.1:1-451-[Brassica-rapa]-AKI>                   (1)   MASELKQTLTKRYGALELWEIIVIALFAAFILILAVSVWLSFRKKSKRSHFIQLLPITETPRHQDEIKDISVDHVSSHNNN---------GTPLDEKFS-
XP_013749575.1:1-451-[Brassica-napus]-AKI>              (1)   MASELKQTLTKRYGALELWEIIVIALFAAFILILAVSVWLSFRKKSKRSHFIQLLPITETPRHQDEIKDISVDHVSSHNNN---------GTPLDEKFS-
XP_009146013.1:1-450-[Brassica-rapa]-AKI>               (1)   MASELKQTLTKRYGAVELWEIIVIALFAAFILILAVSVWLSFRKKSKRSHFIQLLPITETPRHQDEIKDISVDHVSSHNNN---------GTPLDEKFS-
VDC72349.1:1-450-[Brassica-rapa]-AKI>                   (1)   MASELKQTLTKRYGALELWEIIVIALFAAFILILAVSVWLSFRKKSKRSHFIQLLPITETPRHQDEIKDISVDHVSSNNNN---------GTPLDDKFS-
XP_013583744.1:1-449-[Brassica-oleracea-var.-olera...   (1)   MASELKQTLTKRYGALELWEIIVIALFAAFILILAVSVWLSFRKKSKRSNFIQLLPTTETPRHQDEIKDISVVHVSSDNNG----------TPLDDKFP-
KAF2548204.1:1-449-[Brassica-cretica]-AKI>              (1)   MASELKQTLTKRYGALELWEIIVIALFAAFILILAVSVWLSFRKKSKRSNFIQLLPTTDTPRHQDEIKDISVVHVSSNKNG----------TPLDDKFP-
VDD46094.1:1-449-[Brassica-oleracea]-AKI>               (1)   MASELKQTLTKRYGALELWEIIVIALFAAFILILAVSVWLSFRKKCKRSNFIQLLPTTDTPRHQDEIKDISVVHVSSNKNG----------TPLDDKFP-
KAF3587966.1:1-449-[Brassica-cretica]-AKI>              (1)   MASELKQTLTKRYGALELWEIIVIALFAAFMLILAVSVWLSFRKKCKRSNFIQLLPTTDTPRHQDEIKDISVVHVSSNKNG----------TPLDDKFP-
XP_013713104.1:1-449-[Brassica-napus]-AKI>              (1)   MASELKQTLTKRYGALELWEIIVIALFAAFILILAVSVWLSFRKKSKRSNFIQLLPTTDTPRHQDEIKDISVVHVSSDNNG----------TPLDDKFR-
                                                              101                                                                                                200
NP_188368.2:1-467-[Arabidopsis-thaliana]-AKI>          (91)   ------VEDIENGDKFS-----GSLEKKPLVG-----------SHLPPSTPSTTAPSPLLGLPEVSHIGWGHWFTLRDLQLATNHFSKESIIGDGGYGVV
XP_020889142.1:1-467-[Arabidopsis-lyrata-subsp.-ly...  (91)   ------VEDIENGDKFS-----GSLEKKPAVG-----------SHLPPSTPLTTAPSPLLGLPEVSHIGWGHWFTLRDLQLATNHFSKENIIGDGGYGVV
VVA97981.1:1-467-[Arabis-nemorensis]-AKI>              (92)   ------EKDIENGDKYS-----GSLEKKTTVE-----------SHLAPTTPPTTSPSPLSGLPEVSHIGWGHWFTLRDLQLATNHFAKENIIGDGGYGVV
CAA7051236.1:1-466-[Microthlaspi-erraticum]-AKI>      (100)   ------KNDIENGNNYS-----GSLEKK---------------SHLPPSTS-----SPISGLPEDSHIGWGHWFTLRDLQVATNHFSKENIIGDGGYGVV
XP_006406732.1:1-468-[Eutrema-salsugineum]-AKI>        (93)   ------NNDIENGNNYS-----GSLEKKTPVG-----------SHLPCSTSPTTSPSPLSGLPEVSHIGWGHWFTLRDLQLATNQFAKENIIGDGGYGVV
PPD78825.1:1-474-[Gossypium-barbadense]-SKI>           (90)   RDSDKVSYHVGNGDDSGLSGSFNHLGKDVKGSQPAEEKGVGVVSVFRPSSHPLTAPSPLSGLPEFSHLGWGHWFTLRDLQLATSRFSKDNIIGDGGYGFV
XP_007035974.1:1-493-[Theobroma-cacao]-SRM>            (90)   RDSEKVVFSVDNGDDSGQSGSFNHVEKDVKGSQPGEEGGNGTVSTYRPSSHPLTAPSPLSGLPEFSHLGWGHWFTLRDLQLSTNRFSKDNIIGDGGYGVV
XP_022723016.1:1-484-[Durio-zibethinus]-SRM>           (90)   RDSEKVLFDVDNGDDSGQSGSFNHLEKDVKGSQPKEEGGTESVSTYRPSSHPLTASSPLSGLPEFSQLGWGHWFTLRDLHLATNWFSKDNIIGDGGYGVV
XP_018487317.1:1-460-[Raphanus-sativus]-AKI>           (93)   ------ERDIENGHNYS-----GSLEKHVP-------------------TS-ETTHSPLSGLQEGSHIGWGHWFTLRDLQLATNHFSKENIIGDGGYGVV
RID63431.1:1-451-[Brassica-rapa]-AKI>                  (91)   ------ERNIENGDN-------NNYEKHVP-------------------TSETTHSPHLSGLQEGSHIGWGHWFTLRDLQLATNHFSKENIIGDGGYGVV
XP_013749575.1:1-451-[Brassica-napus]-AKI>             (91)   ------ERNIENGDN-------NNYEKHVP-------------------TSETTHSPHLSGLQEGSHIGWGHWFTLRDLQLATNHFSKENIIGDGGYGVV
XP_009146013.1:1-450-[Brassica-rapa]-AKI>              (91)   ------ERDIENGDN--------NYEKHVP-------------------TSETTHSPHLSGLQEGSHIGWGHWFTLRDLQVATNHFSKENIIGDGGYGVV
VDC72349.1:1-450-[Brassica-rapa]-AKI>                  (91)   ------ERDIENGDN--------NYEKHVP-------------------TSETTHSPHLSGLQEGSHIGWGHWFTLRDLQVATNHFSKENIIGDGGYGVV
XP_013583744.1:1-449-[Brassica-oleracea-var.-olera...  (90)   ------ERDIENGDN--------NYEKHVP-------------------PSETTHSPHLSGLQEGSHIGWGHWFTLRDLQLATNHFSKENIIGDGGYGVV
KAF2548204.1:1-449-[Brassica-cretica]-AKI>             (90)   ------ERDIENGDN--------NYEKHVP-------------------TSETTHSPHLSGLQEGSHIGWGHWFTLRDLQLATNHFSKENIIGDGGYGVV
VDD46094.1:1-449-[Brassica-oleracea]-AKI>              (90)   ------ERDIENGDN--------NYEKHVP-------------------TSETTHSPHLSGLQEGSHIGWGHWFTLRDLQLATNHFSKENIIGDGGYGVV
KAF3587966.1:1-449-[Brassica-cretica]-AKI>             (90)   ------ERDIENGDN--------NYEKHVP-------------------PSETTHSPHLSGLQEGSHIGWGHWFTLRDLQLATNHFSKENIIGDGGYGVV
XP_013713104.1:1-449-[Brassica-napus]-AKI>             (90)   ------ERDIENGDN--------NYEKHVP-------------------PSETTHSPHLSGLQEGSHIGWGHWFTLRDLQLATNHFSKENIIGDGGYGVV
                                                              201                                                                                                300
NP_188368.2:1-467-[Arabidopsis-thaliana]-AKI>         (169)   YHGTLTNKTPVAVKKLLNNPGQADKDFRVEVEAIGHVRHKNLVRLLGYCVEGTHRMLVYEYMNNGNLEQWLHGDMIHKGHLTWEARIKVLVGTAKALAYL
```

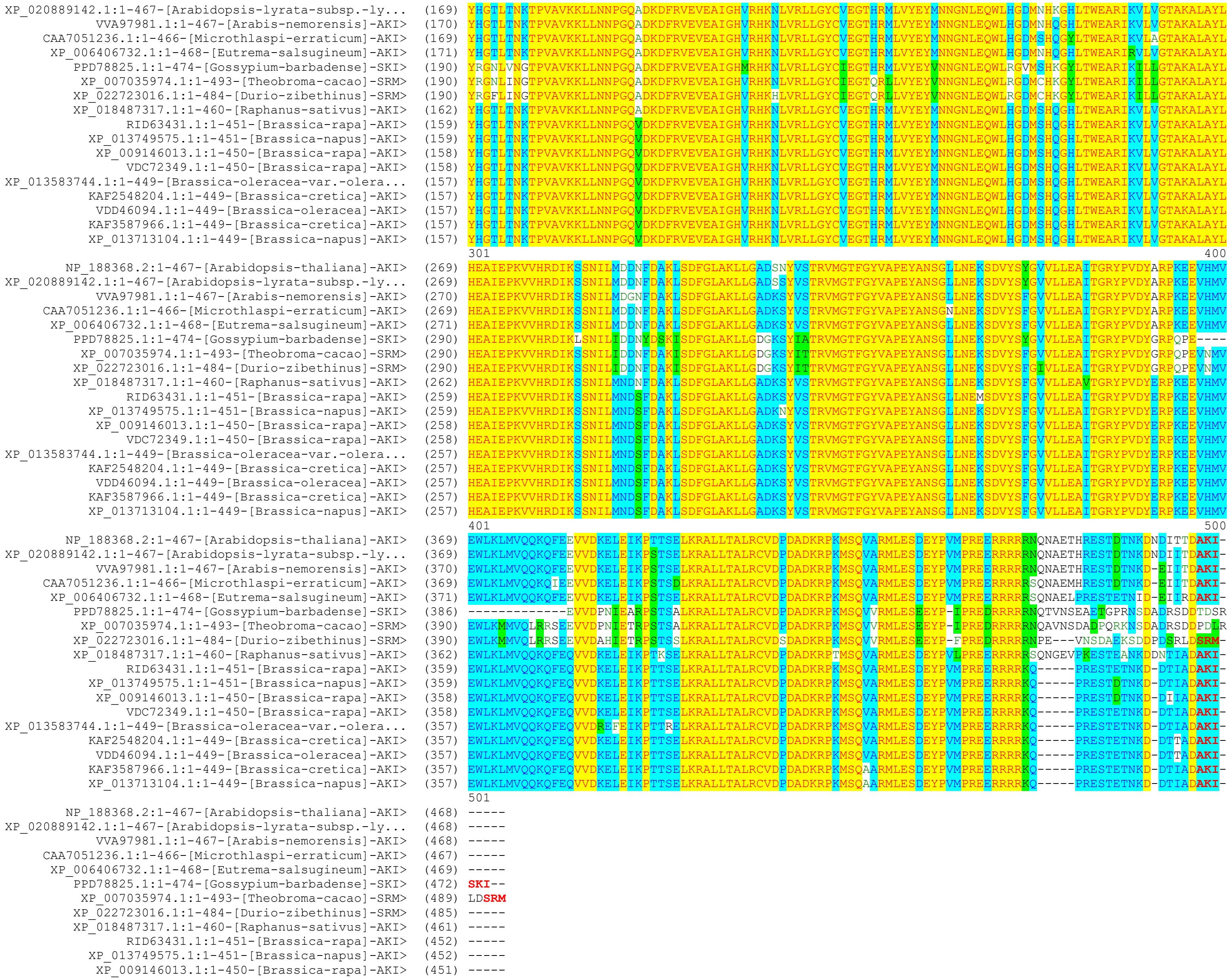

```
               VDC72349.1:1-450-[Brassica-rapa]-AKI>  (451) -----
XP_013583744.1:1-449-[Brassica-oleracea-var.-olera...  (450) -----
           KAF2548204.1:1-449-[Brassica-cretica]-AKI>  (450) -----
            VDD46094.1:1-449-[Brassica-oleracea]-AKI>  (450) -----
           KAF3587966.1:1-449-[Brassica-cretica]-AKI>  (450) -----
           XP_013713104.1:1-449-[Brassica-napus]-AKI>  (450) -----
```

# K18_ AT5G04870.1

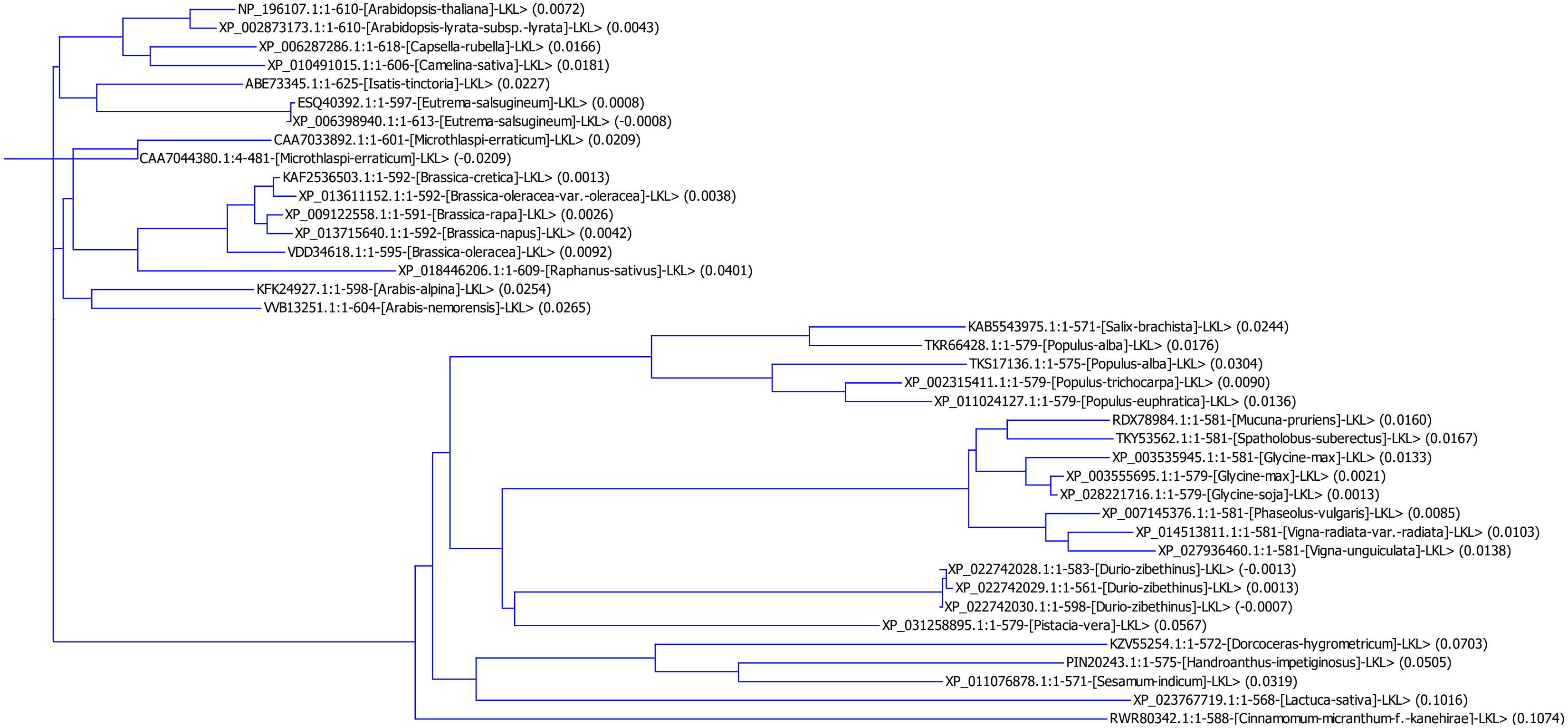


```
                                                                  1                                                                                                            110
          NP_196107.1:1-610-[Arabidopsis-thaliana]-LKL>      (1) MGNTCVGPS--RNGFLQSVSAAMWRPRDGDDSASMSNGDIASEA---VSGELRSR-----------LSDEVQNKPPEQVTMPKPGTDVETKDR---------------EI
 XP_002873173.1:1-610-[Arabidopsis-lyrata-subsp.-ly...      (1) MGNTCVGPS--RNGFLQSVSAAMWRPRDGDDSASMSNGDIASEA---VSGELRSP-----------LSDEVQNKPPEQVTMPKPGTNVETKDR---------------EI
       XP_006287286.1:1-618-[Capsella-rubella]-LKL>      (1) MGNTCVGPS--RNGFLQSVSAAMWRPRDGDDSASMSNGDIASEP---VSGELRSP-----------FSDDVQNKPPEQVTMPKSGTNIETKDREIR--------------
        XP_010491015.1:1-606-[Camelina-sativa]-LKL>      (1) MGNTCVGPS--RNGFLQSVSAAMWRPRDGDDSASVSNGDIASRP---VSGELRSP-----------LSDDVQNKPPEQVTMPKPGANIETKDR-----------------
          ABE73345.1:1-625-[Isatis-tinctoria]-LKL>      (1) MGNTCVGPS--RNGFLQSVSAAMWRPRDADDSVSQSNGDTASEA---VSGELRSP-----------SSDQVQNKPPEQLTMPKPGTNIEIKAKSDVEIQPEIKLETQEVT
       ESQ40392.1:1-597-[Eutrema-salsugineum]-LKL>      (1) MGNTCVGPS--RNGFLQSVSAAMWRPRDADDSVSQRNGDTASEA---VSGELRSP-----------LSNQVMNKPPEQLTMPKPGADVETKAKSDIQIQ-----------
    XP_006398940.1:1-613-[Eutrema-salsugineum]-LKL>      (1) MGNTCVGPS--RNGFLQSVSAAMWRPRDADDSVSQRNGDTASEA---VSGELRSP-----------LSNQVMNKPPEQLTMPKPGADVETKAKSDIQIQ-----------
   CAA7033892.1:1-601-[Microthlaspi-erraticum]-LKL>      (1) MGNTCVGPS--GNGFFHSVSAAMWRPRDVDDSVSQSNGDTASEA---VSGELRSP-----------LPDHVQNKPPELLTMPKPGTNSETK-------------------
   CAA7044380.1:4-481-[Microthlaspi-erraticum]-LKL>      (1) --------------------------------------------------------------------------------------------------------------
         KAF2536503.1:1-592-[Brassica-cretica]-LKL>      (1) MGNTCVGPS--RNGFLQSVSAAMWRPRDGDDSVSQSNGGTASEAA--VSGELRSPSS---------SDHQVLNKPPEHLTMPKPGETKAKS-------------------
 XP_013611152.1:1-592-[Brassica-oleracea-var.-olera...      (1) MGNTCVGPS--RNGFLQSVSAAMWRPRDGDDSVSQSNGGTASEAA--VSGELRSPSS---------SDHQVLNKPPEHLTMPKPGETKAKS-------------------
         XP_009122558.1:1-591-[Brassica-rapa]-LKL>      (1) MGNTCVGPS--RNGFLQSVSAAMWRPRDGDDSVSQSNGDTASEAA--VSGELRSPSS---------PDQQVLNKPPEHLTMPKPVETKAKS-------------------
        XP_013715640.1:1-592-[Brassica-napus]-LKL>      (1) MGNTCVGPS--RNGFLQSVSAAMWRPRDGDDSVSQSNGDTTSEAA--VSGELRSPSS---------PDHQVLNKPPEHLTMPKPVETKAKS-------------------
          VDD34618.1:1-595-[Brassica-oleracea]-LKL>      (1) MGNTCVGPS--RNGFLQSVSAAMWRPRDGDDSVSQSNGDTASEAA--VSGELRSPSS---------SDHQVLNKPPEHLTMPKPAETKAKS-------------------
     XP_018446206.1:1-609-[Raphanus-sativus]-LKL>      (1) MGNTCVGPSR-NGGFLQSVSAAMWRPRDGDDSVSQSNGGDTASEA--VSGELRSPSSS------SSSHQVVMNKPPEQITMPKPPVETNNIET-----------------
            KFK24927.1:1-598-[Arabis-alpina]-LKL>      (1) MGNTCVGPSR-DGGFLQSVSAAMWRPRDADDSVSQSNGDTASEA---VSGELRSL-----------SSDQVQNKPPEHFTMPKPVTNIETK-------------------
        VVB13251.1:1-604-[Arabis-nemorensis]-LKL>      (1) MGNTCVGPS--RNGFLQSVSAAMWRPRDVDDSVSQSNGDNASEA---VSGELRSRSQ---------LSDQVLNKPPEHLTMPKPIETKAKSDIEIR--------------
        KAB5543975.1:1-571-[Salix-brachista]-LKL>      (1) MGNTCVGPSISKNVFFQSVSAAMWRTRSPDDSMSQTNGEPVHEHEA-ASRESELP-------------VPVQSKPPEQMTIPKP--------------------------
              TKR66428.1:1-579-[Populus-alba]-LKL>      (1) MGNTCVGPSISKNGFFQSVSAAMWRTRSPDDSMSRTNGESVHELEA-VSRESEPP-------------LPVQSKPPEQMTIPKP--------------------------
              TKS17136.1:1-575-[Populus-alba]-LKL>      (1) MGNTCVGPSISRNGFFQSVSAAMWRNQSPDDSMSQTNGEPVHEHEA-ASRELESP-------------LPVQSKPPEQMTMPKP--------------------------
    XP_002315411.1:1-579-[Populus-trichocarpa]-LKL>      (1) MGNTCVGPSISRNGFFQSVSAAMWRNRSPDDSMSQTNGESVHEHEA-ASRELESP-------------LPVQSKPPEQMTIPKP--------------------------
```

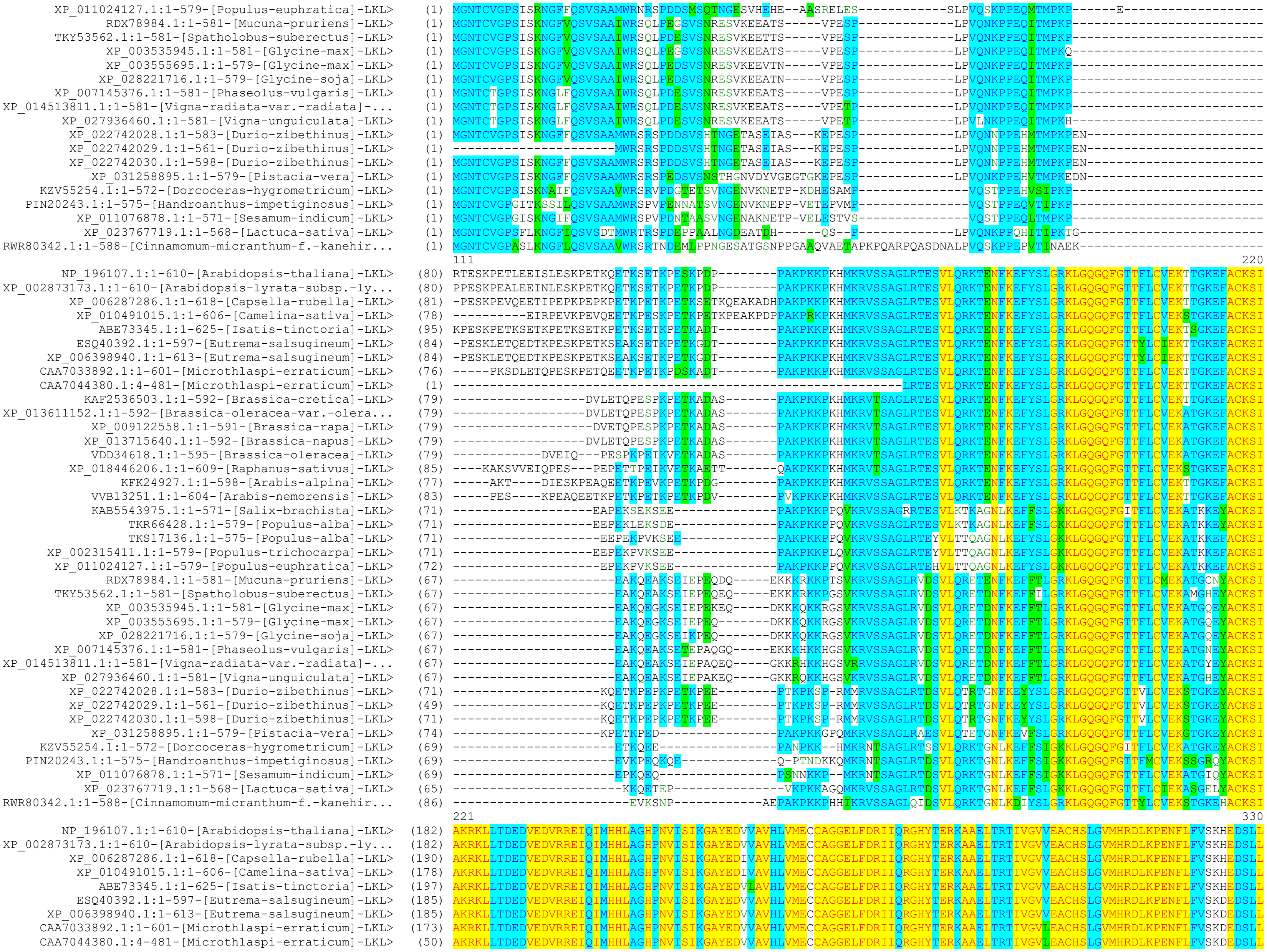

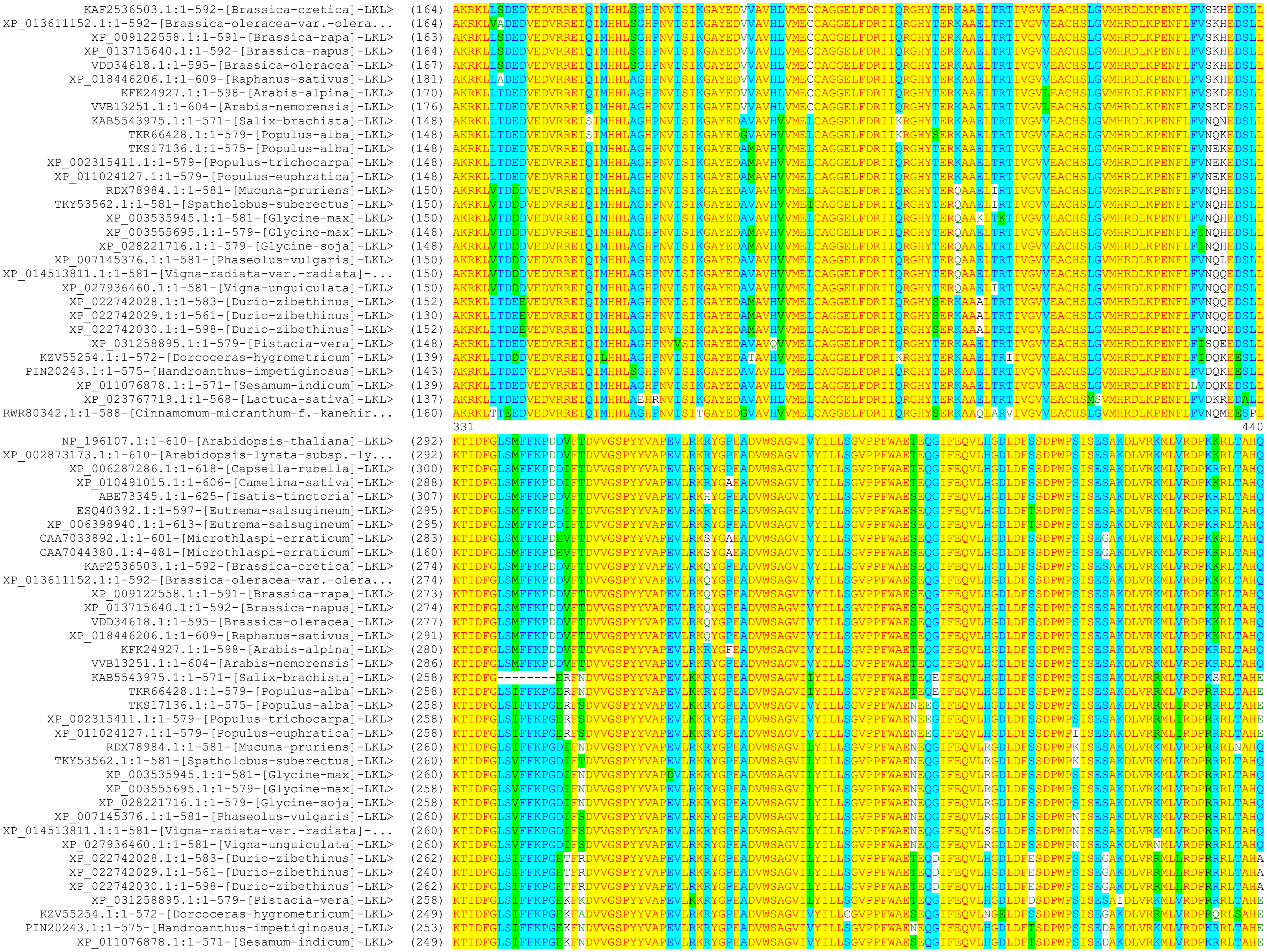

KAF2536503.1:1-592-[Brassica-cretica]-LKL> (164) AKRKLLSDEDVEDVRREIQIMHHLSGHPNVISIKGAYEDVVAVHLVMECCAGGELFDRIIQRGHYTERKAAELTRTIVGVVEACHSLGVMHRDLKPENFLFVSKHEDSLL
XP_013611152.1:1-592-[Brassica-oleracea-var.-olera... (164) AKRKLVADEDVEDVRREIQIMHHLSGHPNVISIKGAYEDVVAVHLVMECCAGGELFDRIIQRGHYTERKAAELTRTIVGVVEACHSLGVMHRDLKPENFLFVSKHEDSLL
XP_009122558.1:1-591-[Brassica-rapa]-LKL> (163) AKRKLLSDEDVEDVRREIQIMHHLSGHPNVISIKGAYEDVVAVHLVMECCAGGELFDRIIQRGHYTERKAAELTRTIVGVVEACHSLGVMHRDLKPENFLFVSKHEDSLL
XP_013715640.1:1-592-[Brassica-napus]-LKL> (164) AKRKLLSDEDVEDVRREIQIMHHLSGHPNVISIKGAYEDVVAVHLVMECCAGGELFDRIIQRGHYTERKAAELTRTIVGVVEACHSLGVMHRDLKPENFLFVSKHEDSLL
VDD34618.1:1-595-[Brassica-oleracea]-LKL> (167) AKRKLLSDEDVEDVRREIQIMHHLSGHPNVISIKGAYEDVVAVHLVMECCAGGELFDRIIQRGHYTERKAAELTRTIVGVVEACHSLGVMHRDLKPENFLFVSKHEDSLL
XP_018446206.1:1-609-[Raphanus-sativus]-LKL> (181) AKRKLLADEDVEDVRREIQIMHHLAGHPNVISIKGAYEDVVAVHLVMECCAGGELFDRIIQRGHYTERKAAELTRTIVGVVEACHSLGVMHRDLKPENFLFVSKHEDSLL
KFK24927.1:1-598-[Arabis-alpina]-LKL> (170) AKRKLLTDEDVEDVRREIQIMHHLAGHPNVISIKGAYEDVVAVHLVMECCAGGELFDRIIQRGHYTERKAAELTRTIVGVLEACHSLGVMHRDLKPENFLFVSKDEDSLL
VVB13251.1:1-604-[Arabis-nemorensis]-LKL> (176) AKRKLLTDEDVEDVRREIQIMHHLAGHPNVISIKGAYEDVVAVHLVMECCAGGELFDRIIQRGHYTERKAAELTRTIVGVLEACHSLGVMHRDLKPENFLFVSKDEDSLL
KAB5543975.1:1-571-[Salix-brachista]-LKL> (148) AKRKLLTDEDVEDVRREISIMHHLAGHPNVISIKGAYEDAVAVHVVMELCAGGELFDRIIKRGHYTERKAAELTRTIVGVVEACHSLGVMHRDLKPENFLFVNQNEDSLL
TKR66428.1:1-579-[Populus-alba]-LKL> (148) AKRKLLTDEDVEDVRREISIMHHLAGHPNVISIKGAYEDGVAVHVVMELCAGGELFDRIIKRGHYSERKAAELTRTIVGVVEACHSLGVMHRDLKPENFLFVNQKEDSLL
TKS17136.1:1-575-[Populus-alba]-LKL> (148) AKRKLLTDEDVEDVRREIQIMHHLAGHPNVISIKGAYEDAMAVHVVMELCAGGELFDRIIQRGHYTERKAAELTRTIVGVVEACHSLGVMHRDLKPENFLFVNEKEDSLL
XP_002315411.1:1-579-[Populus-trichocarpa]-LKL> (148) AKRKLLTDEDVEDVRREIQIMHHLAGHPNVISIKGAYEDAMAVHVVMELCAGGELFDRIIQRGHYTERKAAELTRTIVGVVEACHSLGVMHRDLKPENFLFVNEKEDSLL
XP_011024127.1:1-579-[Populus-euphratica]-LKL> (148) AKRKLLTDEDVEDVRREIQIMHHLAGHPNVISIKGAYEDAMAVHVVMELCAGGELFDRIIQRGHYTERKAAELTRTIVGVVEACHSLGVMHRDLKPENFLFVNEKEDSLL
RDX78984.1:1-581-[Mucuna-pruriens]-LKL> (150) AKRKLVTDDDVEDVRREIQIMHHLAGHPNVISIKGAYEDAVAVHVVMELCAGGELFDRIIQRGHYTERQAAELIRTIVGVVEACHSLGVMHRDLKPENFLFVNQHEDSLL
TKY53562.1:1-581-[Spatholobus-suberectus]-LKL> (150) AKRKLVTDDDVEDVRREIQIMHHLAGHPNVISIKGAYEDAVAVHVVMEICAGGELFDRIIQRGHYTERQAAELIRTIVGVVEACHSLGVMHRDLKPENFLFVNQHEDSLL
XP_003535945.1:1-581-[Glycine-max]-LKL> (150) AKRKLVTDDDVEDVRREIQIMHHLAGHPNVISIKGAYEDAVAVHVVMELCAGGELFDRIIQRGHYTERQAAKLTKTIVGVVEACHSLGVMHRDLKPENFLFVNQHEDSLL
XP_003555695.1:1-579-[Glycine-max]-LKL> (148) AKRKLVTDDDVEDVRREIQIMHHLAGHPNVISIKGAYEDAMAVHVVMELCAGGELFDRIIQRGHYTERQAAELTRTIVGVVEACHSLGVMHRDLKPENFLFINQHEDSLL
XP_028221716.1:1-579-[Glycine-soja]-LKL> (148) AKRKLVTDDDVEDVRREIQIMHHLAGHPNVISIKGAYEDAMAVHVVMELCAGGELFDRIIQRGHYTERQAAELTRTIVGVVEACHSLGVMHRDLKPENFLFINQHEDSLL
XP_007145376.1:1-581-[Phaseolus-vulgaris]-LKL> (150) AKRKLVTDDDVEDVRREIQIMHHLAGHPNVISIKGAYEDAVAVHVVMELCAGGELFDRIIQRGHYTERQAAELTRTIVGVVEACHSLGVMHRDLKPENFLFVNQLEDSLL
XP_014513811.1:1-581-[Vigna-radiata-var.-radiata]-... (150) AKRKLVTDDDVEDVRREIQIMHHLAGHPNVISIKGAYEDAVAVHVVMELCAGGELFDRIIQRGHYTERQAAELTRTIVGVVEACHSLGVMHRDLKPENFLFVNQQEDSLL
XP_027936460.1:1-581-[Vigna-unguiculata]-LKL> (150) AKRKLVTDDDVEDVRREIQIMHHLAGHPNVISIKGAYEDAVAVHVVMELCAGGELFDRIIQRGHYTERQAAELIRTIVGVVEACHSLGVMHRDLKPENFLFVNQQEDSLL
XP_022742028.1:1-583-[Durio-zibethinus]-LKL> (152) AKRKLLTDEEVEDVRREIQIMHHLAGHPNVISIKGAYEDAMAVHVVMELCAGGELFDRIIQRGHYSERKAAALTRTIVGVVEACHSLGVMHRDLKPENFLFVNQQEDSLL
XP_022742029.1:1-561-[Durio-zibethinus]-LKL> (130) AKRKLLTDEEVEDVRREIQIMHHLAGHPNVISIKGAYEDAMAVHVVMELCAGGELFDRIIQRGHYSERKAAALTRTIVGVVEACHSLGVMHRDLKPENFLFVNQQEDSLL
XP_022742030.1:1-598-[Durio-zibethinus]-LKL> (152) AKRKLLTDEEVEDVRREIQIMHHLAGHPNVISIKGAYEDAMAVHVVMELCAGGELFDRIIQRGHYSERKAAALTRTIVGVVEACHSLGVMHRDLKPENFLFVNQQEDSLL
XP_031258895.1:1-579-[Pistacia-vera]-LKL> (148) AKRKLLTDEDVEDVRREIQIMHHLAGHPNVVSIKGAYEDAVAVQVVMELCAGGELFDRIIQRGHYTERKAAELTRTIVGVVEACHSLGVMHRDLKPENFLFISQEEDSLL
KZV55254.1:1-572-[Dorcoceras-hygrometricum]-LKL> (139) AKRKLLTDDDVEDVRREIQILHHLAGHPNVISIKGAYEDATAVHVVMELCAGGELFDRIIKRGHYTERKAAELTRIIVGVVEACHSLGVMHRDLKPENFLFIDQKEESLL
PIN20243.1:1-575-[Handroanthus-impetiginosus]-LKL> (143) AKRKLLTDEDVEDVRREIQIMHHLSGHPNVISIKGAYEDAVAVHLVMELCAGGELFDRIIQRGHYTERKAAELTRTIVGVVEACHSLGVMHRDLKPENFLFVDQKEESLL
XP_011076878.1:1-571-[Sesamum-indicum]-LKL> (139) AKRKLLTDEDVEDVRREIQIMHHLAGHPNVISIKGAYEDAVAVHLVMELCAGGELFDRIIQRGHYTERKAAELTRTIVGVVEACHSLGVMHRDLKPENFLLVDQKEDSLL
XP_023767719.1:1-568-[Lactuca-sativa]-LKL> (137) AKRKLLTDEDVEDVRREIQIMHHLAEHRNVISIKGAYEDAVAVHLVMELCAGGELFDRIIQRGHYTERKAAELTRTIVGVVEACHSMSVMHRDLKPENFLFVDKREDALL
RWR80342.1:1-588-[Cinnamomum-micranthum-f.-kanehir... (160) AKRKLTTEEDVEDVRREIQIMHHLAGHPNVISITGAYEDGVAVHVVMELCAGGELFDRIIQRGHYSERKAAQLARVIVGVVEACHSLGVMHRDLKPENFLFVNQMEESPL
331 440
NP_196107.1:1-610-[Arabidopsis-thaliana]-LKL> (292) KTIDFGLSMFFKPDDVFTDVVGSPYYVAPEVLRKRYGPEADVWSAGVIVYILLSGVPPFWAETEQGIFEQVLHGDLDFSSDPWPSISESAKDLVRKMLVRDPKKRLTAHQ
XP_002873173.1:1-610-[Arabidopsis-lyrata-subsp.-ly... (292) KTIDFGLSMFFKPDDVFTDVVGSPYYVAPEVLRKRYGPEADVWSAGVIVYILLSGVPPFWAETEQGIFEQVLHGDLDFSSDPWPSISESAKDLVRKMLVRDPKKRLTAHQ
XP_006287286.1:1-618-[Capsella-rubella]-LKL> (300) KTIDFGLSMFFKPDDIFTDVVGSPYYVAPEVLRKRYGPEADVWSAGVIVYILLSGVPPFWAETEQGIFEQVLHGDLDFSSDPWPSISESAKDLVRKMLVRDPKRRLTAHQ
XP_010491015.1:1-606-[Camelina-sativa]-LKL> (288) KTIDFGLSMFFKPDDIFTDVVGSPYYVAPEVLRKRYGAEADVWSAGVIVYILLSGVPPFWAETEQGIFEQVLHGDLDFSSDPWPSISESAKDLVRKMLVRDPKKRLTAHQ
ABE73345.1:1-625-[Isatis-tinctoria]-LKL> (307) KTIDFGLSMFFKPDDVFTDVVGSPYYVAPEVLRKHYGPEADVWSAGVIVYILLSGVPPFWAESEQGIFEQVLHGDLDFSSDPWPSISESAKDLVRKMLVRDPKRRLTAHQ
ESQ40392.1:1-597-[Eutrema-salsugineum]-LKL> (295) KTIDFGLSMFFKPDDIFTDVVGSPYYVAPEVLRKRYGPEADVWSAGVIVYILLSGVPPFWAESEQGIFEQVLHGDLDFTSDPWPSISESAKDLVRKMLVRDPKRRLTAHQ
XP_006398940.1:1-613-[Eutrema-salsugineum]-LKL> (295) KTIDFGLSMFFKPDDIFTDVVGSPYYVAPEVLRKRYGPEADVWSAGVIVYILLSGVPPFWAESEQGIFEQVLHGDLDFTSDPWPSISESAKDLVRKMLVRDPKRRLTAHQ
CAA7033892.1:1-601-[Microthlaspi-erraticum]-LKL> (283) KTIDFGLSMFFKPDEVFTDVVGSPYYVAPEVLRKSYGAEADVWSAGVIVYILLSGVPPFWAESEQGIFEQVLHGDLDFSSDPWPSISEGAKDLVRKMLVRDPKKRLTAHQ
CAA7044380.1:4-481-[Microthlaspi-erraticum]-LKL> (160) KTIDFGLSMFFKPDEVFTDVVGSPYYVAPEVLRKSYGAEADVWSAGVIVYILLSGVPPFWAESEQGIFEQVLHGDLDFSSDPWPSISEGAKDLVRKMLVRDPKKRLTAHQ
KAF2536503.1:1-592-[Brassica-cretica]-LKL> (274) KTIDFGLSMFFKPDDVFTDVVGSPYYVAPEVLRKQYGPEADVWSAGVIVYILLSGVPPFWAESEQGIFEQVLHGDLDFSSDPWPSISESAKDLVRKMLVRDPKKRLTAHQ
XP_013611152.1:1-592-[Brassica-oleracea-var.-olera... (274) KTIDFGLSMFFKPDDVFTDVVGSPYYVAPEVLRKQYGPEADVWSAGVIVYILLSGVPPFWAESEQGIFEQVLHGDLDFSSDPWPSISESAKDLVRKMLVRDPKKRLTAHQ
XP_009122558.1:1-591-[Brassica-rapa]-LKL> (273) KTIDFGLSMFFKPDDVFTDVVGSPYYVAPEVLRKQYGPEADVWSAGVIVYILLSGVPPFWAESEQGIFEQVLHGDLDFSSDPWPSISESAKDLVRKMLVRDPKKRLTAHQ
XP_013715640.1:1-592-[Brassica-napus]-LKL> (274) KTIDFGLSMFFKPDDVFTDVVGSPYYVAPEVLRKQYGPEADVWSAGVIVYILLSGVPPFWAESEQGIFEQVLHGDLDFSSDPWPSISESAKDLVRKMLVRDPKKRLTAHQ
VDD34618.1:1-595-[Brassica-oleracea]-LKL> (277) KTIDFGLSMFFKPDDVFTDVVGSPYYVAPEVLRKQYGPEADVWSAGVIVYILLSGVPPFWAESEQGIFEQVLHGDLDFSSDPWPSISESAKDLVRKMLVRDPKKRLTAHQ
XP_018446206.1:1-609-[Raphanus-sativus]-LKL> (291) KTIDFGLSMFFKPDDVFTDVVGSPYYVAPEVLRKQYGPEADVWSAGVIVYILLSGVPPFWAESEQGIFEQVLHGDLDFSSDPWPSISESAKDLVRKMLVRDPKKRLTAHQ
KFK24927.1:1-598-[Arabis-alpina]-LKL> (280) KTIDFGLSMFFKPDDVFTDVVGSPYYVAPEVLRKRYGFEADVWSAGVIVYILLSGVPPFWAETEQGIFEQVLHGDLDFSSDPWPSISESAKDLVRKMLVRDPKRRLTAHQ
VVB13251.1:1-604-[Arabis-nemorensis]-LKL> (286) KTIDFGLSMFFKPDDVFTDVVGSPYYVAPEVLRKRYGPEADVWSAGVIVYILLSGVPPFWAETEQGIFEQVLHGDLDFSSDPWPSISESAKDLVRKMLVRDPKRRLTAHQ
KAB5543975.1:1-571-[Salix-brachista]-LKL> (258) KTIDFG--------ERFNDVVGSPYYVAPEVLKKRYGPEADVWSAGVIIYILLSGVPPFWAETEQEIFEQVLHGDLDFSSDPWPSISESAKDLVRRMLVRDPKSRLTAHE
TKR66428.1:1-579-[Populus-alba]-LKL> (258) KTIDFGLSIFFKPGERFNDVVGSPYYVAPEVLKKRYGPEADVWSAGVIIYILLSGVPPFWAETEQEIFEQVLHGDLDFSSDPWPSISESAKDLVRRMLVRDPRRRLTAHE
TKS17136.1:1-575-[Populus-alba]-LKL> (258) KTIDFGLSIFFKPGERFSDVVGSPYYVAPEVLKKRYGPEADVWSAGVIVYILLSGVPPFWAENEEGIFEQVLHGDLDFSSDPWPSISESAKDLVRRMLIRDPRRRLTAHE
XP_002315411.1:1-579-[Populus-trichocarpa]-LKL> (258) KTIDFGLSIFFKPGERFSDVVGSPYYVAPEVLKKRYGPEADVWSAGVIVYILLSGVPPFWAENEEGIFEQVLHGDLDFSSDPWPSISESAKDLVRRMLIRDPRRRLTAHE
XP_011024127.1:1-579-[Populus-euphratica]-LKL> (258) KTIDFGLSIFFKPGERFSDVVGSPYYVAPEVLKKRYGPEADVWSAGVIVYILLSGVPPFWAENEEGIFEQVLHGDLDFSSDPWPIISESAKDLVRRMLIRDPRRRLTAHE
RDX78984.1:1-581-[Mucuna-pruriens]-LKL> (260) KTIDFGLSIFFKPGDIFNDVVGSPYYVAPEVLRKRYGPEADVWSAGVILYILLSGVPPFWAENEQGIFEQVLRGDLDFSSDPWPKISESAKDLVRKMLVRDPRRRLNAHQ
TKY53562.1:1-581-[Spatholobus-suberectus]-LKL> (260) KTIDFGLSVFFKPGDIFTDVVGSPYYVAPEVLRKRYGPEADVWSAGVILYILLSGVPPFWAENEQGIFEQVLRGDLDFSSDPWPKISESAKDLVRKMLVRDPRRRLTAHQ
XP_003535945.1:1-581-[Glycine-max]-LKL> (260) KTIDFGLSVFFKPGDIFNDVVGSPYYVAPDVLRKRYGPEADVWSAGVILYILLSGVPPFWAENEQGIFEQVLRGDLDFSSDPWPSISESAKDLVRKMLVRDPRRRLTAHQ
XP_003555695.1:1-579-[Glycine-max]-LKL> (258) KTIDFGLSVFFKPGDIFNDVVGSPYYVAPEVLRKRYGPEADVWSAGVILYILLSGVPPFWAENEQGIFEQVLRGDLDFSSDPWPSISESAKDLVRKMLVRDPRRRLTAHQ
XP_028221716.1:1-579-[Glycine-soja]-LKL> (258) KTIDFGLSVFFKPGDIFNDVVGSPYYVAPEVLRKRYGPEADVWSAGVILYILLSGVPPFWAENEQGIFEQVLRGDLDFSSDPWPSISESAKDLVRKMLVRDPRRRLTAHQ
XP_007145376.1:1-581-[Phaseolus-vulgaris]-LKL> (260) KTIDFGLSVFFKPGDIFSDVVGSPYYVAPEVLRKRYGPEADVWSAGVILYILLSGVPPFWAENEQGIFEQVLRGDLDFSSDPWPNISESAKDLVRKMLVRDPRRRLTAHQ
XP_014513811.1:1-581-[Vigna-radiata-var.-radiata]-... (260) KTIDFGLSVFFKPGDIFSDVVGSPYYVAPEVLRKRYGPEADVWSAGVILYILLSGVPPFWAENEQGIFEQVLSGDLDFSSDPWPNISESAKDLVRKMLVRDPRRRLTAHQ
XP_027936460.1:1-581-[Vigna-unguiculata]-LKL> (260) KTIDFGLSVFFKPGDIFSDVVGSPYYVAPEVLRKRYGPEADVWSAGVILYILLSGVPPFWAENEQGIFEQVLRGDLDFSSDPWPNISESAKDLVRNMLVRDPRRRLTAHQ
XP_022742028.1:1-583-[Durio-zibethinus]-LKL> (262) KTIDFGLSIFFKPGETFRDVVGSPYYVAPEVLRKRYGPEADVWSAGVILYILLSGVPPFWAETEQDIFEQVLHGDLDFESDPWPSISEGAKDLVRRMLLRDPRRRLTAHA
XP_022742029.1:1-561-[Durio-zibethinus]-LKL> (240) KTIDFGLSIFFKPGETFRDVVGSPYYVAPEVLRKRYGPEADVWSAGVILYILLSGVPPFWAETEQDIFEQVLHGDLDFESDPWPSISEGAKDLVRRMLLRDPRRRLTAHA
XP_022742030.1:1-598-[Durio-zibethinus]-LKL> (262) KTIDFGLSIFFKPGETFRDVVGSPYYVAPEVLRKRYGPEADVWSAGVILYILLSGVPPFWAETEQDIFEQVLHGDLDFESDPWPSISEGAKDLVRRMLLRDPRRRLTAHA
XP_031258895.1:1-579-[Pistacia-vera]-LKL> (258) KTIDFGLSIFFKPGEKFKDVVGSPYYVAPEVLRKRYGPEADVWSAGVILYILLSGVPPFWAETEQGIFEQVLHGDLDFDSDPWPSISESAIDLVRKMLVRDPRRRLTAHE
KZV55254.1:1-572-[Dorcoceras-hygrometricum]-LKL> (249) KTIDFGLSIFFKPGEKFADVVGSPYYVAPEVLRKRYGPEADVWSAGVIVYILLCGVPPFWAESEQGIFEQVLNGELDFSSDPWPSISEGAKDLVRRMLVRDPRQRLSAHE
PIN20243.1:1-575-[Handroanthus-impetiginosus]-LKL> (253) KTIDFGLSIFFKPGEKFNDVVGSPYYVAPEVLRKRYGPEADVWSAGVIVYILLSGVPPFWAENEQGIFEQVLHGDLDFTSDPWPSISEDAKDLVRKMLVRDPKRRLTAHE
XP_011076878.1:1-571-[Sesamum-indicum]-LKL> (249) KTIDFGLSIFFKPGEKFNDVVGSPYYVAPEVLRKRYGPEADVWSAGVIVYILLSGVPPFWAESEQGIFEQVLHGDLDFTSDPWPSISEDAKDLVRKMLVRDPKRRLTAHE

```
         XP_023767719.1:1-568-[Lactuca-sativa]-LKL>  (247) KTIDFGLSVFFKPGESFHDVVGSPYYVAPEILRKNYGPEADVWSAGVIVYILLSGVPPFWAETEQGIFEQVLHGDLDFTSDPWPCISDGAKDLVRRMLIRDPKRRLTAHE
 RWR80342.1:1-588-[Cinnamomum-micranthum-f.-kanehir...  (270) KTIDFGLSMFFKPGDIFTDVVGSPYYVAPEVLRKRYGPECDVWSAGVIIYILLCGVPPFWAETEQGIFEQVLHGDLDFLSDPWPSISESAKDLVRKMLVRDPKNRLTAHQ
                                                           441                                                                                                         550
       NP_196107.1:1-610-[Arabidopsis-thaliana]-LKL>  (402) VLCHPWVQVDGVAPDKPLDSAVLSRMKQFSAMNKFKKMALRVIAESLSEEEIAGLKEMFNMIDADKSGQITFEELKAGLKRVGANLKESEILDLMQA-------------
 XP_002873173.1:1-610-[Arabidopsis-lyrata-subsp.-ly...  (402) VLCHPWVQVDGVAPDKPLDSAVLSRMKQFSAMNKFKKMALRVIAESLSEEEIAGLKEMFNMIDADKSGQITFEELKAGLKRVGANLKESEILDLMQA-------------
       XP_006287286.1:1-618-[Capsella-rubella]-LKL>  (410) VLCHPWVQVDGVAPDKPLDSAVLSRMKQFSAMNKFKKMALRVIAESLSEEEIAGLKEMFNMIDADKSGQITFEELKAGLKRVGANLKESEILDLMQA-------------
        XP_010491015.1:1-606-[Camelina-sativa]-LKL>  (398) VLCHPWVQVDGVAPDKPLDSAVLSRMKQFSAMNKFKKMALRVIAESLSEEEIAGLKEMFNMIDADKSGQITFEELKAGLKRVGANLNESEILDLMQA-------------
          ABE73345.1:1-625-[Isatis-tinctoria]-LKL>  (417) VLCHPWVQIDGVAPDKPLDSAVLSRMKQFSAMNKFKKMALRVIAESLSEEEIAGLKEMFNMIDADKSGQITFEELKAGLKRVGANLKESEILDLMQA-------------
        ESQ40392.1:1-597-[Eutrema-salsugineum]-LKL>  (405) VLCHPWVQVDGVAPDKPLDSAVLSRMKQFSAMNKFKKMALRVIAESLSEEEIAGLKEMFNMIDADKSGQITFEELKVGLKR-----------------------------
     XP_006398940.1:1-613-[Eutrema-salsugineum]-LKL>  (405) VLCHPWVQVDGVAPDKPLDSAVLSRMKQFSAMNKFKKMALRVIAESLSEEEIAGLKEMFNMIDADKSGQITFEELKVGLKRVGANLKESEILDLMQA-------------
    CAA7033892.1:1-601-[Microthlaspi-erraticum]-LKL>  (393) VLCHPWVQVDGVAPDKPLDSAVLSRMKQFSAMNKFKKMALRVIAESLSEEEIAGLKEMFNMIDADKSGQITFEELKAGLKRVGANLKESEILDLMQA-------------
    CAA7044380.1:4-481-[Microthlaspi-erraticum]-LKL>  (270) VLCHPWVQVDGVAPDKPLDSAVLSRMKQFSAMNKFKKMALRVIAESLSEEEIAGLKEMFNMIDADKSGQITFEELKAGLKRVGANLKESEILDLMQA-------------
          KAF2536503.1:1-592-[Brassica-cretica]-LKL>  (384) VLCHPWVQVDGVAPDKPLDSAVLSRMKQFSAMNKFKKMALRVIAESLSEEEIAGLKEMFNMIDADQSGQITFEELKAGLKRVGANLKESEILDLMQA-------------
 XP_013611152.1:1-592-[Brassica-oleracea-var.-olera...  (384) VLCHPWVQVDGVAPDKPLDSAVLSRMKQFSAMNKFKKMALRVIAESLSEEEIAGLKEMFNMIDADQSGQITFEELKAGLKRVGANLKESEILDLMQA-------------
          XP_009122558.1:1-591-[Brassica-rapa]-LKL>  (383) VLCHPWVQVDGVAPDKPLDSAVLSRMKQFSAMNKFKKMALRVIAESLSEEEIAGLKEMFNMIDADQSGQITFEELKAGLKRVGANLKESEILDLMQA-------------
         XP_013715640.1:1-592-[Brassica-napus]-LKL>  (384) VLCHPWVQVDGVAPDKPLDSAVLSRMKQFSAMNKFKKMALRVIAESLSEEEIAGLKEMFNMIDADQSGQITFEELKAGLKRVGANLKESEILDLMQA-------------
          VDD34618.1:1-595-[Brassica-oleracea]-LKL>  (387) VLCHPWVQVDGVAPDKPLDSAVLSRMKQFSAMNKFKKMALRVIAESLSEEEIAGLKEMFNMIDADQSGQITFEELKAGLKRVGANLKESEILDLMQA-------------
      XP_018446206.1:1-609-[Raphanus-sativus]-LKL>  (401) VLCHPWVQVDGVAPDKPLDSAVLSRMKQFSAMNKFKKMALRVIAESLSEEEIAGLKEMFNMIDADQSGQITFEELKAGLKRVGANLKESEILDLMQA-------------
              KFK24927.1:1-598-[Arabis-alpina]-LKL>  (390) VLCHPWVQVDGVAPDKPLDSAVLSRMKQFSAMNKLKKMALRVIAESLSEEEIAGLKEMFNMIDADNSGQITFEELKAGLKRVGANLKESEILDLMQA-------------
          VVB13251.1:1-604-[Arabis-nemorensis]-LKL>  (396) VLCHPWVQVDGVAPDKPLDSAVLSRMKQFSAMNKLKKMALRVIAESLSEEEIAGLKEMFNMIDADNSGQITFEELKAGLKRVGANLKESEILDLMQA-------------
          KAB5543975.1:1-571-[Salix-brachista]-LKL>  (360) VLCHPWVQEDGVAPDKPLDSAVLSRLKQFSAMNKLKKMALRIIAETLSEEEIAGLKEMFKMIDSDGSGQITFEELKAGLKRAGANLKESEIYDLMHA-------------
               TKR66428.1:1-579-[Populus-alba]-LKL>  (368) VLCHPWVQEDGVAPDKPLDSAVLSRLKQFSAMNKLKKMALRIIAETLSEEEIAGLKEMFQMIDSDGSGQITFEELKAGLKRVGANLKESEIYDLMQA-------------
               TKS17136.1:1-575-[Populus-alba]-LKL>  (368) VLCHPWVQEDGVAPDKPLDSAVLSRLKQFSAMNKFKKMALRVIAETLSEEEIAGLKEMFKMIDTDGSGHITFEELKAGLKRFGANLKESEIYDLMHA-------------
     XP_002315411.1:1-579-[Populus-trichocarpa]-LKL>  (368) VLCHPWVQEDGVAPDKPLDSAVLSRLKQFSAMNKFKKMALRVIAETLSEEEIAGLKEMFKMIDTDGSGHITFEELKAGLKRFGANLKESEIYDLMQA-------------
      XP_011024127.1:1-579-[Populus-euphratica]-LKL>  (368) VLCHPWVQEDGVAPDKPLDSAVLSRLKQFSAMNKFKKMALRVIAETLSEEEIAGLKEMFKMIDTDGSGHITFEELKAGLKRFGANLKESEIYDLMQA-------------
           RDX78984.1:1-581-[Mucuna-pruriens]-LKL>  (370) VLCHPWIQVDGVAPDKPLDSAVLSRLKQFSAMNKLKKMALIIIAETLSEEEIAGLKEMFKMIDADNSGQITFEELKAGLKRVGANLKESEIYDLMQA-------------
     TKY53562.1:1-581-[Spatholobus-suberectus]-LKL>  (370) VLCHPWIQVDGVAPDKPLDSAVLSRLKQFSAMNKLKKMALIIIAENLSEEEIAGLKEMFKMIDTDNSGQITFEELKAGLKRVGANLKESEIYDLMQA-------------
            XP_003535945.1:1-581-[Glycine-max]-LKL>  (370) VLCHPWIQVDGVAPDKPLDSAVLSRLKQFSAMNKLKKMALIIIAESLSEEEIAGLKEMFKMIDADNSGQITFEELKAGLKRVGANLKESEIYDLMQA-------------
            XP_003555695.1:1-579-[Glycine-max]-LKL>  (368) VLCHPWIQVDGVAPDKPLDSAVLSRLKQFSAMNKLKKMALIIIAESLSEEEIAGLKEMFKMIDADNSGQITFEELKAGLKRVGANLKESEIYDLMQA-------------
           XP_028221716.1:1-579-[Glycine-soja]-LKL>  (368) VLCHPWIQVDGVAPDKPLDSAVLSRLKQFSAMNKLKKMALIIIAESLSEEEIAGLKEMFKMIDADNSGQITFEELKAGLKRVGANLKESEIYDLMQA-------------
      XP_007145376.1:1-581-[Phaseolus-vulgaris]-LKL>  (370) VLCHPWIQVDGVAPDKPLDSAVLSRLKQFSAMNKLKKMALIIIAESLSEEEIAGLKEMFKMIDADNSGQITFEELKAGLKRVGANLNESEIYDLMQA-------------
 XP_014513811.1:1-581-[Vigna-radiata-var.-radiata]-...  (370) VLCHPWIQVDGVAPDKPLDSAVLSRLKQFSAMNKLKKMALIIIAESLSEEEIAGLKEMFKMIDADNSGQITFEELKAGLKRVGANLNESEIYDLMQA-------------
     XP_027936460.1:1-581-[Vigna-unguiculata]-LKL>  (370) VLCHPWIQVDGVAPDKPLDSAVLSRLKQFSAMNKLKKMALIIIAESLSEEEIAGLKEMFKMIDADNSGTITFEELKAGLKRVGANLNESEIYDLMQA-------------
      XP_022742028.1:1-583-[Durio-zibethinus]-LKL>  (372) VLCHPWIQINGVAPDKPLDSAVLSRLKQFSAMNKLKKMALRVIAESLSEEEIAGLKEMFKMIDTDNSGQITFEELKAGLKRVGANLKESEIYDLMQA-------------
      XP_022742029.1:1-561-[Durio-zibethinus]-LKL>  (350) VLCHPWIQINGVAPDKPLDSAVLSRLKQFSAMNKLKKMALRVIAESLSEEEIAGLKEMFKMIDTDNSGQITFEELKAGLKRVGANLKESEIYDLMQA-------------
      XP_022742030.1:1-598-[Durio-zibethinus]-LKL>  (372) VLCHPWIQINGVAPDKPLDSAVLSRLKQFSAMNKLKKMALRVIAESLSEEEIAGLKEMFKMIDTDNSGQITFEELKAGLKRVGANLKESEIYDLMQAVSMGLNLDIACLK
         XP_031258895.1:1-579-[Pistacia-vera]-LKL>  (368) VLCHPWVQIDGVAPDKPLDSAVLSRLKQFSAMNKLKKMALRVIAETLSEEEIAGLKEMFKMIDTDNSGQITFEELKAGLKRVGANLKESEIYDLMQA-------------
    KZV55254.1:1-572-[Dorcoceras-hygrometricum]-LKL>  (359) VLCHPWVQVDGVAPDRPLDSAVLSRLKQFSAMNRLKKMALRVIAESLSEEEIAGLKQMFKMLDADNSGQITFEELKVGLKRAGANLNESEIYDLMQA-------------
   PIN20243.1:1-575-[Handroanthus-impetiginosus]-LKL>  (363) VLCHPWVQVEGVAPDKPLGSAVLSRLKQFSAMNRLKKMAFRVIAESLSEDEIAGLKQMFKMIDTDNSGQITFEELKAGLKRVGANLKESEIYDLMQA-------------
        XP_011076878.1:1-571-[Sesamum-indicum]-LKL>  (359) VLCHPWVQVDGVAPDKPLDSAVLSRMKHFSAMNRLKKMALRVIAESLSEDEIAGLKEMFKMIDTDNSGQITFEELKAGLKRVGANLMESEIYDLMQA-------------
         XP_023767719.1:1-568-[Lactuca-sativa]-LKL>  (357) VLCHPWVQVDGVAPDKPLDSAVLSRMKQFTAMNKLKKMALRVIAESLSEEEIAGLKQMFEMIDTDNSGQITFDELKDGLKRVGANLNESEIYDLMQA-------------
 RWR80342.1:1-588-[Cinnamomum-micranthum-f.-kanehir...  (380) VLCHPWVQVDGVAPDKPLDSAVLSRLKQFSAMNKLKKMALRVIAESLSEEEIAGLKEMFKMIDADNSGQITFEELKAGLKRVGANLKESEIYALMEA-------------
                                                           551                                                                                                         660
       NP_196107.1:1-610-[Arabidopsis-thaliana]-LKL>  (499) --ADVDNSGTIDYKEFIAATLHLNKIEREDHLFAAFTYFDKDGSGYITPDELQQACEEFGVEDVRIEELMRDVDQDNDGRIDYNEFVAMMQKGSITGGPV-KMGLEKSFS
 XP_002873173.1:1-610-[Arabidopsis-lyrata-subsp.-ly...  (499) --ADVDNSGTIDYKEFIAATLHLNKIEREDHLFAAFTYFDKDGSGYITPDELQQACEEFGVEDVRIEELMRDVDQDNDGRIDYNEFVAMMQKGSITGGPV-KMGLEKSFS
       XP_006287286.1:1-618-[Capsella-rubella]-LKL>  (507) --ADVDNSGTIDYKEFIAATLHLNKIEREDHLFAAFTYFDKDGSGYITPDELQQACEEFGVEDVRIEELMRDVDQDNDGRIDYNEFVAMMQKGSITGGPV-KMGLEKSFS
        XP_010491015.1:1-606-[Camelina-sativa]-LKL>  (495) --ADVDNSGTIDYKEFIAATLHLNKIEREDHLFAAFTYFDKDGSGYITPDELQQACEEFGVEDVRIEELMRDVDQDNDGRIDYNEFVAMMQKGSITGGPV-KMGLEKSFS
          ABE73345.1:1-625-[Isatis-tinctoria]-LKL>  (514) --ADVDNSGTIDYKEFIAATLHLNKIEREDHLFAAFTYFDKDGSGYITPDELQQACEEFGVEDVRIEEMMRDVDQDNDGRIDYNEFVAMMQKGSITGVPV-KMGLEKSFS
        ESQ40392.1:1-597-[Eutrema-salsugineum]-LKL>  (486) --ADVDNSGTIDYKEFIAATLHLNKIEREDHLFAAFTYFDKDGSGYITPDELQQACEEFGVEDVRIEEMMRDVDQDNDGQIDYNEFVAMMQKGSITGGPV-KMGLEKSFS
     XP_006398940.1:1-613-[Eutrema-salsugineum]-LKL>  (502) --ADVDNSGTIDYKEFIAATLHLNKIEREDHLFAAFTYFDKDGSGYITPDELQQACEEFGVEDVRIEEMMRDVDQDNDGQIDYNEFVAMMQKGSITGGPV-KMGLEKSFS
    CAA7033892.1:1-601-[Microthlaspi-erraticum]-LKL>  (490) --ADVDNSGTIDYKEFIAATLHLNKIEREDHLFAAFTYFDKDGSGYITPDELQQACEEFGVEDVRIEEMMRDVDQDNDGRIDYNEFVAMMHKGSITGGPL-KMGLEKSFS
    CAA7044380.1:4-481-[Microthlaspi-erraticum]-LKL>  (367) --ADVDNSGTIDYKEFIAATLHLNKIEREDHLFAAFTYFDKDGSGYITPDELQQACEEFGVEDVRIEEMMRDVDQDNDGRIDYNEFVAMMHKGSITGGPL-KMGLEKSFS
          KAF2536503.1:1-592-[Brassica-cretica]-LKL>  (481) --ADVDNSGTIDYKEFIAATLHLNKIEREDHLFAAFTYFDKDGSGYITPDELQQACEEFGVEDVRIEEMMRDVDQDNDGRIDYNEFVAMMQKGSITGGPV-KMGLEKSFS
 XP_013611152.1:1-592-[Brassica-oleracea-var.-olera...  (481) --ADVDNSGTIDYKEFIAATLHLNKIEREDHLFAAFTYFDKDGSGYITPDELQQACEEFGVEDVRIEEMMRDVDQDNDGRIDYNEFVAMMQKGSITGGPV-KMGLEKSFS
          XP_009122558.1:1-591-[Brassica-rapa]-LKL>  (480) --ADVDNSGTIDYKEFIAATLHLNKIEREDHLFAAFTYFDKDGSGYITPDELQQACEEFGVEDVRIEEMMRDVDQDNDGRIDYNEFVAMMQKGSITGGPV-KMGLEKSFS
         XP_013715640.1:1-592-[Brassica-napus]-LKL>  (481) --ADVDNSGTIDYKEFIAATLHLNKIEREDHLFAAFTYFDKDGSGYITPDELQQACEEFGVEDVRIEEMMRDVDQDNDGRIDYNEFVAMMQKGSITGGPV-KMGLEKSFS
          VDD34618.1:1-595-[Brassica-oleracea]-LKL>  (484) --ADVDNSGTIDYKEFIAATLHLNKIEREDHLFAAFTYFDKDGSGYITPDELQQACEEFGVEDVRIEEMMRDVDQDNDGRIDYNEFVAMMQKGSITGGPV-KMGLEKSFS
      XP_018446206.1:1-609-[Raphanus-sativus]-LKL>  (498) --ADVDNSGTIDYKEFIAATLHLNKIEREDHLFAAFTYFDKDGSGYITPDELQQACEEFGVEDFRIEEMMRDVDQDNDGRIDYNEFVAMMQKGSITGGPV-KMGLEKSFS
              KFK24927.1:1-598-[Arabis-alpina]-LKL>  (487) --ADVDNSGTIDYKEFIAATLHLNKIEREDHLFAAFTYFDKDGSGYITPDELQHACEEFGVEDVRIEEMMRDVDQDNDGRIDYNEFVAMMHKGSITGGPV-KMGLEKSFS
          VVB13251.1:1-604-[Arabis-nemorensis]-LKL>  (493) --ADVDNSGTIDYKEFIAATLHLNKIEREDHLFAAFTYFDKDGSGYITPDELQQACEEFGVEDVRIEEMMRDVDQDNDGRIDYNEFVAMMHKGSITGGPV-KMGLEKSFS
          KAB5543975.1:1-571-[Salix-brachista]-LKL>  (457) --ADVDNSGTIDYGEFIAATLHLNKIERQDHLYAAFSYFDKDGSGYITPDELQQACEEFGLEDVRLEEMIREVDQDNDGRIDYNEFVAMMQKGN-AAGPA-RKGLEHSFS
               TKR66428.1:1-579-[Populus-alba]-LKL>  (465) --ADVDNSGTIDYGEFIAATLHLNKIERQDHLFAAFSYFDKDGSGYITPDELQQACEEFGIEDIRLEEMIREVDQDNDGRIDYNEFVAMMQKGN-VAGPA-RKGLEHSFS
               TKS17136.1:1-575-[Populus-alba]-LKL>  (465) --ADVDNSGTIDYGEFIAATLHLNKIERDDHLFAAFSYFDKDGSGYITPDELQKACEEFGWEDVRLEEMIREVDQDNDGRIDYNEFVAMMQKGN-VASPA-SHSFSINFR
     XP_002315411.1:1-579-[Populus-trichocarpa]-LKL>  (465) --ADVDNSGTIDYGEFIAATLHLNKIERDDHLFAAFSYFDKDGSGYITPDELQKACEEFGWEDVRLEEMIREVDQDNDGRIDYNEFVAMMQKGN-VASPA-RKGLEHSFS
      XP_011024127.1:1-579-[Populus-euphratica]-LKL>  (465) --ADVDNSGTIDYGEFIAATLHLNKIEREDHLFAAFSYFDKDGSGYITPDELQQACEEFGWEDVRLEEMIREVDQDNDGRIDYNEFVAMMQKGN-VASPA-RKGLEHSFS
           RDX78984.1:1-581-[Mucuna-pruriens]-LKL>  (467) --ADIDNSGTIDYGEFLAATLHRNKIEREDNLFAAFSYFDKDGSGYITQEELQQACDEFGIKDVRLEEIIKEIDEDNDGRIDYNEFVAMMQKGNLPAVGK--KGLENSFS
     TKY53562.1:1-581-[Spatholobus-suberectus]-LKL>  (467) --ADIDNSGTIDYGEFLAATLHRNKIERGDNLFAAFSYFDKDGSGYITQEELQQACDEFGIKDVHLEEIIKEIDEDNDGRIDYNEFVAMMQKGNLPAVGK--KGLENSFS
            XP_003535945.1:1-581-[Glycine-max]-LKL>  (467) --ADVDNSGTIDYGEFLAATLHRNKIEREDNLFAAFSYFDKDGSGYITQEELQQACDEFGIKDVRLEEIIKEIDEDNDGRIDYNEFVAMMQKGNLPAVGK--KGLENSFS
```

```
XP_003555695.1:1-579-[Glycine-max]-LKL>  (465) --ADVDNSGTIDYGEFLAATLHRNKIEREDNLFAAFSYFDKDGSGYITQEELQQACDEFGIKDVRLEEIIKEIDEDNDGRIDYNEFVAMMQKGNLPAVGK--KGLENSFS
XP_028221716.1:1-579-[Glycine-soja]-LKL>  (465) --ADVDNSGTIDYGEFLAATLHRNKIEREDNLFAAFSYFDKDGSGYITQEELQQACDEFGIKDVRLEEIIKEIDEDNDGRIDYNEFVAMMQKGNLPAVGK--KGLENSFS
XP_007145376.1:1-581-[Phaseolus-vulgaris]-LKL>  (467) --ADIDNSGTIDYGEFLAATLHRNKIEREDNLFAAFSYFDKDGSGYITQEELQQACDEFGIKDVRLEEIIKEIDEDNDGRIDYNEFVAMMQKGNLPSVGK--KGLENSFS
XP_014513811.1:1-581-[Vigna-radiata-var.-radiata]-...  (467) --ADIDNSGTIDYGEFLAATLHRNKIEREDNLFAAFSYFDKDGSGYITQEELQQACDEFGIKDVRLEEIIKEIDEDNDGRIDYNEFVAMMQKGNLPAIGK--KGLENSFS
XP_027936460.1:1-581-[Vigna-unguiculata]-LKL>  (467) --ADIDNSGTIDYGEFLAATLHRNKIEREDHLFAAFSYFDKDGSGYITQEELQQACDEFGIKDVRLEEIIKEIDEDNDGRIDYNEFVAMMQKGNLPVVGK--KGLENSFS
XP_022742028.1:1-583-[Durio-zibethinus]-LKL>  (469) --ADVDNSGTIDYGEFVAATLHLNKIEREDYLFAAFSYFDKDGSGYITLDELQEACKEFGIEDIRLEELIREVDQDNDGTIDYNEFVAMMQKGN-VAGNG-KKGLENSFS
XP_022742029.1:1-561-[Durio-zibethinus]-LKL>  (447) --ADVDNSGTIDYGEFVAATLHLNKIEREDYLFAAFSYFDKDGSGYITLDELQEACKEFGIEDIRLEELIREVDQDNDGTIDYNEFVAMMQKGN-VAGNG-KKGLENSFS
XP_022742030.1:1-598-[Durio-zibethinus]-LKL>  (482) LQADVDNSGTIDYGEFVAATLHLNKIEREDYLFAAFSYFDKDGSGYITLDELQEACKEFGIEDIRLEELIREVDQDNDGTIDYNEFVAMMQKGN-VAGNG-KKGLENSFS
XP_031258895.1:1-579-[Pistacia-vera]-LKL>  (465) --ADVDNSGTIDYGEFIAATLHLNKIEREDHLFAAFSYFDKDGSGYITPDELQQACEEFGIEDVRLEEMIREVDQDNDGRIDYNEFVAMMQNGTVSGPPK--KGLQNSFS
KZV55254.1:1-572-[Dorcoceras-hygrometricum]-LKL>  (456) --ADVDNSGTIDYGEFVAATLHLNKIEREDHLFAAFSYFDKDGSGYITPDELQHVCQEYGFDDSRLEEMIREVDQDNDGRIDYNEFVTMMQKGNTVAGGGGKRGLEKSFS
PIN20243.1:1-575-[Handroanthus-impetiginosus]-LKL>  (460) --ADVDNSGTIDYGEFIAATLHFNKIEREDHLFAAFSYFDKDGSGYITPDELQQACQEFGLDDSRLEEMIREVDQDNDGRIDYNEFVTMMQKGNPIAGGV-KRGLENSFS
XP_011076878.1:1-571-[Sesamum-indicum]-LKL>  (456) --ADVDNSGTIDYGEFIAATLHLNKIEREDHLFTAFSYFDKDGSGYITPDELQQACVEFGFDDTRLEEMIREVDQDNDGRIDYNEFVTMMQKGNPAAGGG-KRGMENSFS
XP_023767719.1:1-568-[Lactuca-sativa]-LKL>  (454) --ADVDNSGTIDYGEFVAATLHMNKIEKENHLFAAFSYFDKDGSGYITADELQHACEEFG-IDAHLEELIQDVDQDNDGRIDYNEFVAMMQGHANVNVGA-KKSLDTSFS
RWR80342.1:1-588-[Cinnamomum-micranthum-f.-kanehir...  (477) --ADVDNSGTIDYGEFIAATLHLNKIEREDHLFAAFSYFDKDGSGYITPDELQQACEEFGIEDVQLEDMIREVDQDNDGRIDYNEFVEMMQKGN--AGFG-KKGIQNN--
      661
NP_196107.1:1-610-[Arabidopsis-thaliana]-LKL>  (606) IALKL----
XP_002873173.1:1-610-[Arabidopsis-lyrata-subsp.-ly...  (606) IALKL----
XP_006287286.1:1-618-[Capsella-rubella]-LKL>  (614) IALKL----
XP_010491015.1:1-606-[Camelina-sativa]-LKL>  (602) IALKL----
ABE73345.1:1-625-[Isatis-tinctoria]-LKL>  (621) IALKL----
ESQ40392.1:1-597-[Eutrema-salsugineum]-LKL>  (593) INLKL----
XP_006398940.1:1-613-[Eutrema-salsugineum]-LKL>  (609) INLKL----
CAA7033892.1:1-601-[Microthlaspi-erraticum]-LKL>  (597) IALKL----
CAA7044380.1:4-481-[Microthlaspi-erraticum]-LKL>  (474) IALKL----
KAF2536503.1:1-592-[Brassica-cretica]-LKL>  (588) IALKL----
XP_013611152.1:1-592-[Brassica-oleracea-var.-olera...  (588) IALKL----
XP_009122558.1:1-591-[Brassica-rapa]-LKL>  (587) IALKL----
XP_013715640.1:1-592-[Brassica-napus]-LKL>  (588) IALKL----
VDD34618.1:1-595-[Brassica-oleracea]-LKL>  (591) IALKL----
XP_018446206.1:1-609-[Raphanus-sativus]-LKL>  (605) IALKL----
KFK24927.1:1-598-[Arabis-alpina]-LKL>  (594) ISLKL----
VVB13251.1:1-604-[Arabis-nemorensis]-LKL>  (600) ISLKL----
KAB5543975.1:1-571-[Salix-brachista]-LKL>  (563) INFREALKL
TKR66428.1:1-579-[Populus-alba]-LKL>  (571) INFREALKL
TKS17136.1:1-575-[Populus-alba]-LKL>  (571) EALKL----
XP_002315411.1:1-579-[Populus-trichocarpa]-LKL>  (571) INFREALKL
XP_011024127.1:1-579-[Populus-euphratica]-LKL>  (571) INFREALKL
RDX78984.1:1-581-[Mucuna-pruriens]-LKL>  (573) IKFREALKL
TKY53562.1:1-581-[Spatholobus-suberectus]-LKL>  (573) IKFREALKL
XP_003535945.1:1-581-[Glycine-max]-LKL>  (573) VKFREALKL
XP_003555695.1:1-579-[Glycine-max]-LKL>  (571) IKFREALKL
XP_028221716.1:1-579-[Glycine-soja]-LKL>  (571) IKFREALKL
XP_007145376.1:1-581-[Phaseolus-vulgaris]-LKL>  (573) IKFREALKL
XP_014513811.1:1-581-[Vigna-radiata-var.-radiata]-...  (573) IKFRDALKL
XP_027936460.1:1-581-[Vigna-unguiculata]-LKL>  (573) IKFREALKL
XP_022742028.1:1-583-[Durio-zibethinus]-LKL>  (575) IRFREALKL
XP_022742029.1:1-561-[Durio-zibethinus]-LKL>  (553) IRFREALKL
XP_022742030.1:1-598-[Durio-zibethinus]-LKL>  (590) IRFREALKL
XP_031258895.1:1-579-[Pistacia-vera]-LKL>  (571) INFREALKL
KZV55254.1:1-572-[Dorcoceras-hygrometricum]-LKL>  (564) INFRDALKL
PIN20243.1:1-575-[Handroanthus-impetiginosus]-LKL>  (567) INFREALKL
XP_011076878.1:1-571-[Sesamum-indicum]-LKL>  (563) INFREALKL
XP_023767719.1:1-568-[Lactuca-sativa]-LKL>  (560) IKFREALKL
RWR80342.1:1-588-[Cinnamomum-micranthum-f.-kanehir...  (580) IGFREALKL
```

# K19_ AT5G03730.1

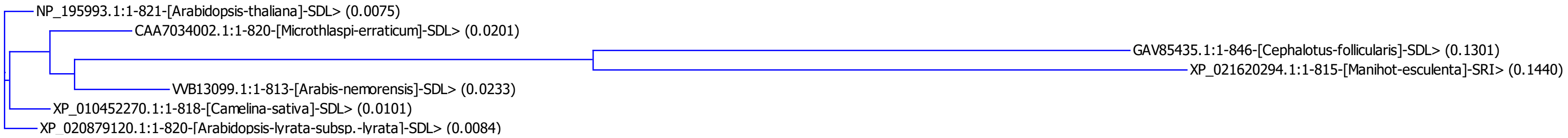


```
                                                           1                                                                                                  100
          NP_195993.1:1-821-[Arabidopsis-thaliana]-SDL>    (1) MEMPGRRSNYTLLSQFSDDQVSVSVTGAPPPHYDSLSSENR--SNHNSGNTGKAKAERGGFDWDPSGGGGGDHRLNNQ--PNRVGNN-MYASSLGLQRQS
       CAA7034002.1:1-820-[Microthlaspi-erraticum]-SDL>    (1) MEMPGRRSNYTLLSQFSDDQVSVSVTGAPPPHYDSLSSENRSASNNNSGNTGKTKADRGGFDWDPSG-G--DHRLSGQ--PNRAGN--MYSSSLGLQRQS
        GAV85435.1:1-846-[Cephalotus-follicularis]-SDL>    (1) MEMAGRRSNYTLLSQYPDDQFSVG-----APYYESLSGVDS--------RKADKQDRGGAFDWDSNT---ADHRLNQQ--SNRVVAN-VYTSSIGLQRQS
          XP_021620294.1:1-815-[Manihot-esculenta]-SRI>    (1) MEMPGRRPNYTLLSQYPDENLAAAP---PPPYYDSSISSDS---------KSSKLKPDRSFDWDSSG----DHKVNQQGNSNRIGN-MNLFSSIGLQRQS
            VVB13099.1:1-813-[Arabis-nemorensis]-SDL>    (1) MEMPGRRSNYTLLSQFSDDQVSVSVTGAPPPHYDSLSSDNRSAGKNNSGNNGKTKAERGGFDWDPNV---ADHRLSNQ--PNRVGN--MYSSSLGLQRQS
          XP_010452270.1:1-818-[Camelina-sativa]-SDL>    (1) MEMPGRRSNYNLLSQFSDDQVSVSVTGAPPPHYDSLSSENRSASNNNSGNNGKNKADRSGFDWDPSGAG--DHRLSNQ--PNRGGNNNMYASSLGLQRQS
 XP_020879120.1:1-820-[Arabidopsis-lyrata-subsp.-ly...    (1) MEMPGRRSNYTLLSQFSEDQVSVSVTGAPPPHYDSLSSENR--SNNNSGNNGKTKTERGGFDWDPSGGGGGDHRLSNQ--PNRVGNN-MYASSLGLQRQS
                                                           101                                                                                                200
          NP_195993.1:1-821-[Arabidopsis-thaliana]-SDL>   (96) SGSSFGESSLSGDYYMPTLSAAANEIESVGFPQDDGFRLGFGGGGGDLRIQMAADSAGGSSSGKSWAQQTEESYQLQLALALRLSSEATCADDPNFLDPV
       CAA7034002.1:1-820-[Microthlaspi-erraticum]-SDL>   (94) SGSSFGESSLSGDYYMPTLSAAANEIESIGFPQDDGFRLGFGGGGGDLRIQMAADSAGGSSSGKSWAQQTEESYQLQLALALRLSSEATCADDPNFLDPV
        GAV85435.1:1-846-[Cephalotus-follicularis]-SDL>   (82) SGSSFGESSFSGEYYVPTLSNAAASEIDGFGCGCYGMEDGFKVGGKE-----VGLAGGPSSSGKSWAQQTEESYQLQLALALRLSSEATCADDPNFLDPV
          XP_021620294.1:1-815-[Manihot-esculenta]-SRI>   (84) SGSSFGESSLSGEYCAPTLSTAGGNEIEAYGYMHEDGNLMRGRAVDAAVIP----GMGTGSSVKSWAQQTEESYQLQLALALRLSSEATCADDPNFLDPM
            VVB13099.1:1-813-[Arabis-nemorensis]-SDL>   (94) SGSSFGESSLSGDYYMPTLSTAANEIESVGFPQDDGFRLGFGGGGGDLRIQMTADSAGGSASGKSWAQQTEESYQLQLALALRLSSEATCADDPNFLDPV
          XP_010452270.1:1-818-[Camelina-sativa]-SDL>   (97) SGSSFGESSLSGDYYMPTLSAAANEIESVGFPQDDGFRLGFGGGGGDLRIQMTADSAGGSSSGKSWAQQTEESYQLQLALALRLSSEATCADDPNFLDPV
 XP_020879120.1:1-820-[Arabidopsis-lyrata-subsp.-ly...   (96) SGSSFGESSLSGDYYMPTLSAAANEIESVGFPQDDGFRLGFGGGGGDLRIQMAGESAGGSSSGKSWAQQTEESYQLQLALALRLSSEATCADDPNFLDPV
                                                           201                                                                                                300
          NP_195993.1:1-821-[Arabidopsis-thaliana]-SDL>  (196) PDESALRTSP--SSAETVSHRFWVNGCLSYYDKVPDGFYMMNGLDPYIWTLCIDLHESGRIPSIESLRAVDSGVDSSLEAIIVDRRSDPAFKELHNRVHD
       CAA7034002.1:1-820-[Microthlaspi-erraticum]-SDL>  (194) PDESALRTSP--SSAETVSHRFWVNGCLSYYDKVPDGFYMINGLDPYIWTLCIDLHESGRIPSIESLRAVDSGADSSLEAILVDRRSDPSFKELHNRVHD
        GAV85435.1:1-846-[Cephalotus-follicularis]-SDL>  (177) PDEASSRSSSGSSSSESVSHRFWVNGCLSYFDKIPDGFYVIHGIDPYVWTVCTDLHENGRIPSLESLRSVDPIANSSIEVVLIDRRSDPSLKELQNRVHG
          XP_021620294.1:1-815-[Manihot-esculenta]-SRI>  (180) PDESGLRSTS-SNSPEALSHRFWVNGCLSYFDKIPDGFYQIHGMDPYVWTVCIDLQENGRIPSIELLKSVDPSVDSSLEVVLVDRRSDPSLKELQNRVHS
            VVB13099.1:1-813-[Arabis-nemorensis]-SDL>  (194) PDESALRTSP--SSAETVSHRFWVNGCLSYYDKVPDGFYMMHGLDPYIWTLCIDLHESGRIPSIESLRAVDSGVDSSLEAIIVDRRSDPTFKELHNRVHD
          XP_010452270.1:1-818-[Camelina-sativa]-SDL>  (197) PDESALRTSP--SSAETVSHRFWVNGCLSYYDKVPDGFYMMNGLDPYIWTLCIDLHESGRIPSIESLRAVDSGVDSSLEAIIVDRRSDPAFKELHNRVHD
 XP_020879120.1:1-820-[Arabidopsis-lyrata-subsp.-ly...  (196) PDESALRTSP--SSAETVSHRFWVNGCLSYYDKVPDGFYMMNGLDPYIWTLCIDLHESGRIPSIESLRAVDSGVDSSLEAIIVDRRSDPAFKELHNRVHD
                                                           301                                                                                                400
          NP_195993.1:1-821-[Arabidopsis-thaliana]-SDL>  (294) ISCSCITTKEVVDQLAKLICNRMGGPVIMGEDELVPMWKECIDGLKEIF-KVVVPIGSLSVGLCRHRALLFKVLADIIDLPCRIAKGCKYCNRDDAASCL
       CAA7034002.1:1-820-[Microthlaspi-erraticum]-SDL>  (292) ISCSCITTKEVVDQLAKLVCNRMGGPVIMGEDELVPMWKECIDGLKECL-KVVVPIGSLSVGLCRHRALLFKVLADIIDLPCRIAKGCKYCDRDDAASCL
        GAV85435.1:1-846-[Cephalotus-follicularis]-SDL>  (277) ISCSCITLKEVVDQLAKLVCNRMGGSATLGEDDIVSVWKECSDDLKDCLGSVVVPMGSLSVGLCRHRALLFKVLADSIHLPCRIVKGCKYCTRDDASSCL
          XP_021620294.1:1-815-[Manihot-esculenta]-SRI>  (279) ISCSCITTKEVVDQLAKLVSNRMGGLTNMAEDDLVSNWRDCSNDLKDCLGSVVVPIGSLSVGLCRHRALLFKVLADTIDLPCRIARGCKYCKRDDASSCL
            VVB13099.1:1-813-[Arabis-nemorensis]-SDL>  (292) ISCSCITTKEVVDQLAKLVCNRMGGPVMMGEDELVPMWKECINGLKECF-KVVVPIGSLSVGLCRHRALLFKVLADIIDLPCRIAKGCKYCNRDDSASCL
          XP_010452270.1:1-818-[Camelina-sativa]-SDL>  (295) ISCSCITTKEVVDQLAKLICNRMGGPVIMGEDELVPMWKECIDGLKEIF-KVVVPIGSLSVGLCRHRALLFKVLADIIDLPCRIAKGCKYCNRDDAASCL
 XP_020879120.1:1-820-[Arabidopsis-lyrata-subsp.-ly...  (294) ISCSCITTKEVVDQLAKLICNRMGGPVIMGEDELVPMWKECIDGLKEIF-KVVVPIGSLSVGLCRHRALLFKVLADIIDLPCRIAKGCKYCNRDDAASCL
                                                           401                                                                                                500
          NP_195993.1:1-821-[Arabidopsis-thaliana]-SDL>  (393) VRFGLDREYLVDLVGKPGHLWEPDSLLNGPSSISISSPLRFPRPKPVEPAVDFRLLAKQYFSDSQSLNLVFDPASD-------DMGFSMFHRQYDNPGGE
       CAA7034002.1:1-820-[Microthlaspi-erraticum]-SDL>  (391) VRFGLDREYLIDLVGKPGHLWEPDSLINGPSSISISSPLRFPRPRPVEPAVDYRLLAKQYFTDSQSLNLVFDPASD-------DLGFSMFHRQYDNPGGE
        GAV85435.1:1-846-[Cephalotus-follicularis]-SDL>  (377) VRFGLDREFFVDFLGKPGCLCEPDSLLNGPSTISISSPLRFPRYKSAEPTIDFRSVAKQYFADCQSLDLTFDDSSAG------DHGLSMFQKQSDKVGMD
          XP_021620294.1:1-815-[Manihot-esculenta]-SRI>  (379) VRFGLEREYLVDLVGKPGCLCEPDSLLNGPASISISSPLCFPRMKSAEPIIDFRTLAKQYFSDCQSLNIVFDDASAATVLDEAAPGFSMYPKKIERMDAE
            VVB13099.1:1-813-[Arabis-nemorensis]-SDL>  (391) VRFGLDREYLVDLVGKPGHLWEPDSLLNGPLTISISSPLRFPRPRPVEPAIDFRSLAKQYFSDSQSLNLVFDPASD-------DIGFSMFHR-----GGE
          XP_010452270.1:1-818-[Camelina-sativa]-SDL>  (394) VRFGLDREYLVDLVGKPGHLWEPDSLLNGPSSISISSPLRFPRPKPVEPAVDFRLLAKQYFTDSQSLNLVFDPASD-------DMGFSMFHRQYDNPGGE
 XP_020879120.1:1-820-[Arabidopsis-lyrata-subsp.-ly...  (393) VRFGLDREYLVDLVGKPGHLWEPDSLLNGPSSISISSPLRFPRPKPVEPAVDFRLLAKQYFSDSQSLNLVFDPASD-------DMGFSMFHRQYDNPGGE
                                                           501                                                                                                600
          NP_195993.1:1-821-[Arabidopsis-thaliana]-SDL>  (486) NDALAENGGG---------------------------SLPPSANMPPQNMMRASNQI-EAAPMNAPPISQPV-----------------------PNRAN
       CAA7034002.1:1-820-[Microthlaspi-erraticum]-SDL>  (484) NDVLPENGGG---------------------------SLPPSANMPPQNMMRASNQVQEAVPMNAPPISQPA-----------------------PNRAN
        GAV85435.1:1-846-[Cephalotus-follicularis]-SDL>  (471) RNNFVPIPIAGN------EVPKIARPNAHDSDSEHFKSCNPSHNIAHSTKMVKDPIPLEHIPHIQPPLALPDRRADTSKDLR-----FPERGQLVPSKAS
          XP_021620294.1:1-815-[Manihot-esculenta]-SRI>  (479) RNDPVQITTNSSEISQLPLPMKVARTSAQDRNAQNFKSYNPSQNIEQATNLMKDPIHLKHIPTRGHRDVQPLLSLSDQKVDTSKNSRFSEGFQLVSSKTS
            VVB13099.1:1-813-[Arabis-nemorensis]-SDL>  (479) NDVLAENGGG---------------------------SLPPSANMPPQNMMRAPNQVQEAVPINAPPINQP-------------------------VRAN
          XP_010452270.1:1-818-[Camelina-sativa]-SDL>  (487) NDALAENGG-------------------------------GSANMPPQNMMRASNQV-EAVPMNAPPINQTV-----------------------PNRAN
 XP_020879120.1:1-820-[Arabidopsis-lyrata-subsp.-ly...  (486) NDASAENGG----------------------------SLPPSANMPPQNMMRASNQV-EAVPMNAPPTNQPV-----------------------PNRAN
                                                           601                                                                                                700
          NP_195993.1:1-821-[Arabidopsis-thaliana]-SDL>  (535) RELGL-DGDDMDIPWCDLNIKEKIGAGSFGTVHRAEWHGSDVAVKILMEQDFHAERVNEFLREVAIMKRLRHPNIVLFMGAVTQPPNLSIVTEYLSRGSL
       CAA7034002.1:1-820-[Microthlaspi-erraticum]-SDL>  (534) RDLGL-DGDDMDIPWCDLNIKERIGAGSFGTVHRAEWHGSDVAVKILMEQDFHAERVNEFLREVAIMKRLRHPNIVLFMGAVTQPPNLSIVTEYLSRGSL
        GAV85435.1:1-846-[Cephalotus-follicularis]-SDL>  (560) KEISL-DVEDLNIPWSDLVLKERIGAGSFGTVHRADWQGSDVAVKILMEQDFHVERLEEFLREVTIMKRLRHPNIVLFMGAVTQPPNLSIVTEYLSRGSL
          XP_021620294.1:1-815-[Manihot-esculenta]-SRI>  (579) NELSLGDVEDLDIPWGDLILKERIGAGSFGTVHRADWHGSDVAVKILMEQDFHAERFKEFLREVAIMKRLRHPNIVLFMGAVTKPPNLSIVTEYLSRGSL
            VVB13099.1:1-813-[Arabis-nemorensis]-SDL>  (527) RDLGL-DGDDMDISWCDLNIKERIGAGSFGTVHRAEWHGSDVAVKILMEQDFHAERVNEFLREVAIMKRLRHPNIVLFMGAVTQPPNLSIVTEYLSRGSL
          XP_010452270.1:1-818-[Camelina-sativa]-SDL>  (532) RELGL-DGDDMDIPWCDLNIKEKIGAGSFGTVHRAEWHGSDVAVKILMEQDFHAERVNEFLREVAIMKRLRHPNIVLFMGAVTQPPNLSIVTEYLSRGSL
 XP_020879120.1:1-820-[Arabidopsis-lyrata-subsp.-ly...  (534) RELGL-DGDDMDIPWCDLNIKEKIGAGSFGTVHRAEWHGSDVAVKILMEQDFHAERVNEFLREVAIMKRLRHPNIVLFMGAVTQPPNLSIVTEYLSRGSL
```

```
                                                         701                                                                                               800
        NP_195993.1:1-821-[Arabidopsis-thaliana]-SDL>  (634) YRLLHKSGAREQLDERRRLSMAYDVAKGMNYLHNRNPPIVHRDLKSPNLLVDKKYTVKVCDFGLSRLKASTFLSSKSAAGTPEWMAPEVLRDEPSNEKSD
     CAA7034002.1:1-820-[Microthlaspi-erraticum]-SDL>  (633) YRLLHKSGAREQLDERRRLSMAYDVAKGMNYLHNRNPPIVHRDLKSPNLLVDKKYTVKVCDFGLSRLKASTFLSSKSAAGTPEWMAPEVLRDEPSNEKSD
      GAV85435.1:1-846-[Cephalotus-follicularis]-SDL>  (659) YRLLHKPGAREALDERRRWNMAYDVAKGMNYLHKRNPPIVHRDLKSPNLLVDKKYTVKVCDFGLSRLKKHTFLSSKSAAGTAEWMAPEVLRDEPSNEKSD
      XP_021620294.1:1-815-[Manihot-esculenta]-SRI>  (679) YRLLHKSGAREVLDERRRLNMAYDVAKGMNYLHKRNPPIVHRDLKSPNLLVDKKYTVKVCDFGLSRLKANTFLSSKSAAGTPEWMAPEVLRDEPSNEKSD
          VVB13099.1:1-813-[Arabis-nemorensis]-SDL>  (626) YRLLHKSGAREQLDERRRLSMAYDVAKGMNYLHNRNPPIVHRDLKSPNLLVDKKYTVKVCDFGLSRLKASTFLSSKSAAGTPEWMAPEVLRDEQSNEKSD
        XP_010452270.1:1-818-[Camelina-sativa]-SDL>  (631) YRLLHKSGAREQLDERRRLSMAYDVAKGMNYLHNRNPPIVHRDLKSPNLLVDKKYTVKVCDFGLSRLKASTFLSSKSAAGTPEWMAPEVLRDEPSNEKSD
XP_020879120.1:1-820-[Arabidopsis-lyrata-subsp.-ly...  (633) YRLLHKSGAREQLDERRRLSMAYDVAKGMNYLHNRNPPIVHRDLKSPNLLVDKKYTVKVCDFGLSRLKASTFLSSKSAAGTPEWMAPEVLRDEPSNEKSD
                                                         801                                                                                888
        NP_195993.1:1-821-[Arabidopsis-thaliana]-SDL>  (734) VYSFGVILWELATLQQPWGNLNPAQVVAAVGFKCKRLEIPRNLNPQVAAIIEGCWTNEPWKRPSFATIMDLLRPLIKSAVPPPNRSDL
     CAA7034002.1:1-820-[Microthlaspi-erraticum]-SDL>  (733) VYSFGVILWELATLQQPWGNLNPAQVVAAVGFKGKRLEIPRNLNPQVAAIIEGCWTNEPWKRPSFATIMDLLRPLIKSAVPQPNRSDL
      GAV85435.1:1-846-[Cephalotus-follicularis]-SDL>  (759) VYSFGVILWELATLQQPWGNLNTAQVVAAVGFKIERLGIPRDLNPQVAAIIEACWANEPWKRPSFTNIMESLRSLIKPPTPLPSRSDL
      XP_021620294.1:1-815-[Manihot-esculenta]-SRI>  (779) VYSFGVILWELATLQQPWSNLNPAQVCFLKLHDCSRI---------------------------------------------------
          VVB13099.1:1-813-[Arabis-nemorensis]-SDL>  (726) VYSFGVILWELATLQQPWGNLNPAQVVAAVGFKGKRLEIPRNLNPQVAAIIESCWTNEPWKRPSFATIMDLLRPLIKSAVPPPNRSDL
        XP_010452270.1:1-818-[Camelina-sativa]-SDL>  (731) VYSFGVILWELATLQQPWGNLNPAQVVAAVGFKCKRLEIPRNLNPQVAAIIEGCWTNEPWKRPSFATIMDLLRPLIKSAVPPPNRSDL
XP_020879120.1:1-820-[Arabidopsis-lyrata-subsp.-ly...  (733) VYSFGVILWELATLQQPWGNLNPAQVVAAVGFKCKRLEIPRNLNPQVAAIIEGCWTNEPWKRPSFATIMDLLRPLIKSAVPPPNRSDL
```
