# Supplementary material for: Identification of Arabidopsis Protein Kinases That Harbor Functional Type 1 Peroxisomal Targeting Signals
Source: Front Cell Dev Biol. 2022 Feb 15;10:745883. doi: 10.3389/fcell.2022.745883 (PMC8886021; doi:10.3389/fcell.2022.745883)
Supplement: Supplementary file 3 [file DataSheet3.PDF]

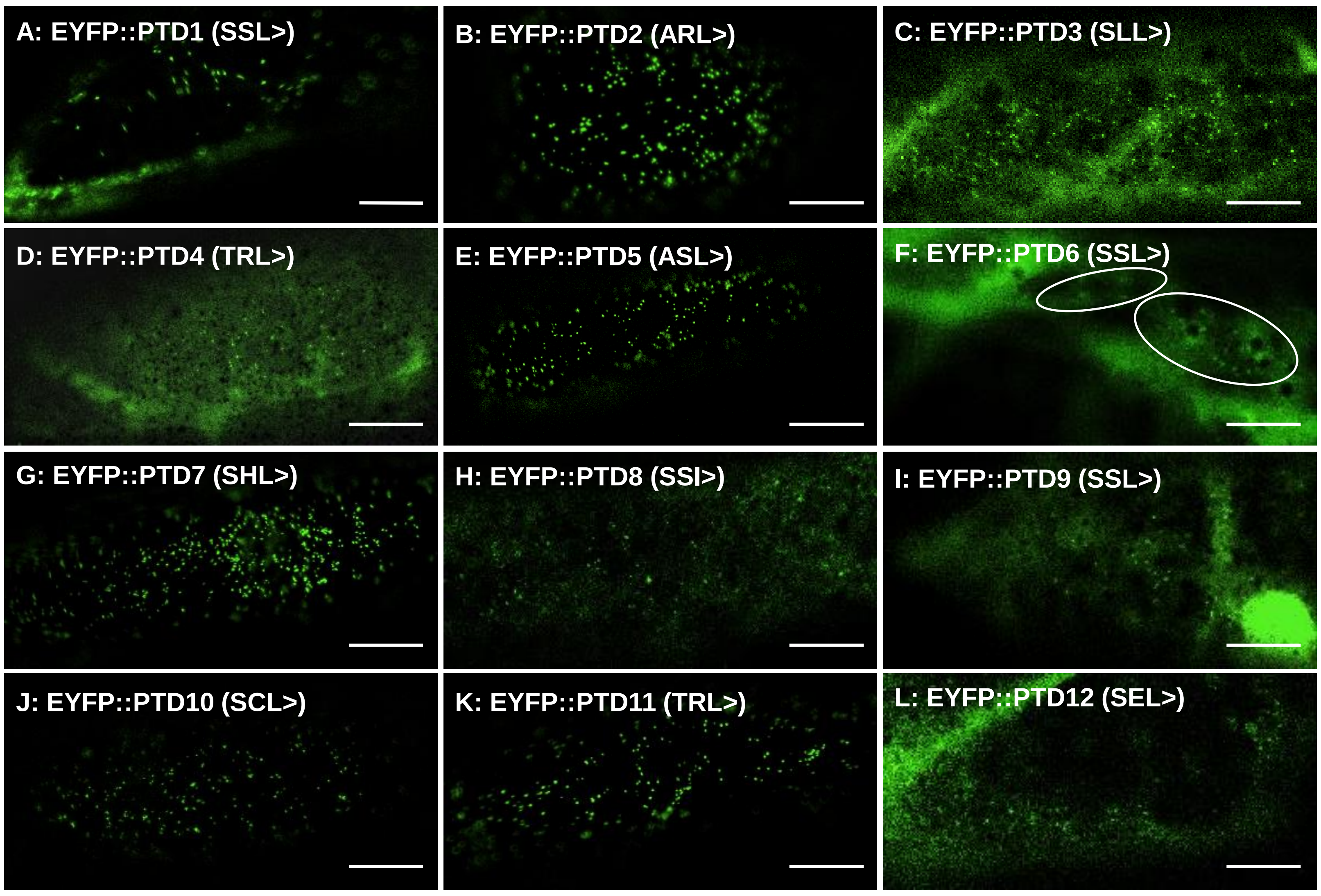

Supplemental Figure 2. Experimental investigation of *in vivo* peroxisome targeting by the C-terminal residues of selected protein kinases. To investigate the functionality of peroxisomal targeting of the 31 selected kinases (Supplemental Table 1), EYFP was extended C-terminally with each of the kinases' C-terminal decapeptides containing the putative peroxisomal targeting domains (PTD). The subcellular localization of the extended reporter proteins was investigated through transient expression in onion epidermal cells (after ~18 h expression at room temperature) upon biolistic bombardment. Twelve of the 31 extended reporters (Supplemental Table 1) were detected in punctate structures and are labelled. PTD refers to the protein kinase it was derived from (ex. PTD1=K1) and the C-terminal 3 residues are indicated in brackets. White ovals in F are highlighting the presence of punctate structures. The remaining 19 fused extended reporters remained in the cytosol (summarized in Supplemental Table 1) and are not shown. Representative images (produced by Nikon Confocal microscopy) are shown. Scale bars are 20 µm.

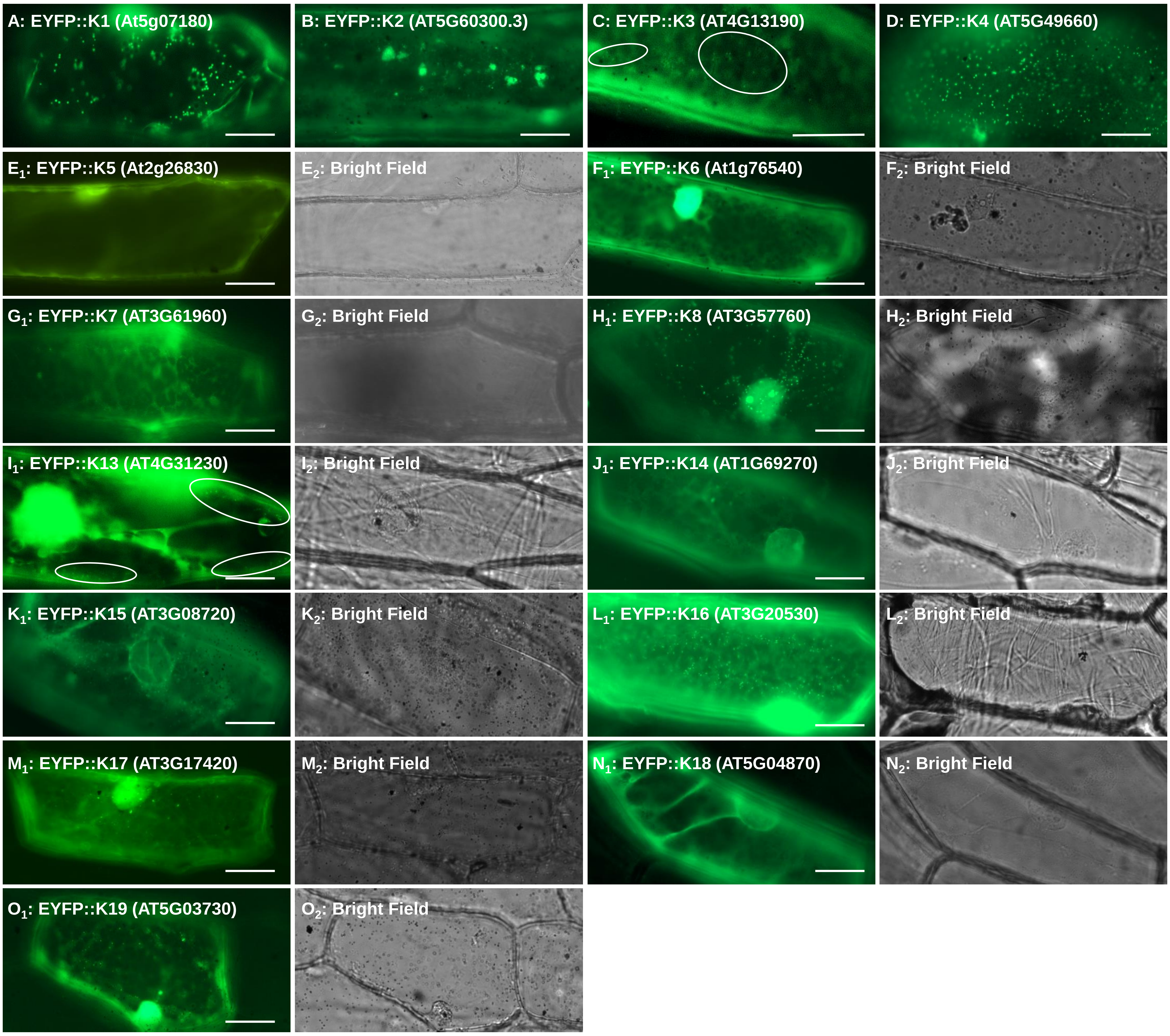

Supplemental Figure 3. Subcellular localization of full-length protein kinases with PTS1 signals. Onion epidermal cells were transformed biolistically with EYFP fusion constructs that were C-terminally fused with Arabidopsis protein kinase full-length cDNAs. The fusion constructs targeted to either cytosol and/or punctate structures, network-like structures, and/or nucleus. See Table 1 for the summary of these results and the comparison with the subcellular localization in tobacco cells. Representative images (produced by fluorescence microscopy) are shown. Scale bars are 20 µm.

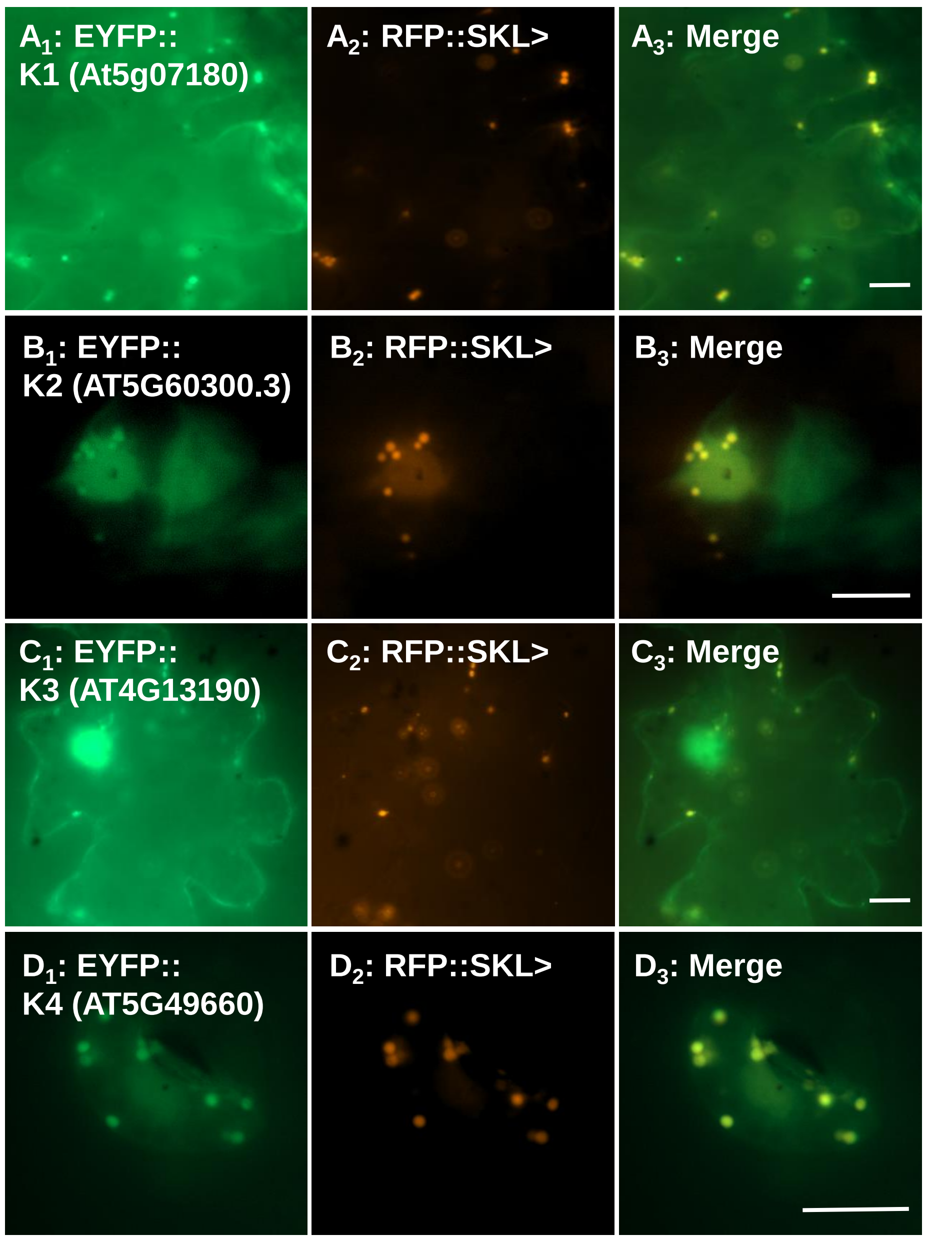

Supplemental Figure 4. Peroxisome targeting validation of RLK and RLCK designated protein kinases by in vivo subcellular targeting analysis in tobacco. *Nicotiana tabacum* cells were transformed biolistically with EYFP fusion constructs that were C-terminally fused with Arabidopsis full-length cDNAs. These 3 RLKs and 1 RLCK were demonstrated to have functional PTS1s (Figure 1, Supplemental Table 1) and their full-length fusion constructs targeted to punctate structures that coincided with RFP::SKL> in peroxisomes (see Table 1 for details and summary). Representative images (produced by fluorescence microscopy) are shown. Scale bars 5 µm.

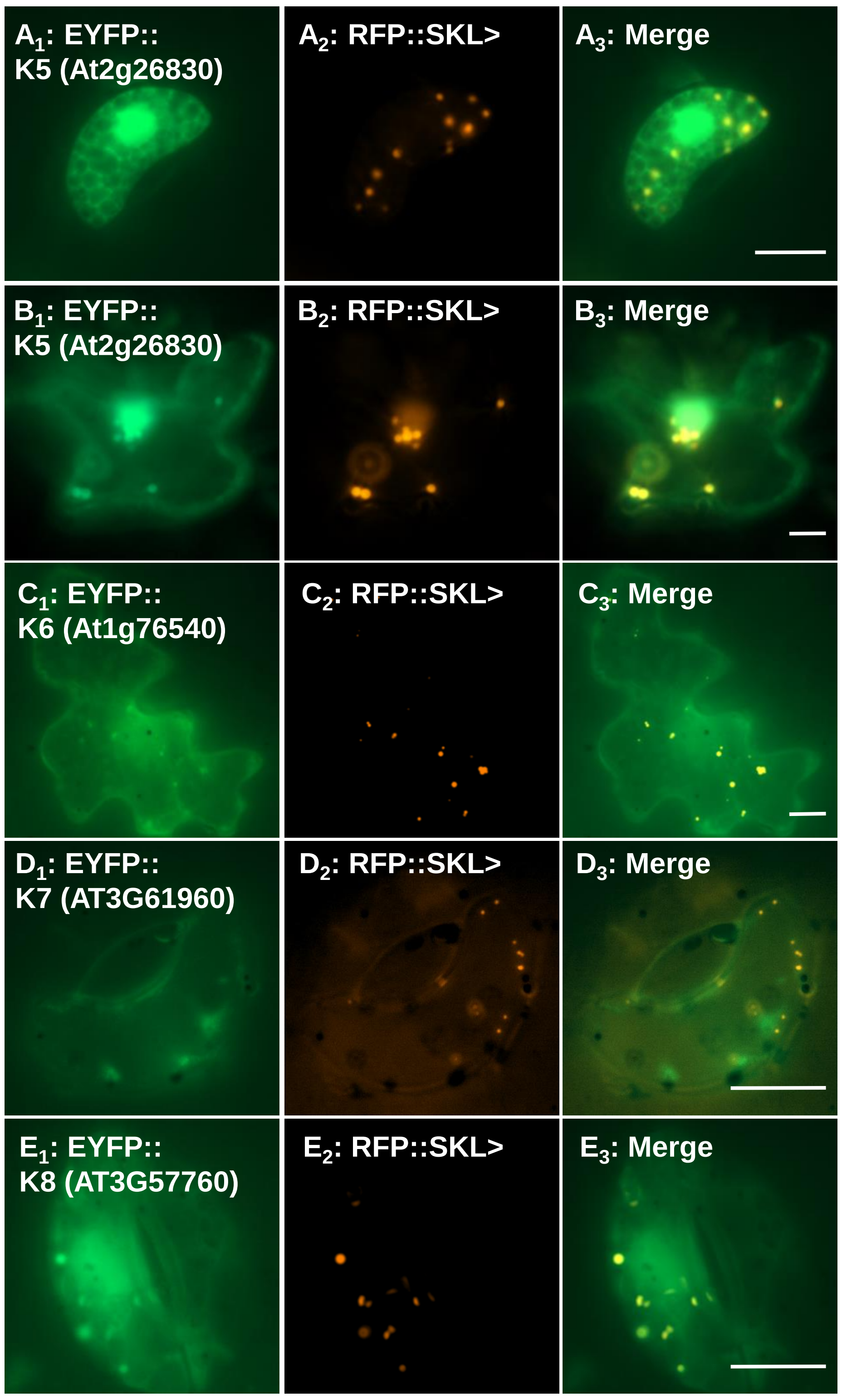

Supplemental Figure 5. Peroxisome targeting validation of designated soluble protein kinases by in vivo subcellular targeting analysis in tobacco. *Nicotiana tabacum* cells were transformed biolistically with EYFP fusion constructs that were C-terminally fused with Arabidopsis full-length cDNAs. These protein kinases were proved to have a functional PTS1 (Figure 1, Supplemental Table 1) and their full-length fusion constructs were targeted to either cytosol, punctate structures that coincided with RFP::SKL> in peroxisomes, or both. The K5 kinase fusion protein was mostly detected in the cytosol (A) but also partially detected in peroxisomes (B). Similarly, the K6 kinase however mostly detected in the cytosol, but peroxisomal targeting was detected (C). Only K7, which have a functional peroxisomal domain remained in the cytosol (D). These data prove the subcellular localization of three soluble kinases to peroxisomes (see Table 1 for more details and summary). Representative images (produced by fluorescence microscopy) are shown. Scale bars are 5 µm.

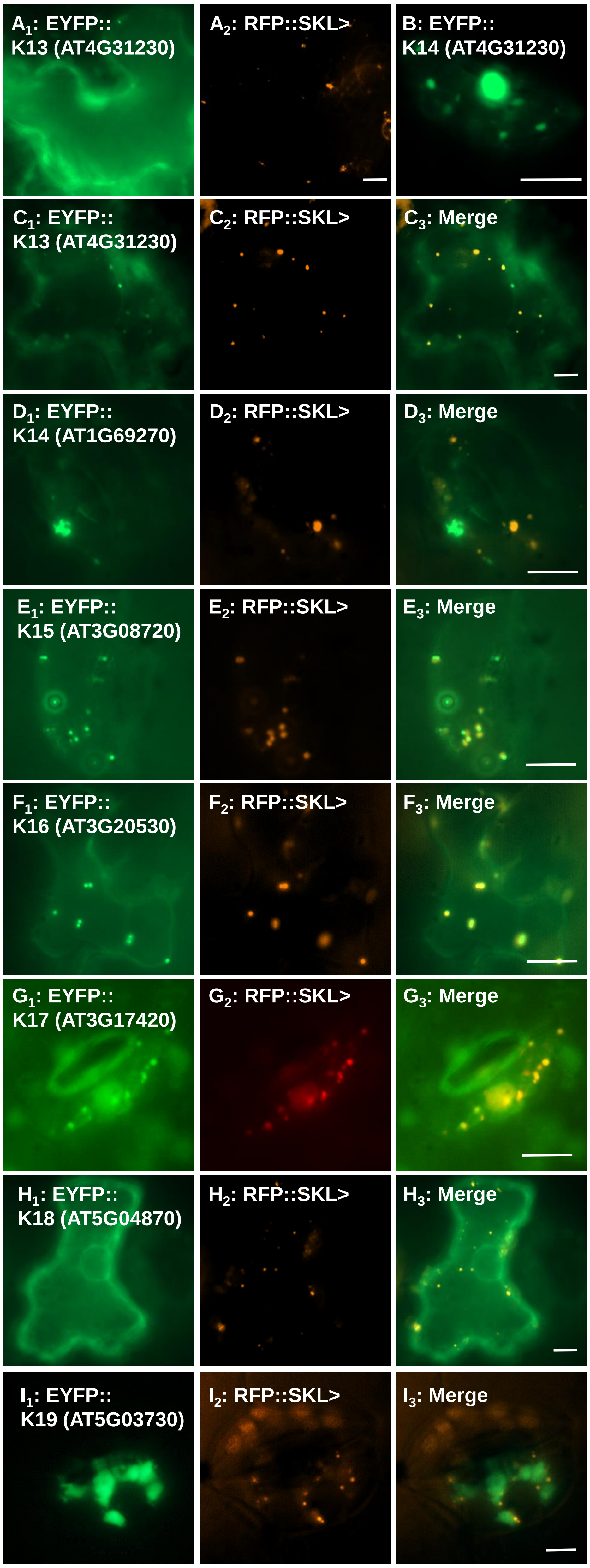

Supplemental Figure 6. Peroxisome targeting validation for previously reported peroxisomal protein kinases by in vivo subcellular targeting analysis in tobacco. *Nicotiana tabacum* cells were transformed biolistically with EYFP fusion constructs that were C-terminally fused with Arabidopsis full-length cDNAs. These proteins were previously shown to have functional PTS1s and/or detected in isolated peroxisome proteomes (see Table 1 for more details) and their full-length peroxisomal targeting was not previously validated. Interestingly, the full-length fusion K13 construct some cells remained in the cytosol (A) and also targeted to punctate structures in other cells (B), which were confirmed to be peroxisomes (C). K14 did not target to peroxisomes (D), and K15 targeted structures found to coincide in peroxisomes (E). Also, K16 proved to localize in peroxisomes (F). K17 did target structures (G), which proved to coincide with RFP::SKL> in peroxisomes (Figure 5E). In addition, K18 (CPK1) failed to target to peroxisomes when fused with a free c-terminus (H). Representative images (produced by fluorescence microscopy) are shown. Scale bars 5 µm.

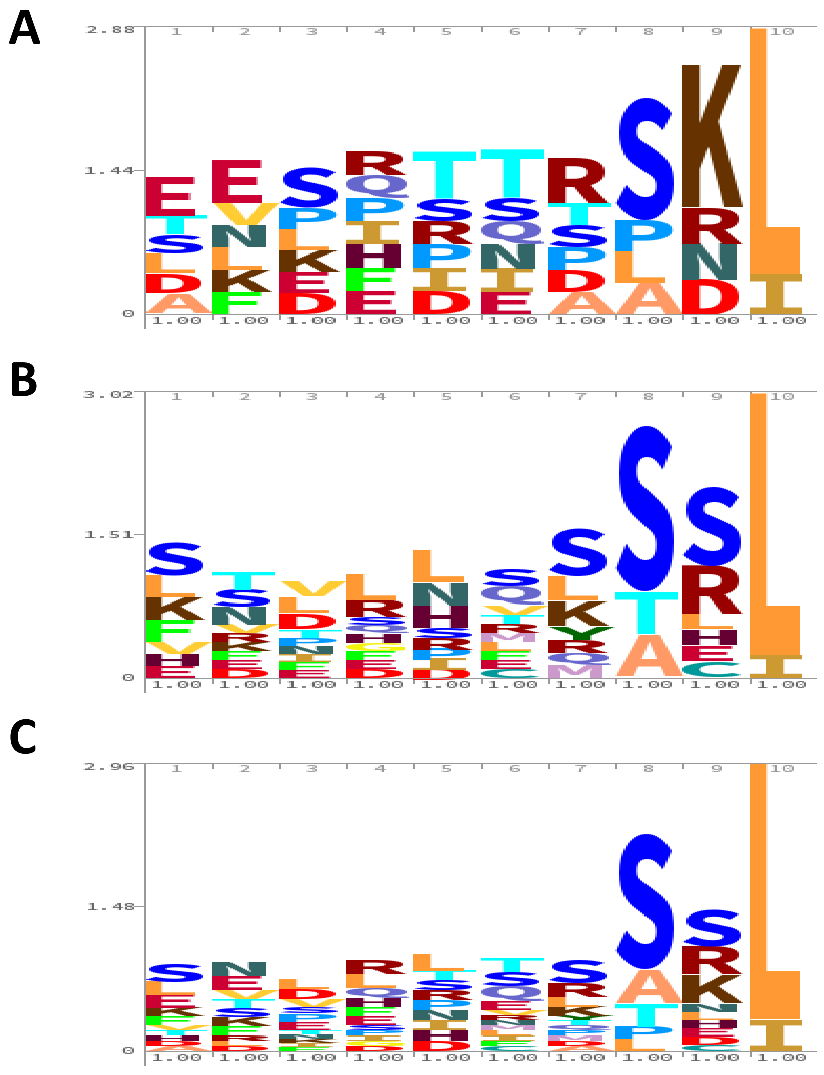

Supplemental Figure 8. Logo plots for the peroxisomal targeting domain (PTD) sequences of protein kinases studied here. Sequences are grouped to highlight the PTS1 tripeptides and upstream residues in (A) the group of protein kinases that were studied prior to this work, (B) the protein kinases from this study that targeted to peroxisomes and (C) the combination of the two groups (A+B). Non-canonical PTS1s are prevalent in newly studied kinases. The height of each letter represents the probability of the corresponding amino acid at each position. Logos were done using https://skylign.org/ (Wheeler et al., 2014).

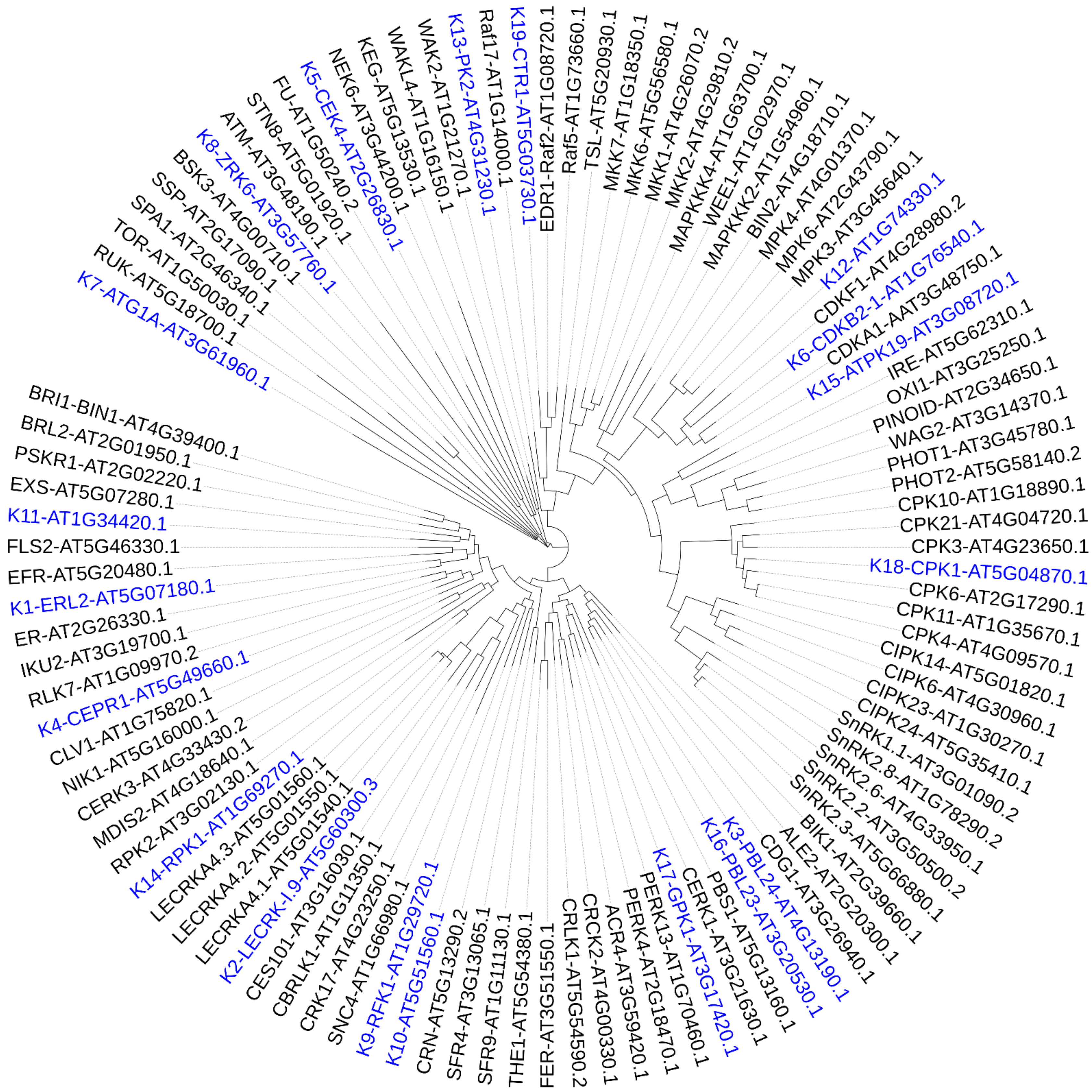

Supplemental Figure 9. Phylogenetic analysis and comparison of the peroxisomal protein kinases with other protein kinases having defined roles. Evolutionary relationship of peroxisomal protein kinase domains (in blue, Table 1) and the published Arabidopsis protein kinase domains implemented in physiological phenotypes (in black) from Zulawski et al. (2014). Protein kinase evolutionary relationship was inferred by using the Maximum Likelihood Method and JTT matrix-based model (Jones et al., 1992). Initial tree(s) for the heuristic search were obtained automatically by applying Neighbor-Join and BioNJ algorithms to a matrix of pairwise distances estimated using the JTT model, and then selecting the topology with superior log likelihood value. The tree is drawn to scale, with branch lengths measured in the number of substitutions per site. This analysis involved 105 total (amino acid) sequences. Evolutionary analyses were conducted in MEGAX (Kumar et al., 2018). The phylogenetic tree was manipulated and displayed by the online tool Interactive Tree Of Life (https://itol.embl.de) (Letunic and Bork, 2019).

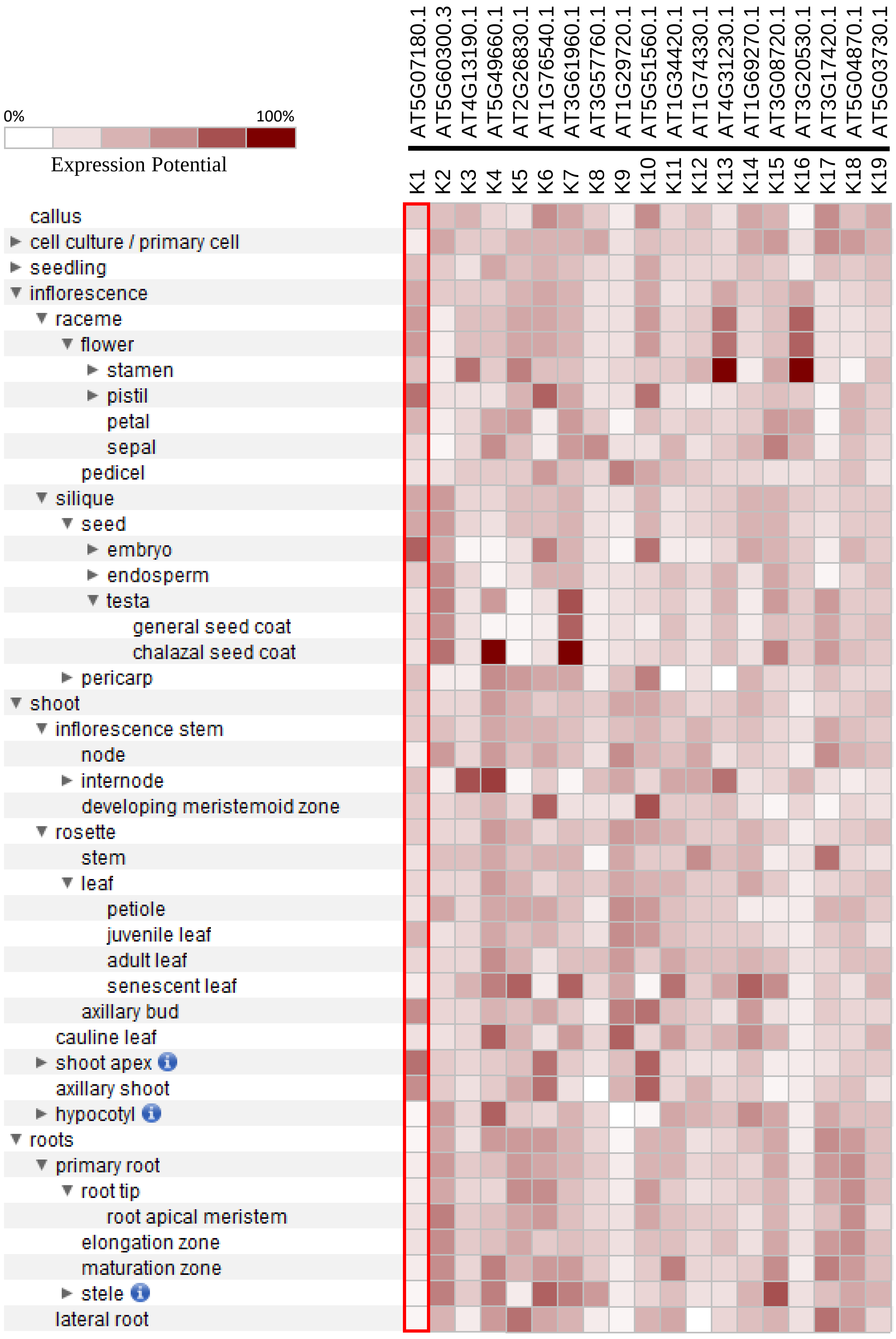

Supplemental Figure 10. Anatomical expression analysis of peroxisome targeted protein kinases in *Arabidopsis thaliana*. Expression profiles obtained from Arabidopsis microarray experiments as reported by Genevestigator (www.geneinvestigator.com; Zimmermann et al., 2004). Data are reported as heat maps with absolute expression values and shaded such that higher expression values are darker as indicated by the scale. Dataset: 127 anatomical parts from data selection: AT_AFFY_ATH1-0 and showing twenty measures.

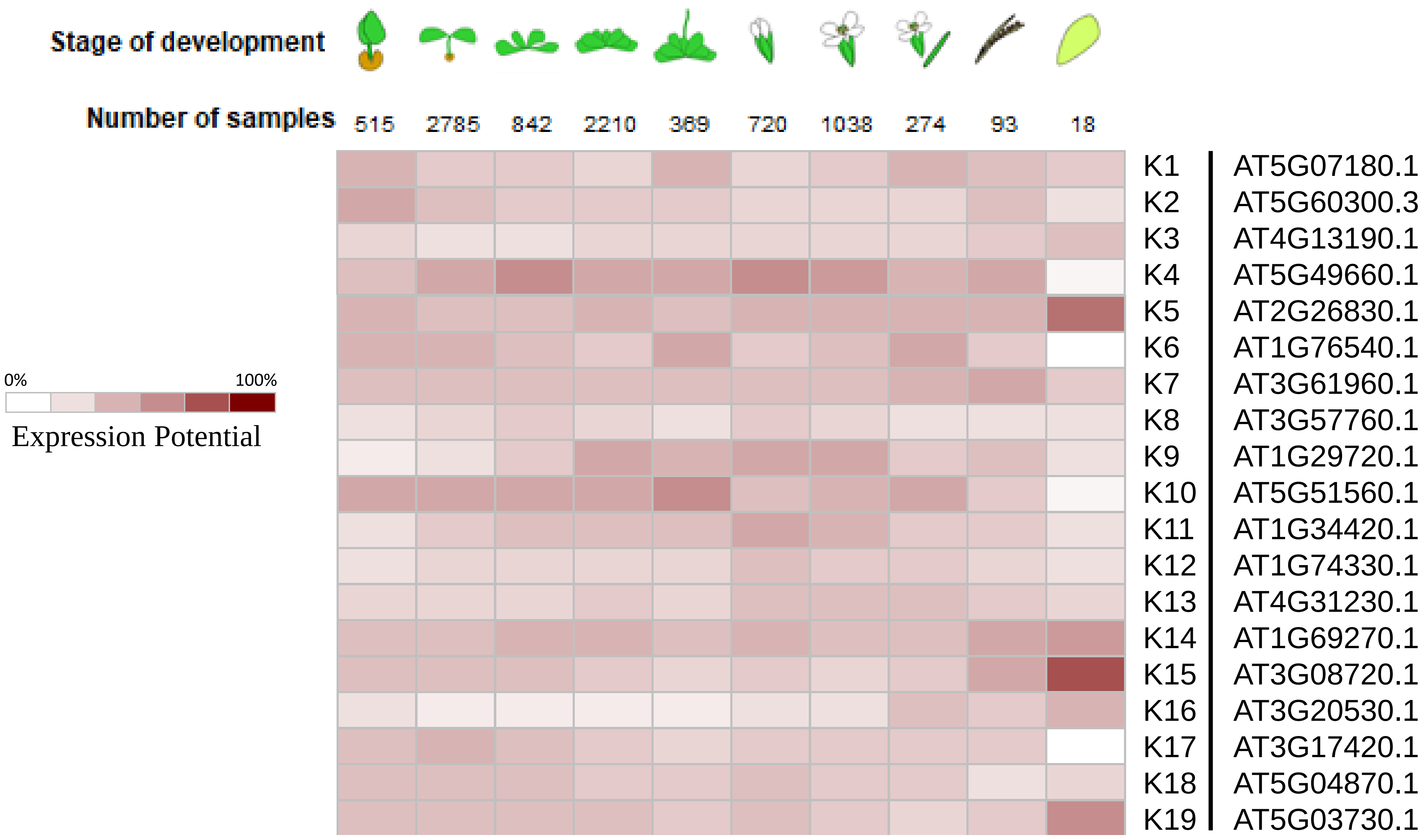

Supplemental Figure 11. Developmental expression analysis of peroxisome targeted protein kinases in *Arabidopsis thaliana*. Expression profiles obtained from Arabidopsis microarray experiments as reported by Genevestigator (www.geneinvestigator.com; Zimmermann et al., 2004). Data are reported as heat maps with absolute expression values and shaded such that higher expression values are darker as indicated by the scale. Dataset: 10 developmental stages from data selection: AT_AFFY_ATH1-0 and showing twenty measures.

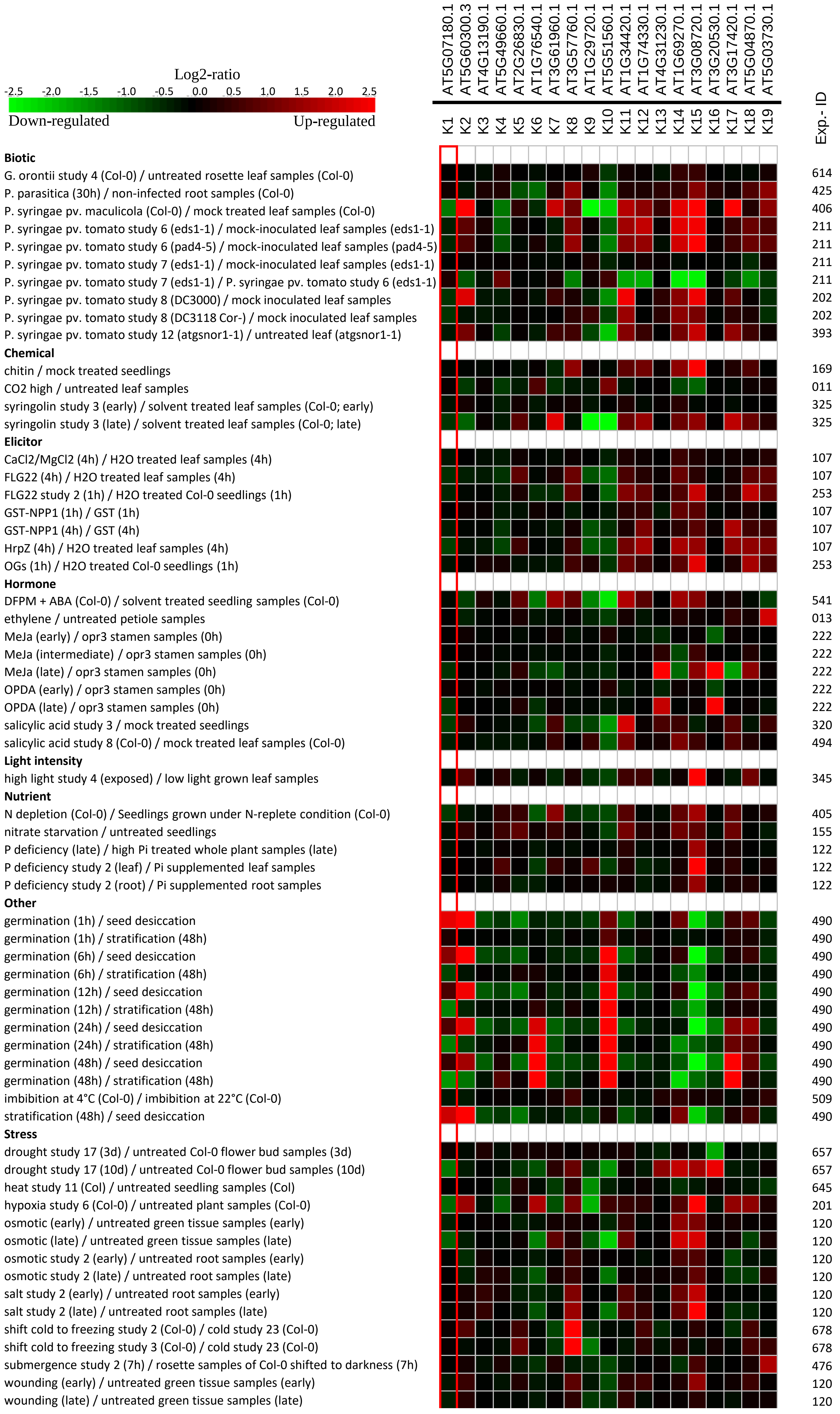

Supplemental Figure 12. Gene expression profiles of Arabidopsis peroxisome targeted protein kinases in response to various stresses/stimuli**.** The mRNA data was obtained from Arabidopsis microarray experiments collated by Genevestigator (**www.geneinvestigator.com**). Color code represents fold change in mRNA level. Exp. ID = Experiment Identifier. Dataset: 76 perturbations from data selection: AT_AFFY_ATH1-0.
