# Supplementary material for: Identification of Arabidopsis Protein Kinases That Harbor Functional Type 1 Peroxisomal Targeting Signals
Source: Front Cell Dev Biol. 2022 Feb 15;10:745883. doi: 10.3389/fcell.2022.745883 (PMC8886021; doi:10.3389/fcell.2022.745883)
Supplement: Supplementary file 4 [file DataSheet1.PDF]

Supplemental Figure 1. Multiple sequence alignments and phylograms for protein kinases harboring PTS1-like sequence conservation.

The full-length kinase sequences extracted from TAIR were subjected to protein BLAST and the orthologs harboring PTS1 were extracted and used for alignments. The C-terminal PTS1 tripeptides are highlighted in bold red in each alignment and on phylograms (see attached table of contents and attached linked pages). AlignX (Vector NTI Advance, Invitrogen) was used to conduct and manage multiple sequence alignment projects based on the Clustal W algorithm (Nucleic Acid Research, 22 (22): 4673-4680, 1994). Color background: yellow, identical aa; blue, conservative aa; white, weakly similar aa; green, block of similar aa. The phylograms were generated by the AlignX program (Vector NTI Advance, Invitrogen) using the Neighbor Joining method (NJ) (Saitou and Nei, 1987). These distances are related to the degree of divergence between the sequences. PTD: Peroxisomal targeting domains

Contents

PTD1_AT5G07180.1 (Verified in this study as peroxisomal) 2

PTD2_AT5G60300.3 (Verified in this study as peroxisomal) 7

PTD3_ AT4G13190.1 (Verified in this study as peroxisomal) 8

PTD4_ AT5G49660.1 (Verified in this study as peroxisomal) 11

PTD5_ AT2G26830.1 (Verified in this study as peroxisomal) 14

PTD6_ AT1G76540.1 (Verified in this study as peroxisomal) 15

PTD7_ AT3G61960.1 (Verified in this study as peroxisomal) 18

PTD8_ AT3G57760.1 (Verified in this study as peroxisomal) 20

PTD9_AT1G29720.1 (Verified in this study as peroxisomal) 21

PTD10_ AT5G51560.1 (Verified in this study as peroxisomal) 26

PTD11_ AT1G34420.1 (Verified in this study as peroxisomal) 31

PTD15_AT3G24790.1 (Appeared in this study in Cytosol).......... 34

PTD23_ AT1G20930.1 (Appeared in this study in Cytosol).......... 38

PTD24_ AT1G66880.1 (Appeared in this study in Cytosol).......... 41

PTD26_ AT4G18950.1 (Appeared in this study in Cytosol).......... 44

PTD1_AT5G07180.1 (Verified in this study as peroxisomal)

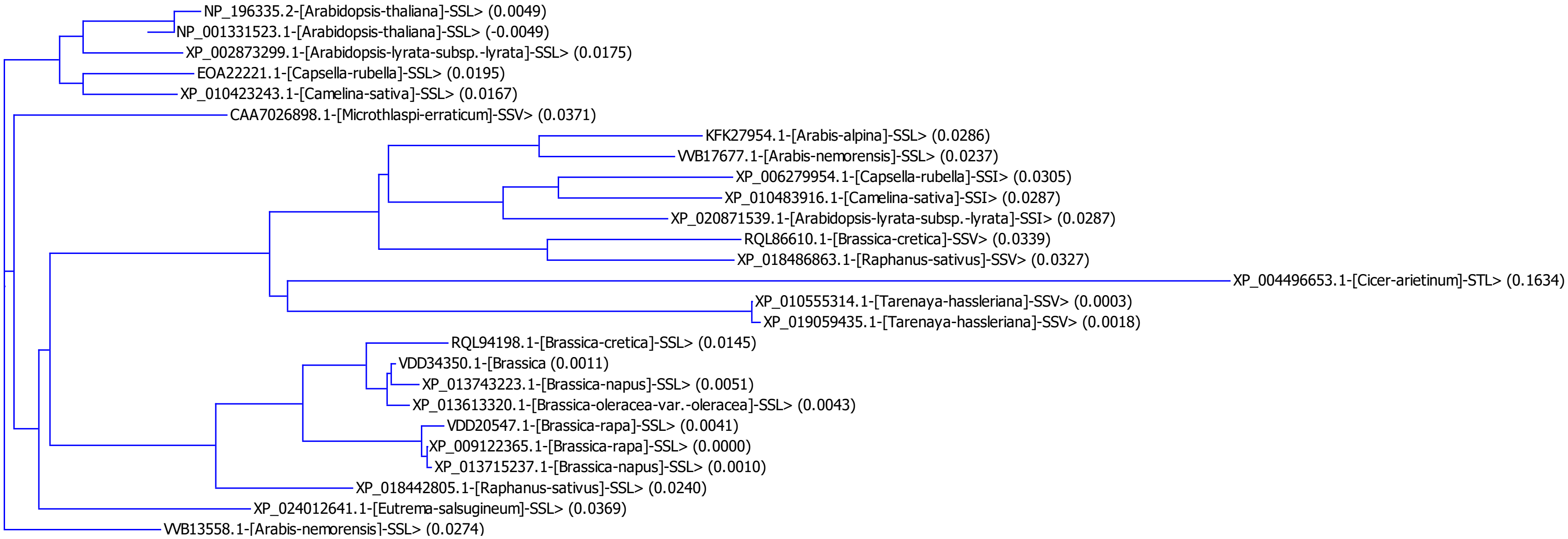


```
                                                            1                                                                                                  100
             NP_196335.2-[Arabidopsis-thaliana]-SSL>   (1) ------------MRRIETMKGLFFCLGMVVFMLLG-SVSPMNN-EGKALMAIKASFSNVANMLLDWDDVHNHDFCSWRGVFCDNVSL--NVVSLNLSNLN
          NP_001331523.1-[Arabidopsis-thaliana]-SSL>   (1) ----------------------------------------------------------------------------------------------------
XP_002873299.1-[Arabidopsis-lyrata-subsp.-lyrata]-...  (1) --------------MKVELKGLVFGLVMVVFMLLG-FVSPMNNNEGKALMAIKASFSNVANMLLDWGDVHNNDFCSWRGVFCDNVSL--TVVSLNLSNLN
                 EOA22221.1-[Capsella-rubella]-SSL>    (1) ------------MSRIEKMKGLGFRLGMVVFMLLG-VVSPMNN-QGKALMAIKASFSNVANMLLDWDDVHNNDFCSWRGVFCDNVSL--SVVSLNLSNLN
              XP_010423243.1-[Camelina-sativa]-SSL>    (1) ------------MIRIEKMKGLVFGLGMVVFMLIG-AVSPMNN-EGKALMAIKASFSNVANMLLDWDDVHNNDFCSWRGVSCDNLTL--SVVSLNLSTLN
        CAA7026898.1-[Microthlaspi-erraticum]-SSV>     (1) ------------------MKGLVLCLGLLVFMLTG-FVSPMNN-EGKALMEIKASFSNVANMLLDWDDVHNNDFCSWKGVLCDNVSL--SVVSLNLSSLN
                    KFK27954.1-[Arabis-alpina]-SSL>    (1) --------------MKKKMQRMCLCLAMVVFMLLG-SASPLND-EGKALMEIKGSFSNLVNMLLDWDDVHSNDFCSWRGVFCDNFSS--SVVSLNLSNLN
                VVB17677.1-[Arabis-nemorensis]-SSL>    (1) ---------------------------MVVFMLLG-PASPLND-EGKALIEIKGSFSNLVNMLLDWDDVHSNEFCSWRGVFCDNVSS--SVVSLNLSNLN
             XP_006279954.1-[Capsella-rubella]-SSI>    (1) -------------MKEKKMQRVVLCLAVVLSMLLG-VASSMNN-EGKALMAIKGSFSNLVNMLLDWDDVHNTDFCSWRGVFCDNVSF--SVVSLNLSNLN
              XP_010483916.1-[Camelina-sativa]-SSI>    (1) ----------------MKMQRVVLCLAVVVSMLLG-VASSMNN-EGKALMAIKGSFSNLVNMLLDWDDVHNTDFCSWRGVFCDNVTF--SVVSLNLSNLN
XP_020871539.1-[Arabidopsis-lyrata-subsp.-lyrata]-...  (1) --------------MKEKMQRMVLCLAMVVFLLLG-VASSINN-EGKALMAIKGSFSNLVNMLLDWDDVHNSDFCSWRGVYCDIVTF--SVVSLNLSSLN
                 RQL86610.1-[Brassica-cretica]-SSV>    (1) --------------------MKEMMELMVVFMLLLGVASPMNN-EGTALMAIKGSFSNVVNMLLDWDDVHNSDFCSWRGVVCDNVSF--SVVTLNLSNLN
            XP_018486863.1-[Raphanus-sativus]-SSV>     (1) ------------------MKEKKMQLMVVFILLLG-VASPMND-EGKALMEIKGSFSNVVNMLLDWDDVHNNDYCSWRGVYCDNASF--SVVSLNLSNLN
             XP_004496653.1-[Cicer-arietinum]-STL>     (1) MEENGLVLLFYVGQHNLRFLMFPVTLTLLMLLVFSPFASPLSE-EGQALMAMKSSFNNIADVLLDWDDVHNDDLCSWRGVFCDNVTLTQTVVSLNLSSLN
        XP_010555314.1-[Tarenaya-hassleriana]-SSV>     (1) --------------MKVRVKGWVLSLAMAVFVLVG-VASSIND-EGKALMAIKASFSNVANVLLDWDDVHNDDFCSWRGVYCDNISL--SVVSLNLSNLN
        XP_019059435.1-[Tarenaya-hassleriana]-SSV>     (1) --------------MKVRVKGWVLSLAMAVFVLVG-VASSIND-EGKALMAIKASFSNVANVLLDWDDVHNDDFCSWRGVYCDNISL--SVVSLNLSNLN
                 RQL94198.1-[Brassica-cretica]-SSL>    (1) ---------------------MEKVKSLALFFMLCGVVSPMND-EGKALTEMKASFSNVANMLLDWDDVHNSDFCSWRGVLCDNVSL--SVVSLNLSNLN
                              VDD34350.1-[Brassica     (1) ---------------------MEKVKSLALFFMLCGVVSPMND-EGRALMEMKASFSNVANMLLDWDDVHNSDFCSWRGVLCDNVSL--SVVSLNLSNLN
               XP_013743223.1-[Brassica-napus]-SSL>    (1) ---------------------MEKVKSLALFFMLCGVVSPMND-EGRALMEMKASFSNVANMLLDWDDVHNSDFCSWRGVLCDNVSL--SVVSLNLSNLN
XP_013613320.1-[Brassica-oleracea-var.-oleracea]-S...  (1) ---------------------MEKVKSLALFFMLCGVVSPMND-EGRALMEMKASFSNVANMLLNWDDVHNSDFCSWRGVLCDNVSL--SVVSLNLSNLN
                   VDD20547.1-[Brassica-rapa]-SSL>     (1) ------------------MEKVKSLGMVVVFFMLCGVVSPMND-EGKALMEMKASFSNVANMLLDWDDVHNSDFCSWRGVLCDNVSL--SVVSLNLSNLN
               XP_009122365.1-[Brassica-rapa]-SSL>     (1) ------------------MEKVKSLGMVVVFFMLCGVVSPMND-EGKALMEMKASFSNVANMLLDWDDVHNSDFCSWRGVLCDNVSL--SVVSLNLSNLN
              XP_013715237.1-[Brassica-napus]-SSL>     (1) ------------------MEKVKSLGMVVVFFMLCGVVSPMND-EGKALMEMKASFSNVANMLLDWDDVHNSDFCSWRGVLCDNVSL--SVVSLNLSNLN
            XP_018442805.1-[Raphanus-sativus]-SSL>     (1) ---------------MGKMKSLALCLGMVVFFMLCRVVSPMIN-EGKALMEMKASFSNVANMLLDWDDVHNSDFCSWRGVLCDNLSL--SVVSLNLSNLN
          XP_024012641.1-[Eutrema-salsugineum]-SSL>    (1) ------------------MKGLALCLGMVVFTFLG-FVSPINN-EGKALMAIKASFSNVANMLLDWDNVHNNDFCSWRGVICDNVSF--SVVSLNLSNLN
                VVB13558.1-[Arabis-nemorensis]-SSL>    (1) ---------------------------MVVFMLIG-VVSPMNN-EGKALMAIKASFSNVANMLLDWDNVHN-DFCSWRGVFCDNISL--SVVSLNLSNLN
                                                           101                                                                                                200
```

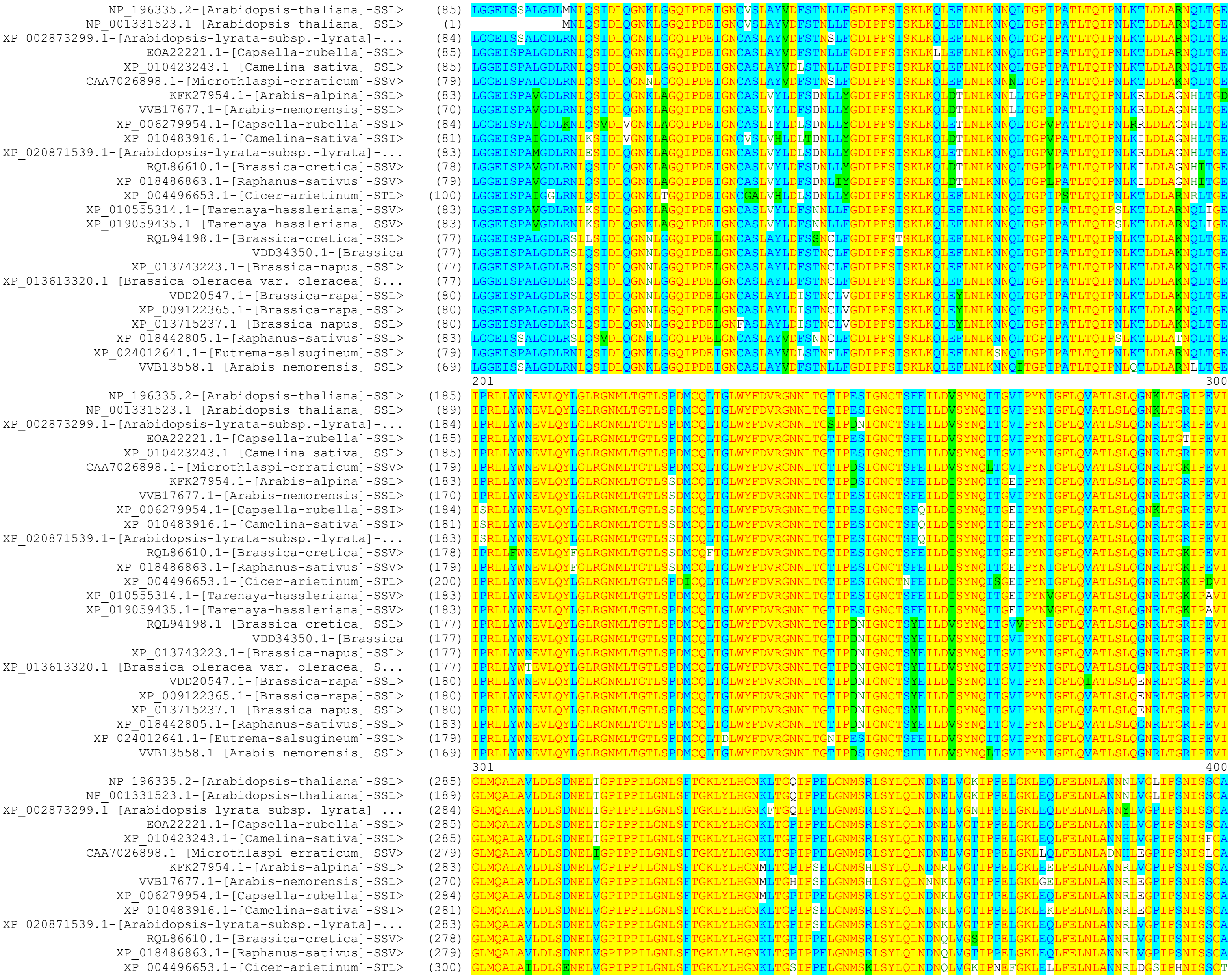

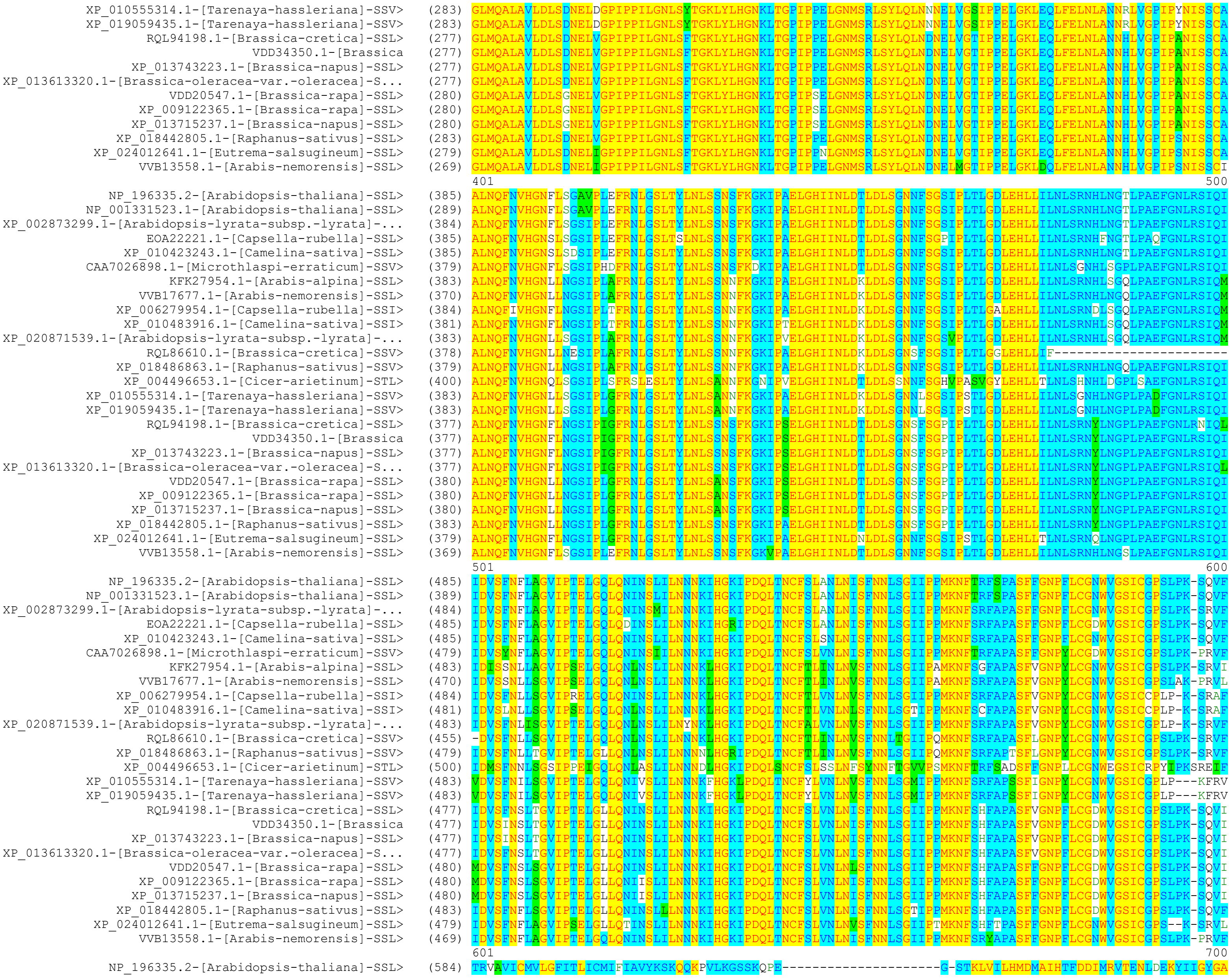

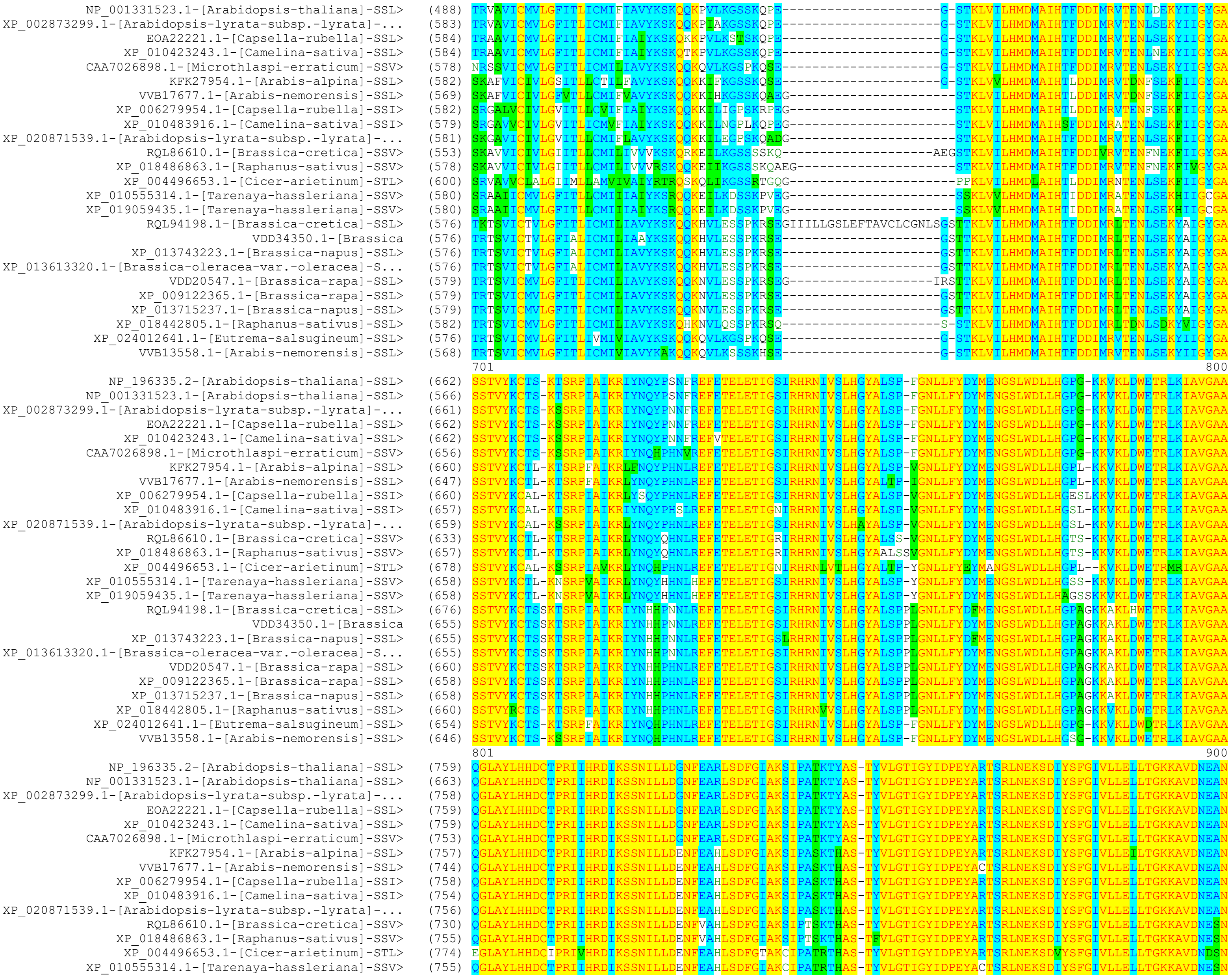

```
              XP_019059435.1-[Tarenaya-hassleriana]-SSV>   (756) QGLAYLHHDCTPRIIHRDIKSSNILLDENFEAHLSDFGIAKCIPATRTHAS-TYVLGTIGYIDPEYACTSRLNEKSDIYSFGIVLLELLTGKKAVDNESN
                      RQL94198.1-[Brassica-cretica]-SSL>   (776) QGLAYLHHDCTPRIIHRDVKSSNILLDGNFEARLSDFGIAKSIAAAKAYASSTYVLGTIGYIDPEYARTSRLNEKSDVYSFGVVLLELLTGKKAVDDEAN
                                 VDD34350.1-[Brassica      (755) QGLAYLHHDCTPRIIHRDVKSSNILLDGNFEARLSDFGIAKSIAAAKAYASSTYVLGTIGYIDPEYARTSRLNEKSDVYSFGVVLLELLTGKKAVDDEAN
                   XP_013743223.1-[Brassica-napus]-SSL>   (755) HGLAYLHHDCTPRIIHRDVKSSNILLDGNFEARLSDFGIAKSIAAAKAYASSTYVLGTIGYIDPEYARTTRLNEKSDVYSFGVVLLELLTGKKAVDDEAN
XP_013613320.1-[Brassica-oleracea-var.-oleracea]-S...   (755) QGLAYLHHDCTPRIIHRDVKSSNILLDGNFEGRLSDFGIAKSIAAAKAYASSTYVLGTIGYIDPEYARTSRLNEKSDVYSFGVVLLELLTGKKAVDDEAN
                         VDD20547.1-[Brassica-rapa]-SSL>   (760) QGLAYLHHDCTPRIIHRDVKSSNILLDGNFEARLSDFGIAKSIAAAKAYAS-TYVLGTIGYIDPEYARTSRLNEKSDVYSFGVVLLELLTGKKAVDNEAN
                    XP_009122365.1-[Brassica-rapa]-SSL>   (758) QGLAYLHHDCTPRIIHRDVKSSNILLDGNFEARLSDFGIAKSIAAAKAYAS-TYVLGTIGYIDPEYARTSRLNEKSDVYSFGVVLLELLTGKKAVDNEAN
                   XP_013715237.1-[Brassica-napus]-SSL>   (758) QGLAYLHHDCTPRIIHRDVKSSNILLDGNFEARLSDFGIAKSIAAAKAYAS-TYVLGTIGYIDPEYARTSRLNEKSDVYSFGVVLLELLTGKKAVDNEAN
               XP_018442805.1-[Raphanus-sativus]-SSL>   (759) QGLAYLHHDCTPRIIHRDVKSSNILLDGNFEARLSDFGIAKSIPAAKTYAS-TYVLGTIGYIDPEYARTSRLNEKSDVYSFGVVLLELLTGKKAVDNEAN
            XP_024012641.1-[Eutrema-salsugineum]-SSL>   (751) QGLAYLHHDCTPRIIHRDIKSSNILLDGNFEARLSDFGIAKSIPASKTYAS-TYVLGTIGYIDPEYARTSRLNEKSDVYSFGIVLLELLTGKKAVDNEAN
                  VVB13558.1-[Arabis-nemorensis]-SSL>   (743) QGLAYLHHDCTPRIIHRDIKSSNILLDGNFEARLSDFGIAKSIPATKTYAS-TYVLGTIGYIDPEYARTSRLNEKSDIYSFGIVLLELLTGKKAVDNEAN
                                                              901                                                                                              1000
                NP_196335.2-[Arabidopsis-thaliana]-SSL>   (858) LHQMILSKADDNTVMEAVDAEVSVTCMDSGHIKKTFQLALLCTKRNPLERPTMQEVSRVLLSLVP---------------SPPPKKLPSPAKVQ------
             NP_001331523.1-[Arabidopsis-thaliana]-SSL>   (762) LHQMILSKADDNTVMEAVDAEVSVTCMDSGHIKKTFQLALLCTKRNPLERPTMQEVSRVLLSLVP---------------SPPPKKLPSPAKVQ------
XP_002873299.1-[Arabidopsis-lyrata-subsp.-lyrata]-...   (857) LHQMILSKADDNTVMEAVDAEVSVTCMDSGHIKKTFQLALLCTKRNPLERPTMQEVSRVLLSLLP---------------SPPPKKLPSPAKLQ------
                   EOA22221.1-[Capsella-rubella]-SSL>   (858) LHQMILSKADDNTVMEAVDAEVSVTCVDSGHIKKTFQLALLCTKRNPLERPTMQEVSRVLLSLLP---------------SPPPKKLQSPAKLP------
              XP_010423243.1-[Camelina-sativa]-SSL>   (858) LHQMILSKADDNTVMEAVDAEVSVTCVDSGHIKKTFQLALLCTKRNPLERPTMQEVARVLLSLFP---------------SPPPKKLPSPARLQ------
          CAA7026898.1-[Microthlaspi-erraticum]-SSV>   (852) LHQMILSKADDNTVMEAVDAEVSVTCVDSGHIRKTFQLALMCTKRNPLERPTMQEVSRVLLSLLP---------------SPPPKKLPSPLKLQ------
                    KFK27954.1-[Arabis-alpina]-SSL>   (856) LHQLIMSKTDDNTVMEAVDPEVTVTCMDLGHIKKTFKLALLCTKRNPLERPTMLEVSRVLLSLVPSLQVAKKLP-----SLDPSTKKPIDYVVG------
                VVB17677.1-[Arabis-nemorensis]-SSL>   (843) LHQLILSKTDDNTVMEAVDPEVTVTCMDLGHIRKTFKLALLCTKRNPLERPTMLEVSRVLLSLLPSLQVAKKLP-----SLDPSTKKPIDYGVGX-----
               XP_006279954.1-[Capsella-rubella]-SSI>   (857) LHQLILSKADDNTVMEAVDPEVTVTCVDLGHIRKTFQLALLCTKRNPLERPTMLEVSRVLLSLLPS-------L-----QVAKKLQEPSLDKSTK-----
              XP_010483916.1-[Camelina-sativa]-SSI>   (853) LHQLILSKADDNTVMEAVDPEVTVTCMDLGHIRKTFQLALLCTKRNPLERPTMLEVSRVLLSLLPS--------------LQVAKKLPSLDPSTK-----
XP_020871539.1-[Arabidopsis-lyrata-subsp.-lyrata]-...   (855) LHQLILSKADDNTVMEAVDPEVTVTCMDLGHIRKTFQLALLCTKRNPLERPTMLEVSRVLLSLLPS--------------LQVAKKLPSHDQSTK-----
                  RQL86610.1-[Brassica-cretica]-SSV>   (829) LHQLILSKADDNTVMEAVDPEVTVTCMDVGHIRKTFKLALLCTKRNPLERPTMLEVSRVLLSLLPS--------------MQVAKKLPSPDPSKKPT--V
               XP_018486863.1-[Raphanus-sativus]-SSV>   (854) LHQLILSKADDNTVMEAVDPEVTVTCMDLGHIRKTFKLALLCTKRNPLERPTMLEVSRVLISLLPSMQAAKKLS-----SSPDPSKKATDYGVR------
              XP_004496653.1-[Cicer-arietinum]-STL>   (873) LHHLILSKAENNTIMEAVDPEVSITCIDLAHVKKTFRLALLCTKKNPSERPTMHEVARVLISQLP---------------APPSKVVAAAAKSFDYN---
          XP_010555314.1-[Tarenaya-hassleriana]-SSV>   (854) LHQLILSRADDNTVMEAVDPEVSVTCMDLGHIRKTFQLALLCTKRNPSERPTMHEVSRVLHSLLHPATALKKLSPVDYAQFFVEKKQHGVRQLQEEQQEV
          XP_019059435.1-[Tarenaya-hassleriana]-SSV>   (855) LHQLILSRADDNTVMEAVDPEVSVTCMDLGHIRKTFQLALLCTKRNPSERPTMHEVSRVLHSLLHPATALKKLSPVDYAQFFVEKKQHGVRQLQEEQQEV
                      RQL94198.1-[Brassica-cretica]-SSL>   (876) LHQLILSKADDNTVMDAVDAEVSVTCVDSGHIKKTFQLALLCTKRNPMERPTMQEVARVLLSLLLS---------------PPSKKPPSPQGME------
                                 VDD34350.1-[Brassica      (855) LHQLILSKADDNTVMDAVDAEVSVTCVDSGHIKKTFQLALLCTKRNPMERPTMQEVARVLLSLLP---------------SPPPKKPPSPAKLQ------
                   XP_013743223.1-[Brassica-napus]-SSL>   (855) LHQLILSKADDNTVMDAVDPEVSVTCVDSGHIKKTFQLALLCTKRNPMERPTMQEVARVLLSLLP---------------SPPPKKPPSPAKLQ------
XP_013613320.1-[Brassica-oleracea-var.-oleracea]-S...   (855) LHQLILSKADDNTVMDAVDAEVSVTCVDSGHIKKTFQLALLCTKRNPMERPTMQEVARVLLSLLP---------------SPPPKKPPSPAKLQ------
                         VDD20547.1-[Brassica-rapa]-SSL>   (859) LHQLILSKADDNTVMDAVDAEVSVTCVDSGHIKKTFQLALMCAKRNPMERPTMQEVARVLLSLLP---------------SPPPKKPPSPQGVE------
                    XP_009122365.1-[Brassica-rapa]-SSL>   (857) LHQLILSKADDNTVMDAVDAEVSVTCVDSGHIKKTFQLALMCTKRNPMERPTMQEVARVLLSLLP---------------SPPPKKPPSPQGVE------
                   XP_013715237.1-[Brassica-napus]-SSL>   (857) LHQLILSKADDNTVMDAVDAEVSVTCVDSGHIKKTFQLALMCTKRNPMERPTMQEVARVLLSLLP---------------SPPPKKPPSPQGVE------
               XP_018442805.1-[Raphanus-sativus]-SSL>   (858) LHQMILSKADDNTVMDAVDAEVSVTCVDSGHIKKTFQLALLCTKRNPMERPTMQEVARVLLSLLP---------------SPPPKKPPSPAKLQ------
            XP_024012641.1-[Eutrema-salsugineum]-SSL>   (850) LHHMILSKADDNTVMEAVDAEVSVTCVDSGHIKKTFQLALLCTKQNPLERPTMQEVSRVLLSLLP---------------SPPSKKLTSPAKLQ------
                  VVB13558.1-[Arabis-nemorensis]-SSL>   (842) LHQMILSKTDDNTVMEAVDGEVSVTCVDSGHIKKTFQLALLCTKRNPLERPTMQEVSRVLLSLLPFS-------------STPPKKLPSPVKLQG-----
                                                              1001                            1033
                NP_196335.2-[Arabidopsis-thaliana]-SSL>   (937) --EGEERRESHSSDTTTPQWFVQFREDISKSSL
             NP_001331523.1-[Arabidopsis-thaliana]-SSL>   (841) --EGEERRESHSSDTTTPQWFVQFREDISKSSL
XP_002873299.1-[Arabidopsis-lyrata-subsp.-lyrata]-...   (936) --EGEERRECHSSDTTTPQWFVQFREDISKSSL
                   EOA22221.1-[Capsella-rubella]-SSL>   (937) --EAEERRESHSSDTATPQWFVQFREDISKSSL
              XP_010423243.1-[Camelina-sativa]-SSL>   (937) --EGEERRESHSSDTTTPQWFVQFREDISKSSL
          CAA7026898.1-[Microthlaspi-erraticum]-SSV>   (931) --EVEDRRESHSSDTTSPEWFVQFREAISKSSV
                    KFK27954.1-[Arabis-alpina]-SSL>   (945) --QQQEKSEAQDREAEASQWFVQFHEDISKSSL
                VVB17677.1-[Arabis-nemorensis]-SSL>   (933) ------XXXXXXXXXXXSQWFVQFRENISRSSL
               XP_006279954.1-[Capsella-rubella]-SSI>   (940) --KPQQESEVRNREAEASEWFVQFREVISKSSI
              XP_010483916.1-[Camelina-sativa]-SSI>   (934) --TPQHESEVRNREAEASQWFVQFREVISNSSI
XP_020871539.1-[Arabidopsis-lyrata-subsp.-lyrata]-...   (936) --KPQQENEVRNHDAEASQWFVQFREVISKSSI
                  RQL86610.1-[Brassica-cretica]-SSV>   (913) YGVRDQQKQQQERSEEGSQWFDQFHEVISKSSV
               XP_018486863.1-[Raphanus-sativus]-SSV>   (943) ------DQQQHSRSEEGSQWFDQFHEVISKSSV
              XP_004496653.1-[Cicer-arietinum]-STL>   (955) -QKVDGLQPQPDNNASNAQWFVRFGDDISKSTL
          XP_010555314.1-[Tarenaya-hassleriana]-SSV>   (954) QRLRENHSSDAAAAEAPSQWFDQFREVISKSSV
          XP_019059435.1-[Tarenaya-hassleriana]-SSV>   (955) QRLRENHSSDAAAAEAPSQWFDQFREVISKSSV
                      RQL94198.1-[Brassica-cretica]-SSL>   (955) -----ERRESHSSDTTSPQWFVQFREAISKSSL
                                 VDD34350.1-[Brassica      (934) --GVEERRESQSSDTTSPQWFVQFREAISKSSL
                   XP_013743223.1-[Brassica-napus]-SSL>   (934) --GVEERRESQSSDTTSPQWFVQFREAISKSSL
XP_013613320.1-[Brassica-oleracea-var.-oleracea]-S...   (934) --GVEERRESQSSDTTSPQWFVQFREAISKSSL
                         VDD20547.1-[Brassica-rapa]-SSL>   (938) -----ERRESHSLDTASPQWFVQFREAISKSSL
                    XP_009122365.1-[Brassica-rapa]-SSL>   (936) -----ERRESHSLDTASPQWFVQFREAISKSSL
                   XP_013715237.1-[Brassica-napus]-SSL>   (936) -----ERRESHSLDTASPQWFVQFREAISKSSL
               XP_018442805.1-[Raphanus-sativus]-SSL>   (937) --GVEERRESHSSETTSPQWFVQFREAISKSSL
            XP_024012641.1-[Eutrema-salsugineum]-SSL>   (929) --GGEERRESHSSDTTSPQWFVQFREVISKSSL
                  VVB13558.1-[Arabis-nemorensis]-SSL>   (924) --EDEPPRESHSSDKTTPQWVVQFREVISKSSL
```

PTD2_AT5G60300.3 (Verified in this study as peroxisomal)

```
                                                         1                                                                                                  100
NP_001078773.1-[Arabidopsis-thaliana]-ARL>      (1)   ----------------------------------------------------------------------------------------------------
          KFK27565.1-[Arabis-alpina]-QRL>       (1)   MAQGLDLICMVIFFLFLIHPSSQQVTGFVFNGFRQGDLHVDGVAQILPGGLLQLTNTSEQKMGHAFFKQPFEFNLSGSLTFSTHFVCAMVRQRGVTGGNG
      XP_023920694.1-[Quercus-suber]-ASL>       (1)   ----------------------------------------------------------------------------------------------------
                                                         101                                                                                                200
NP_001078773.1-[Arabidopsis-thaliana]-ARL>      (1)   ----------------------------------------------------------------------------------------------------
          KFK27565.1-[Arabis-alpina]-QRL>     (101)   IAFFLSPTMDLSNADATQYFGLFNTTTNRSPSSHIFAIELDTVQSAEFDDIDNNHVGIDVNSLTSIESAPASYFSDKEGLNKSITLLSGDSIQVWIDYDG
      XP_023920694.1-[Quercus-suber]-ASL>       (1)   ----------------------------------------------------------------------------------------------------
                                                         201                                                                                                300
NP_001078773.1-[Arabidopsis-thaliana]-ARL>      (1)   ----------------------------------------------------------------------------------------------------
          KFK27565.1-[Arabis-alpina]-QRL>     (201)   AVLNVSLAPLGIQKPSRSLLSRSINLSEVIQDRMFVGFSAATGQLANNHYILGWSFSRSKALLQNLDISKLPQVPRPPKPSKKPPLLLILLLVLLGIILL
      XP_023920694.1-[Quercus-suber]-ASL>       (1)   ----------------------------------------------------------------------------------------------------
                                                         301                                                                                                400
NP_001078773.1-[Arabidopsis-thaliana]-ARL>      (1)   ----------------------------------------------------------------------------------------------------
          KFK27565.1-[Arabis-alpina]-QRL>     (301)   ALLGGAYLYRRNKYAEVREEWEKEYGPHRYSYKSMYKATKGFHKDGFLGKGGFGEVYKGTLPQDIDIAVKRFSHDGERGMKQFVAEIASMGRLDHRNLVP
      XP_023920694.1-[Quercus-suber]-ASL>       (1)   ----------------------------------------------------------------------------------------------------
                                                         401                                                                                                500
NP_001078773.1-[Arabidopsis-thaliana]-ARL>      (1)   ----------------------------------------------------------------------------------------------------
          KFK27565.1-[Arabis-alpina]-QRL>     (401)   LLGYCRRKGEFLLVSKYMPNGSLDQFLFHNKEPSLPWSKRLPILKGIASALHYLHTGATQVVLHRDVKASNVMLDTNFIAKLGDFGMARLHDHGANPNTT
      XP_023920694.1-[Quercus-suber]-ASL>       (1)   ----------------------------------------------------------------------------------------------------
                                                         501                                                                                                600
NP_001078773.1-[Arabidopsis-thaliana]-ARL>      (1)   ----------------------------------------------------------------------------------------------------
          KFK27565.1-[Arabis-alpina]-QRL>     (501)   GAVGTVGYMAPELTSMGASTKTDVYAFGAFILEVACGRRPVEPTMEIEKQFLVEWVCDCWRKRSLLDARDPKLSGEGESSSSLESMEMVMKLGLICTNFL
      XP_023920694.1-[Quercus-suber]-ASL>       (1)   ----------------------------------------------------------------------------------------------------
                                                         601                                                                                                700
NP_001078773.1-[Arabidopsis-thaliana]-ARL>      (1)   ----------------------------------------------------------------------------------------------------
          KFK27565.1-[Arabis-alpina]-QRL>     (601)   PESRPEMEEVVRYLNGRRLLPDFSPESPGIGILSPVMVGGSSSVMASSPANETMSSPSGYTLVMLGAEVKGKVRKFCYGEASRSRSQLKHPGLCGNLRLA
      XP_023920694.1-[Quercus-suber]-ASL>       (1)   ----------------------------------------------------------------------------------------------------
                                                         701                                                                                                800
NP_001078773.1-[Arabidopsis-thaliana]-ARL>      (1)   ----------------------------------MARWLLQILIISSLHLSSVSSQQETSFVYESFLDRQNLYLDKSAIVLPSGLLQLT---NASEHQMG
          KFK27565.1-[Arabis-alpina]-QRL>     (701)   EQPDIKMFKSTQCAACKVREDLHIRSRLSDSSSAMARWLLQILIISSLHLFSLSNQQETRFVYDNFTNQENLYLDKSARILPSGLLQLT---SASEHQMG
      XP_023920694.1-[Quercus-suber]-ASL>       (1)   --------------------------------------MGTILVILYVSNIHMAFAQNENQFIYNGFLQAKLHLDGSAKITSNGLLQLTNTVNNSYPQVC
                                                         801                                                                                                900
NP_001078773.1-[Arabidopsis-thaliana]-ARL>     (64)   HAFHKKPIEFSSSGP-----LSFSTHFVCALVPKPGFEGGHGIVFVLSPSMDFTHAESTRYLGIFNASTNGSSSYHVLAVELDTIWNPDFKDIDHNHVGI
          KFK27565.1-[Arabis-alpina]-QRL>     (798)   HTFYNKPIDFSSSGS-----LSFSTHFVCALVPKPGVEGGHGIAFVISPSMDFSHAESTRYLGVFNVSSSEASSTRVLAVELDTIWNPDFKDINNNHVGI
      XP_023920694.1-[Quercus-suber]-ASL>      (63)   HALYQFPLKFNTTSSGSIPSLSFSTNFVFAIVPQVPNVGGHGMAFTISPSSNFTNVVGNQYLGLFNSSNEGLTSNHILAIELDTALNPEFGDIDRNHVGI
                                                         901                                                                                                1000
NP_001078773.1-[Arabidopsis-thaliana]-ARL>    (159)   DVNSPISVAIASASYYSDMKGSNESINLLSGNPIQVWVDYEGTLLNVSVAPLEVQKPTRPLLSHPINLTELFPNRSSLFAGFSAATGTAISDQYILWWSF
          KFK27565.1-[Arabis-alpina]-QRL>     (893)   DVNSPLSVAIASASYYSDMKGSNESINLLSGKPIQVWVDYEGTMLNVTIAPLEVQKPSRPLLSHPINISENFPNISRLFVGFSAATGTAISDQYILWWSF
      XP_023920694.1-[Quercus-suber]-ASL>     (163)   DVNSLNSTDSASAMYFSNKEGKNISLELASGNPTHLWIDYDEAEKLLSVALAPNGIPKPNQPLMSKSIDLSQYLLESMYVGFSAATGTLIPSNHYVLGWS
                                                         1001                                                                                               1100
NP_001078773.1-[Arabidopsis-thaliana]-ARL>    (259)   SIDRGSLQRLDISKLPEVPHPRAPHKKVSTLIILLPVCLAILVLAVLAGLYFRRRKYSEVSETWEKEFDAHRFSYRSLFKATKGFSKDEFLGKGGFGEV
          KFK27565.1-[Arabis-alpina]-QRL>     (993)   STNRGSLQRLDISKLPEVPHPKAPHKKLSPLVIILPVGLAVGVLGVLAGLYFRRKKYSEVSETWEKEFDAHRFSYRTLYKATKGFSKDEFLGKGGFGEV
      XP_023920694.1-[Quercus-suber]-ASL>     (263)   FNKSGQAESLDVSKLPSLPRQRKAKEIPRLMIMVFPIAVTLVLITITGAAYIIRRKKYEEIREDWEREYGPHRFIYKNLYKATKGFSDKELLGAGGFGKV
                                                         1101                                                                                               1200
NP_001078773.1-[Arabidopsis-thaliana]-ARL>    (359)   YRGNLPQG-REIAVKRVSHNGDEGVKQFVAEVVSMRCLKHRNLVPLFGYCRRKRELLLVSEYMPNGSLDEHLFDDQKPVLSWSQRLVVVKGIASALWYLH
          KFK27565.1-[Arabis-alpina]-QRL>    (1093)   YRGNLPQG-REIAVKRVSHNGDEGVKQFVAEVVSMRCLKHRNLVPLFGYCRRKRELLLVSEYMPNGSLDEHLFDDQKPVLSWPQRLVAVKGIASALCYLH
      XP_023920694.1-[Quercus-suber]-ASL>     (363)   YRGILPSSNVQIAVKRVSHNSKQGMKEFVAEIISMGRLRHRNLVQLLGYCRRRGELLLVYDYMANGSLDKFLYSNEKPNLSWFQRFRIIKGVASGLLYLH
                                                         1201                                                                                               1300
NP_001078773.1-[Arabidopsis-thaliana]-ARL>    (458)   TGADQVVLHRDVKASNIMLDAEFHGRLGDFGMARFHEHGGNAATTAAVGTVGYMAPELI-TMGASTGTDVYAFGVFMLEVTCGRRPVEPQLQVEKRHMIK
          KFK27565.1-[Arabis-alpina]-QRL>    (1192)   TGADQVVLHRDVKASNIMLDAEFNGRLGDFGMARFHDHGANAATTAAVGTIGYMAPELI-TMGASTGTDVYAFGVFMLEVTCGRRPVEPQLQVEKRHMIK
      XP_023920694.1-[Quercus-suber]-ASL>     (463)   EEWEQVVLHRDVKASNVLLDAELNGRLGDFGLARLYDHGTNPQTTHVVGTVGYLAPEIIRTRRATTCTDVFSFGAFMLEVACGRRPVGLQGLTEDIILVD
                                                         1301                                                                                               1400
NP_001078773.1-[Arabidopsis-thaliana]-ARL>    (557)   WVCECWKKDSLLDATDPRLGGKFVAEEVEMVMKLGLLCSNIVPESRPTMEQVVLYLNKNLPLPDFSPYTLGIGTFAPVLVDASSLVVSSASWSLSGPSMS
          KFK27565.1-[Arabis-alpina]-QRL>    (1291)   WVCECWKKDSLLDATDPRLEGEYIPEEVEMVMKLGLLCSNIVPESRPTMEQVVLYLNKNLPLPDFSPYTLGIGTFAPVLVDASSLVVSSASWNWSAPSMS
      XP_023920694.1-[Quercus-suber]-ASL>     (563)   WVFECWRRGAILDASDPRLEGNYVVEEIELVLKLGLLCSHSVPTARPSMRQVMQFLDGNANLPELPDGSTFFGTFKGNEEHDFLLSFPSSSGKGSAASL-
                                                         1401                                                                                               1500
NP_001078773.1-[Arabidopsis-thaliana]-ARL>    (657)   SSSPNHSPYAWQSTDQPWGQTIDTKNSLHIVAEPEKP----SPAVKMVTLPAEDPQSNHSSISSQRVQPVKREKRRLHQILVAFPWINKQYFKLGLPKHI
          KFK27565.1-[Arabis-alpina]-QRL>    (1391)   SSS-NHSPYAYQAPDQPWGQTIDTTNSLHLVAESEKPPKIVSTPVKTATLPAEDLESNLSSLSSQRL---------------------------------
      XP_023920694.1-[Quercus-suber]-ASL>     (662)   ----------------------------------------------------------------------------------------------------
                                                         1501      1514
NP_001078773.1-[Arabidopsis-thaliana]-ARL>    (753)   VHVSLFFFLQLARL
          KFK27565.1-[Arabis-alpina]-QRL>    (1457)   --------------
      XP_023920694.1-[Quercus-suber]-ASL>     (662)   --------------
```

PTD3_ AT4G13190.1 (Verified in this study as peroxisomal)

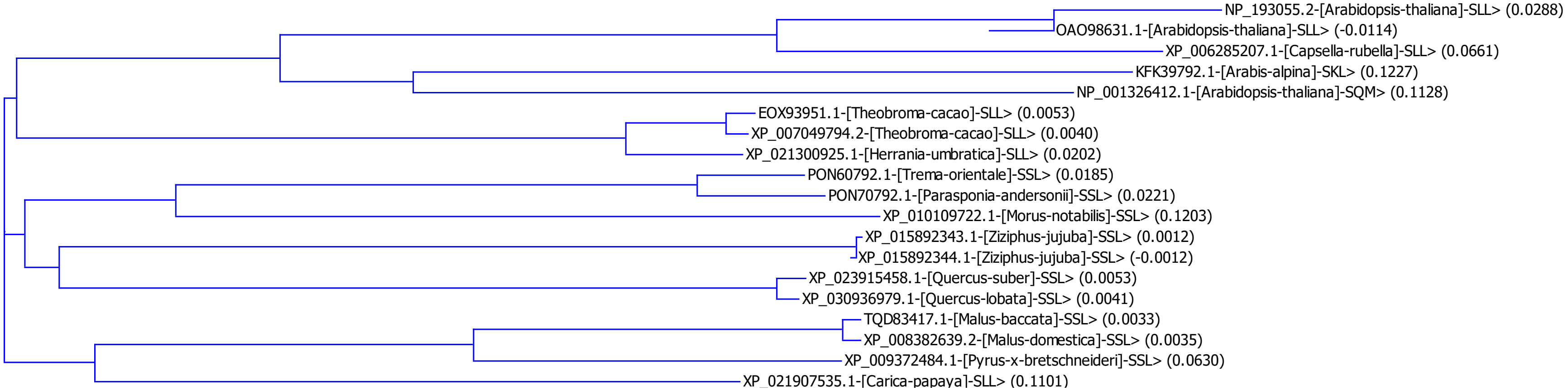


```
                                                   1                                                                                                  100
        NP_193055.2-[Arabidopsis-thaliana]-SLL>   (1) MSCFLGP---------STNNKSRENEGSSMAAPYEQQ-NLPRN-----------DRRQITTWEAVGTNKESP----------KNIKAKSFKFRELATATN
        OAO98631.1-[Arabidopsis-thaliana]-SLL>    (1) ------------------------------------------------------------------MNKESP----------KNIKAKSFKFRELATATN
     XP_006285207.1-[Capsella-rubella]-SLL>       (1) MSCFLGP---------SMNNKTRENEGSSMAAPYQQ---------------------QITTRDVVAMNKEST----------NNIEAECFTFRELAT---
            KFK39792.1-[Arabis-alpina]-SKL>       (1) MSCFS-----------CFSSTNTDNEGSSMPAPYKQP-SSP-------------KRTTGQVVANAPSNN---------------IEAETFTFRELASATK
  NP_001326412.1-[Arabidopsis-thaliana]-SQM>      (1) MSCFS-----------CFSSKVLDNEGSSMPAPYKQP-NSP-------------KRTTGEVVAKNANGPS------------NNMGARIFTFRELATATK
             EOX93951.1-[Theobroma-cacao]-SLL>    (1) MSCFSCFS-SQDKKARRLNNPSRRSQTSYYSAPPRQA-LLPRQRPENNP--KPKTSAPEPTKDTDAPKAAGN----------NNIAAQTFTFRELATATR
         XP_007049794.2-[Theobroma-cacao]-SLL>    (1) MSCFSCFS-SQDKKARRLNNPSRGSQTSYYSAPPRQA-LLPRQRPVNNP--KPKTSAPEPTKDTDAPKATGN----------NNIAAQTFTFRELATATR
    XP_021300925.1-[Herrania-umbratica]-SLL>      (1) MSCFSCFS-SQDKKARRLNNPSRRTQTSYYAAPPREA-LPPQQSLENNP--KPKTSAPEPTKNTDAPKATGN----------NNIAAQTFTFRELATATK
          PON60792.1-[Trema-orientale]-SSL>       (1) MSCFSCFQPSKKKSAKRSISLIRRPGQTPVAVPRKEPNIVPQPRPSENPKPKSPPKIKTTTTNSNTNNNKDTNKEVAAS-GNNNIGAQTFTFRELATATK
      PON70792.1-[Parasponia-andersonii]-SSL>     (1) MSCFSCFQPSKKKSAKRSISLIRRPGQTPVAIPRKEPNIVPQPRPSENPKPKSPPKIETTTTNSNNNNNNKDTNKEVAASGNNNIGAQTFTFRELATATK
         XP_010109722.1-[Morus-notabilis]-SSL>    (1) MSCFSCFQSSNQKRSVKRSRSGRRSGQTLGSVLRKEANIAPQHHHN-----------HHHHHYPATNKEAAG---------NNNIAAQTFSFRELATATR
         XP_015892343.1-[Ziziphus-jujuba]-SSL>    (1) MSCFSCFS-SHEKK--APRRANSARRAQLPPAVHKEP-VSQPHRP-----VTEIPRTKTPAPAEAANKETGN----------NNIAAQTFTFRELATATR
         XP_015892344.1-[Ziziphus-jujuba]-SSL>    (1) MSCFSCFS-SHEKK--APRRANSARRAQLPPAVHKEP-VSQPHRP-------EIPRTKTPAPAEAANKETGN----------NNIAAQTFTFRELATATR
          XP_023915458.1-[Quercus-suber]-SSL>     (1) MSCFSCFS-SHGN---KAKGTNSGRRRELAAAPKELP-ISSQPRPEN----PKPKVIPVETTNKDAIKETAN----------KNIAAQTFTFRELAMATK
         XP_030936979.1-[Quercus-lobata]-SSL>     (1) MSCFSCFS-SHRN---KAKGTNSGRRRELAAAPKELP-ISSQPRPEN----PKPKVIPVETTNKDAIKETAN----------KNIAAQTFTFRELAMATK
             TQD83417.1-[Malus-baccata]-SSL>      (1) MSCFSCFS-SHEKKASNKRSNSGRKEQLPTNLSQEHN-VPPAQQPRPPP-DEVPKPKPTPPAEDPAANTSSANKEPVN---HNNIAAQTFTFRELATATK
        XP_008382639.2-[Malus-domestica]-SSL>     (1) MSCFSCFS-SHEKKASNKRSNSGRKEQLPANLSQEHN-VPPAQQPRPPP-DEVPKPKPTPPAEDPAANTSSANKEPVN---HNNIAAQTFTFRELATATK
 XP_009372484.1-[Pyrus-x-bretschneideri]-SSL>     (1) MSCFSCFS-SHEKKSSNTRSNSGRRAQLPTTVSQKEH-NVPPAQQPRPPPAEVPKPKPTPPAENPAANAHNVNKEPVN---HNNIAAQTFTFRELATATK
          XP_021907535.1-[Carica-papaya]-SLL>     (1) MSCFACFS-SHEKK--TRRTNSHRSAQLPTSAPYKES-HVPPPQPRPDH--GKPKAAPREPTNTGDHGHKEAG---------NNIKAETFTFRELATATK
                                                   101                                                                                                200
        NP_193055.2-[Arabidopsis-thaliana]-SLL>  (70) SFRQEFLIGEGGFGRVYKGKMEKTGQVVAVKQLDRNGLQGNREFLVEIFRLSLLHHPNLANLIGYCLDGDQRLLVHEFMPLGSLEDHLLDVVVGQQPLDW
        OAO98631.1-[Arabidopsis-thaliana]-SLL>   (25) SFRQEFLIGEGGFGRVYKGKMEKTGQVVAVKQLDRNGLQGNREFLVEIFRLSLLHHPNLANLIGYCLDGDQRLLVHEFMPLGSLEDHLLDVVVGQQPLDW
     XP_006285207.1-[Capsella-rubella]-SLL>      (58) SFRQEFLTGEGGFGRVYKGKLEKTGQVVAVKQLDRNGLQGNREFLEEIFRLSLLHHPNLANLIGYCLDGDQRLLVYEFMPLGSLEDHLLDVGVEQQPLDW
            KFK39792.1-[Arabis-alpina]-SKL>      (61) NFRQECLIGEGGFGRVYKGKLEKTGQVVAVKQLDRNGLQGHKEFLVEVLMLSLLHHSNLVNLIGYCADGDQRLLVYEYMPLGSLEDHLLDLEPEQKPLDW
  NP_001326412.1-[Arabidopsis-thaliana]-SQM>     (64) NFRQECLIGEGGFGRVYKGKLENPAQVVAVKQLDRNGLQGQREFLVEVLMLSLLHHRNLVNLIGYCADGDQRLLVYEYMPLGSLEDHLLDLEPGQKPLDW
             EOX93951.1-[Theobroma-cacao]-SLL>   (87) NFRQECLIGEGGFGRVYKGKLEKTGQVVAVKQLDRNGLQGNREFLVEVLMLSLLHHPNLVNLTGYCADGDQRLLVYEYMPLGSLEDHLLDITPDQKPLDW
         XP_007049794.2-[Theobroma-cacao]-SLL>   (87) NFRQECLIGEGGFGRVYKGKLEKTGQVVAVKQLDRNGLQGNREFLVEVLMLSLLHHPNLVNLTGYCADGDQRLLVYEYMPLGSLEDHLLDITPDQKPLDW
    XP_021300925.1-[Herrania-umbratica]-SLL>     (87) NFRQECLIGEGGFGRVYKGNLEKTGQVVAVKQLDRNGLQGNREFLVEVLMLSLLHHPNLVNLTGYCADGDQRLLVYEYMPLGSLEDHLLDITPDQKPLDW
          PON60792.1-[Trema-orientale]-SSL>     (100) NFRQECLVGEGGFGRVYKGRLEKTGQVVAVKQLDRNGLQGNREFLVEVLMLSLLHHQNLVNLIGYCADGDQRLLVYEYMPLGSLEDHLLEFPPGQKPLDW
      PON70792.1-[Parasponia-andersonii]-SSL>   (101) NFRQECLVGEGGFGRVYKGRLEKTGQVVAVKQLDRNGLQGNREFLVEVLMLSLLHHQNLVNLIGYCADGDQRLLVYEYMPLGSLEDHLLEFPPGQKPLDW
         XP_010109722.1-[Morus-notabilis]-SSL>   (81) NFRHECLVGEGGFGRVYKGRLEKTAQVVAVKQLDRNGLQGNREFLVEVLMLSLLHHQNLVNLIGYCADGDQRLLVYEYMPLGSLEDHLLDFAPGQRPLDW
         XP_015892343.1-[Ziziphus-jujuba]-SSL>   (82) NFRQECLIGEGGFGRVYKGRLEKTGQVVAVKQLDRNGLQGNREFLVEVLMLSLLHHQNLVNLIGYCADGEQRLLVYEYMPLGSLEDHLLDFPPDQQPLDW
         XP_015892344.1-[Ziziphus-jujuba]-SSL>   (80) NFRQECLIGEGGFGRVYKGRLEKTGQVVAVKQLDRNGLQGNREFLVEVLMLSLLHHQNLVNLIGYCADGEQRLLVYEYMPLGSLEDHLLDFPPDQQPLDW
          XP_023915458.1-[Quercus-suber]-SSL>    (82) NFRQECLIGEGGFGRVYKGRIEKTSQVVAIKQLDRNGLQGNREFLVEVLMLSLLHHENLVNLIGYCADGEQRLLVYEYMQLGSLEDHLLDVPQEQKPLDW
         XP_030936979.1-[Quercus-lobata]-SSL>    (82) NFRQECLIGEGGFGRVYKGRIEKTSQVVAIKQLDRNGLQGNREFLVEVLMLSLLHHENLVNLIGYCADGEQRLLVYEYMQLGSLEDHLLDVPQEQKPLDW
             TQD83417.1-[Malus-baccata]-SSL>     (95) NFRQECLIGEGGFGRVYKGKLDKTAQVVAVKQLDRNGLQGNREFLVEVLMLSLLHHEHLVNLIGYCADGDQRLLVYEYMPLGSVEDHLLDLPEDQKPLDW
        XP_008382639.2-[Malus-domestica]-SSL>    (95) NFRQECLIGEGGFGRVYKGKLDKTAQVVAVKQLDRNGLQGNREFLVEVLMLSLLHHEHLVNLIGYCADGDQRLLVYEYMPLGSVEDHLLDLPEDQKPLDW
```

```
XP_009372484.1-[Pyrus-x-bretschneideri]-SSL>  (96)  NFRQECLIGEGGFGRVYKGKLDKTEQVVAVKQLDRNGLQGNREFLVEVLMLSLLHHENLVNLIGYCADGDQRLLVYEYMPLGSVEDHLLDLPEGQKPLDW
     XP_021907535.1-[Carica-papaya]-SLL>  (86)  NFRQECLIGEGGFGRVYRGKLDKTGQVVAVKQLDRNGLQGNREFLVEVLMLSLLHHQNLVNLIGYCADGEQRLLVYEYMPLGSLEDHLLDLGENQKPIDW
                                                201                                                                                               300
  NP_193055.2-[Arabidopsis-thaliana]-SLL> (170)  NSRIRIALGAAKGLEYLHEKANPPVIYRDFKSSNILLNVDFDAKLSDFGLAKLGSVGDTQNVSSRVVGTYGYCAPEYHKTGQLTVKSDVYSFGVVLLELI
    OAO98631.1-[Arabidopsis-thaliana]-SLL> (125)  NSRIRIALGAAKGLEYLHEKANPPVIYRDFKSSNILLNVDFDAKLSDFGLAKLGSVGDTQNVSSRVVGTYGYCAPEYHKTGQLTVKSDVYSFGVVLLELI
  XP_006285207.1-[Capsella-rubella]-SLL> (158)  NSRIRIALGAAKGLEYLHEKANPPVIYRDFKSSNILLDEDFDAKLSDFGLAKLGSVGDTQNVSSRVLGTYGYCAPEYQKTGQLTVKSDVYSFGIVLLELI
       KFK39792.1-[Arabis-alpina]-SKL> (161)  NSRIKIALGAAKGLEYLHDKANPPVIYRDLKSSNILLNRDFDAKLSDFGLAKLGPIGDTQHVSSRIMGTYGYCAPEYQRTGHLTVKSDVYSFGVVLLELI
 NP_001326412.1-[Arabidopsis-thaliana]-SQM> (164)  NTRIKIALGAAKGIEYLHDEADPPVIYRDLKSSNILLDPEYVAKLSDFGLAKLGPVGDTLHVSSRVMGTYGYCAPEYQRTGYLTNKSDVYSFGVVLLELI
        EOX93951.1-[Theobroma-cacao]-SLL> (187)  YARMKTALGAAKGLEYLHDKANPPVIYRDMKSSNILLDNGFNAKLSDFGLAKLGPTGDKTHVSSRVMGTYGYCAPEYQRTGQLTVKSDVYSFGVVLLELI
    XP_007049794.2-[Theobroma-cacao]-SLL> (187)  YARMKIALGAAKGLEYLHDKANPPVIYRDMKSSNILLDNGFNAKLSDFGLAKLGPTGDKTHVSSRVMGTYGYCAPEYQRTGQLTVKSDVYSFGVVLLELI
  XP_021300925.1-[Herrania-umbratica]-SLL> (187)  YARMKIALGAAKGLEYLHDKANPPVIYRDMKSSNILLDKGFNAKLSDFGLAKLGPTGDKTHVSSRVMGTYGYCAPEYQRTGQLTVKSDVYSFGVVLLELI
         PON60792.1-[Trema-orientale]-SSL> (200)  YKRMRIALGAAKGLEYLHDKANPPVIYRDLKSSNILLDKSFNAKLSDFGLAKLGPVGDKTHVSSRVMGTYGYCAPEYQRTGQLTLKSDVYSFGVVLLELI
   PON70792.1-[Parasponia-andersonii]-SSL> (201)  YKRMRIALGAAKGLEYLHDKANPPVIYRDLKSSNILLDKSFNAKLSDFGLAKLGPVGDKTHVSSRVMGTYGYCAPEYQRTGQLTLKSDVYSFGVVLLELI
    XP_010109722.1-[Morus-notabilis]-SSL> (181)  YKRMRIALGAAKGLEYLHDKANPPVIYRDLKSSNILLDKNFNAKLSDFGLAKLGPVGDKTHVSSRVMGTYGYCAPEYQRTGQLTVKSDVYSFGVVLLELI
    XP_015892343.1-[Ziziphus-jujuba]-SSL> (182)  YKRMKIALGAAKGLEYLHDKANPPVIYRDLKSSNILLDKDFNAKLSDFGLAKLGPTGDKTHVSSRVMGTYGYCAPEYQRTGQLTLKSDVYSFGVVLLELI
    XP_015892344.1-[Ziziphus-jujuba]-SSL> (180)  YKRMKIALGAAKGLEYLHDKANPPVIYRDLKSSNILLDKDFNAKLSDFGLAKLGPTGDKTHVSSRVMGTYGYCAPEYQRTGQLTLKSDVYSFGVVLLELI
     XP_023915458.1-[Quercus-suber]-SSL> (182)  FTRMKIALEAAKGLEYLHDKANPPVIYRDLKSSNILLDKDFNAKLSDFGLAKLGPVGDKTHVSSRVMGTYGYCAPEYQRTGQLTVKSDVYSFGVVLLELI
    XP_030936979.1-[Quercus-lobata]-SSL> (182)  FTRMKIALEAAKGLEYLHDKANPPVIYRDLKSSNILLDKDFNAKLSDFGLAKLGPVGDKTHVSSRVMGTYGYCAPEYQRTGQLTVKSDVYSFGVVLLELI
        TQD83417.1-[Malus-baccata]-SSL> (195)  FKRLRIALGAAKGLEYLHDKANPPVIYRDLKSSNILLDADFNAKLSDFGLAKLGPIGDKTHVSSRVMGTYGYCAPEYQRTGQLTVKSDVYSFGVVLLELI
    XP_008382639.2-[Malus-domestica]-SSL> (195)  FKRLRIALGAAKGLEYLHDKANPPVIYRDLKSSNILLDADFNAKLSDFGLAKLGPIGDKTHVSSRVMGTYGYCAPEYQRTGQLTVKSDVYSFGVVLLELI
XP_009372484.1-[Pyrus-x-bretschneideri]-SSL> (196)  FKRMRIALGAAKGLEYLHDKANPPVIYRDLKSSNILLDNDFNAKLSDFGLAKLGPTGDKTHVSSRVMGTYGYCAPEYQRTGQLTVKSDVYSFGVVLLELI
     XP_021907535.1-[Carica-papaya]-SLL> (186)  FTRMKIALGAAKGLEYLHDKANPPVIYRDLKSSNILLDKEFRAKLSDFGLAKLGPVGDKTHVSSRVMGTYGYCAPEYQRTGQLTVKSDVYSFGVVLLELI
                                                301                                                                                               400
  NP_193055.2-[Arabidopsis-thaliana]-SLL> (270)  TGKRVIDTTRPCHEQNLVTWAQPIFREPNRFPELADPLLQGEFPEKSLNQAVAIAAMCLQEEPIVRPLISDVVTALSFMSTETGSPSGLT-------GTA
    OAO98631.1-[Arabidopsis-thaliana]-SLL> (225)  TGKRVIDTTRPCHEQNLVTWAQPIFREPNRFPELADPLLRGEFQEKSLNQAVAIAAMCLQEEPIVRPLISDVVTALSFMSTETGSPSDIT-------GTA
  XP_006285207.1-[Capsella-rubella]-SLL> (258)  TGRRVIDTTRPSREQNLVTWAQPIFRDPNRFPELADPLLQGEFPEKCLNQAVAVAAMCLQEEPIVRPLMSDIVTTLSFMSEKTDSP-----------TV
       KFK39792.1-[Arabis-alpina]-SKL> (261)  TGRRVIDPTRPSNEQNLVTWAQPVFREPSKFPELADPFLRGEFREKSLNQAVAVAAMCLHEEPTVRPLISDVVTALSFLGEEHTSRRDDYEKEPSVEATA
 NP_001326412.1-[Arabidopsis-thaliana]-SQM> (264)  SGRRVIDTMRPSHEQNLVTWALPIFRDPTRYWQLADPLLRGDYPEKSLNQAIAVAAMCLHEEPTVRPLMSDVITALSFLGASSNSSN-----------TG
        EOX93951.1-[Theobroma-cacao]-SLL> (287)  TGRRAIDTTRPNKEQNLVTWAQPLFKEPRRFPELADPLLKGDFPIRGLNQAVAVAAMCLQEEASVRPLISDVVTALSFLGNGPDAN------------TA
    XP_007049794.2-[Theobroma-cacao]-SLL> (287)  TGRRAIDTTRPNKEQNLVTWAQPLFKEPRRFPELADPLLKGDFPIRGLNQAVAVAAMCLQEEASVRPLISDVVTALSFLGNGPDAN------------TA
  XP_021300925.1-[Herrania-umbratica]-SLL> (287)  TGRRAIDTTRPNKEQNLVTWAEPLFKEPRRFPELADPLLKGDFPVRGLNQAVAVAAMCLQEEASVRPLISDVVTALSFLGNGPDAN------------TA
         PON60792.1-[Trema-orientale]-SSL> (300)  TGRRAIDTTKPSTEQNLVTWANPVFKTPQRYPELADPLLRGEYPVRGLHQAVAVAAMCLNEEPSVRPLISDVVTALSFLGTSPDTAN---------VATA
   PON70792.1-[Parasponia-andersonii]-SSL> (301)  TGRRAIDTTKPSTEQNLVTWANPVFKTPQRYPELADPLLRGEYPVRGLHQAVAVAAMCLNEEPSVRPLISDAVTALSFLGTSPGAT-------------A
    XP_010109722.1-[Morus-notabilis]-SSL> (281)  TGRRAIDTTKPTTEHNLVCWANPAFKDPKKYPQLADPLLQGDFPIRGLHQAVAVAAMCLNEEPSVRPLISDVVTALSFLGSGPDTDTDTCTTP-----RI
    XP_015892343.1-[Ziziphus-jujuba]-SSL> (282)  TGRRAIDTTRNSEEQNLVSWAHPIFKEPHRYGELADPLIKGDLPVRGLNQAVAVAAMCLNEEASARPLMSDVVTALSFLGTEPEHG------------AT
    XP_015892344.1-[Ziziphus-jujuba]-SSL> (280)  TGRRAIDTTRNSEEQNLVSWAHPIFKEPHRYGELADPLIKGDLPVRGLNQAVAVAAMCLNEEASARPLMSDVVTALSFLGTEPEHG------------AT
     XP_023915458.1-[Quercus-suber]-SSL> (282)  TGKRAIDTTRYTEEQNLVTWAQPIFKDPQRFPELADPLLHGDFPLRALHQAVAVAAMCLQEEPSVRPLISDVVTALSFLGTSPGAN------------TT
    XP_030936979.1-[Quercus-lobata]-SSL> (282)  TGRRAIDTTRYTEEQNLVTWAQPIFKDPQRFPELADPLLHGDFPLRALHQAVAVAAMCLQEEPSVRPLISDVVTALSFLGTSPSAN------------TT
        TQD83417.1-[Malus-baccata]-SSL> (295)  TGRRAIDTTLTTREQNLVSWAQPVFKDPNRYPELADPLLQGNFPMRALNQAVAIAAMCLHEEAPVRPLMSDVVSALSFLGTGPDTAT-----------SP
    XP_008382639.2-[Malus-domestica]-SSL> (295)  TGRRAIDTTLTTREQNLVAWAQPVFKDPNRYPELADPLLQGNFPMRALNQAVAIAAMCLHEEAPVRPLMSDVVSALSFLGTGPDTAT-----------SP
XP_009372484.1-[Pyrus-x-bretschneideri]-SSL> (296)  TGRRAIDTTLSTREQNLVAWAQPVFKDPNRYPELADPLLQGKFPVRALNQAMAVAAMCLHEEAPVRPLISDVVSALSVLGTGPDTA------------TS
     XP_021907535.1-[Carica-papaya]-SLL> (286)  TGRRAIDTTRSTNEQNLVAWAQPVFKEPSRFPELADPLLKGEFPVRGLNQAVAVAAMCLHEEPAVRPLISDVVTALSFLGTDPDQT------------TV
                                                401                                                                                               500
  NP_193055.2-[Arabidopsis-thaliana]-SLL> (363)  LN---PLSPKTVEDQGWLQCE-SPRDV-YSLL---------------------------------------------------------------------
    OAO98631.1-[Arabidopsis-thaliana]-SLL> (318)  LNHFQPLSPKTVEDQGWLQCESAPRDV-YSLL---------------------------------------------------------------------
  XP_006285207.1-[Capsella-rubella]-SLL> (346)  LNHFHPSFGKTVEDQGVFQRELAPRDM-YSLL---------------------------------------------------------------------
       KFK39792.1-[Arabis-alpina]-SKL> (361)  TN--VAAQGDITKVKMMLETFLTEQKK-QAKINEATSKELKALSRRKRGRLNTARLRARVDPQRLDFSAPRTAQRNPDDLPQPPPPRRELDKSPKRVVDIS
 NP_001326412.1-[Arabidopsis-thaliana]-SQM> (353)  SN--HLQQNRSNKYQDAVQWDSSPRYA-NSQM---------------------------------------------------------------------
        EOX93951.1-[Theobroma-cacao]-SLL> (375)  TSSFPSLESDKQPIFGNGASYVEDSKK-ERQRAVAEAIEWGSSSRNAQSQTHSSLL---------------------------------------------
    XP_007049794.2-[Theobroma-cacao]-SLL> (375)  TSSFPSLESDKQPIFGNGASYVEDSKK-ERQRAVAEAIEWGSSSRNAQSQTHSSLL---------------------------------------------
  XP_021300925.1-[Herrania-umbratica]-SLL> (375)  TSSFPSLESDQQPIFGNGASYIEDSKK-ERQRAVAEAIEWGSSSRNTQSQTHSSLL---------------------------------------------
         PON60792.1-[Trema-orientale]-SSL> (391)  HTSSSSPSSSPPSDQNGRSQQDEERLLQERQRAVQEAIEWGSTSRHNNGKSRFGSASSL------------------------------------------
   PON70792.1-[Parasponia-andersonii]-SSL> (388)  H--TSSPSSSPPSDQNGRSQQDEDRLLQERQRAVQEAIEWGSTSRHNNGKSRFGSASSL------------------------------------------
    XP_010109722.1-[Morus-notabilis]-SSL> (376)  SSSSSPRSDKNVGGAKHGRSQEEENTLRERQRAVAEAIEWGSTSRHNSKARFGSASSL-------------------------------------------
    XP_015892343.1-[Ziziphus-jujuba]-SSL> (370)  AISLPSPPIEHNMSGAQAKPEDEESMIKERERAVAEAMEWGGSHSRHKTKSRSTSASSL------------------------------------------
    XP_015892344.1-[Ziziphus-jujuba]-SSL> (368)  AISLPSPPIEHNMSGAQAKPEDEESMIKERERAVAEAMEWGGSHSRHKTKSRSTSASSL------------------------------------------
     XP_023915458.1-[Quercus-suber]-SSL> (370)  SFDDPSPPSDHNMMSENQNLRDEESIR-ERQRAVAEAIEWGSNSRHTASRCGSASSL--------------------------------------------
    XP_030936979.1-[Quercus-lobata]-SSL> (370)  SFDDPSPPSDHNMMSENQNLRDEESIR-ERQRAVAEAIEWGSNSRRTASRCGSASSL--------------------------------------------
        TQD83417.1-[Malus-baccata]-SSL> (384)  ISSLPSPSPDKTMSVVNEDPQLEDSVT-ERQRAVAEAMEWGSSSRHNGVEALRCGSTSSL-----------------------------------------
    XP_008382639.2-[Malus-domestica]-SSL> (384)  ISSLPSPSPDQTMSVVNEDPQLEDSVT-ERQRAVAEAMEWGSSSRHNGVEALRCGSTSSL-----------------------------------------
XP_009372484.1-[Pyrus-x-bretschneideri]-SSL> (384)  PVSSLPSPSPDQTMVINADSQLADSVT-ERQRAVAEAMEWGSSSRHNGVVASRCGSTSSL-----------------------------------------
     XP_021907535.1-[Carica-papaya]-SLL> (374)  PVSVPSPASDKNSGDHRDATE--------RQRAVAEAMEWGSNSRNQSESGTASLL---------------------------------------------
                                                501                                                                                               600
  NP_193055.2-[Arabidopsis-thaliana]-SLL> (390)  ----------------------------------------------------------------------------------------------------
    OAO98631.1-[Arabidopsis-thaliana]-SLL> (349)  ----------------------------------------------------------------------------------------------------
  XP_006285207.1-[Capsella-rubella]-SLL> (377)  ----------------------------------------------------------------------------------------------------
       KFK39792.1-[Arabis-alpina]-SKL> (458)  DGDEPAPQKRAQAEGAEGSRQEPSASNTQSASDSSKTWVVKDTSEYTTYSNEEQNRKERERRALKRSYTTQLAEMQAQMERVASDIKRTQSQIHHVTGRA
 NP_001326412.1-[Arabidopsis-thaliana]-SQM> (382)  ----------------------------------------------------------------------------------------------------
```

```
          EOX93951.1-[Theobroma-cacao]-SLL>  (430) ----------------------------------------------------------------------------------------------------
      XP_007049794.2-[Theobroma-cacao]-SLL>  (430) ----------------------------------------------------------------------------------------------------
   XP_021300925.1-[Herrania-umbratica]-SLL>  (430) ----------------------------------------------------------------------------------------------------
          PON60792.1-[Trema-orientale]-SSL>  (450) ----------------------------------------------------------------------------------------------------
    PON70792.1-[Parasponia-andersonii]-SSL>  (445) ----------------------------------------------------------------------------------------------------
      XP_010109722.1-[Morus-notabilis]-SSL>  (434) ----------------------------------------------------------------------------------------------------
      XP_015892343.1-[Ziziphus-jujuba]-SSL>  (429) ----------------------------------------------------------------------------------------------------
      XP_015892344.1-[Ziziphus-jujuba]-SSL>  (427) ----------------------------------------------------------------------------------------------------
        XP_023915458.1-[Quercus-suber]-SSL>  (426) ----------------------------------------------------------------------------------------------------
       XP_030936979.1-[Quercus-lobata]-SSL>  (426) ----------------------------------------------------------------------------------------------------
            TQD83417.1-[Malus-baccata]-SSL>  (443) ----------------------------------------------------------------------------------------------------
      XP_008382639.2-[Malus-domestica]-SSL>  (443) ----------------------------------------------------------------------------------------------------
XP_009372484.1-[Pyrus-x-bretschneideri]-SSL>  (443) ----------------------------------------------------------------------------------------------------
        XP_021907535.1-[Carica-papaya]-SLL>  (422) ----------------------------------------------------------------------------------------------------
                                                   601                                                654
    NP_193055.2-[Arabidopsis-thaliana]-SLL>  (390) ------------------------------------------------------
     OAO98631.1-[Arabidopsis-thaliana]-SLL>  (349) ------------------------------------------------------
     XP_006285207.1-[Capsella-rubella]-SLL>  (377) ------------------------------------------------------
            KFK39792.1-[Arabis-alpina]-SKL>  (558) PAIAQVLEKVRNMPFNERIARTKISGLGEHALRKNYRPFGPSDRIRSGLGESKL
 NP_001326412.1-[Arabidopsis-thaliana]-SQM>  (382) ------------------------------------------------------
          EOX93951.1-[Theobroma-cacao]-SLL>  (430) ------------------------------------------------------
      XP_007049794.2-[Theobroma-cacao]-SLL>  (430) ------------------------------------------------------
   XP_021300925.1-[Herrania-umbratica]-SLL>  (430) ------------------------------------------------------
          PON60792.1-[Trema-orientale]-SSL>  (450) ------------------------------------------------------
    PON70792.1-[Parasponia-andersonii]-SSL>  (445) ------------------------------------------------------
      XP_010109722.1-[Morus-notabilis]-SSL>  (434) ------------------------------------------------------
      XP_015892343.1-[Ziziphus-jujuba]-SSL>  (429) ------------------------------------------------------
      XP_015892344.1-[Ziziphus-jujuba]-SSL>  (427) ------------------------------------------------------
        XP_023915458.1-[Quercus-suber]-SSL>  (426) ------------------------------------------------------
       XP_030936979.1-[Quercus-lobata]-SSL>  (426) ------------------------------------------------------
            TQD83417.1-[Malus-baccata]-SSL>  (443) ------------------------------------------------------
      XP_008382639.2-[Malus-domestica]-SSL>  (443) ------------------------------------------------------
XP_009372484.1-[Pyrus-x-bretschneideri]-SSL>  (443) ------------------------------------------------------
        XP_021907535.1-[Carica-papaya]-SLL>  (422) ------------------------------------------------------
```

PTD4_ AT5G49660.1 (Verified in this study as peroxisomal)

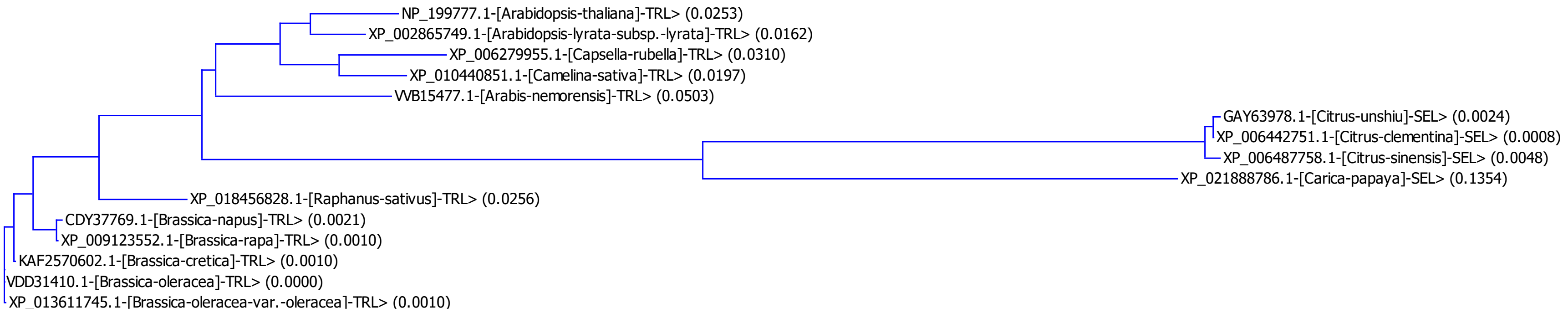


```
                                                                 1                                                                                                                      120
NP_199777.1-[Arabidopsis-thaliana]-TRL>                    (1) ------------------------------------------------------------------------------------------------------------------------
XP_002865749.1-[Arabidopsis-lyrata-subsp.-lyrata]-...      (1) ------------------------------------------------------------------------------------------------------------------------
XP_006279955.1-[Capsella-rubella]-TRL>                     (1) ------------------------------------------------------------------------------------------------------------------------
XP_010440851.1-[Camelina-sativa]-TRL>                      (1) ------------------------------------------------------------------------------------------------------------------------
VVB15477.1-[Arabis-nemorensis]-TRL>                        (1) ------------------------------------------------------------------------------------------------------------------------
GAY63978.1-[Citrus-unshiu]-SEL>                            (1) ------------------------------------------------------------------------------------------------------------------------
XP_006442751.1-[Citrus-clementina]-SEL>                    (1) ------------------------------------------------------------------------------------------------------------------------
XP_006487758.1-[Citrus-sinensis]-SEL>                      (1) ------------------------------------------------------------------------------------------------------------------------
XP_021888786.1-[Carica-papaya]-SEL>                        (1) ------------------------------------------------------------------------------------------------------------------------
XP_018456828.1-[Raphanus-sativus]-TRL>                     (1) ------------------------------------------------------------------------------------------------------------------------
CDY37769.1-[Brassica-napus]-TRL>                           (1) MCNHHSSGKYLETSPKACIDRHHPPDIVRHHPRDTDRHHRSDIDRQLLLEEPPGYIVELKPVEERMHKSEASHLAVREHLRPPVCAEEATGFHKRLKMIHDPMKLVVPCTIFEVESPSPG
XP_009123552.1-[Brassica-rapa]-TRL>                        (1) ------------------------------------------------------------------------------------------------------------------------
KAF2570602.1-[Brassica-cretica]-TRL>                       (1) ------------------------------------------------------------------------------------------------------------------------
VDD31410.1-[Brassica-oleracea]-TRL>                        (1) ------------------------------------------------------------------------------------------------------------------------
XP_013611745.1-[Brassica-oleracea-var.-oleracea]-T...      (1) ------------------------------------------------------------------------------------------------------------------------
                                                                 121                                                                                                                    240
NP_199777.1-[Arabidopsis-thaliana]-TRL>                    (1) ------------------------MRLKNFPFFVLFFFFCFNSNQSWGLMSSNQQPQFFKLMKNSLFGDALSTWNV-YDVGTNYCNFTGVRCDGQGLVTDLDLSGLSLSGIFPDGVCSYF
XP_002865749.1-[Arabidopsis-lyrata-subsp.-lyrata]-...      (1) ------------------------MRLKIFPFLVLFFFFCFNNNQSWGLMSSIQQPQFFKLMKNSLSG--LSSWNV-SDVGTYYCNFNGVRCDGQGLVTDLDLSGLYLSGIFPEGICSYL
XP_006279955.1-[Capsella-rubella]-TRL>                     (1) --------------------------MRLKLFLFFFFFFCFSNNQSWGLMSSNQQPQFFKLMKSSLFGDALSSWNVSDDVGAYYCDFTGVRCDGQGLVTDLDLSGWSLSGVFPDGICSYF
XP_010440851.1-[Camelina-sativa]-TRL>                      (1) ----------------------MHLNFFTVFVLFFFFFFCFSNNQSWGLMSSNQQPQFFKLMKNSLFGDALSSWNVSDDVGTNYCDFTGVRCDGQGLVTDLDLSSWSLSGVFPDGICSYF
VVB15477.1-[Arabis-nemorensis]-TRL>                        (1) -------------------------MRLRFFPFFVLFFFSFNSNLSWGLMSPNQQPQFFKLMRNSFSGNALSSWNL----SADNCKLSGVICDGEGLVTDLDLSGRSLSGIFPDGICSYL
GAY63978.1-[Citrus-unshiu]-SEL>                            (1) ---------------------------MALKSIFFLLVLLTGLSNPSQAISVNHESQFFKLMKTSLSGDKLSDWDIDG--GKPYCNFSGISCNDQGHVSMIDISGWLLAGQFPSGVCAYL
XP_006442751.1-[Citrus-clementina]-SEL>                    (1) ---------------------------MALKSIFFLLVLLTGLSNPSQAISVNHESQFFKLMKTSLSGDKLSDWDIDG--GKPYCNFSGISCNDQGHVSMIDISGWLLAGQFPSGVCAYL
XP_006487758.1-[Citrus-sinensis]-SEL>                      (1) ---------------------------MALKSIFFLLVLLTGLSNPSQAISVNHESQFFKLMKTSLSGDKLSDWDIDG--GKPYCNFSGISCNDQGHVSMIDISGWLLAGQFPSGVCSYL
XP_021888786.1-[Carica-papaya]-SEL>                        (1) ---------------------------MASKSTFVILFVLIMFLSCPSEVTSTNQSQFFTLMKASLSGNALSDWDVSG--GKPFCNFTGVGCNDRGYVEEIDISGWMLSGYFPSDICSYL
XP_018456828.1-[Raphanus-sativus]-TRL>                     (1) -------------------------MLLRFVPVFVLLIFCFNSKLSCGLMSSNQQSEFFKLMKNSLSSDALSSWNLSDEVTSYYCNFTGVRCDGQGLVTDLDLSGLSLSGIFPDGICSFL
CDY37769.1-[Brassica-napus]-TRL>                         (121) DINPKVLSFLRPKFAPAAYFRAFPEMLFRFFPVFVLLIFCFNSNQSWGLMTSNQQSQFFKLMKNSLSSDALSYWNLSDAVTSYYCNFTGVRCDGQGLVTDLDLSGLSLSGIFPDGICSFL
XP_009123552.1-[Brassica-rapa]-TRL>                        (1) -------------------------MLFRLFPVFVLLIFCFNSNQSWGLMTSNQQSQFFKLMKNSLSSDALSSWNLSDAVTSYYCNFTGVRCDGQGLVTDLDLSGLSLSGIFPDGICSFL
KAF2570602.1-[Brassica-cretica]-TRL>                       (1) ---PKVLSFLRPKFAPAAYFRAFPEMLFRFFPVFVLLIFCFNSNQLWGLMTSNQQSQFFKLMKNSLSSDALSSWNLSDAVTSYYCNFTGVRCDGQGLVTDLDLSGLSLSGIFPDGICSFL
VDD31410.1-[Brassica-oleracea]-TRL>                        (1) --DPKVLSFLRPKFAPAAYFRAFPEMLFRFFPVFVLLIFCFNSNQLWGLMTSHQQSQFFKLMKNSLSSDALSSWNLSDAVTSYYCNFTGVRCDGQGLVTDLDLSGLSLSGIFPDGICSFL
XP_013611745.1-[Brassica-oleracea-var.-oleracea]-T...      (1) -------------------------MLFRFFPVFVLLIFCFNSNQLWGLMTSHQQSQFFKLMKNSLSSDALSSWNLSDAVTSYYCNFTGVRCDGQGLVTDLDLSGLSLSGIFPDGICSFL
                                                                 241                                                                                                                    360
NP_199777.1-[Arabidopsis-thaliana]-TRL>                   (96) PNLRVLRLSHNHLNKSSSFLNTIPNCSLLRDLNMSSVYLKGTLPDFSQMKSLRVIDMSWNHFTGSFPLSIFNLTDLEYLNFNENPELDLWTLPDS-VSKLTKLTHMLLMTCMLHGNIPRS
XP_002865749.1-[Arabidopsis-lyrata-subsp.-lyrata]-...     (94) PNLRVLRLSHNHLNRSSSFLNTIPNCSLLQELNMSSVYLKGTLPDFSPMKSLRVIDMSWNHFTGSFPISIFNLTDLEYLNFNENPELDLWTLPDY-VSKLTKLTHMLLMTCMLHGNIPRS
XP_006279955.1-[Capsella-rubella]-TRL>                    (95) PNLRVLRLSRNHLNRSSSFLSSIPNCSLLRELNMSSLYLQGTLPDFSSMKALRVIDMSWNYFSGSFPFSIFDLTDLEYLNFNENPELDLWTLPES-ASKLKKLTHMLLMTCMLHGNIPRF
XP_010440851.1-[Camelina-sativa]-TRL>                     (99) PNLRVLRLSRNHLNRSSSFLSTIPSCSLLRELNMSSLYLKGTLPDFSSMKALRVIDMSWNHFTGSFPFSIFNLTDLEYLNFNENPELDLWTLPDS-VSKLTKLTHMLLMTCMLHGNIPRS
VVB15477.1-[Arabis-nemorensis]-TRL>                       (92) PNLRVLRLSHNHLNRSSSFLHTIPNCSMLRELNLSALYLKATLPDFSPMKSLRVIDMSWNHFTGGFPLSIFNLTDLEYLNFNENPELHLWTLPDY-VSNLKKLTHMLLMTCMLHGNIPRS
GAY63978.1-[Citrus-unshiu]-SEL>                           (92) PELQVLRLARNH-VDGSNLLDSIVNCSLLEELNMSFMYLTGTLPDFSPMQNLRRLDLSNNLFTGQFPLSVFNLTNLEVLSFNENPGFKLWKLPESSIFRLTKLRIMVLATCALHGQIPAS
XP_006442751.1-[Citrus-clementina]-SEL>                   (92) PELQVLRLARNH-VDGSNLLDSIVNCSLLEELNMSFMYLTGTLPDFSPMQNLRRLDLSNNLFTGQFPLSVFNLTNLEVLSFNENPGFKLWKLPESSIFRLTKLRIMVLATCALHGQIPAS
XP_006487758.1-[Citrus-sinensis]-SEL>                     (92) PELQVLRLARNH-VHGSNLLDSIVNCSLLEELNMSFMYLTGTLPDFSPMQNLQRLDLSNNLFTGQFPLSVFNLTNLEVLSFNENPGFKLWKLPESSIFRLTKLRIMVLATCALHGQIPAS
XP_021888786.1-[Carica-papaya]-SEL>                       (92) PELRVLRLSQN--NLHGNFLNSIVNCSLLEEFNMSFVYLRANLPDLSRMKSIWILDMSYNLFTGDFPMSITNLTSLELLTFNENGEFNLWELPDN-ISRLVNLKVMVLSTCMLQGPIPAS
XP_018456828.1-[Raphanus-sativus]-TRL>                    (96) PNLRVLRLSRNHLNRSSSFLKDIPDCSLLRELNMSSLYLTATLPDFSPMKSLRVIDMSWNHFTGSFPLSIFNLTDLEYLNFNENPELDLWTLPDY-VSKLTKLTHMLLMTCMLHGNIPRS
CDY37769.1-[Brassica-napus]-TRL>                         (241) PNLRVLRLSRNHLNRSSSFLNDIPDCSLLQELNMSSLYLTATLPDFSPMKSLRVIDMSWNHFTGSFPLSIFNLTDLQYLNFNENPEFDLWTLPYY-VSKLTKLTHMLLMTCMLHGNIPRS
XP_009123552.1-[Brassica-rapa]-TRL>                       (96) PNLRVLRLSRNHLNRSSSFLNDIPDCSLLQELNMSSLYLTATLPDFSPMKSLRVIDMSWNHFTGSFPLSIFNLTDLQYLNFNENPEFDLWTLPYY-VSKLTKLTHMLLMTCMLHGNIPRS
KAF2570602.1-[Brassica-cretica]-TRL>                     (118) PNLRVLRLSRNHLNRSSSFLNDIPDCSLLQELNMSSLYLTATLPDFSPMKSLRVIDMSWNHFTGSFPLSIFNLTDLEYLNFNENPEFDLWTLPDY-VSKLKKLTHMLLMTCMLHGNIPRS
VDD31410.1-[Brassica-oleracea]-TRL>                      (119) PNLRVLRLSRNHLNRSSSFLNDIPDCSLLQELNMSSLYLTATLPDFSPMKSLRVIDMSWNHFTGSFPLSIFNLTDLEYLNFNENPEFDLWTLPDY-VSKLKKLTHMLLMTCMLHGNIPRS
XP_013611745.1-[Brassica-oleracea-var.-oleracea]-T...     (96) PNLRVLRLSRNHLNRSSSFLNDIPDCSLLQELNMSSFYLTATLPDFSPMKSLRVIDMSWNHFTGSFPLSIFNLTDLEYLNFNENPEFDLWTLPDY-VSKLKKLTHMLLMTCMLHGNIPRS
                                                                 361                                                                                                                    480
NP_199777.1-[Arabidopsis-thaliana]-TRL>                  (215) IGNLTSLVDLELSGNFLSGEIPKEIGNLSNLRQLELYYNYHLTGSIPEEIGNLKNLTDIDISVSRLTGSIPDSICSLPNLRVLQLYNNSLTGEIPKSLGNSKTLKILSLYDNYLTGELPP
XP_002865749.1-[Arabidopsis-lyrata-subsp.-lyrata]-...    (213) IGNLTSLVDLELSGNFLSGEIPKEIGNLSNLRQLELYYNYHLTGSIPEEIGNLKNLTDIDISVSRLTGSIPDSICSLPKLRVLQLYNNSLTGEIPKSLGKSKTLKILSLYDNYLTGELPP
```

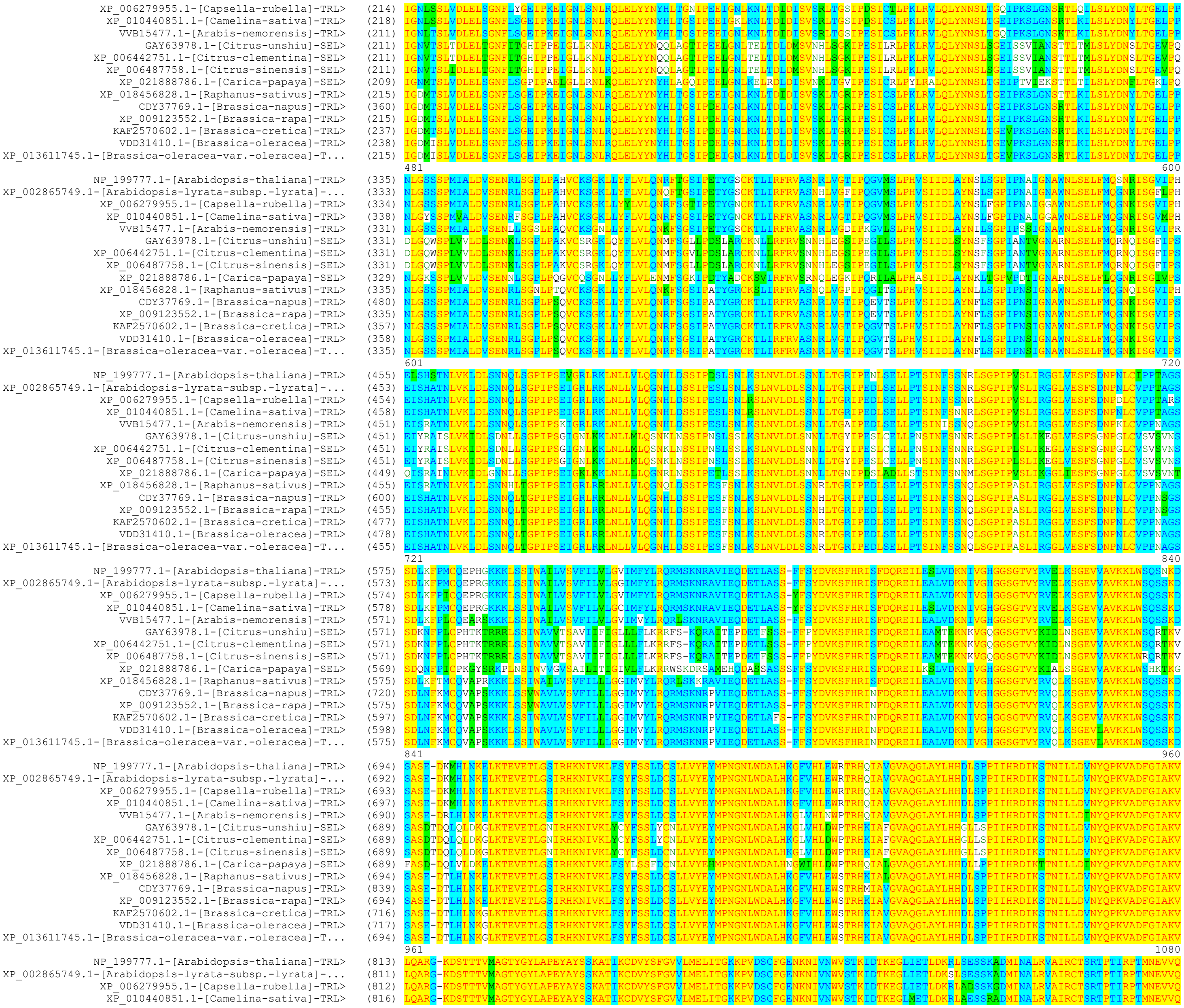

```
                             VVB15477.1-[Arabis-nemorensis]-TRL>   (809) LQARS-KDSTTTVIAGTYGYLAPEYAYSSKATIKCDVYSFGVVLMELITGKKPVDACFGENKNIVNWVSTKIDTKEGLMETLDKRLSESSKADMINALRVAIRCTSRTPTIRPTMNEVVQ
                                GAY63978.1-[Citrus-unshiu]-SEL>   (809) LQARGGKDSTTTVIAGTYGYLAPEYAYSSKATTKCDVYSFGVVLMELITGRKPVEDDFGDNKNIIYWVSIKVDTKEGIMEVLDKKLSGSFRDEMIEVLRIAIRCTSKSPATRPTMNEVVQ
                         XP_006442751.1-[Citrus-clementina]-SEL>   (809) LQARGGKDSTTTVIAGTYGYLAPEYAYSSKATTKCDVYSFGVVLMELITGRKPVEDDFGDNKNIIYWVSTKVDTKEGIMEVLDKKLSGSFRDEMIEVLRIAIRCTSKSPATRPTMNEVVQ
                           XP_006487758.1-[Citrus-sinensis]-SEL>   (809) LQARGGKDSTTTVIAGTYGYLAPEYAYSSKATTKCDVYSFGVVLMELITGRKPVEDDFGDNKNIIYWVSIKVDTKEGIMEVLDKKLSGSFRDEMIQVLRIAIRCTSKSPATRPTMNEVVQ
                             XP_021888786.1-[Carica-papaya]-SEL>   (808) LQARG-KDSTTTVIAGTYGYLAPEYAYSSKATTKCDVYSFGVVLMELITGKKPVEADFGENKNIIYWVATKVETKEGVMQVLDKCLSGSFRDEMIQVLRIAIRCTCKTPSLRPSMNEVVQ
                         XP_018456828.1-[Raphanus-sativus]-TRL>   (813) LQARG-KDSTTTVIAGTYGYLAPEYAYSTKATIKCDVYSFGVMLMELITGKKPVDSCFGENKNIVNWVSTKIDTKEGLIETLDKRLSEPSKADMINALRVAIRCTSRSPTIRPTMNEVVQ
                                CDY37769.1-[Brassica-napus]-TRL>   (958) LQARG-KDSTTTVIAGTYGYLAPEYAYSSKATTKCDVYSFGVMLMELITGKKPVDSCFGENKNIVNWVSTKIDTKEGLIETLDKRLSESSKADMINALRVAIRCTSRTPTIRPSMNEVVQ
                             XP_009123552.1-[Brassica-rapa]-TRL>   (813) LQARG-KDSTTTVIAGTYGYLAPEYAYSSKATTKCDVYSFGVMLMELITGKKPVDSCFGENKNIVNWVSTKIDTKEGLIETLDKRLSESSKADMINALRVAIRCTSRTPTIRPSMNEVVQ
                            KAF2570602.1-[Brassica-cretica]-TRL>   (835) LQARG-KDSTTTVIAGTYGYLAPEYAYSSKATIKCDVYSFGVMLMELITGKKPVDSCFGENKNIVNWVSTKIDTKEGLIETLDKRLSESSKTDMINALRVAIRCTSRTPTIRPSMNEVVQ
                             VDD31410.1-[Brassica-oleracea]-TRL>   (836) LQARG-KDSTTTVIAGTYGYLAPEYAYSSKATIKCDVYSFGVMLMELITGKKPVDSCFGENKNIVNWVSTKIDTKEGLIETLDKRLSESSKTDMINALRVAIRCTSRTPTIRPSMNEVVQ
       XP_013611745.1-[Brassica-oleracea-var.-oleracea]-T...   (813) LQARG-KDSTTTVIAGTYGYLAPEYAYSSKATIKCDVYSFGVMLMELITGKKPVDSCFGENKNIVNWVSTKIDTKEGLIETLDKRLSESSKTDMINALRVAIRCTSRTPTIRPSMNEVVQ
                                                                        1081                                 1118
                      NP_199777.1-[Arabidopsis-thaliana]-TRL>   (932) LLIDATPQGGPDMTS--KPTTKIKDSIVS-DHLTQTRL
     XP_002865749.1-[Arabidopsis-lyrata-subsp.-lyrata]-...   (930) LLIDAAPQGGPDMTS--KSTTKIKDSIVS-DHLTQTRL
                         XP_006279955.1-[Capsella-rubella]-TRL>   (931) LLIDAAPQGGPDHMTSKSTTTKIKDLVLSSDQLTQTRL
                          XP_010440851.1-[Camelina-sativa]-TRL>   (935) LLIDAAPQGGPDMTS--KSTTKIKDLVLSSDHLTQTRL
                             VVB15477.1-[Arabis-nemorensis]-TRL>   (928) LLIDAAPQGGPDMTS--KSTTKIKDAMLS-DHITQTRL
                                GAY63978.1-[Citrus-unshiu]-SEL>   (929) LLAEADPCRFESCKFP---NKSNKESSNATKIKNPSEL
                         XP_006442751.1-[Citrus-clementina]-SEL>   (929) LLAEADPCRFESCKFP---NKSNKESSNATKIKNPSEL
                           XP_006487758.1-[Citrus-sinensis]-SEL>   (929) LLAEADPCRFESCKFP---NKSNKESSNATKIKNPSEL
                             XP_021888786.1-[Carica-papaya]-SEL>   (927) MLIEADPCRFDSCKFS--NKTKDESNVMN-KPKNTSEL
                         XP_018456828.1-[Raphanus-sativus]-TRL>   (932) LLIDAAPQEGLDMAS--KSTTKIKDAILF-DNLTQTRL
                                CDY37769.1-[Brassica-napus]-TRL>  (1077) LLIDAAPQEGLDMAS--KSRTKIKDAILS-DNPTQTRL
                             XP_009123552.1-[Brassica-rapa]-TRL>   (932) LLIDAAPQEGLDMAS--KSRTKIKDAILS-DNPTQTRL
                            KAF2570602.1-[Brassica-cretica]-TRL>   (954) LLIDAAPQEGLDMAS--KSRTKIKDAILS-DNPTQTRL
                             VDD31410.1-[Brassica-oleracea]-TRL>   (955) LLIDAAPQEGLDMAS--KSRTKIKDAILS-DNPTQTRL
       XP_013611745.1-[Brassica-oleracea-var.-oleracea]-T...   (932) LLIDAAPQEGLDMAS--KSRTKIKDAILS-DNPTQTRL
```

PTD5_ AT2G26830.1 (Verified in this study as peroxisomal)

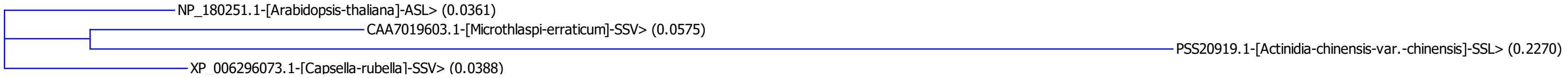


```
                                                              1                                                                                                                     120
            NP_180251.1-[Arabidopsis-thaliana]-ASL>     (1)  MGAAKN---IWALANAEDAAND-------AEQIPYSSFVVDTSLPLPLMIPRIIELCKDLFKNWGELDDSLFSVERVSGGITNLLLKVSVKEDTNKEVSVTVRLYGPNTEYVINREREIL
         CAA7019603.1-[Microthlaspi-erraticum]-SSV>     (1)  MGATKN---IWEILEAEDEASGN-----VASRIPYSSFVVDTSLPLPVMIPRIIQLCKDLFKNWRELDDSLFSVERVSGGITNLLLKVSVKEESDREVSVTVRLYGPNTEYVINRERELL
PSS20919.1-[Actinidia-chinensis-var.-chinensis]-SS...     (1)  MGAVKI------CT-AMEDGSSE---------IPSSSLTVDHTLSLPEMTPRIIELCKDIFKKWSKLDNSHFSVETVSGGITNLLLKVSVREENGSVVDLTIRLYGPNTEYVINRERELQ
            XP_006296073.1-[Capsella-rubella]-SSV>     (1)  MGAAKNNNNIWALANAADGANGNDDAAAAAEQIPYSSSVVDTSLPLPLMIPRIIELCKDLFKNWRDLDDSVFSVERVSGGITNLLLKVSVKEDTDREVSVTVRLYGPNTEYVINREREIL
                                                              121                                                                                                                   240
            NP_180251.1-[Arabidopsis-thaliana]-ASL>   (111)  AIKYLSAAGFGAKLLGGFGNGMVQSFINARTLEPSDMREPKIAAQIARELGKFHKVDIPGSKEPQLWVDILKFYEKASTLTFEEPDKQKLFETISFEELHKEIIELREFTGLLNAPVVFA
         CAA7019603.1-[Microthlaspi-erraticum]-SSV>   (113)  AIKYLSTAGFGAKLLGGFGNGMVQSFINARTLEPSDMRQPKIAAQIAKEVGKFHKVDIPGSKEPQLWVDILKFFEKASTLKFEEPDKQKLFETISFEELHKEIIELREFTGLLNAPVVFA
PSS20919.1-[Actinidia-chinensis-var.-chinensis]-SS...   (105)  AIRYLSAAGFGAKLLGVFGNGMVQSFINARTLVPSDMSQPKLAAEIAKQLRRFHQVEIPGSKEPQLWKDILKFFQKASTLLFDDSEKQTKYETISFDEVYAEVVELKELTDRLNAPVVFA
            XP_006296073.1-[Capsella-rubella]-SSV>   (121)  AIKYLSAAGFGAKLLGGFGNGMVQSFINARTLEPSDMRQPKVAAEIAKELGRFHKVEIPGSKEPQLWFDILKFFEKASTLRFEDPDKQKLFETISFEELRKEIIELREFTGLLNSPVVFA
                                                              241                                                                                                                   360
            NP_180251.1-[Arabidopsis-thaliana]-ASL>   (231)  HNDLLSGNFMLNDEEEKLYLIDFEYGSYNYRGFDIGNHFNEYAGYDCDYSLYPSKEEQYHFIKHYLQPDKPDEVSIAEVESVFVETDAYKLASHLYWAIWAIIQARMSPIEFEYLGYFFL
         CAA7019603.1-[Microthlaspi-erraticum]-SSV>   (233)  HNDLLSGNLMLNEEEEKLYLIDFEYGSYSYRGFDIGNHFNEYAGYDCDYSVYPSKEEQYHFIKHYLQPDKPDEVTMGEVESVYVETDAYKLASHLYWATWAIIQARMSPIDFDYLGYFFL
PSS20919.1-[Actinidia-chinensis-var.-chinensis]-SS...   (225)  HNDLLSGNLMHNEEDEKLYFIDFEYGSYNYRGFDIGNHFNEYAGYDCDYSLYPSKDEQYHFFRHYLEPENPLEVSDKDLEALYIETNTYMLASHLYWALWALIQAKMSPIDFEYLGYFFL
            XP_006296073.1-[Capsella-rubella]-SSV>   (241)  HNDLLSGNLMLNEEEEKLYLIDFEYGSYNYRGFDIGNHFNEYAGYDCDYTLYPSKEEQYHFIKHYLQPDKPNEVSIAEVESVFVETDAYKLASHLYWAIWAIIQARMSPIEFEYLGYFFL
                                                              361                           391
            NP_180251.1-[Arabidopsis-thaliana]-ASL>   (351)  RYNEYKKQKPLTFSLVTSHLSASL-------
         CAA7019603.1-[Microthlaspi-erraticum]-SSV>   (353)  RYNEYKKQKPLTLSLVTSYLSSSSV------
PSS20919.1-[Actinidia-chinensis-var.-chinensis]-SS...   (345)  RYDEYKKQKETCFSLAQSYLSRSDTGQCSSL
            XP_006296073.1-[Capsella-rubella]-SSV>   (361)  RYNEYKKQKPLTFSLVKSHMSASSV------
```

PTD6_ AT1G76540.1 (Verified in this study as peroxisomal)

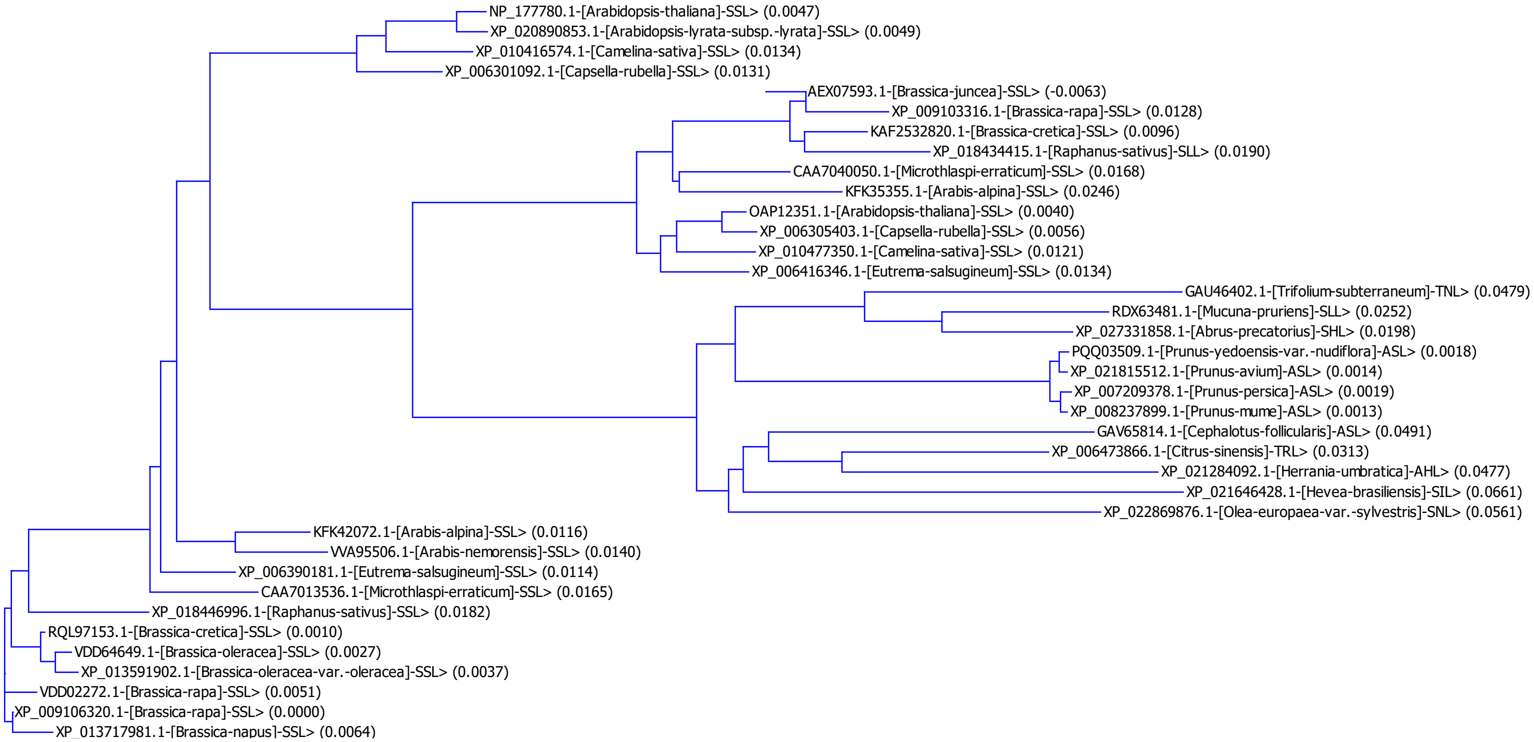

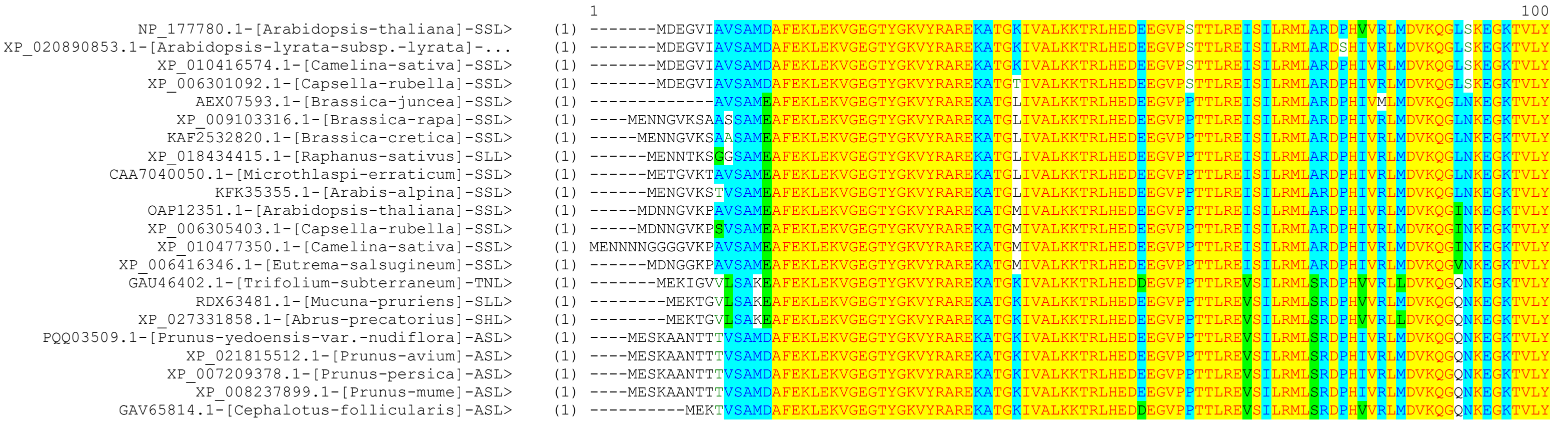

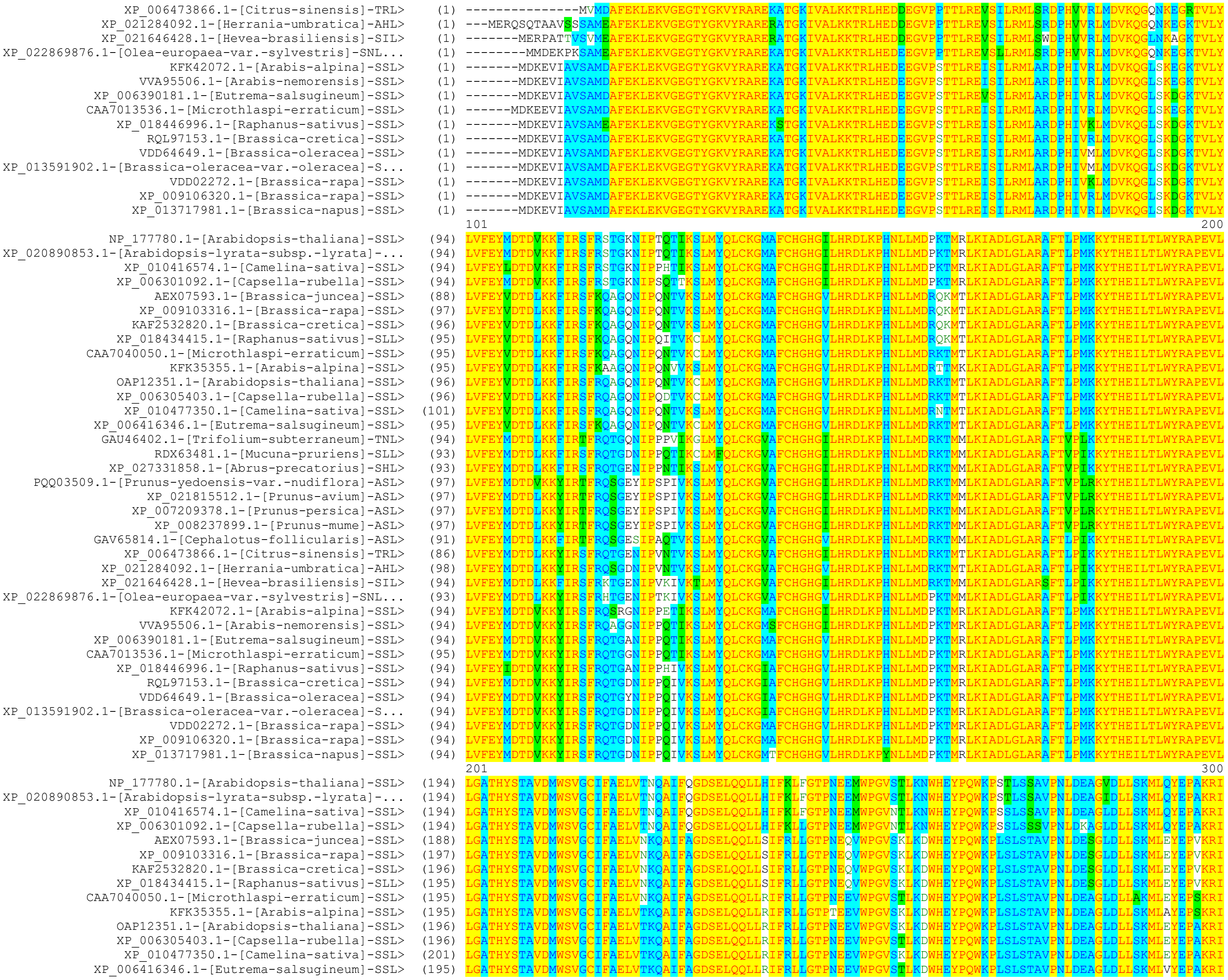

```
                        GAU46402.1-[Trifolium-subterraneum]-TNL>  (194) LGATHYSMAVDIWSVACIFAELVTKQALFPGDSELQQLLHIFRLLGTPNEDMWPGVSKLMNWHEYPQWSPQSLSKAVPTLEEAGLDLLAQMLQYEPAKRI
                             RDX63481.1-[Mucuna-pruriens]-SLL>  (193) LGATHYSMAVDIWSVGCIFAELVTKQALFPGDSELQQLLHIFRLLGTPNEEMWPGVSKLMNWHEYPQWNPQSLTTAVPSLDVLGLDLLSQMLKYEPSKRI
                      XP_027331858.1-[Abrus-precatorius]-SHL>  (193) LGATHYSMAVDMWSVACIFAELVTKQALFPGDSELQQLLHIFRLLGTPNEEMWPGVSKLMNWHEYPQWSPQRLSKAVPNLDELGLDLLSQMLKYEPSKRI
              PQQ03509.1-[Prunus-yedoensis-var.-nudiflora]-ASL>  (197) LGATHYSTAVDIWSVGCIFAELVTKQALFPGDSELQQLLHIFRLLGTPNEEVWPGVSKLMNWHEYPQWSPQSLSKAVPNLDVMGLDLLLQMLQYEPAKRI
                          XP_021815512.1-[Prunus-avium]-ASL>  (197) LGATHYSTAVDIWSVGCIFAELVTKQALFPGDSELQQLLHIFRLLGTPNEEVWPGVSKLMNWHEYPQWSPQSLSKAVPNLDVMGLDLLLQMLQYEPAKRI
                        XP_007209378.1-[Prunus-persica]-ASL>  (197) LGATHYSTAVDIWSVGCIFAELVTKQALFPGDSELQQLLHIFRLLGTPNEEVWPGVSKLMNWHEYPQWNPQSLSKAVPNLDAMGLDLLLQMLQYEPAKRI
                           XP_008237899.1-[Prunus-mume]-ASL>  (197) LGATHYSTAVDIWSVGCIFAELVTKQALFPGDSELQQLLHIFRLLGTPNEEVWPGVSKLMNWHEYPQWSPQSLSKAVPNLDAMGLDLLLQMLQYEPAKRI
                    GAV65814.1-[Cephalotus-follicularis]-ASL>  (191) LGATHYSTSVDMWSVGCIFAELVTKQALFPGDSELQQLLHIFRLLGTPNEQVWPGVSSLMNWHEYPQWSPQRVSSAVPDLDENGEDLLTNMLHYDPSKRI
                       XP_006473866.1-[Citrus-sinensis]-TRL>  (186) LGSTHYSTAVDMWSVACIFAELVTKTALFPGDSELQQLLHIFRLLGTPNEKVWPGVSSLMNWHEYPQWNPQSLATAVPNLDKDGLDLLEQMLQYDPSKRI
                    XP_021284092.1-[Herrania-umbratica]-AHL>  (198) LGATHYSTAVDMWSVACIFAELVTKQALFPGDSELQQLLHIFRLLGTPNEQVWPGVSLLPNWHDYPQWSPQGLSSAVPNLDDDGLHLLSQMLQYDPSKRI
                    XP_021646428.1-[Hevea-brasiliensis]-SIL>  (194) LGATHYSTAVDMWSVGCIFAELVTKQALFPGDSELQQLLHIFRLLGTPNEEVWPGVSNLVNWHEYPQWSPHSMPSAVPNLEKDGLDLLAQMLQYEPSKRI
XP_022869876.1-[Olea-europaea-var.-sylvestris]-SNL...  (193) LGATHYSTAVDMWSVACIFAELVTKQALFPGDSELQQLLHIFRLLGTPNEEVWPGVSKLVNWHEYPQWSPKPLSSAVPGLDEAGRSLLSEMLHYEPSKRI
                            KFK42072.1-[Arabis-alpina]-SSL>  (194) LGATHYSTAVDMWSVGCIFAELVTNQAIFAGDSELQQLLHIFRLLGTPNEEMWPGVSTLKNWHEYPQWKPLSLSSSVPNLDATGLDLLSKMLQYEPAKRI
                        VVA95506.1-[Arabis-nemorensis]-SSL>  (194) LGATHYSTAVDMWSVGCIFAELVTNQAIFAGDSELQQLLHIFRLLGTPNEEMWPGVSTLKNWHEYPQWKPLSLSSSIPNLDEDGLDLLSKMLQYEPAKRI
                  XP_006390181.1-[Eutrema-salsugineum]-SSL>  (194) LGATHYSTAVDMWSVGCIFAELVTNQAIFAGDSELQQLLHIFRLLGTPNEERWPGVSTLKNWHEYPQWKPLSLSSSVPNLDEAGLDLLSKMLQYEPAKRI
                 CAA7013536.1-[Microthlaspi-erraticum]-SSL>  (195) LGATHYSTAVDMWSVGCIFGELVTNQAIFAGDSELQQLLHIFRLLGTPNEEMWPGVSTLKNWHEYPQWKPLSLSTSVPKLDEAGLDLLSKMLQYEPAKRI
                     XP_018446996.1-[Raphanus-sativus]-SSL>  (194) LGATHYSTAVDMWSVGCVFAELVTNQAIFAGDSELQQLLHIFRLLGTPNEEMWPGVSTLKNWHEYPQWKPLSLSTSVPKLDQAGLDLLSKMLQYEPAKRI
                         RQL97153.1-[Brassica-cretica]-SSL>  (194) LGATHYSTAVDMWSVGCVFAELVTNHAIFAGDSELQQLLHIFRLLGTPNEEMWPGVSTLKNWHEYPQWKPLSLSTSVPKLDQAGLDLLSKMLQYEPAKRI
                        VDD64649.1-[Brassica-oleracea]-SSL>  (194) LGATHYSTAVDMWSVGCVFAELVTNHAIFAGDSELQQLLHIFRLLGTPNEEMWPGVSTLKNWHEYPQWKPLSLSTSVPKLDQAGLDLLSKMLQYEPAKRI
XP_013591902.1-[Brassica-oleracea-var.-oleracea]-S...  (194) LGATHYSTAVDMWSVGCVFAELVTNHAIFAGDSELQQLLHIFRLLGTPNEEMWPGVSTLKNWHAYPQWKPLSLSTSVPKLDQAGLDLLSKMLQYEPAKRI
                            VDD02272.1-[Brassica-rapa]-SSL>  (194) LGATHYSTAVDMWSVGCVFAELVTNQAIFAGDSELQQLLHIFRLLGTPNEEMWPGVSTLKNWHEYPQWKPLSLSTSVPKLDQPGLDLLSKMLQYEPAKRI
                        XP_009106320.1-[Brassica-rapa]-SSL>  (194) LGATHYSTAVDMWSVGCVFAELVTNQAIFAGDSELQQLLHIFRLLGTPNEEMWPGVSTLKNWHEYPQWKPLSLSTSVPKLDQAGLDLLSKMLQYEPAKRI
                       XP_013717981.1-[Brassica-napus]-SSL>  (194) LGATHYSTAVDMWSVGCVFAELVTNQAIFAGDSELQQLLHIFRLLGTPNEEMWPGVSTLKNWHEYPQWKPLSLSTSVPKLDQAGLDLLSKMLQYEPAKRI
                                                                  301                320
                   NP_177780.1-[Arabidopsis-thaliana]-SSL>  (294) SAKMAMEHPYFDDLPEKSSL
XP_020890853.1-[Arabidopsis-lyrata-subsp.-lyrata]-...  (294) SAKMAMEHPYFDDLPEKSSL
                      XP_010416574.1-[Camelina-sativa]-SSL>  (294) SAKMAMEHPYFNDLPDKSSL
                     XP_006301092.1-[Capsella-rubella]-SSL>  (294) SAKMAMEHPYFDDLPEKSSL
                         AEX07593.1-[Brassica-juncea]-SSL>  (288) SAKKAMEHPYFDDLPDKSSL
                        XP_009103316.1-[Brassica-rapa]-SSL>  (297) SAKKAMEHPYFDDLPDKSSL
                      KAF2532820.1-[Brassica-cretica]-SSL>  (296) SAKKAMEHPYFDDLPDKSSL
                     XP_018434415.1-[Raphanus-sativus]-SLL>  (295) SAKKAMEHPYFDDLPDKSLL
                 CAA7040050.1-[Microthlaspi-erraticum]-SSL>  (295) SAKKAMEHPYFDDLPDKSSL
                            KFK35355.1-[Arabis-alpina]-SSL>  (295) SAKKAMEHPYFDDLPDKSSL
                    OAP12351.1-[Arabidopsis-thaliana]-SSL>  (296) SAKKAMEHPYFDDLPDKSSL
                     XP_006305403.1-[Capsella-rubella]-SSL>  (296) SAKKAMEHPYFDDLPDKSSL
                      XP_010477350.1-[Camelina-sativa]-SSL>  (301) SAKKAMEHPYFDDLPDKSSL
                  XP_006416346.1-[Eutrema-salsugineum]-SSL>  (295) SAKKAMEHPYFDDLPDKSSL
                        GAU46402.1-[Trifolium-subterraneum]-TNL>  (294) SAKKAMEHPYFDDLDKTNL-
                             RDX63481.1-[Mucuna-pruriens]-SLL>  (293) SAKKAMEHPYFDDLDKSLL-
                      XP_027331858.1-[Abrus-precatorius]-SHL>  (293) SAKKAMEHPYFDDLDKSHL-
              PQQ03509.1-[Prunus-yedoensis-var.-nudiflora]-ASL>  (297) SAKKAMEHPYFDDLNKASL-
                          XP_021815512.1-[Prunus-avium]-ASL>  (297) SAKKAMEHPYFDDLNKASL-
                        XP_007209378.1-[Prunus-persica]-ASL>  (297) SAKKAMEHPYFDDLNKASL-
                           XP_008237899.1-[Prunus-mume]-ASL>  (297) SAKKAMEHPYFDDLNKASL-
                    GAV65814.1-[Cephalotus-follicularis]-ASL>  (291) SAKKAMEHPYFDDLDKASL-
                       XP_006473866.1-[Citrus-sinensis]-TRL>  (286) SAKKAMEHPYFDDLDKTRL-
                    XP_021284092.1-[Herrania-umbratica]-AHL>  (298) SAKKAMEHPYFADLDKAHL-
                    XP_021646428.1-[Hevea-brasiliensis]-SIL>  (294) SAKKAMEHPYFDDLNKSIL-
XP_022869876.1-[Olea-europaea-var.-sylvestris]-SNL...  (293) SAKKAMEHSYFDDLDKSNL-
                            KFK42072.1-[Arabis-alpina]-SSL>  (294) SAKMAMEHPYFDDLPEKSSL
                        VVA95506.1-[Arabis-nemorensis]-SSL>  (294) SAKMAMEHPYFADLPEKSSL
                  XP_006390181.1-[Eutrema-salsugineum]-SSL>  (294) SAKMAMEHPYFDDLPEKSSL
                 CAA7013536.1-[Microthlaspi-erraticum]-SSL>  (295) SAKMAMEHPYFDDLPEKSSL
                     XP_018446996.1-[Raphanus-sativus]-SSL>  (294) SAKMAMEHPYFDDLPDKSSL
                         RQL97153.1-[Brassica-cretica]-SSL>  (294) SAKMAMEHPYFDDLQDKSSL
                        VDD64649.1-[Brassica-oleracea]-SSL>  (294) SAKMAMEHPYFDDLQDKSSL
XP_013591902.1-[Brassica-oleracea-var.-oleracea]-S...  (294) SAKMAMEHPYFDDLQDKSSL
                            VDD02272.1-[Brassica-rapa]-SSL>  (294) SAKMAMEHPYFDDLQDKSSL
                        XP_009106320.1-[Brassica-rapa]-SSL>  (294) SAKMAMEHPYFDDLQDKSSL
                       XP_013717981.1-[Brassica-napus]-SSL>  (294) SAKMAMEHPYFDDLQDKSSL
```

PTD7_ AT3G61960.1 (Verified in this study as peroxisomal)

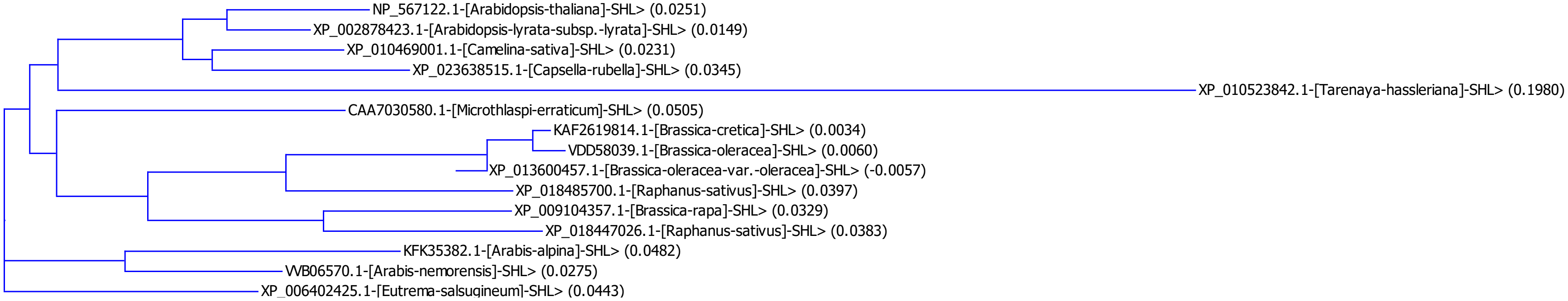


```
                                                           1                                                                                                           110
          NP_567122.1-[Arabidopsis-thaliana]-SHL>     (1) -MESARLVGDYALGPRIGSGSFAVVWLAKHRSSGLEVAVKEIDKKLLSPKVRDNLLKEISILSTIDHPNIIRFYEAIETGDRIFLVLEYCSGGDLAGYINRHGKVPEAVA
XP_002878423.1-[Arabidopsis-lyrata-subsp.-lyrata]-...     (1) -MESARLVGDYELGPRIGSGSFAVVWLAKHRSSGLEVAVKEIDKKLLSPKVRDNLLKEISILSTIDHPNIIRFYEAIETGDRIFLVLEYCSGGDLAGYINRHGKVPEAVA
             XP_010469001.1-[Camelina-sativa]-SHL>     (1) -MESARLVGDYELGPRIGSGSFAVVWLAKHRSSGLEVAVKEIDKKLLSPKVRDNLLKEISILSTIDHPNIIRFYEAIETGDRIFLVLEYCSGGDLAGYINRHGKVPEAVA
            XP_023638515.1-[Capsella-rubella]-SHL>     (1) -MESARLVGDYELGPRIGSGSFAVVWLAKHRSSGFEVAVKEIDKKLLSPKVRDNLLKEISILRTIDHPNIIRFYESIETGDRIYLVLEYCSGGDLAGYINRHGKVPEAVA
        XP_010523842.1-[Tarenaya-hassleriana]-SHL>     (1) -MDEARLVGDYVLGPRIGSGSFAVVWRASHRSSGSEVAVKEIDKKLLSPKVRDNLLKEISILSTIDHPNIIRFHEAIETSDRIYLVLEYCGGGDLAGYIYRHGKVSEHVA
        CAA7030580.1-[Microthlaspi-erraticum]-SHL>     (1) -MDAARLVGDYELGPKIGSGSFAVVWLAKHRSSGLEVAVKEIDKKLLSPKVRDNLLKEISILSTIDHPNIIRFYEAIETGDRIFLVLEYCSGGDLAGYINLHGKVSEAVA
              KAF2619814.1-[Brassica-cretica]-SHL>     (1) --------------------------------------------------------------------------------------------------------------
                 VDD58039.1-[Brassica-oleracea]-SHL>     (1) --------------------------------------------------------------------------------------------------------------
XP_013600457.1-[Brassica-oleracea-var.-oleracea]-S...     (1) -MDTARLVGDYELGPRLGSGSFAVVWLATHRSSGLKVAIKEIDKKKLSPKVRDNLLKEISILSTIDHPNIIRFYEAIETGDRIFLVLEYCSGGDLADYINAHGKVSQPVA
           XP_018485700.1-[Raphanus-sativus]-SHL>     (1) MDEAARLVGDYELGPKLGSGSFAVVWLAKHRSSGLEVAVKEIDKKKLSPKVRDNLLKEISILSTIHHPNIIRFYEAIETGDRIFLVLEYCSGGDLAEYINLHGKVSEPVA
              XP_009104357.1-[Brassica-rapa]-SHL>     (1) MDAAARLVGDYELGRRLGSGSFAVVWLAKHRSSGLEVAIKEIDKKKLNPKVRDSLLKEISILRTIDHPNIIRLHEAIETGDRIFLVLEYCSGGDLAEYINLHGKVSEPVA
           XP_018447026.1-[Raphanus-sativus]-SHL>     (1) MDAAQRLVGDYELGRRLGSGSFAVVWQAKHRSSGSEVAIKEIDKKLLNPKVRDSLLKEISILRTIDHPNIIRLHESIETGDRIFLVLEYCSGGDLADYINLHGKVSEPVA
                 KFK35382.1-[Arabis-alpina]-SHL>     (1) -METTRLVGDYELGPKIGFGSFAVVWLAKHRSSGLEVAVKEIDKKLLSPKVRDNLLKEISILSTIDHPNIIRFYEAIETGDKIFLVLEYCSGGDLAGYINRHGKVQEAVA
             VVB06570.1-[Arabis-nemorensis]-SHL>     (1) -MDATRLVGDYELGPKIGFGSFAVVWLAKHRSSGLEVAVKEIDKKLLSPKVRDNLLKEISILSTIDHPNIIRFYEAIETGDRIFLVLEYCSGGDLAGYINRHGKVPEAVA
         XP_006402425.1-[Eutrema-salsugineum]-SHL>     (1) -MDAARLVGDYELGPKIGSGSFAVVWLAKHRSSGLEVAVKEIDKKLLSPKVRDNLLKEISILSTIDHPNIIRFYEAVETGDRIFLVLEYCSGGDLAGYINLHGKVSEAVA
                                                           111                                                                                                         220
          NP_567122.1-[Arabidopsis-thaliana]-SHL>   (110) KHFMRQLALGLQVLQEKHFIHRDLKPQNLLLSSKEVTPLLKIGDFGFARSLTPESMAETFCGSPLYMAPEIIRNQKYDAKADLWSAGAILFQLVTGKPPFDGNNHIQLFH
XP_002878423.1-[Arabidopsis-lyrata-subsp.-lyrata]-...   (110) KHFMRQLALGLQVLQEKHFIHRDLKPQNLLLSSKEVTPLLKIGDFGFARSLTPESMAETFCGSPLYMAPEIIRNQKYDAKADLWSAGAILFQLVTGKPPFDGNNHIQLFH
             XP_010469001.1-[Camelina-sativa]-SHL>   (110) KHFMRQLALGLQVLQDKHFIHRDLKPQNLLLSSKEVTPLLKIGDFGFARSLTPESMAETFCGSPLYMAPEIIRNQKYDAKADLWSAGAILFQLVTGKPPFDGNNHIQLFH
            XP_023638515.1-[Capsella-rubella]-SHL>   (110) KHFMRQLALGLQVLHEKHFIHRDLKPQNLLLSSKEVTPLLKIGDFGFARSLTPESMAETFCGSPLYMAPEIIRNQKYDAKADLWSAGAILFQLVTGKPPFDGNNHIQLFH
        XP_010523842.1-[Tarenaya-hassleriana]-SHL>   (110) RHFMRQLAAGLQVLQEKHLIHRDLKPQNLLLSTNEVTPLLKIGDFGFARSLTPQNLADTFCGSPLYMAPEIIQNQKYDAKADLWSAGAILYQLVTGKPPFDGNNHIQLFQ
        CAA7030580.1-[Microthlaspi-erraticum]-SHL>   (110) KHFMRQLALGLQVLQEKHYIHRDLKPQNLLLSSKEVTPLLKIGDFGFARSLTPESMAETFCGSPLYMAPEIIRNQKYDAKADLWSAGAILFQLVTGKPPFDGTNHIQLFH
              KAF2619814.1-[Brassica-cretica]-SHL>     (1) -------------------------------------------------------MAETFCGSPLYMAPEIIRNQKYDAKADLWSAGAILFQLVTGKPPFDGTNHIQLFQ
                 VDD58039.1-[Brassica-oleracea]-SHL>     (1) -------------------------------------------------------MAETFCGSPLYMAPEIIRNQKYDAKADLWSAGAILFQLVTGKPPFDGTNHIQLFQ
XP_013600457.1-[Brassica-oleracea-var.-oleracea]-S...   (110) KHFMRQLALGLQVLQEKHCIHRDLKPQNLLLSSKDVTPLLKIGDFGFARSLTPESMAETFCGSPLYMAPEIIRNQKYDAKADLWSAGAILFQLVTGKPPFDGTNHIQLFQ
           XP_018485700.1-[Raphanus-sativus]-SHL>   (111) KHFMRQLALGLQVLQEKHCIHRDLKPQNLLLSSKEVTPLLKIGDFGFARSLTPESMAETFCGSPLYMAPEIIRNQKYDAKADLWSAGAILFQLVTGKPPFDGTNHIQLFQ
              XP_009104357.1-[Brassica-rapa]-SHL>   (111) KHFMRQLALGLQALQEKHCIHRDLKPQNLLLSSKEVTPLLKIGDFGFARSLTPEAMAETFCGSPLYMAPEIIRNRKYDAKADLWSAGAILFQLVTGKPPFDGTNQFQLFH
           XP_018447026.1-[Raphanus-sativus]-SHL>   (111) KHFMRQLALGLQVLQEKHCIHRDLKPQNLLLSSKEVTPLLKIADFGFARSLTPESMADTFCGSPLYMAPEIIRNQKYDAKADLWSAGAILFQLVTGRPPFDGTNQFQLFH
                 KFK35382.1-[Arabis-alpina]-SHL>   (110) KHFMKQLALGLQVLEEKHFIHRDLKPQNLLLSSKEVTPLLKIGDFGFARSLTPESMAETFCGSPLYMAPEIIRNQKYDAKADLWSAGAILFQLVTGKPPFDGTNHMQLFQ
             VVB06570.1-[Arabis-nemorensis]-SHL>   (110) KHFMRQLALGLQVLEEKHFIHRDLKPQNLLLSSKEVTPLLKIGDFGFARSLTPESMAETFCGSPLYMAPEIIRNQKYDAKADLWSAGAILFQLVTGKPPFDGTNHMQLFQ
         XP_006402425.1-[Eutrema-salsugineum]-SHL>   (110) KHFMRQLALGLQVLQEKHFIHRDLKPQNLLLSSKEETPLLKIGDFGFARSLQPQSMAETFCGSPLYMAPEIIQNQKYDAKADLWSAGAILFQLVTGKPPFDGTNHMQLFN
                                                           221                                                                                                         330
          NP_567122.1-[Arabidopsis-thaliana]-SHL>   (220) NIVRDTELKFPEDTRNEIHPDCVDLCRSLLRRNPIERLTFREFFNHMFLREPRQIPDVE---------------HSGFSTCTGKSL-LPSAQPSTSTNRFKSSAENVHKH
XP_002878423.1-[Arabidopsis-lyrata-subsp.-lyrata]-...   (220) NIVRDTELKFPEDARNEIHPDCVDLCRSLLRRNPIERLTFREFFNHKFLREPRQMPDVV---------------HSGSTTSTGKSS-LPSAQPSTSTNRFKSSADNVHKH
             XP_010469001.1-[Camelina-sativa]-SHL>   (220) NIVRDTELKFPKDALNEIHPDCVDLCRSLLRRNPIERLTFREFFTHMFLREPRQVPDVE---------------RSDTTTSTGKSS-LPSAQP-ISTNRFKSSAENAYRH
            XP_023638515.1-[Capsella-rubella]-SHL>   (220) NIVRDTELKFPEDSVKEIHPDCVDLCRSLLRRNPIERLTFREFFTHMFLREPRQVPDVE---------------HSDTTTSMGKSS-LPSAQP-ISTNRLKSSAENVYKH
        XP_010523842.1-[Tarenaya-hassleriana]-SHL>   (220) NIVRGSDLQFPEDPLQEIHLDCIDLCRSLLRRNPIERLTFREFFNHKFLQMPRPSLLDVEQSQSYPTPRAELRGHSDLSTS-GEKSLLHSQQP-GSTSRIKKSVNEQKGE
        CAA7030580.1-[Microthlaspi-erraticum]-SHL>   (220) NIVRDTELKFPEDALNEIHPDCVDLCRSLLRRNPIERLIFREFFNHRFLQEPRQMPVVA---------------RSDSSTTSTGKSLLPSAQP-SSINRFKSSAENVYKH
              KAF2619814.1-[Brassica-cretica]-SHL>    (56) NIVRDTELNFPEDALNEIHPDCVHLCRRLLRRDPIERLTFREFFSHRFLQAPRQMPGVA---------------HSDP-------------------NRSKSSSENVYKH
                 VDD58039.1-[Brassica-oleracea]-SHL>    (56) NIVRDTELKFPEDALNEIHPDCVHLCRRLLRRDPIERLTFREFFSHRFLQAPRQMPGVA---------------HSDP-------------------NRSKSSSENVYKH
XP_013600457.1-[Brassica-oleracea-var.-oleracea]-S...   (220) NIVRDTELKFPEDALNEIHPDCVDLCRRLLRRDPIERLTFREFFSHRFLQAPRQMPGVA---------------HSDP-------------------NRSKSSSENVYKH
           XP_018485700.1-[Raphanus-sativus]-SHL>   (221) NIVRDTELKFPRDGLNEIHPDCVDLCRSLLRRDPIERLTFREFFSHRFLQEPRQMPGVA---------------HSDDSTS---S----SAQPSGGINRSKSSSENVYKH
              XP_009104357.1-[Brassica-rapa]-SHL>   (221) NIVRDTELKFPEDALNEIHPDCVDLCKSLLRRDPIERLTFREFFSHRFLQEARQTPGVA---------------HSDSTTSTGKSS-LPSGQL-TSTNRFKASAENVYKQ
           XP_018447026.1-[Raphanus-sativus]-SHL>   (221) NIVRDTELKFPKDALNEIHPDCVDLCKSLLRRDPIERLTFREFFSHRFLQESRQTP-----------------AAVSTTSTGKSS-LPSGQP-SSTNRFKATAENVYKQ
                 KFK35382.1-[Arabis-alpina]-SHL>   (220) NIVRDTELKFPEASLKEIHPDCVDLCKSLLRRNPIERLSFREFFTHKFFQEPRQMPDVV---------------HSDPTTSKGKSS-LPSAQP-SSTNRVKSTAENVYKQ
```

```
                                   VVB06570.1-[Arabis-nemorensis]-SHL> (220) NIVRDTELKFPQDALNEIHPDCVDLCKSLLRRNPVERLTFREFFTHKFFQEPRQMPGVV---------------YSDSTTSKGKSS-LPSAQP-SCTNRFKSSAENVYKQ
                              XP_006402425.1-[Eutrema-salsugineum]-SHL> (220) NIVRDTELKFPEDASKEIHPDCVDLCRSLLRRNPIERFTFREFFHHKFLQEPRQLPCVE---------------HSDSTTSGKSSSLPSAQP-SSTNRLKPSADNVYKP
                                                                              331                                                                                                        440
                                NP_567122.1-[Arabidopsis-thaliana]-SHL> (314) GSSSS-ASNSQISMPHTS-FEKTRKDTEGQCSSNQSGVVDSLELIEREYVLVNRPSASLEGSSDCFDTSLQDSGFPNILPRNEKVSSSSLEAQKPLSDVSGPRP-ASVSY
       XP_002878423.1-[Arabidopsis-lyrata-subsp.-lyrata]-... (314) GSSSS-APNSQILMPHIS-FEKTRKDTEGQCSSNQSGVVDSLELIEREYVLVNRPSASLEGSSDFFDTSLQDSGFPNILPRNEKVSSSSLEAQRPLSDVSGPRP-TSGSY
                              XP_010469001.1-[Camelina-sativa]-SHL> (313) GSSSS-ASNSQILMPHLS-FEKTRKDTEGQSSSNQFGVVDSLELIEREYVLVNRPSASLEGSSDCFDTSLQDSGLPNFLPRNEKVSSSSLEAQRPLSDVSVPRP-ASGSY
                            XP_023638515.1-[Capsella-rubella]-SHL> (313) GSSSS-APISQNLMPHIS-FEKTRKDTEGQCSSNQSGVVDSLELIEREYVLVNRPSASLEGSSDCFDTSLHNSGLPNFLPRNEKVSLSSLEAQRPLSDASVPRP-ASGSY
                    XP_010523842.1-[Tarenaya-hassleriana]-SHL> (328) TYKNDGGSSSSTSSRHLF--------IEGQCSSNQPPVVDSLELIEREYVLVNRPSVSMEASSECLDTSQQETG---LLP-------SSSLAENPSSNMSGPQP-TTRTY
                    CAA7030580.1-[Microthlaspi-erraticum]-SHL> (314) VNTSG-VSTSHILMPHVVSCEKTKKDTEGQCSSNQFGVFDSLELIEREYVLVNRPSASLEGSSDCFDTSLQDS--TNFLPRN---FKASLEAQRPLPDVSGPPP-ASGSY
                          KAF2619814.1-[Brassica-cretica]-SHL> (132) GSSSS-ASTSNVLMQHDNSCEKTRKDNEGQCSSNQLGVIDSLELIEREYVLVNRPS---EGSSECFDASLQDS----------------VEAQRPLSDVSGPRPETGSSY
                           VDD58039.1-[Brassica-oleracea]-SHL> (132) GSSSS-TSTSNVLMQHDNSCEKTRKDNEGQCSSNQLGVIDSLELIEREYVLVNRPS---EGSSECFDASLQDS----------------VEAQRPLSDVSGPRPETGSSY
   XP_013600457.1-[Brassica-oleracea-var.-oleracea]-S... (296) GSSSS-ASTSNVLMQHDNSCEKTRKDNEGQCSSNQLGVIDSLELIEREYVLVNRPS---EGSSECFDASLQDS----------------VEAQRPLSDVSGPRPETGSSY
                          XP_018485700.1-[Raphanus-sativus]-SHL> (309) ASSSSSASTSNMLMQHDVSCDKTRKDNEGQCSSNPLGVLDSLELIEREYVLVNRPS---EGSSDCFDTSLHKAS------------SSSLEAQRPLPAS-------GGSH
                            XP_009104357.1-[Brassica-rapa]-SHL> (314) GSSSS-ASPSHIVSS-----EKTRKDTEGQSSSNQFGVLDSLELIEREYVLVNRPTE---GSSDCFDTSLQDSGTRNLLPKN---DKGSLEAQRPASG--------S-SY
                          XP_018447026.1-[Raphanus-sativus]-SHL> (311) GISSS-ASTSHVGMPHVS-SEKTRKDTEGQCSSNQFGVLDSLELIEREYVLVSRPS---EGSSDCFDTSLQDSGTRNLLPKN---EKASLEAQRPLSDGSGPRL-ASGSY
                               KFK35382.1-[Arabis-alpina]-SHL> (313) GSSSS-ASSAHTLMPHIS-CEKTRKDTEGQNSLNQFRVFDSLELIEREYVLVSRPS---EGSSDCFDTSLQDSGIPNFLPRNDKASSSSVEPQRPVSDVSGPRP-ATGSY
                           VVB06570.1-[Arabis-nemorensis]-SHL> (313) GSSSS-ASNSQILMPHIS-CEKTRKDTEGQYSSNQFRVFDSLELIEREYVLVNRPSASLEG-SDCFDTSLQDSGIPNFLPRNDKGSSSSLEAQRPLPDVSGPRP-ATGSY
                      XP_006402425.1-[Eutrema-salsugineum]-SHL> (314) GNSSS-ASKSHILMPHVS-CEKTGKDTEGQFSSNQLGVFDSLELIEREYVLVNRPSASLEGSSDCFDTSLQDSGIPN--------DKGSLEAQRPLSGGSGPRP-ASGSY
                                                                              441                                                                                                        550
                                NP_567122.1-[Arabidopsis-thaliana]-SHL> (421) LLTEVQRLTIVHPPTKLQLLHQYAQALTELASEMGNTGQVKESFAVTLVVLAVWRKALEICDSWMMSVG-----------ENEVNPDPTTAPETSIPDLNSPAPAKTWVT
       XP_002878423.1-[Arabidopsis-lyrata-subsp.-lyrata]-... (421) LLTEVQRLTIVHPPTKLQLLHQYAQALTELAREMGNTGQVKESFAVTLVVLAVWRKALEICDSWMISVG-----------ENKVNPDPTTAPEFSIPDLNSPASAKTWVT
                              XP_010469001.1-[Camelina-sativa]-SHL> (420) LLTEVQRLTIVHPPTKLQVLHQYAQVLTELAREMGNTGQVKESFAVTLVVLAVWRKAIEICDSWMLSVGG----------ENRMNPDPTTAPETSIPDLNSPAPAKTWVT
                            XP_023638515.1-[Capsella-rubella]-SHL> (420) LLTEVQRLTIVHPPTKLQHLHQYAQVLTELAGEMGNTGQVKESFAVTLVVLAVWRKALEICDSWMISVG-----------ENSVNPDPIAAPETSIPDLNSPVPAKTWVT
                    XP_010523842.1-[Tarenaya-hassleriana]-SHL> (419) LVTEVQRSTMVHPPTRLKLLHQYAQTLTELAREMYNGGQVRESFAVDLVVLAIWTKALDICDSWMISAGEDLPPETSSRSEHRANQGPSTVPETSGLDFSSPSSAKTRVA
                    CAA7030580.1-[Microthlaspi-erraticum]-SHL> (417) LLTEVQRSTIVHPPTKLQLLHQYAEALSEVAREMGNAGQVKESFAVTLVVLAVWRKALEISDSWMMSVG-----------EEGANTDPTTAPETSIPDLNSPALAKTWVT
                          KAF2619814.1-[Brassica-cretica]-SHL> (222) LLTEVQRLTIVHPPTKLQVLQQYAEALTEVAREMDNAGQVKESFAVTLVVLAAWRKALEICDSWMVSVG--------------EGRVNTTAPGTS----NSPAVAKTWVT
                           VDD58039.1-[Brassica-oleracea]-SHL> (222) LLTEVQRLTIVHPPTKLQVLQQYAEALTEVAREMDNAGQVKESFAVTLVVLAAWRKALEICDSWMVSVG--------------EGRLNTTAPGTS----NSPAVAKTWVT
   XP_013600457.1-[Brassica-oleracea-var.-oleracea]-S... (386) LLTEVQRLTIVHPPTKLQVLQQYAEALTEVAREMDNAGQVKESFAVTLVVLAAWRKALEICDSWMVSVG--------------EGRVNTTAPGTS----NSPAVAKTWVT
                          XP_018485700.1-[Raphanus-sativus]-SHL> (397) LLTEVQRLTIVHPPMKLQLLHQYAEALTQVAREMDNAGQVKESFAVTLVVLAAWRKALEICDSWMVSVG--------------EDRVNTAAPGTSNPDLNSPAVAKTWVT
                            XP_009104357.1-[Brassica-rapa]-SHL> (403) LLTEVQRLTIVHPPTKLQLLHQYAEALAEVAREMGNAGQVKESFAVTLVVLAAWRKALEICDSWMMSVGE----------ERVNPADPTTAPETSNPDLNSPAVAKTWVT
                          XP_018447026.1-[Raphanus-sativus]-SHL> (412) LLTEVQRLTIVHPPTKLQLLHQYAESLAEVAREMGNAGQVKESFAVTLVVLAAWRKALEICDSWMVSVG-----------EERVSPDPTTAPETSNPDLNSPAVAKTWVT
                               KFK35382.1-[Arabis-alpina]-SHL> (417) LLTERQRLTIVHPPTKLQLLHQYAEALTEVASEMGNAGQVKESFAVTLVVLAIWTKALEICDSWMTSVG-----------EGRMNQDPTTAPETSNPDLNSPALAKTWVT
                           VVB06570.1-[Arabis-nemorensis]-SHL> (419) LLTERQRLTILHPPKKLQLLHQYAEALTEVAREMGNAGQVKESFAVTLVVLAVWTKALEICDSWMMTVG-----------EGSVIPDPTTAPETSIPDLNSPALAKTWVT
                      XP_006402425.1-[Eutrema-salsugineum]-SHL> (413) LLTEVQRLTIVHPPTKLQLLYQYAEALTEVAREMGNAGQVKESFAVTLVVLAVWTKALEICDSWMTSVG-----------EDRVNPDPTTAPETSIPDLNSPALAKTWVT
                                                                              551                                                                                                        659
                                NP_567122.1-[Arabidopsis-thaliana]-SHL> (520) QEFVTALNQAENLSTQLNETSA--ATHMPDAMETIYERALAYGKSGGAEEYLSNKESAATLYKKAILLLSFIIEEAVTLSLNPSFSLTPDDKKRILYYISNLQHRRSHL
       XP_002878423.1-[Arabidopsis-lyrata-subsp.-lyrata]-... (520) QEFVTAFNQAENSSTQLNETSA--ATHMPDAMETIYERALAYGKSGGAEEYLSNKESAATLYKKAILLLSFIIEEAVTLSLNPPFSLTPDDKKRILYYISNLQHRRSHL
                              XP_010469001.1-[Camelina-sativa]-SHL> (520) QEFVTAFNQAENTSTQLNETSA--ATHMPDAMETIYERALAYGKSGGAEEYLSNKESAATLYKKAILLLSFIIEEAVTLSLNPPFSLTPDDKKRILYYISNLQHRRSHL
                            XP_023638515.1-[Capsella-rubella]-SHL> (519) QEFVTAFNQAENSSTQLNETSA--ATHMPDAMETIYEKALAYGKSGGAEEYLSNKESAATLYKKAILLLSFIIEEAVTLSLNPPFSLTPDDKKRILYYISNLQHRRSHL
                    XP_010523842.1-[Tarenaya-hassleriana]-SHL> (529) EEFVIAYNQAENSSTQLKETSA--ASHMPDAMETIYEKALTYGKNGGVEEYMNNKASAATLYSKAIVLLSFVVEEAVTLPLSPPFSLTPDDKKRILFYISNLQNRRSHL
                    CAA7030580.1-[Microthlaspi-erraticum]-SHL> (516) QAFVTAFNHAENSSTQLNETSAS-TTHMPDAMETIYEKALAYGKSGGAEEYLNNKESAARLYKKAILLLSFIIEEAVTLSLNPPFSLTLDDKKRILYYISNLQHRRSHL
                          KAF2619814.1-[Brassica-cretica]-SHL> (314) QEFVTAFNQAETSSTRLNQTSAASATHMPDAMETIYEKALAYGKSGGAEEYLNNKESAARLYKKAILLLSFIIEEAVTLPLNPPFSLTPDDKKRILYYISNLQHRRSHL
                           VDD58039.1-[Brassica-oleracea]-SHL> (314) QEFVTAFNQAETSSTRLNQTSAASATHMPDAMETIYEKALAYGKSGGAEEYLNNKESAARLYKKAILLLSFIIEEAVTLPLNPPFSLTPDDRKRILYYISNLQHRRSHL
   XP_013600457.1-[Brassica-oleracea-var.-oleracea]-S... (478) QEFVTAFNQAETSSTRLNQTSAASATHMPDAMETIYEKALAYGKSGGAEEYLNNKESAARLYKKAILLLSFIIEEAVTLPLNPPFSLTPDDKKRILYYISNLQHRRSHL
                          XP_018485700.1-[Raphanus-sativus]-SHL> (493) QEFVTAFNQAETSSARLNETSA--ATHMPDAMETIYDKALAYGKSGGAEEYLNNKESAARLYKKAILLLSFIIEEAVTLPLNPPFSLTPDDKKRILYYISNLQHRRSHL
                            XP_009104357.1-[Brassica-rapa]-SHL> (503) QEFVTAFNQAETSSTQLNQTSA--ATHMPDAMETIYEKALAYGKSGGAEEYLNNKESAGKLYKKAILLLSFIIEEAVTLPLNPPFSLTSDDKKRILYYISNLQHRRSHL
                          XP_018447026.1-[Raphanus-sativus]-SHL> (511) QEFVTAFNQAETSSTQLNETSA--ATHMPDAMETIYEKALAYGKSGGAEEYLNNKESAGRLYKKAILLLSFIIEEAVTLPLNPPFSLTSDDKKRILYYISNLQHRLSHL
                               KFK35382.1-[Arabis-alpina]-SHL> (516) QEFVTAFNQAENISTQLNESSD--ASHMPDAMETIYERALAYGKSGGAEEYLNNKEIAATLYNKAILLLSFIIEEAMTLSLNPPFSLTLDDKKRILYYISNLQHRRSHL
                           VVB06570.1-[Arabis-nemorensis]-SHL> (518) KEFVTAFNQAENSSTQLNETSA--ATHMPDAMETIYERALAYGKSGGAEEYLNNKESAATLYNKAILLLSFIIEEAVTLSLNPPFSLTLDDKKRILYYISNLQHRRSHL
                      XP_006402425.1-[Eutrema-salsugineum]-SHL> (512) QEFVTAFNQAENSSTQLNETSA--ATHMPDAMETVYEKALAYGKSGGAEEYLNNKESAATLYKKAILLLSFIIEEAVTLSLNPPFSLTLDDKKRILYYISNLQHRRSHL
```

PTD8_ AT3G57760.1 (Verified in this study as peroxisomal)

```
                                                                 1                                                                                                  100
          NP_001030879.1-[Arabidopsis-thaliana]-SSI      (1)  MDWLRTKRIRAKKRKRNVKENGEVVLKELIECCDGKCNPIKNFSYDQIIKATNNFCQSNRASRIDVYYRCYKGMLDDRPVLIKKGKYTLDMKEICRDIAI
XP_020881357.1-[Arabidopsis-lyrata-subsp.-lyrata]-...    (1)  MDWLTTKRIGAKTRKRNVKENGEVVLKELIECCDGKCNPIKNFSYDQIIKATDNFSQSNRASRIDVYYRCYKGILDDRPVLIKKGKYTLDMKEICRDIAI
                                                              101                                                                                                 200
          NP_001030879.1-[Arabidopsis-thaliana]-SSI    (101)  SSMVSGHKNFLKLLGCCLEFTPPVLVFEYAEVITLGPLLTSHPGYLRRIKIAREVANALTYLHTAFSRVFIHSNLDPFTIFLDGNGVAKLGNFCNCITIP
XP_020881357.1-[Arabidopsis-lyrata-subsp.-lyrata]-...  (101)  SSMVSGHKNFLKLLGCCLEFTPPVLVFEYAEIITLGPLLVSHPRNLRRIKIAREVANSLTYLHTAFSRVFIHSNLDPFTIFLDGNGVAKLGNFCNCITIP
                                                              201                                                                                                 300
          NP_001030879.1-[Arabidopsis-thaliana]-SSI    (201)  EGETFVHDDTLQKYHELRHNTLKGTHGLGVCNLPVIDPDYKSTGKVTTKTDMHSFGGFMLALVQIREVDDELSLSSDMLRALADLFIKPYDDVRYVHFPL
XP_020881357.1-[Arabidopsis-lyrata-subsp.-lyrata]-...  (201)  EGETFVHDDTLQKYHEFRHITLKGTHGLGVCNLPVIDPDCKSTGNVTTKTDMHSFGAFMLALVQIREVDDELSLSSDMLRALSDLFIKPYDDVRYVRFPL
                                                              301                                                                         378
          NP_001030879.1-[Arabidopsis-thaliana]-SSI    (301)  HHHVSKILRKFGYAEVVDSDMSEVAAWPIKAFLRLALRCIGCKLGDPLSSMIQVTKELRLIEKSAYYPSNNRSQMSSI
XP_020881357.1-[Arabidopsis-lyrata-subsp.-lyrata]-...  (301)  HHHISKILRKFGYAEVVDSDMSEVAVWPVKAFLRLALRCIGCNLGDPLNSMIQVAKELRLIEKSAYYPSNCRQMSTI-
```

PTD9_AT1G29720.1 (Verified in this study as peroxisomal)

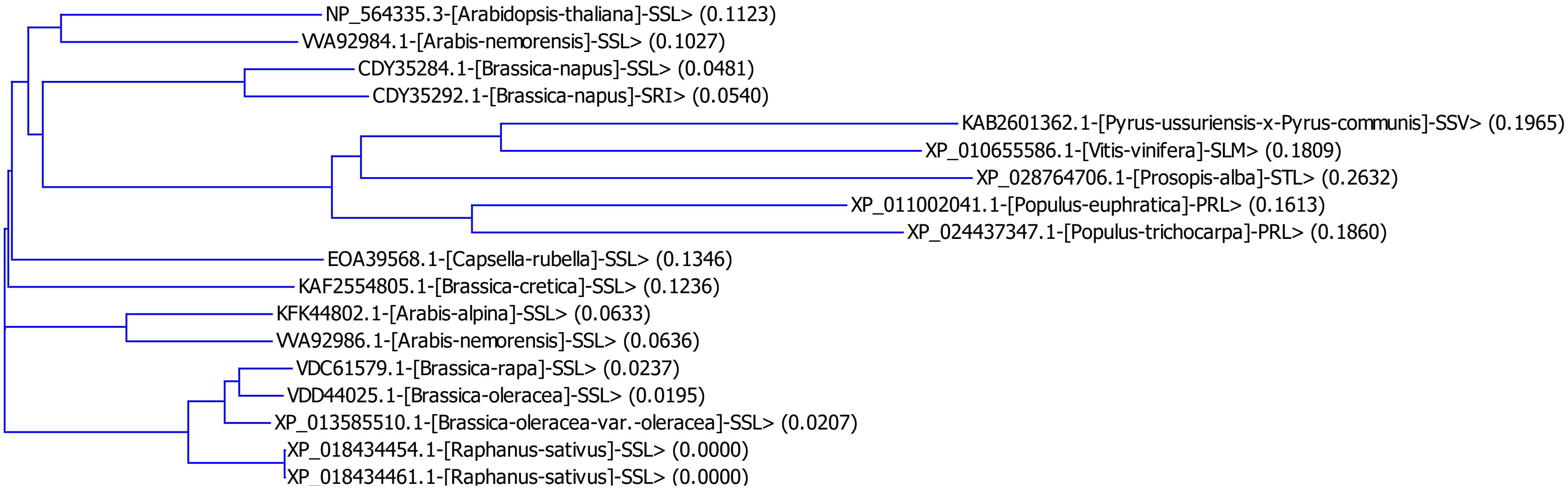


```
                                                              1                                                                                                  100
                       NP_564335.3-[Arabidopsis-thaliana]-SSL>   (1) ------------------MSIILWSFFLFFTIILSSLTNITTLA-SFSSLHADELNALKEIATTLGIKRLNLRDEDPCSSKTLKIIQEVDFVPNLDI-NN
                        VVA92984.1-[Arabis-nemorensis]-SSL>   (1) ------------------MSIVFSKFLLLFTVITSYLATLTTSV-TSPTLHGDELNALKEIATTLGIKGLNLSYGDPCNIRTLKITQKVDFVHNTDI-NN
                           CDY35284.1-[Brassica-napus]-SSL>   (1) ------------------MGVVFSALVLFFTIITSFSSTLTTS--ASPALHPDELKALGEIATTLGIKRLNLSDGDPCLLKTLKLGVVSNPDSDVEN---
                           CDY35292.1-[Brassica-napus]-SRI>   (1) ------------------MWVVFWALLLFFIIITSFFATLTTS--ASPALHADELHALGEITTTLGIKRLNLSDGDPCFLKTLKLGGGVSNVVNMPVNN-
KAB2601362.1-[Pyrus-ussuriensis-x-Pyrus-communis]-...   (1) ------------MAASQNLSKLFFCCVVLLTVLICIEPIKSQE--QVGTLADDEVEALREIAVQLNKKDWN-----FSDPCSNIPTISIPHADQYNN---
                      XP_010655586.1-[Vitis-vinifera]-SLM>   (1) ------------MQMLNLNILRFFFNKCLLLLLSVAVLSCFRPATGAQLPAYELDALSEIASQLGKKDWN----FSLNPCDGNSNWSTPIITEKPLYGN
                      XP_028764706.1-[Prosopis-alba]-STL>   (1) ----------------------MQAANFFELLLVALTCFQFLGISHESKLPQDEVHALQEITRTMGASYWKIN---DSCGIDTVDVTQELSPGSERR---
                  XP_011002041.1-[Populus-euphratica]-PRL>   (1) MKTYSSFFPTRAPNMFTMLQPPRGLLQFCRVMVVVITLFSSATLVVSYQLHPDEVTALKQIGKTVLTQG-----GQPLELGDACIQDSEANSTTLCN---
                 XP_024437347.1-[Populus-trichocarpa]-PRL>   (1) ---------------------MLMMFQPCQVMVILISFSSSITLLASDQLHPGEVAALRQIGKTVNEDGQLSLKLVDSCQQKGVVETELNSAPPNLEGNS
                        EOA39568.1-[Capsella-rubella]-SSL>   (1) ------------------MLIGFSVLLLFFTIITIFFAIPTS---VLSTLHPDELNTLEKIATTLGIKEFNLRNGDPCSLGTLTMMQQVTSVSNPKMKN-
                       KAF2554805.1-[Brassica-cretica]-SSL>   (1) ----------------------------------------------------------------------------------------------------
                          KFK44802.1-[Arabis-alpina]-SSL>   (1) ------------------MLIVLSVLLLFFTIITSFFAISTP-----LALHPDEFKALEKIGTTLGIKGLDLSYEDPCISGTLKMIPEVAVVSNPEIRNN
                        VVA92986.1-[Arabis-nemorensis]-SSL>   (1) ---------------------------MFFTIIISFFAISVTS--VLSALHPDELKALEKIGTTLGIKGLNLSYEDPCISGTLKMIPEVAVVSNPEIRNS
                          VDC61579.1-[Brassica-rapa]-SSL>   (1) ----------------MSIMGIFFSALLCFTIITSFSVIQIQTTSTSPALYSDELNALEKIATTLGIKRVNLSHEDPCSSGTLMITQQVDFVWNPEV-NN
                       VDD44025.1-[Brassica-oleracea]-SSL>   (1) ------------------MMWIVFSALLFFTIITSFSVIQTTS--TSPALHPDELNALEKIATALGIKRVNLSHEDPCSSGTLMITQQVDFVWNPEV-NN
XP_013585510.1-[Brassica-oleracea-var.-oleracea]-S...   (1) -------------------MLIVFSVLLFFIIITSFLASSTS---VSPSLHSDELNALEKIATTLGIKGLNLSYGDPCSLGTLKMMQDVDVISNPDTGNS
                   XP_018434454.1-[Raphanus-sativus]-SSL>   (1) -------------------MLIVFSVLLFFIIITSFLASSTS---ASPSLHSDELNALEKIATTLGIKGLDLSYGDPCSLGTLKMMQDVDVETGNST---
                   XP_018434461.1-[Raphanus-sativus]-SSL>   (1) -------------------MLIVFSVLLFFIIITSFLASSTS---ASPSLHSDELNALEKIATTLGIKGLDLSYGDPCSLGTLKMMQDVDVETGNST---
                                                              101                                                                                                200
                       NP_564335.3-[Arabidopsis-thaliana]-SSL>  (81) TIGCDCSFNNNTICRITELALKTMSLRGKLPPELTKLPYLKSIELCRNYLSGTIPMEWAKMAYLTSISVCANNLSGNLPAGLQNFKNLTFLGVEGNQFSG
                        VVA92984.1-[Arabis-nemorensis]-SSL>  (81) TILCDCSFNNNTICHITEIFLKTMGLPGKLPPQLVKLPYLRSIDLCRNYLSGTIPTEWASLPNLTYISLCANNLSGKLPIGLQKFKNLTFLGLEANRFSG
                           CDY35284.1-[Brassica-napus]-SSL>  (78) IILCDCSFNNNMTCHIIELILKSVSLSGKLPPELAKLQHLQKINLCRNYLSGSIPMEWASMPNLTSISFCANRLSGPLPPGLQNFKSLKFLGVEANQFSG
                           CDY35292.1-[Brassica-napus]-SRI>  (80) -IVCDCSFNNNMTCHITEIFLKSTSLPGKLPPELAKLRYLQKIDLCRNYLSGSIPMEWASMPHLTFISLCANNLSGPLPPGLQNFKNLTFLGVEANQFSG
KAB2601362.1-[Pyrus-ussuriensis-x-Pyrus-communis]-...  (79) TLVCNCSFSGNV-CHIESIFLMGQDLDGVLPPALSKLPYIKQVNLGQNDLSGSIPREWNS-TKLEFLVVSVNNLSGPIPSYLGSMTTLQALGLESNLFSG
                      XP_010655586.1-[Vitis-vinifera]-SLM>  (85) NVSCNCSYPNGE-CHVVNITLKGQDLAGVLPPSLVKLPYLEMIDLTRNYLSGDIPPQWAS-TKLEILSISMNRFSGPIPKFFGNITTLKYLGFEGNFFSG
                      XP_028764706.1-[Prosopis-alba]-STL>  (73) -LECDCKFENNTVCHVVKMTLKRLSLPGMLPAQLVKLPYLQEIDFAYNLFTGTIPKEWAS-MNLTSISLLVNRLSGEIPKHLGNITSLTYLMLEANQFSG
                  XP_011002041.1-[Populus-euphratica]-PRL>  (93) ---CTLNLNNDSYCHITSLSLKTLSLQGKLPFETVNLTYLKYLDLTRNYISGNIPEEWASMKHLTYLSLTSNHLSGNIPWYLGSFRSLTYLSLEANQFSG
                 XP_024437347.1-[Populus-trichocarpa]-PRL>  (80) TIGCNCSITDDNYCHITSFQLKDYSLPGRLPPELANLTYVQKIDFTRNYLYGTIPVEWASMKNLSFISLTANRLSGNIPGHLGSFTALTYLSLESNQFSG
                        EOA39568.1-[Capsella-rubella]-SSL>  (79) AIGCDCSLYNNDTCHITSIDLKTLSLPGILPPELADLRYLQRIELCRNYLSGSIPMEWASMPHLTTISVCANNLSGKLPPGLQNFKNLTSLGIEANQFSG
```

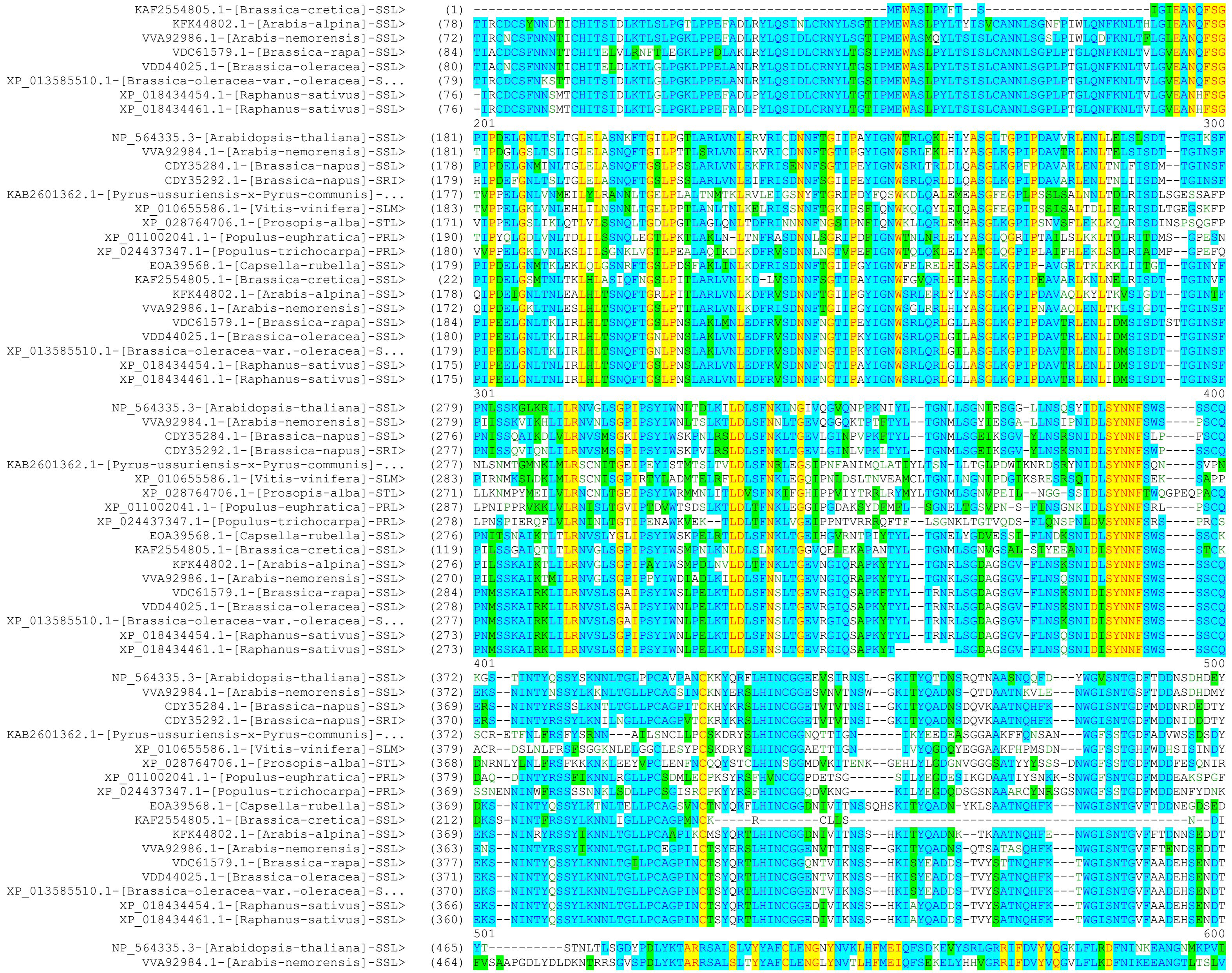

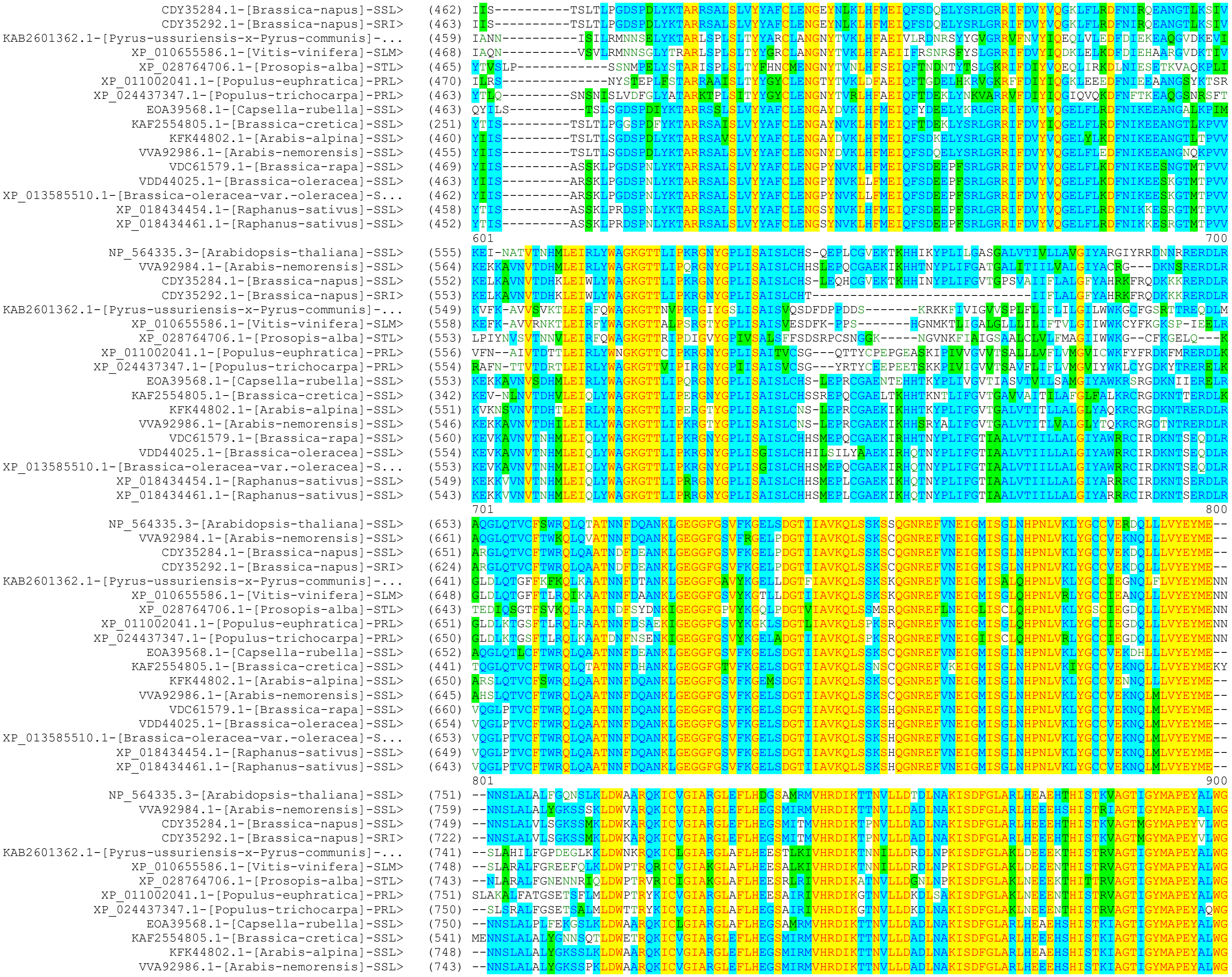

```
VDC61579.1-[Brassica-rapa]-SSL>                           (758) --NNSLAHMLHEKSSLNLDWKARQKICVGIARGLEFLHEGSMIRMVHRDIKTTNVLLDADLNAKISDFGLARLHEEEHSHISTKIAGTIGYMAPEYALWG
VDD44025.1-[Brassica-oleracea]-SSL>                       (752) --NNSLAHMLHGKSSLNLDWKARQKICVGIARGLEFLHEGSMIRMVHRDIKTTNVLLDADLNAKISDFGLARLHEEEHSHISTKIAGTIGYMAPEYALWG
XP_013585510.1-[Brassica-oleracea-var.-oleracea]-S...     (751) --NNSLAHMLHGKSSLNLDWKARQKICVGIARGLEFLHEGSMIRMVHRDIKTTNVLLDADLNAKISDFGLARLHEEEHSHISTKIAGTIGYMAPEYALWG
XP_018434454.1-[Raphanus-sativus]-SSL>                    (747) --NNSLSHMLHGKCSVYLDWKARQKICVGIARGLEFLHEGSMIRMVHRDIKTTNVLLDADLNAKISDFGLARLYEEEHSHISTKIAGTIGYMAPEYALWG
XP_018434461.1-[Raphanus-sativus]-SSL>                    (741) --NNSLSHMLHGKCSVYLDWKARQKICVGIARGLEFLHEGSMIRMVHRDIKTTNVLLDADLNAKISDFGLARLYEEEHSHISTKIAGTIGYMAPEYALWG
                                                                901                                                                                            1000
NP_564335.3-[Arabidopsis-thaliana]-SSL>                   (849) QLTEKADVYSFGVVAMEIVSGKSNTKQQGNADSVSLINWALTLQQTGDILEIVDRMLEGEFNRSEAVRMIKVALVCTNSSPSLRPTMSEAVKMLEGEIEI
VVA92984.1-[Arabis-nemorensis]-SSL>                       (857) QLTEKADVYSFGVVAMEIVSGKSNMKQKGSADHFSLINWALTLQQTGDIMEIVDPMLEGDFNSKEAVRMIKVALVCTNSSPSLRPTMSEVVQMLEGELDI
CDY35284.1-[Brassica-napus]-SSL>                          (847) QLTEKADVYSFGVVAMEIVSGKSNTKHKGTADHISLLDWALSLHQKGNILEVVDPVLEGHFNRKEAVRMINVALVCTNSSPALRPTMSEAVKMLEGVIEL
CDY35292.1-[Brassica-napus]-SRI>                          (820) QLTEKADVYSFGVVAMEIVSGKSNTKHKGTADHISLLDWALSLHQKGNILEVVDPVLEGHFNRKEAVRMINVALVCTNSSPALRPTMSEAVKMLEGVIEL
KAB2601362.1-[Pyrus-ussuriensis-x-Pyrus-communis]-...     (839) YLSDKADVYSFGVVALELVSGKNNIKYRPNENFVCLLDWALVLQQNGHLMKLVDRKLGSEFNKEEAMRMIKVALLCANPSPALRPSMSAVVSMLEGQTLV
XP_010655586.1-[Vitis-vinifera]-SLM>                      (846) YLTYKADVYSFGVVALEIVAGKNNMKYRPNEDYFSLLDWAFFLQQKGNLMELVDPKLESDFNKEEVLRMIKISLLCTNPSPALRPTMSAVVNMLEGRAPV
XP_028764706.1-[Prosopis-alba]-STL>                       (841) HLTYKADVYSFGVLALEIVTGKSNNNYVPDNGAVCLLEWACQLQQSGKLMELVDERMGSMGDEREAEKVLKVALLCTNASPSLRPAMSEVINMLEGRMDI
XP_011002041.1-[Populus-euphratica]-PRL>                  (851) YLTDKADVYSFGVVALEIVSGRSNSSYRTTNEFVCLLDWAHGLQKKRNLMEIVDPKLQSEFNKEEAERMIKLALLCTNASPSLRPAMSEVVSMLEGQTTI
XP_024437347.1-[Populus-trichocarpa]-PRL>                 (848) YLTDKADVYSFGVVALEIVSGKSNSSYRPENENVCLLDWAHVFQKKENLMEIVDPKLHSEFNKEEAERMIKAALLCTNASPSIRPAMSEVVSMLEGQTSI
EOA39568.1-[Capsella-rubella]-SSL>                        (848) QLTEKADVYSFGVVAMEIVSGKSNTKQQGN---VSLINWALTLQQTGDIMDIVDAMLKGEFNNNEAARMIKVALVCINSSPSLRPTMSEVVQMLEGEMEI
KAF2554805.1-[Brassica-cretica]-SSL>                      (641) QLTEKADVYSFGVVAMEIVSGKSNVKPQGNDDHVSLINWALALHQTGDAMEVVDPVLQGDFNSKEAVRMIEVALVCTNSSPALRPTMLEAMQMLEGEMEI
KFK44802.1-[Arabis-alpina]-SSL>                           (846) QLTEKADVYSFGVVAMEIVSGKSNMQQKGGDDNVSLINWALTLHQKGDIMEIVDPMLEGDFNSKEAMRMINVALVCTNSSPSLRPTMSEAVQMLEGEIEI
VVA92986.1-[Arabis-nemorensis]-SSL>                       (841) HLTEKADVYSFGVVAMEIVSGKSNVTKKGGNDSFSLINWALTLHQKGDILEIVDPMLEGEFNSNEAVRMINVALVCTNSSPSLRPSMSEAVQMLEGEMEI
VDC61579.1-[Brassica-rapa]-SSL>                           (856) QLSEKADVYSFGIVAMEIVSGQSNTKQKGSADHVSLINWAVKLQQKGDITEIVDPVLQGDFNTKEAARMIKVAIVCTNSSPSLRPTMSEVVQMLEGEVEI
VDD44025.1-[Brassica-oleracea]-SSL>                       (850) QLSEKADVYSFGIVAMEIVSGQSNTKQKGSADHVSLINWAVKLQQKGDITEIVDPVLQGDFNTKEAVRMIKVAIVCTNSSPSLRPTMSEVVQMLEGEVEI
XP_013585510.1-[Brassica-oleracea-var.-oleracea]-S...     (849) QLSEKADVYSFGIVAMEIVSGQSNTKQKGSADHVSLINWAVKLQQKGDITEIVDPVLQGDFNTKEAVRMIKVAIVCTNSSPSLRPTMSEVVQMLEGEVEI
XP_018434454.1-[Raphanus-sativus]-SSL>                    (845) QLSEKADVYSFGVVAMEIVSGQSNSKYKGSDDQVSLINWAVKLQQKGDLTEIVDPVLQGDYNSNEAVRMIKVAIVCTNSSPSLRPTMSEAVQMLEGEVEI
XP_018434461.1-[Raphanus-sativus]-SSL>                    (839) QLSEKADVYSFGVVAMEIVSGQSNSKYKGSDDQVSLINWAVKLQQKGDLTEIVDPVLQGDYNSNEAVRMIKVAIVCTNSSPSLRPTMSEAVQMLEGEVEI
                                                                1001                                                                                           1100
NP_564335.3-[Arabidopsis-thaliana]-SSL>                   (949) TQVMSDPGIYGHDWSISKLRDIDTHSSSSTSGVT---DQTTTTMKS---SVSGCDLYPLYPESMILNSTVENSSSSSL-----------------------
VVA92984.1-[Arabis-nemorensis]-SSL>                       (957) TQVMSDPGLYGHDLSISKLRDNDTHGSSSTSGVT---DQTTTS-KS---SVSGNDLYPLYPESAILFSTVESSSSSSL-----------------------
CDY35284.1-[Brassica-napus]-SSL>                          (947) TQVSSDPGIYGDDWSLLKLRDIDDTHGSSSTSGVT--DQTRTTTKS---SVSGCDLYPLYPESMTLNSTVEYHSSSL-----------------------
CDY35292.1-[Brassica-napus]-SRI>                          (920) TQVSSDPGIYGDDWSLLKLRDIDDTHGSSSTSVHALEEIAITLGIK---RVNLSYKDPCGSRSLMVIKQVDWNPEVNNTIACDCSFNNNMTCHITELTLV
KAB2601362.1-[Pyrus-ussuriensis-x-Pyrus-communis]-...     (939) QEVKINPSIYGDELRLAFSEDSDTYTEQSTRTLP----------------YSSDAKWTASTSSSV-----------------------------------
XP_010655586.1-[Vitis-vinifera]-SLM>                      (946) QEFPLNPIIFGDEALRSQYSQMHFHRSSETETIKHSSDSTGIGSPS----TSTRDLHQINPDSLM-----------------------------------
XP_028764706.1-[Prosopis-alba]-STL>                       (941) PEAVPEAGAYNEDLRFKALRDLDQWSRQSLNGSQ----TQSSVSLHTFSLESVNTLTGCSTDHQLVHKSEISPSKASSTL--------------------
XP_011002041.1-[Populus-euphratica]-PRL>                  (951) QEMISDPSIYGDDLHSKRLEGHYQQVMDQSLNST-------------------QDLFPPSDKSWIGNSSTSAHVKPRL----------------------
XP_024437347.1-[Populus-trichocarpa]-PRL>                 (948) PEVTSDPSIFYDDLHSKRVNGHYQQVTDQSLNSTQDLFPPSDKSWIGNSSTSTPELYPINPVSISLNLVKPRL---------------------------
EOA39568.1-[Capsella-rubella]-SSL>                        (945) PQVMSGPGLYGPNWSISKLKDIDTHGSSSKFGVT---DHQTTTVKS---SASGSNLYPLYPESMILNSTIEFSSSSL-----------------------
KAF2554805.1-[Brassica-cretica]-SSL>                      (741) TPVMSDHGLYEHNLSISKMRVTTTIGSSSTSGVT---NQTATTMKS---DASGCDIYPLYPESMILNSTSDLSSSSL-----------------------
KFK44802.1-[Arabis-alpina]-SSL>                           (946) TQVMSDPSMYGHDVSFSKLMMENANSHTSTKAGVS-GNEISGTTTM---TSSVSDMYPLYPESFILNSTTEISSSSL-----------------------
VVA92986.1-[Arabis-nemorensis]-SSL>                       (941) TEVMSDPSMYGHDFSFSNLKIANSHALGNSNMCGVSGNEISATTTT---TSSVSDMYPLYPESMILNSTTEISSSSL-----------------------
VDC61579.1-[Brassica-rapa]-SSL>                           (956) TQVLSDPGLYGHNWSISNLRDIDTDGGLSTSVVT---DQTATTMKS---SVSGCDLYPLYPASMILNSTVDFSSSSL-----------------------
VDD44025.1-[Brassica-oleracea]-SSL>                       (950) TQVLSDPGLYGHNWSISNLRDIDTDGGLSTSVVT---DQTATTMKS---SVSGCDLYPLYPESMILNSTVDFSSSSL-----------------------
XP_013585510.1-[Brassica-oleracea-var.-oleracea]-S...     (949) TQVLSDPGLYGHNWSISNLRDIDTDGGLSTSVVT---DQTATTMKS---SVSGCDLYPLYPESMILNSTVDFSSSSL-----------------------
XP_018434454.1-[Raphanus-sativus]-SSL>                    (945) TQVISDRGLYGHNWSISNLRDIDTDGILSTSGVT---DQTATTMKS---SVTGCDLYPLYPESMTLISTVERSSSSL-----------------------
XP_018434461.1-[Raphanus-sativus]-SSL>                    (939) TQVISDRGLYGHNWSISNLRDIDTDGILSTSGVT---DQTATTMKS---SVTGCDLYPLYPESMTLISTVERSSSSL-----------------------
                                                                1101                                                                                           1200
NP_564335.3-[Arabidopsis-thaliana]-SSL>                  (1020) ----------------------------------------------------------------------------------------------------
VVA92984.1-[Arabis-nemorensis]-SSL>                      (1027) ----------------------------------------------------------------------------------------------------
CDY35284.1-[Brassica-napus]-SSL>                         (1019) ----------------------------------------------------------------------------------------------------
CDY35292.1-[Brassica-napus]-SRI>                         (1017) NLTLVGKVPPELAKLRHLRSTNITKVSYEFFSLTCSDLSANYLTGTIPPEWVSMPYLTFMLAPSLCLTLMLHLHDLRITLNILAAGSAGIVCLGIYQLGC
KAB2601362.1-[Pyrus-ussuriensis-x-Pyrus-communis]-...     (988) ----------------------------------------------------------------------------------------------------
XP_010655586.1-[Vitis-vinifera]-SLM>                     (1007) ----------------------------------------------------------------------------------------------------
XP_028764706.1-[Prosopis-alba]-STL>                      (1017) ----------------------------------------------------------------------------------------------------
XP_011002041.1-[Populus-euphratica]-PRL>                 (1010) ----------------------------------------------------------------------------------------------------
XP_024437347.1-[Populus-trichocarpa]-PRL>                (1021) ----------------------------------------------------------------------------------------------------
EOA39568.1-[Capsella-rubella]-SSL>                       (1016) ----------------------------------------------------------------------------------------------------
KAF2554805.1-[Brassica-cretica]-SSL>                      (812) ----------------------------------------------------------------------------------------------------
KFK44802.1-[Arabis-alpina]-SSL>                          (1019) ----------------------------------------------------------------------------------------------------
VVA92986.1-[Arabis-nemorensis]-SSL>                      (1015) ----------------------------------------------------------------------------------------------------
VDC61579.1-[Brassica-rapa]-SSL>                          (1027) ----------------------------------------------------------------------------------------------------
VDD44025.1-[Brassica-oleracea]-SSL>                      (1021) ----------------------------------------------------------------------------------------------------
XP_013585510.1-[Brassica-oleracea-var.-oleracea]-S...    (1020) ----------------------------------------------------------------------------------------------------
XP_018434454.1-[Raphanus-sativus]-SSL>                   (1016) ----------------------------------------------------------------------------------------------------
XP_018434461.1-[Raphanus-sativus]-SSL>                   (1010) ----------------------------------------------------------------------------------------------------
                                                                1201
NP_564335.3-[Arabidopsis-thaliana]-SSL>                  (1020) -----
VVA92984.1-[Arabis-nemorensis]-SSL>                      (1027) -----
CDY35284.1-[Brassica-napus]-SSL>                         (1019) -----
CDY35292.1-[Brassica-napus]-SRI>                         (1117) KTSRI
KAB2601362.1-[Pyrus-ussuriensis-x-Pyrus-communis]-...     (988) -----
```

```
                   XP_010655586.1-[Vitis-vinifera]-SLM>  (1007) -----
                    XP_028764706.1-[Prosopis-alba]-STL>  (1017) -----
               XP_011002041.1-[Populus-euphratica]-PRL>  (1010) -----
              XP_024437347.1-[Populus-trichocarpa]-PRL>  (1021) -----
                     EOA39568.1-[Capsella-rubella]-SSL>  (1016) -----
                   KAF2554805.1-[Brassica-cretica]-SSL>   (812) -----
                        KFK44802.1-[Arabis-alpina]-SSL>  (1019) -----
                    VVA92986.1-[Arabis-nemorensis]-SSL>  (1015) -----
                        VDC61579.1-[Brassica-rapa]-SSL>  (1027) -----
                    VDD44025.1-[Brassica-oleracea]-SSL>  (1021) -----
XP_013585510.1-[Brassica-oleracea-var.-oleracea]-S...  (1020) -----
                 XP_018434454.1-[Raphanus-sativus]-SSL>  (1016) -----
                 XP_018434461.1-[Raphanus-sativus]-SSL>  (1010) -----
```

PTD10_ AT5G51560.1 (Verified in this study as peroxisomal)

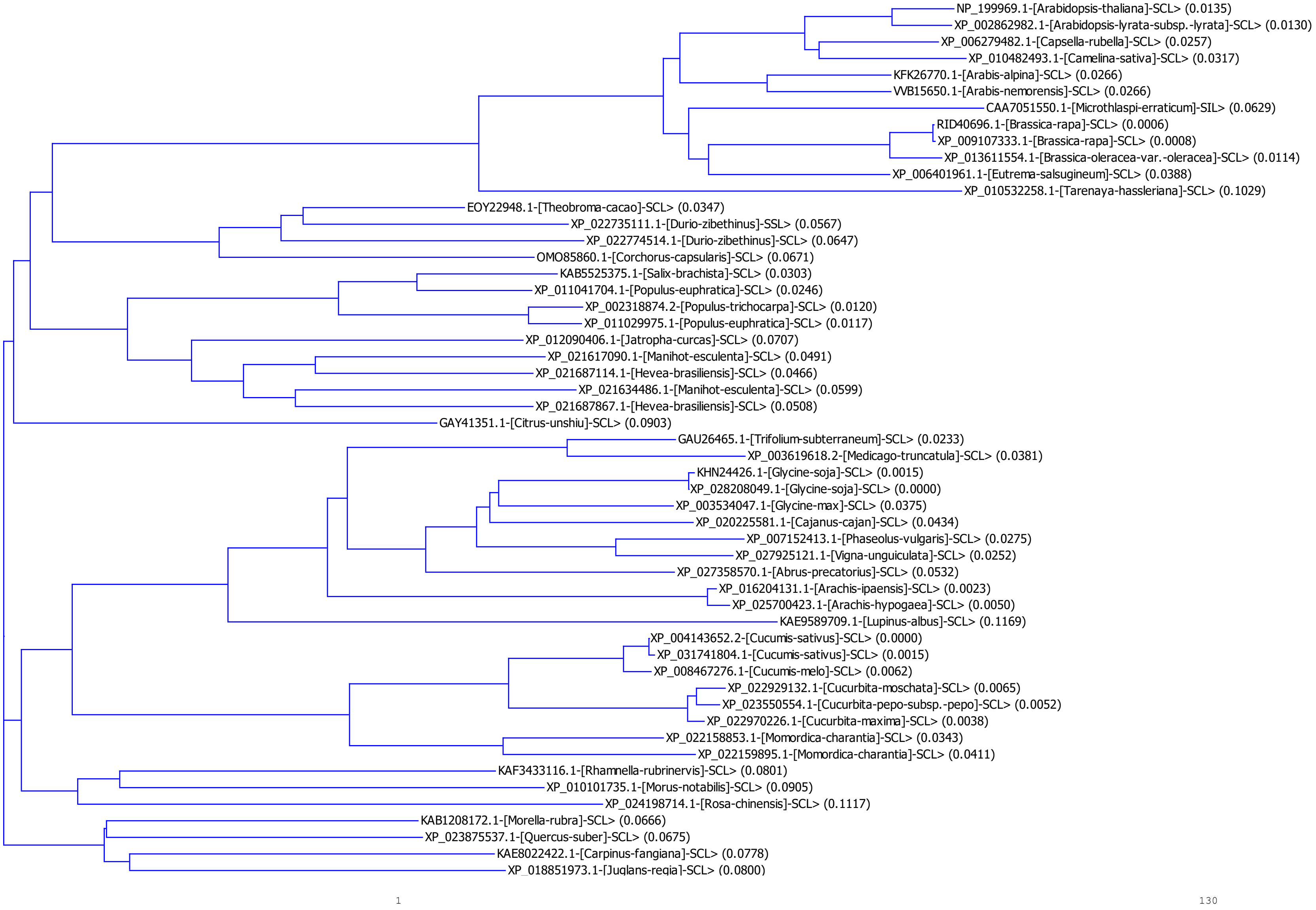

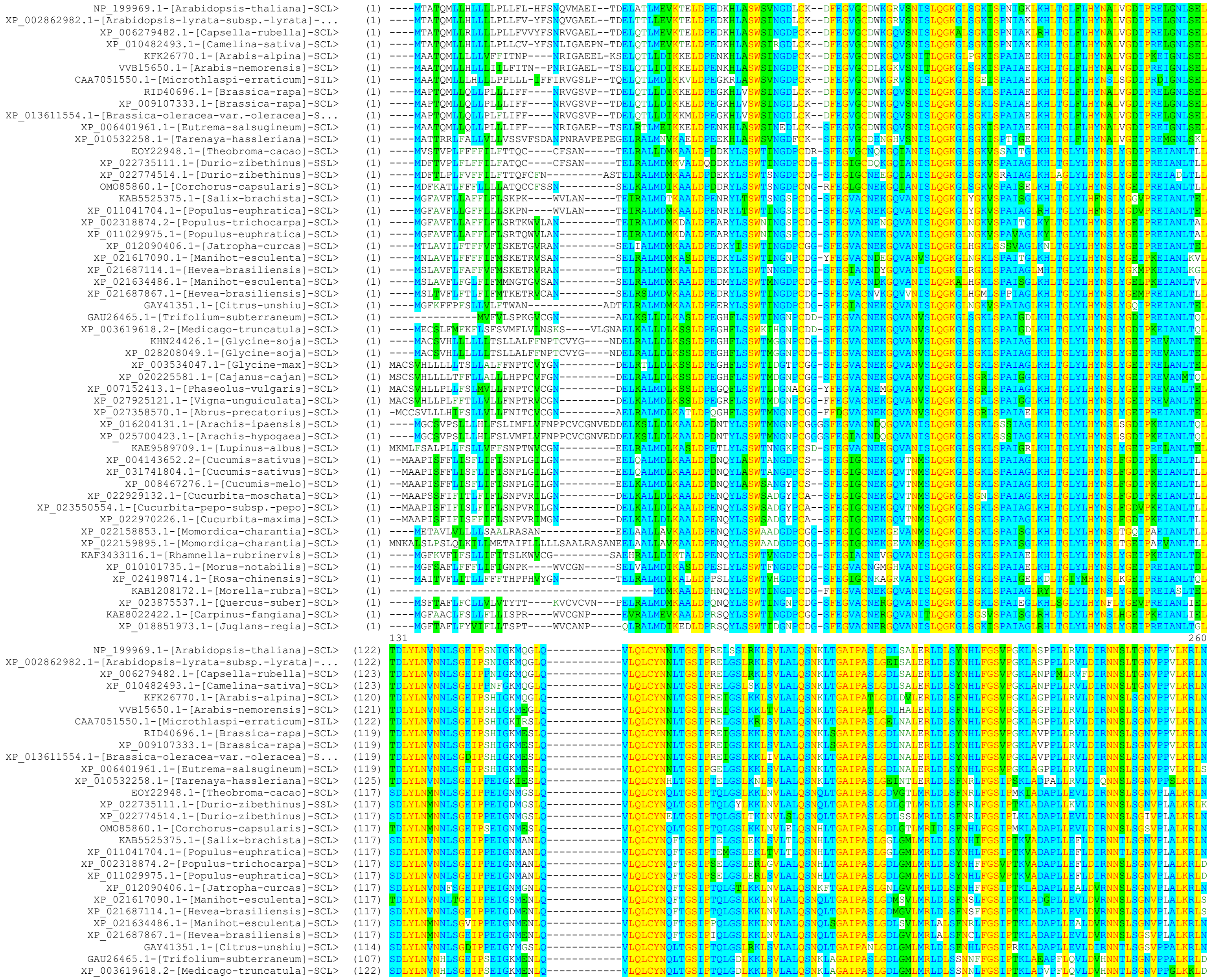

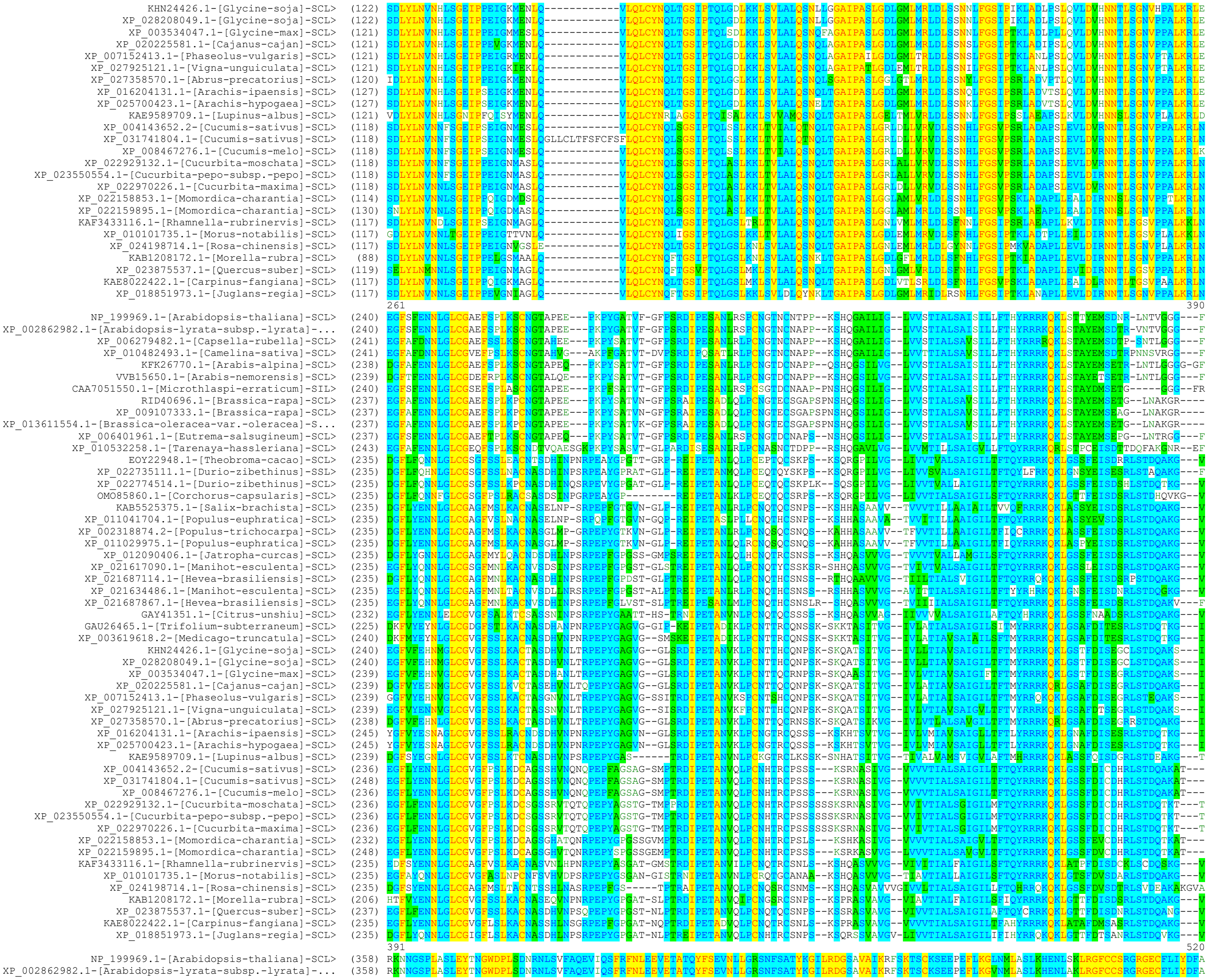

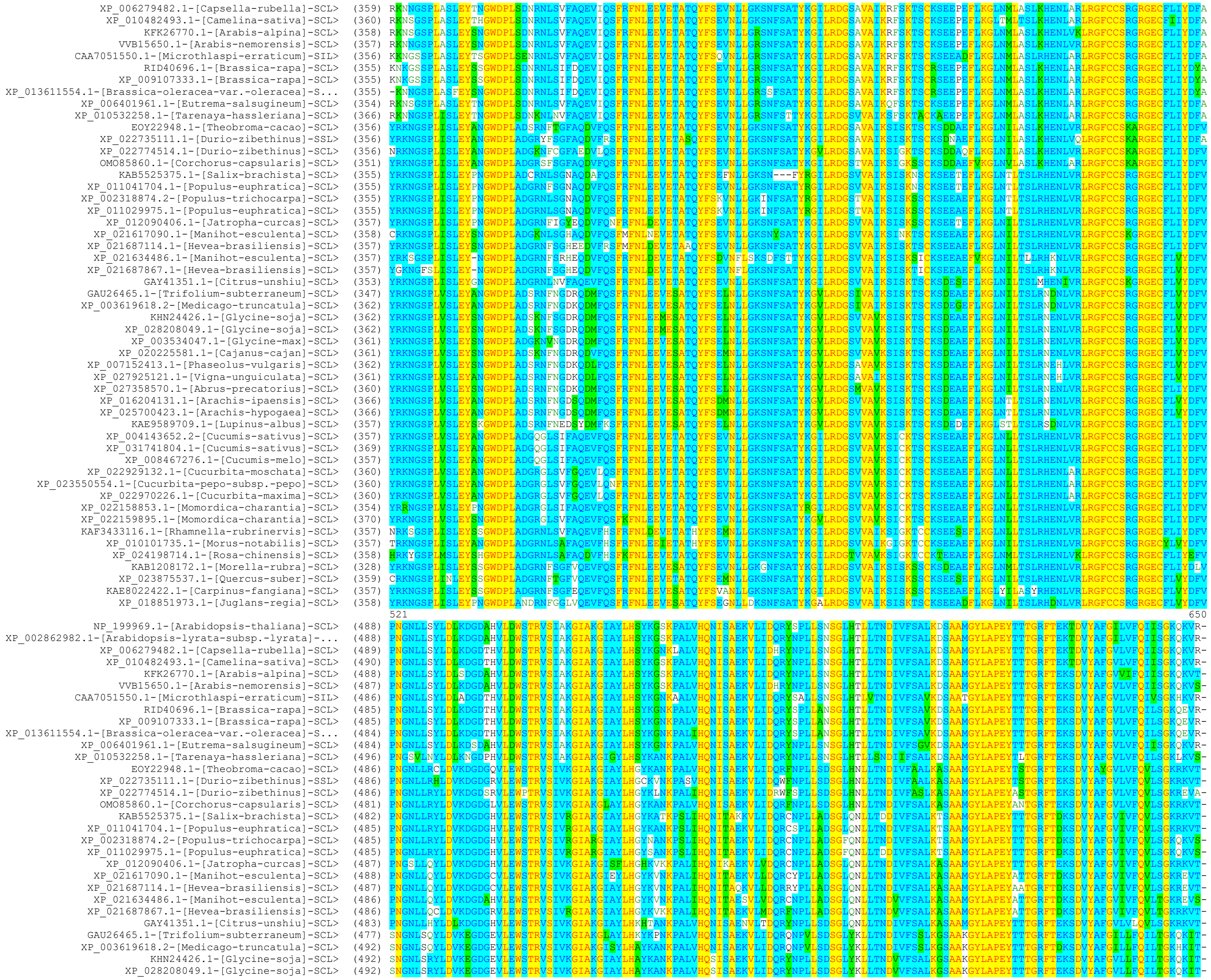

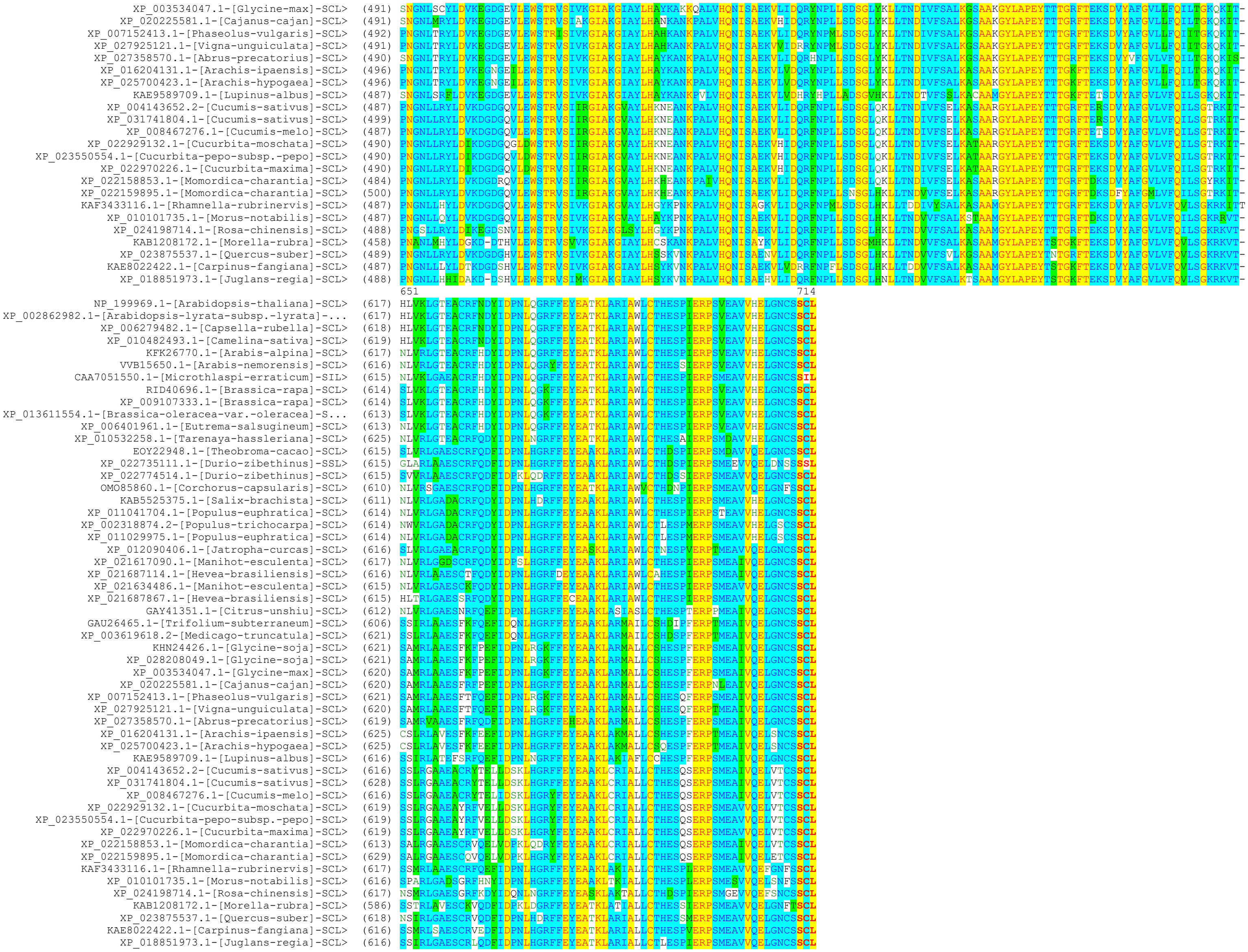

PTD11_ AT1G34420.1 (Verified in this study as peroxisomal)

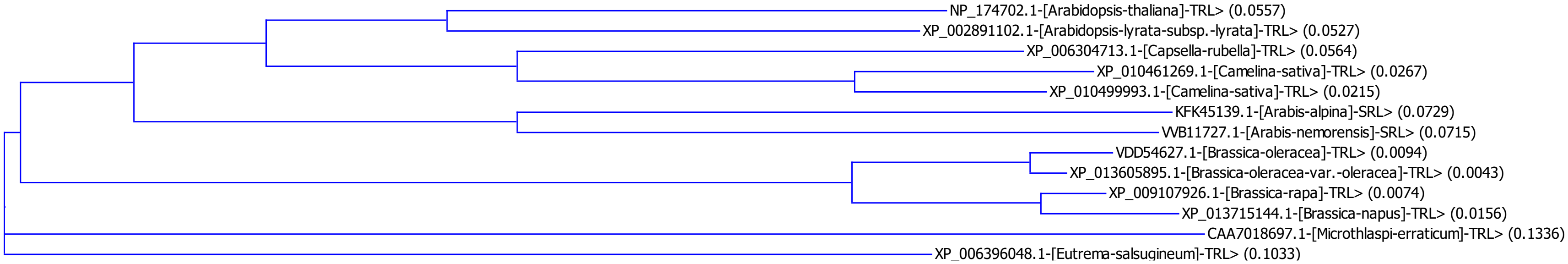


```
                                                         1                                                                                                          110
              NP_174702.1-[Arabidopsis-thaliana]-TRL>    (1) MFTGVKKMKKIHVPPETGKFMFCLFFSFL---SCCHVCFSELSLNQTNTMIELSSFLNISDWNLPGSERNPCSWNGVLCSLPDNSSVISLSLSNFDLSNSSFLPLVCNLQ
XP_002891102.1-[Arabidopsis-lyrata-subsp.-lyrata]-...    (1) -------MKKIHVPPETGKFMFCLFFTFF---SCCHVCFSELSLNQTNTMIELSRFLNISDWNLPGSEGNPCSWNGVLCSRPGNSSVISLSLSNFDLSNSSFLPLVCNLQ
           XP_006304713.1-[Capsella-rubella]-TRL>    (1) -----MKRKTIHVSQETGKFMFCLFFIFLP--CCCHVCFSELSLDQTNTMIELSRFLNISDWNLPGSEMNPCSWNGVLCSGQDNGSVISISVSGFDISNSSFLPVVCKLK
            XP_010461269.1-[Camelina-sativa]-TRL>    (1) ------MMKKIHVPPETGKFMFRLSFIFL---SCCHVCFSELSPDQTNTMIELSSFLNIPDWNRPGSEMNPCSWNGVLCSRPDNGSVISLSVSGFDLSNSSFLPVVCNLQ
            XP_010499993.1-[Camelina-sativa]-TRL>    (1) ------MMKKIHVPPETGKFMFCLFFIFL---SCCHVCFSELSPDQTNTMIELSRFLNIPDWNRPGSEMNPCSWNGIFCSRPDNGSVISLSVSGFDLSNSSFLSVVCNLH
                KFK45139.1-[Arabis-alpina]-SRL>    (1) --------------------------------------------------------------------------------------------------------------
             VVB11727.1-[Arabis-nemorensis]-SRL>    (1) -----------MKEKVISKSMLCXXXXXXXXXXXHVCFSELSSNQTNTMIELSSLLNISVWNLPGSERNPCLWKGVLCSRPDNGSVISLSLSGFSLSNSSFLPLICQIQ
              VDD54627.1-[Brassica-oleracea]-TRL>    (1) -------MKKIQVPPEPGKSMFCLFFFIFLS--CFHVCFPELSSTQTNTMIELYRFLSIPDWNLPGSERNPCSWEGVDCSRPDSSSVISLSLSGFGLSNSSFLPLVCQIQ
XP_013605895.1-[Brassica-oleracea-var.-oleracea]-T...    (1) -------MKKIQVPPEPGKSMFCLFFFIFLS--CFHVCFPELSSTQTNTMIELSRFLSIPDWNFPGSERNPCSWEGVDCSRPDSSSVISLSLSGFDLSNSSFLPLVCQIQ
              XP_009107926.1-[Brassica-rapa]-TRL>    (1) -------MKKIQVPPETGKSMFCLFFFSIFLS-CFHVCFPELSSTQTNTMIELSRFLNIPDWNLPGSERNPCSWEGVDCSRPDSSSVISLSLSGFDLSNSSFLPLVCQIQ
             XP_013715144.1-[Brassica-napus]-TRL>    (1) -------MKKIQVPPETGKSMFCLFFFSIFLS-CFHVCFPDLSSTQTNTMIELSRFLNIPDWNLPGSERNPCSWEGVDCSRPDSSSVISLSLSGFDLSNSSFLPLVCQIQ
      CAA7018697.1-[Microthlaspi-erraticum]-TRL>    (1) ------MKKIHDGPSETGKSIMFCFFFFFLS--CFHVCFSELSTNQTSTMIELSRILNISDWNLPGSERNPCLWRGVLCSQPDNGSVISLTLSGFDLTNSSFLPLLCQIQ
        XP_006396048.1-[Eutrema-salsugineum]-TRL>    (1) -------MKEIHVPSETGKSMFFLFFIIFLS--CCNLSFSELSSNQTSTMIELSRFLNVSDWNLPGSERNPCLWKGVLCSRPDNGSVISLSLSRFDLTNSSFLPLVCQIQ
                                                         111                                                                                                        220
              NP_174702.1-[Arabidopsis-thaliana]-TRL>  (108) TLESLDVSNNRLSSIPEGFVTN-CERLIALKHLNFSTNKFSTS---------PGFRGFSKLAVLDFSHNVLSGNVGDYGFDGLVQLRSLNLSFNRLTGSVPVHLTKSLEK
XP_002891102.1-[Arabidopsis-lyrata-subsp.-lyrata]-...  (101) TLESLDVSNNRLSSIPDGFVTN-CEKLIGLKHLNFSTNKFSSS---------PGFRGFSKLAVLDFSHNVLSGNIGDYGFDGLVQLRSLNLSFNSLTGSVPVHLTKGLEK
           XP_006304713.1-[Capsella-rubella]-TRL>  (104) TLESLDVSNNSLSLIPDGFVTT-CERLLGLKHLNLSSNKFSSS---------PSFRGFSKLVVLDFSYNSLSGKIPDYGFDGLVQLRSLNLSFNSLTGSVPVNLSKRLEK
            XP_010461269.1-[Camelina-sativa]-TRL>  (102) TLESLDVSNNSLRLIPDGFVTN-CERLLGFKHLNLSSNNFSSS---------PSFRGLSKLKVLDFSYNVLSGSIRDYGFDGMVQLRSLNLSFNSLTGSVPINLTKSLEK
            XP_010499993.1-[Camelina-sativa]-TRL>  (102) TLESLDVSNNSLSLIPDGFVTN-CERLLGFKHLNLSSNKFSSS---------PSFRGFSKLAVLDFSHNVLSGNIRDYGFDGLVQLRSLNLSFNSLTGSVPINLTKSLER
                KFK45139.1-[Arabis-alpina]-SRL>    (1) --------------------------------------------------------------------------------------------------------------
             VVB11727.1-[Arabis-nemorensis]-SRL>  (100) TLESLDVSDNILSSIPDEFVTS-CKRLVGLKHLNFSNNKVSSP---------PVFSGFLKLEVLDFSHNALSGNTRDYGFDGLVQLRSLNLSFNSFTGSVPIDLTKSLEK
              VDD54627.1-[Brassica-oleracea]-TRL>  (102) TLESLDVSNNSLSSIPEGFMTN-CGTLVGLKQLNFSGNQVSSF---------RGFRNFSKLEVLDISHNRLSGNIEDYGFDALVQLKNLSLSFNKLAGSVPTNLAKSVVN
XP_013605895.1-[Brassica-oleracea-var.-oleracea]-T...  (102) TLESLDVSNNSLSSIPDGFMTN-CRTLVGLKQLNFSGNQVSSF---------RGFRNFSKLEVLDISHNRLSGNIEDYGFDALVQLKNLSLSFNKLAGSVPTNLAKSVVN
              XP_009107926.1-[Brassica-rapa]-TRL>  (103) TLESLDVSNNSLSSIPDGFMTN-CGTLVGLKQLNFSGNQVSSF---------PGFRNFSKLEVLDISHNRLSGNIGDYGFDALVQLKNLSLNFNKLAGSVPTDLAKSLVN
             XP_013715144.1-[Brassica-napus]-TRL>  (103) TLESLDVSNNSLSSIPDGFMTN-CGTLVGLKQLNFSGNQVSSF---------PGFRNFSKLEVLDISHNRLSGNIGDYGFDALVQLKNLSLNFNKLAGSVPTDLAKSLVN
      CAA7018697.1-[Microthlaspi-erraticum]-TRL>  (103) TLESLDVSNNSLSSIPDGFMTTNCETLVGLKHLNFSMNQLSSSSSSSSSSSPGLRYFSKLEDLDFSYNLLSGNIGDYGLDGLVQLRTLNLNFNNLTGSVPSNLSKTLDK
        XP_006396048.1-[Eutrema-salsugineum]-TRL>  (102) TLESLDVSSNSLSSVPDGFMTN-CETLVGLKQLNFSMNQVSSF---------PRFRNFSKLEVLDFSYNRLSGNIGDYGFDGLVQLKSLNLNFNNLSGSVPTNLAKRVET
                                                         221                                                                                                        330
              NP_174702.1-[Arabidopsis-thaliana]-TRL>  (208) LEVSDNSLSGTIPEGIKDYQELTLIDLSDNQLNGSIPSSLGNLSKLESLLLSNNYLSGLIPESLSSIQTLRRFAANRNRFTGEIPSGLTKHLENLDLSFNSLAGSIPGDL
XP_002891102.1-[Arabidopsis-lyrata-subsp.-lyrata]-...  (201) LEVSDNGLSGTIPEGIDDYQELKLIDLSDNQLSGSIPSSLGNLSKLESLLLSNNYLSGLIPESLSSIQTLRRFAANRNQFTGRIPSGITKHLENLDLSFNKLAESIPDDL
           XP_006304713.1-[Capsella-rubella]-TRL>  (204) LEVSDNFLTGTIPEGIEDYQELTLIDLSDNQLVGSIPSSLGNLTKLESLILSNNKLSGSIPESLSRIQTLRRFAANRNHFTGVIPSGITKNLANLDLSFNNLNGSIPGDL
            XP_010461269.1-[Camelina-sativa]-TRL>  (202) LEVSDNHLSGTIPEGIGHYPELTLIDLSDNHLIGSIPSSLGNLSTLESLILSNNNFGGPVPESLSSIQTLRRFAANRNKFTGVIPSGITKNLANLDLSFNHLNGSIPGDL
            XP_010499993.1-[Camelina-sativa]-TRL>  (202) LEVSDNRLSGTIPEGIGHYQEFRLIDLSDNHLIGSIPSSLGNLSKLESLILSNNNLSGPVPESLSRIQTLRRFAANRNKFTGVIPSGITKNLANLDLSFNHINGSIPGDL
                KFK45139.1-[Arabis-alpina]-SRL>    (1) -----------------------MIDLSDNQLNGSIPSSLRNLSKLESLLLSNNHLSGPIPESLSSIQTLRRFAAHQNRFTGALPSGITKHLENLDLSFNNLNGSLPDDL
             VVB11727.1-[Arabis-nemorensis]-SRL>  (200) LEVSNNNLSGTIPESIQDYQELKLIDLSDNQLNGSIPSSLSNLTKLESLLLSNNQLSGPIPESLSSIQTLRRFAANQNRFTGAIPSGITKHLENLDLSFNQLKGSLPDDL
              VDD54627.1-[Brassica-oleracea]-TRL>  (202) LEVSDNLLSGSIPEGIDDYQELRLIDLSDNQLDGSLPSSLGNLSKLETLLLSNNNLKGAIPESLSRIQTLSRFAANRNGFTGAIPSGITKHLENLDLSFNLLNGSIPDDL
XP_013605895.1-[Brassica-oleracea-var.-oleracea]-T...  (202) LEVSDNLLSGSIPEGIDDYQELRLVDLSDNQLDGSLPSSLGNLSKLETLLLSNNNLKGAIPESLSRIQTLSRFAANRNGFTGAIPSGITKHLENLDLSFNLLNGSIPDDL
              XP_009107926.1-[Brassica-rapa]-TRL>  (203) LEVSDNLLSGSIPEGIEEYQELRLIDLSDNQLNGSLPSSLGNLSKLETLLLSNNNLKGAIPESLSRIQTLSRFAANRNGFTGAIPSGITKHVENLDLSFNLVNGSIPDDL
             XP_013715144.1-[Brassica-napus]-TRL>  (203) LEVSDNLLSGSIPEGIEEYQELRLIDLSDNLLNGSLPSSLGNLSKLETLLLSNNNLKGAIPESLSRIQTLSRFAANRNGFTGAIPSGITKHVENLDLSFNLVNGSIPDDL
      CAA7018697.1-[Microthlaspi-erraticum]-TRL>  (213) LEITNNSLSGTVPESIQDYQELKLLDLSDNQLNGSIPSSLGNLSKLETLLLSNNHLLGPIPESLSRIQTLQRFAANRNRFTGEIPSAITRHVKNLDLSYNNLKGTIPVDL
        XP_006396048.1-[Eutrema-salsugineum]-TRL>  (202) LEVSYNSLSGSIPEGIEDYQELKLIDLSNNQLNGSIPSSLGKLSKLESLLLSNNHLSGSIPESLPNIQTLRRFAANRNRFTGEIPSKITRHLVSLDLSYNSLTGLIPSEL
                                                         331                                                                                                        440
              NP_174702.1-[Arabidopsis-thaliana]-TRL>  (318) LSQ-LKLVSVDLSSNQLVGWIP-QSISSSLVRLRLGSNKLTGSVPSVAFESLQLLTYLEMDNNSLTGFIPPSFGNLVSLNLLNLAMNEFTGILPPAFGNL-SRLQVIKLQ
XP_002891102.1-[Arabidopsis-lyrata-subsp.-lyrata]-...  (311) LSQ-LKLVSVDLSFNHLVGWIP-QSISPSLVRLRLGSNKLTGKVPSTAFESLQNLTYLEMDNNSLTGNIPPSFGNLVILNLLNLAMNEFTGNLPPAFGNL-TRLQVIKLQ
           XP_006304713.1-[Capsella-rubella]-TRL>  (314) LSQ-LKLVSVDLSSNQLVGWIP-RSISPGLFRLRLGSNMLRGPVPSAAFESLQNLTYLEMENNRLTGHIPHAFGNLQSLNLLNLAMNEFTGILPLSFGNL-TRLQVIKLQ
```

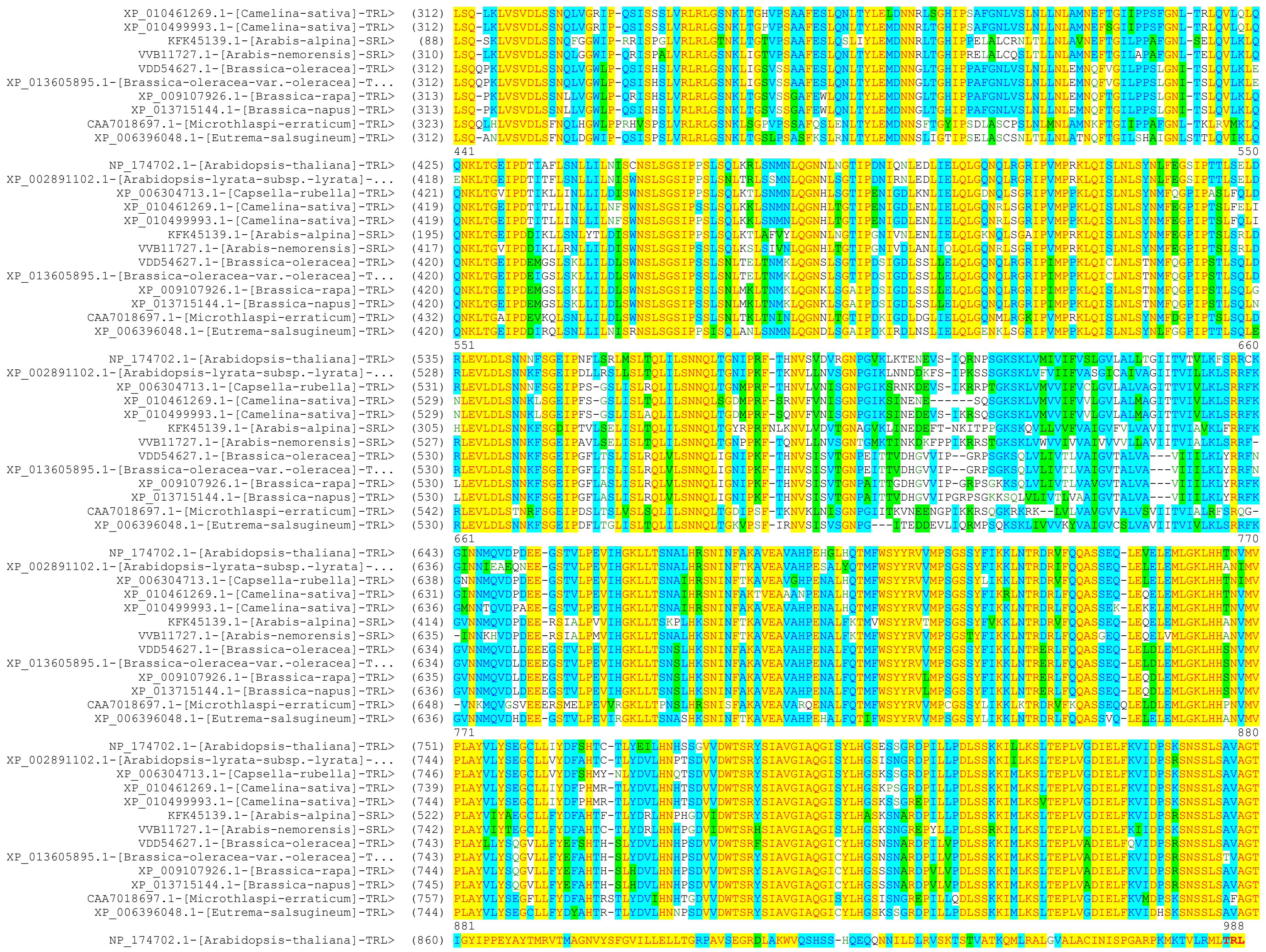

```
XP_002891102.1-[Arabidopsis-lyrata-subsp.-lyrata]-...  (853) IGYIPPEYAYTMRVTMAGNVYSFGVILLELLTGRPAVSEGRDLAKWVQSHSS-HQEQQNNILDLRVSKTSTVATKQMLRALSVALACINISPGARPKMKTVLRMLTRL
             XP_006304713.1-[Capsella-rubella]-TRL>  (855) IGYIPPEYAYTMRVTMAGNVYSFGVILLELLTGRPAVSEGRELAKWVQSHSS-QQEQCNNILDVRVSKTSSVATKQMLRALSVALACINISPGARPKMKTVLRMLTRL
              XP_010461269.1-[Camelina-sativa]-TRL>  (848) IGYIPPEYAYTMRVTMAGNVYSFGVILLELLTGRQAVSEGRELAKWVQSHSS-QQEQCNNILDLRVSKTSSVATRQMLRALSVALACINISPGARPKMKTVLKMLTRL
              XP_010499993.1-[Camelina-sativa]-TRL>  (853) IGYIPPEYAYTMRVTMAGNVYSFGVILLELLTGRQAVSEGRELAKWVQSHSS-QQEQCNNILDLRVSKTSSVATRQMLRALSVALACINISPGARPKMKTVLKMLTRL
                  KFK45139.1-[Arabis-alpina]-SRL>  (631) VGYIPPEYAYTMRVTTAGNVYSFGVILLELLTGKKAVSEGRELAKWVQSHSR-QQEQLNNILDLRVSKTSTVATKQMLRALSVALACINISPGARPNMRNVQRMLSRL
             VVB11727.1-[Arabis-nemorensis]-SRL>  (851) IGYIPPEYAYTMRVTMAGNVYSFGVILLELLTGRPAVSEGRDLAKWVQRHSS-QQEQHNNILDLRVSKTSTVATKQMLRALSVALACINISPGARPSMRNVLRMLSRL
             VDD54627.1-[Brassica-oleracea]-TRL>  (852) IGYIPPEYAYTMRVTMAGNVYSFGVILLELLTGKPAVSEGRELSKWVQS----QQEQRNNILDLRLSKSSPVATKQMIRALSIALACINISPGARPKMKTVLRMLTRL
XP_013605895.1-[Brassica-oleracea-var.-oleracea]-T...  (852) IGYIPPEYAYTMRVTMAGNVYSFGVILLELLTGKPAVSEGRELSKWVQS----QQEQRNNILDLRVSKSSPVATKQMIRALSIALACINISPGARPKMKTVLRMLTRL
              XP_009107926.1-[Brassica-rapa]-TRL>  (853) IGYIPPEYAYTMRVTMAGNVYSFGVILLELLTGKPAVSEGRELSKWVQS----QQEQRNNILDLRVSKSSPVATKQMIRALSIALACINISPGARPKMKTVLRMLTRL
             XP_013715144.1-[Brassica-napus]-TRL>  (854) IGYIPPEYAYTMRVTMAGNVYSFGVILLELLTGKPAVSEGRELSKWVQS----QQEQRNNILDLRVSKSSPVATKQMIRALSIALACINISPGARPKMKTVLRMLTRL
     CAA7018697.1-[Microthlaspi-erraticum]-TRL>  (867) IGYIPPEYAYTMRVTTAGNVYSFGVILLELLTGKPAVSEGRELAKWVMQRHSSQQDECNNILDLRVSKTSTTATKQMLRALSIALACINISPGARPKMKTVLRMLTRL
      XP_006396048.1-[Eutrema-salsugineum]-TRL>  (853) IGYIPPEYAYTMRVTMAGNVYSFGVILLELLTGRPAVCEGRELAKWVQSHPS-QPEQLNSILDIRVSKTSTVATKQMLRALSVALACINISPGARPKMKNVLRMLTRL
```

PTD15_AT3G24790.1 (Appeared in this study in Cytosol)

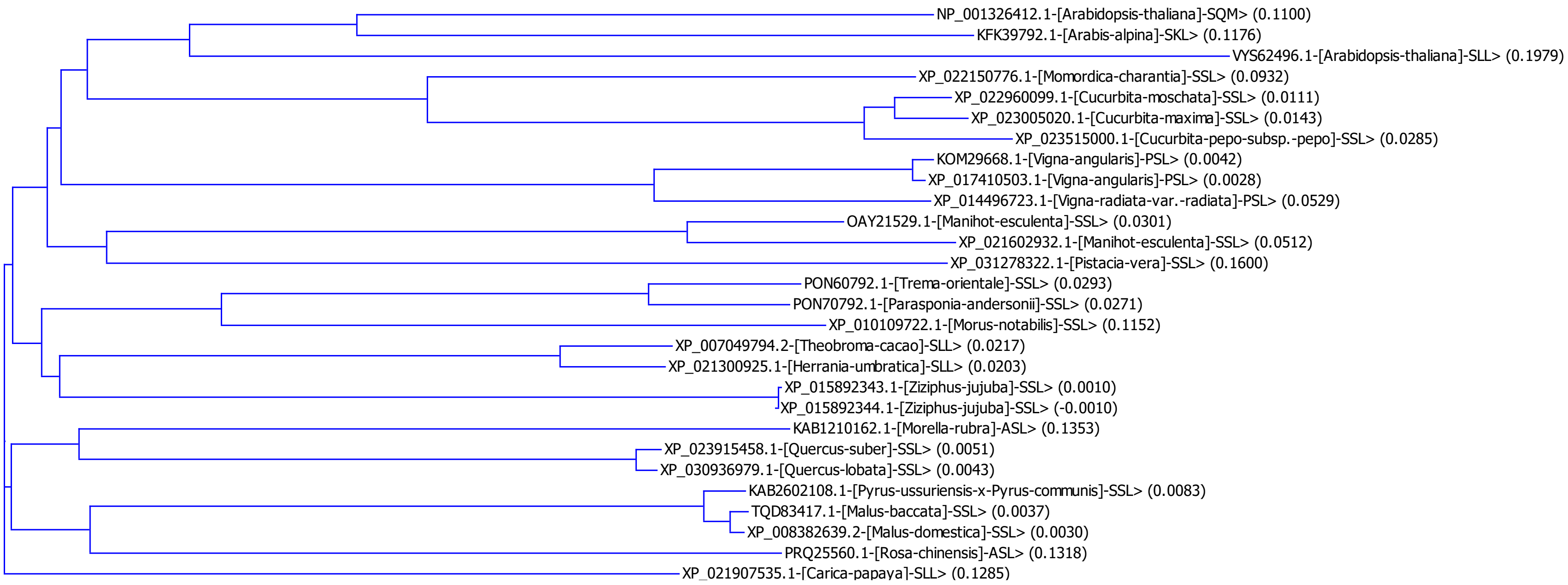


```
                                                        1                                                                                                    100
NP_001326412.1-[Arabidopsis-thaliana]-SQM>          (1) ------------------------------MSCFSCFSSKVLDNEGSSMPAPYKQPNSPKRTTGEVVAKNA-------N---------------------
KFK39792.1-[Arabis-alpina]-SKL>                     (1) ------------------------------MSCFSCFSSTNTDNEGSSMPAPYKQPSSPKRTTGQVVAN-------------------------------
VYS62496.1-[Arabidopsis-thaliana]-SLL>              (1) ------------------------------MSCFLGPSTNNKSRENEGSSMAAPYEQQNLPRNDRRQITT-----------------WEAVGTNKESP--
XP_022150776.1-[Momordica-charantia]-SSL>           (1) ------------------------------MSCFPCFSSHGKAAKRTKSGRKEXSVSLHSRSLKQHQQQQPAAP---PPRPTPTENQKGNQQQNDAERERE
XP_022960099.1-[Cucurbita-moschata]-SSL>            (1) ------------------------------MGCFSCFSSHGKAPKRRKNCRRKQSGSLHSRSLKPQQQHHP---------SPPSENTKGNQVQNEAEKENE
XP_023005020.1-[Cucurbita-maxima]-SSL>              (1) ------------------------------MGCFSCFSSHGKAPKKRKNCRRKQSVSLHSGSLKPQQQHHYPAP-------PPSENPKGNQVQNEAEKENE
XP_023515000.1-[Cucurbita-pepo-subsp.-pepo]-SSL>    (1) ------------------------------MGCFSCFSSHGKAPKRRKNCRRKQSVSLHSRSLKPQQQHHHPAP---PSGHICTDNTKGNQVQNEAEKENE
KOM29668.1-[Vigna-angularis]-PSL>                   (1) ------------------------------MSCFSCFSSSKAAKPSNNGKRMPQPTCHSPKESAQSLPVTEN------HNYKAKTNHQTEPKIEEVKKDK-
XP_017410503.1-[Vigna-angularis]-PSL>               (1) ------------------------------MSCFSCFSSSKAAKPSNNGKRMPQPTCHSPKESAQSLPENHN--------YKAKTNHQTEPKIEEVKKDK-
XP_014496723.1-[Vigna-radiata-var.-radiata]-PSL>    (1) ------------------------------MSCFSCFSSTKAAKPCNNGKRMQQPASHSPKESAQSQPEN------YNNKAKTNHHQSEPKMDKEVNKDN-
OAY21529.1-[Manihot-esculenta]-SSL>                 (1) ------------------------------MSCFSCLSSQDKKAARSSGSSRRSRSLPPASTPRKKESSNA-------ENPKP---KPQADQPHNNSKE--
XP_021602932.1-[Manihot-esculenta]-SSL>             (1) MVLNSSVIVLVSRFLVSLVFLDQAVPKTSSSSSFFFLLSDVIERAWHSFDAFLNQKLFLGFAHLIEFSFHE-------N-PKP---KPQADQPHNNSKE--
XP_031278322.1-[Pistacia-vera]-SSL>                 (1) ------------------------------MSCFSCFSSHGRKASSKKIHSSKKLRVQISTAPRDQNPPLPQNFS-----PKPRPAQDPVSNKDAANKEAG
PON60792.1-[Trema-orientale]-SSL>                   (1) ------------------------------MSCFSCFQPSKKKSAKRSISLIRRPGQTPVAVPRKEPNIVPQPR--PSENPKPKSPPKIKTTTTNSNTNNN
PON70792.1-[Parasponia-andersonii]-SSL>             (1) ------------------------------MSCFSCFQPSKKKSAKRSISLIRRPGQTPVAIPRKEPNIVPQPR--PSENPKPKSPPKIETTTTNSNNNNN
XP_010109722.1-[Morus-notabilis]-SSL>               (1) ------------------------------MSCFSCFQSSNQKRSVKRSRSGRRSGQTLGSVLRKEANIAP---------------QHHHNHHHHHYPATN
XP_007049794.2-[Theobroma-cacao]-SLL>               (1) ------------------------------MSCFSCFSSQDKKARRLNNPSRGSQTSYYSAPPRQALLPRQRPV----NNPKPKTSAPEPTKDTDAPKA--
XP_021300925.1-[Herrania-umbratica]-SLL>            (1) ------------------------------MSCFSCFSSQDKKARRLNNPSRRTQTSYYAAPPREALPPQQSLE---NNPKPKTSAPEPTKNTDAPKA--
XP_015892343.1-[Ziziphus-jujuba]-SSL>               (1) ------------------------------MSCFSCFSSHEKKAPRRANSARRAQLPPAVHKEPVSQPHRP-------------VTEIPRTKTPAPAEAAN
XP_015892344.1-[Ziziphus-jujuba]-SSL>               (1) ------------------------------MSCFSCFSSHEKKAPRRANSARRAQLPPAVHKEPVSQPHRP---------------EIPRTKTPAPAEAAN
KAB1210162.1-[Morella-rubra]-ASL>                   (1) ------------------------------MSCFSCFSSHEKKASKKTKGGGRQELPPVPPRREVASAQQP-------R---PENSKQKVAPVETPEKK--
XP_023915458.1-[Quercus-suber]-SSL>                 (1) ------------------------------MSCFSCFSSHGNKAKGTNSGRRRELAAAPKELPISSQPRPE-------N-PKPK-VIPVETTNKDAIKE--
XP_030936979.1-[Quercus-lobata]-SSL>                (1) ------------------------------MSCFSCFSSHRNKAKGTNSGRRRELAAAPKELPISSQPRPE-------N-PKPK-VIPVETTNKDAIKE--
```

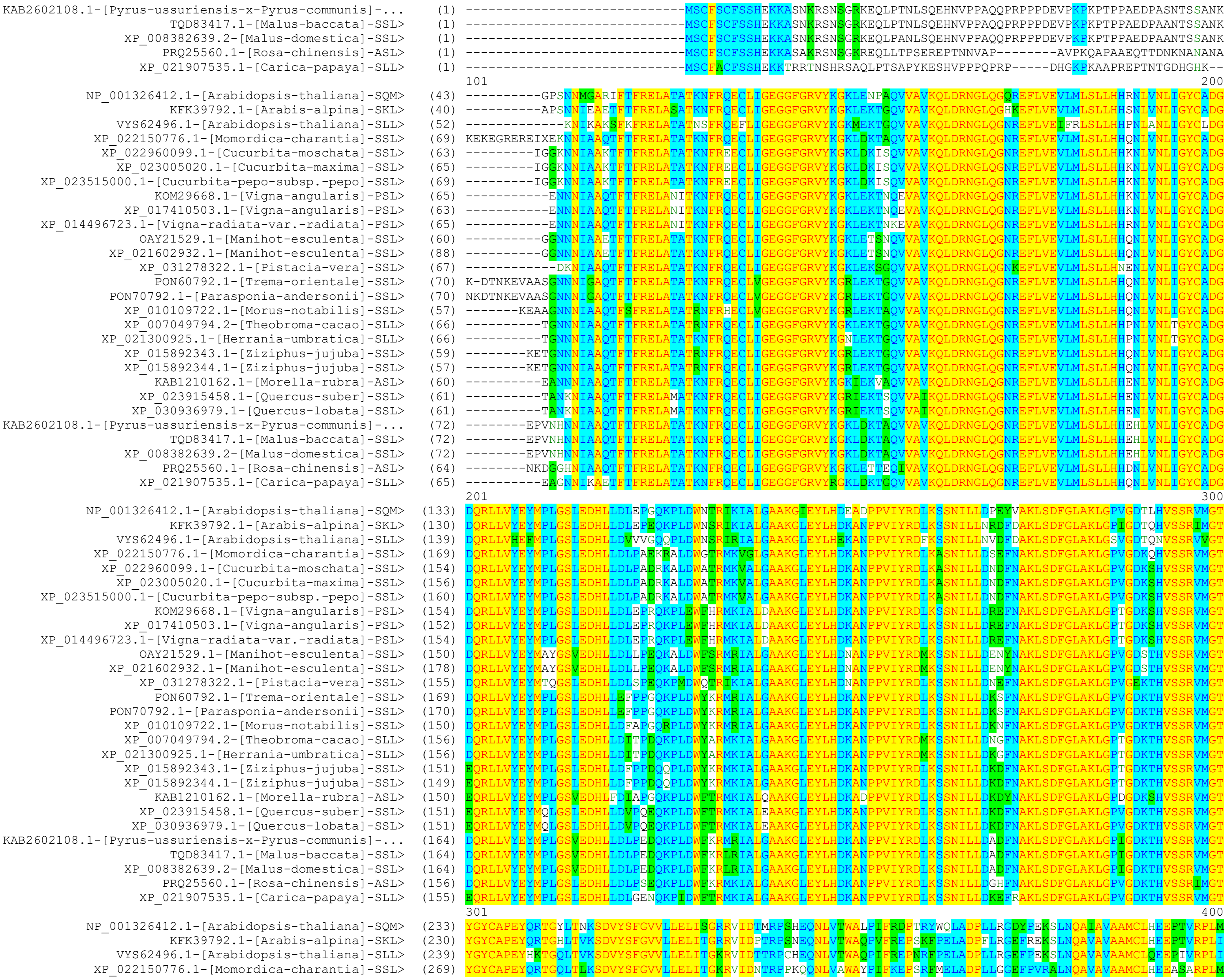

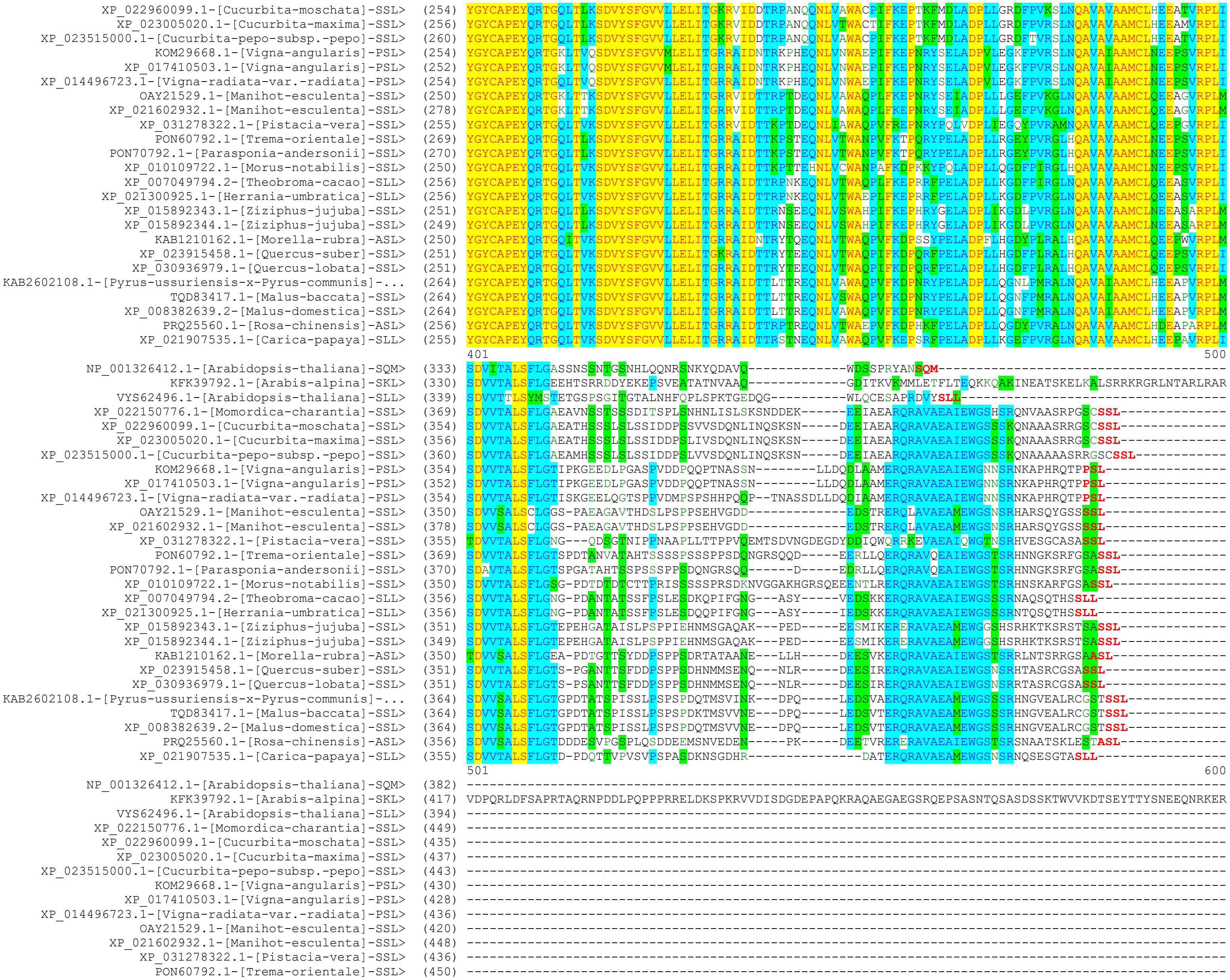

```
                 PON70792.1-[Parasponia-andersonii]-SSL>  (445) ----------------------------------------------------------------------------------------------------
                   XP_010109722.1-[Morus-notabilis]-SSL>  (434) ----------------------------------------------------------------------------------------------------
                   XP_007049794.2-[Theobroma-cacao]-SLL>  (430) ----------------------------------------------------------------------------------------------------
                XP_021300925.1-[Herrania-umbratica]-SLL>  (430) ----------------------------------------------------------------------------------------------------
                   XP_015892343.1-[Ziziphus-jujuba]-SSL>  (429) ----------------------------------------------------------------------------------------------------
                   XP_015892344.1-[Ziziphus-jujuba]-SSL>  (427) ----------------------------------------------------------------------------------------------------
                       KAB1210162.1-[Morella-rubra]-ASL>  (426) ----------------------------------------------------------------------------------------------------
                     XP_023915458.1-[Quercus-suber]-SSL>  (426) ----------------------------------------------------------------------------------------------------
                    XP_030936979.1-[Quercus-lobata]-SSL>  (426) ----------------------------------------------------------------------------------------------------
KAB2602108.1-[Pyrus-ussuriensis-x-Pyrus-communis]-...  (443) ----------------------------------------------------------------------------------------------------
                         TQD83417.1-[Malus-baccata]-SSL>  (443) ----------------------------------------------------------------------------------------------------
                   XP_008382639.2-[Malus-domestica]-SSL>  (443) ----------------------------------------------------------------------------------------------------
                        PRQ25560.1-[Rosa-chinensis]-ASL>  (432) ----------------------------------------------------------------------------------------------------
                     XP_021907535.1-[Carica-papaya]-SLL>  (422) ----------------------------------------------------------------------------------------------------
                                                                601                                                                                     695
              NP_001326412.1-[Arabidopsis-thaliana]-SQM>  (382) -----------------------------------------------------------------------------------------------
                        KFK39792.1-[Arabis-alpina]-SKL>  (517) ERRALKRSYTTQLAEMQAQMERVASDIKRTQSQIHHVTGRAPAIAQVLEKVRNMPFNERIARTKISGLGEHALRKNYRPFGPSDRIRSGLGESKL
                   VYS62496.1-[Arabidopsis-thaliana]-SLL>  (394) -----------------------------------------------------------------------------------------------
                XP_022150776.1-[Momordica-charantia]-SSL>  (449) -----------------------------------------------------------------------------------------------
                 XP_022960099.1-[Cucurbita-moschata]-SSL>  (435) -----------------------------------------------------------------------------------------------
                   XP_023005020.1-[Cucurbita-maxima]-SSL>  (437) -----------------------------------------------------------------------------------------------
          XP_023515000.1-[Cucurbita-pepo-subsp.-pepo]-SSL>  (443) -----------------------------------------------------------------------------------------------
                       KOM29668.1-[Vigna-angularis]-PSL>  (430) -----------------------------------------------------------------------------------------------
                   XP_017410503.1-[Vigna-angularis]-PSL>  (428) -----------------------------------------------------------------------------------------------
          XP_014496723.1-[Vigna-radiata-var.-radiata]-PSL>  (436) -----------------------------------------------------------------------------------------------
                     OAY21529.1-[Manihot-esculenta]-SSL>  (420) -----------------------------------------------------------------------------------------------
                 XP_021602932.1-[Manihot-esculenta]-SSL>  (448) -----------------------------------------------------------------------------------------------
                     XP_031278322.1-[Pistacia-vera]-SSL>  (436) -----------------------------------------------------------------------------------------------
                       PON60792.1-[Trema-orientale]-SSL>  (450) -----------------------------------------------------------------------------------------------
                 PON70792.1-[Parasponia-andersonii]-SSL>  (445) -----------------------------------------------------------------------------------------------
                   XP_010109722.1-[Morus-notabilis]-SSL>  (434) -----------------------------------------------------------------------------------------------
                   XP_007049794.2-[Theobroma-cacao]-SLL>  (430) -----------------------------------------------------------------------------------------------
                XP_021300925.1-[Herrania-umbratica]-SLL>  (430) -----------------------------------------------------------------------------------------------
                   XP_015892343.1-[Ziziphus-jujuba]-SSL>  (429) -----------------------------------------------------------------------------------------------
                   XP_015892344.1-[Ziziphus-jujuba]-SSL>  (427) -----------------------------------------------------------------------------------------------
                       KAB1210162.1-[Morella-rubra]-ASL>  (426) -----------------------------------------------------------------------------------------------
                     XP_023915458.1-[Quercus-suber]-SSL>  (426) -----------------------------------------------------------------------------------------------
                    XP_030936979.1-[Quercus-lobata]-SSL>  (426) -----------------------------------------------------------------------------------------------
KAB2602108.1-[Pyrus-ussuriensis-x-Pyrus-communis]-...  (443) -----------------------------------------------------------------------------------------------
                         TQD83417.1-[Malus-baccata]-SSL>  (443) -----------------------------------------------------------------------------------------------
                   XP_008382639.2-[Malus-domestica]-SSL>  (443) -----------------------------------------------------------------------------------------------
                        PRQ25560.1-[Rosa-chinensis]-ASL>  (432) -----------------------------------------------------------------------------------------------
                     XP_021907535.1-[Carica-papaya]-SLL>  (422) -----------------------------------------------------------------------------------------------
```

PTD23_ AT1G20930.1 (Appeared in this study in Cytosol)

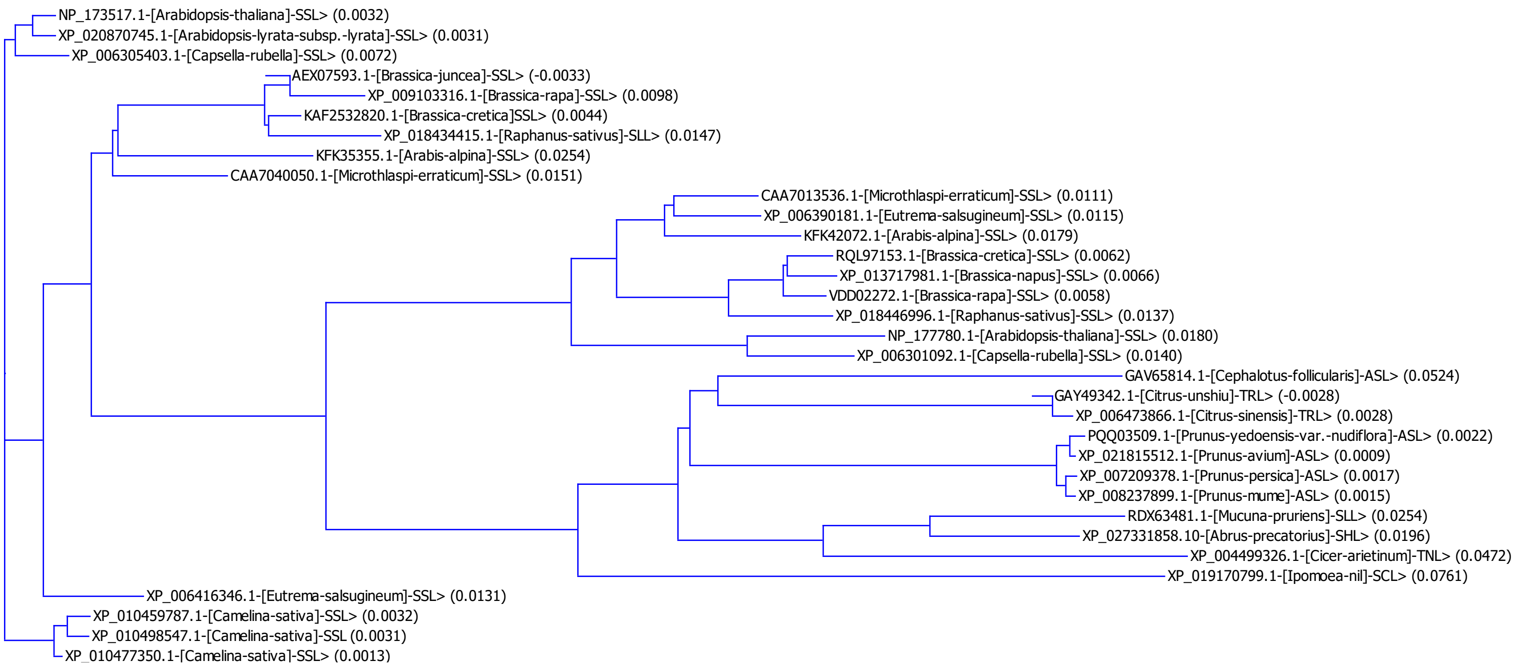


```
                                                               1                                                                                                        110
                    NP_173517.1-[Arabidopsis-thaliana]-SSL> (1) MDNN------GVKPAVSAMEAFEKLEKVGEGTYGKVYRAREKATGMIVALKKTRLHEDEEGVPPTTLREISILRMLARDPHIVRLMDVKQGINKEGKTVLYLVFEYVDTD
XP_020870745.1-[Arabidopsis-lyrata-subsp.-lyrata]-... (1) MDNN------GVKPAVSAMEAFEKLEKVGEGTYGKVYRAREKATGMIVALKKTRLHEDEEGVPPTTLREISILRMLARDPHIVRLMDVKQGINKEGKTVLYLVFEYVDTD
                  XP_006305403.1-[Capsella-rubella]-SSL> (1) MDNN------GVKPSVSAMEAFEKLEKVGEGTYGKVYRAREKATGMIVALKKTRLHEDEEGVPPTTLREISILRMLARDPHIVRLMDVKQGINKEGKTVLYLVFEYVDTD
                       AEX07593.1-[Brassica-juncea]-SSL> (1) --------------AVSAMEAFEKLEKVGEGTYGKVYRAREKATGLIVALKKTRLHEDEEGVPPTTLREISILRMLARDPHIVMLMDVKQGLNKEGKTVLYLVFEYVDTD
                     XP_009103316.1-[Brassica-rapa]-SSL> (1) MENNG-----VKSAASSAMEAFEKLEKVGEGTYGKVYRAREKATGLIVALKKTRLHEDEEGVPPTTLREISILRMLARDPHIVRLMDVKQGLNKEGKTVLYLVFEYVDTD
                      KAF2532820.1-[Brassica-cretica]SSL> (1) MENN------GVKSAASAMEAFEKLEKVGEGTYGKVYRAREKATGLIVALKKTRLHEDEEGVPPTTLREISILRMLARDPHIVRLMDVKQGLNKEGKTVLYLVFEYVDTD
                  XP_018434415.1-[Raphanus-sativus]-SLL> (1) MENN-------TKSGGSAMEAFEKLEKVGEGTYGKVYRAREKATGLIVALKKTRLHEDEEGVPPTTLREISILRMLARDPHIVRLMDVKQGLNKEGKTVLYLVFEYVDTD
                         KFK35355.1-[Arabis-alpina]-SSL> (1) MENG-------VKSTVSAMEAFEKLEKVGEGTYGKVYRAREKATGLIVALKKTRLHEDEEGVPPTTLREISILRMLARDPHIVRLMDVKQGLNKEGKTVLYLVFEYVDTD
             CAA7040050.1-[Microthlaspi-erraticum]-SSL> (1) METG-------VKTAVSAMEAFEKLEKVGEGTYGKVYRAREKATGLIVALKKTRLHEDEEGVPPTTLREISILRMLARDPHIVRLMDVKQGLNKEGKTVLYLVFEYVDTD
             CAA7013536.1-[Microthlaspi-erraticum]-SSL> (1) MDKE-------EVIAVSAMDAFEKLEKVGEGTYGKVYRAREKATGKIVALKKTRLHEDEEGVPSTTLREISILRMLARDPHIVRLMDVKQGLSKEGKTVLYLVFEYMDTD
               XP_006390181.1-[Eutrema-salsugineum]-SSL> (1) --------MDKEVIAVSAMDAFEKLEKVGEGTYGKVYRAREKATGKIVALKKTRLHEDEEGVPSTTLREVSILRMLARDPHIVRLMDVKQGLSKDGKTVLYLVFEYMDTD
                         KFK42072.1-[Arabis-alpina]-SSL> (1) --------MDKEVIAVSAMDAFEKLEKVGEGTYGKVYRAREKATGKIVALKKTRLHEDEEGVPSTTLREISILRMLARDPHIVRLMDVKQGLSKEGKTVLYLVFEYMDTD
                     RQL97153.1-[Brassica-cretica]-SSL> (1) --------MDKEVIAVSAMDAFEKLEKVGEGTYGKVYRAREKATGKIVALKKTRLHEDEEGVPSTTLREISILRMLARDPHIVRLMDVKQGLSKDGKTVLYLVFEYMDTD
                   XP_013717981.1-[Brassica-napus]-SSL> (1) --------MDKEVIAVSAMDAFEKLEKVGEGTYGKVYRAREKATGKIVALKKTRLHEDEEGVPSTTLREISILRMLARDPHIVRLMDVKQGLSKDGKTVLYLVFEYMDTD
                        VDD02272.1-[Brassica-rapa]-SSL> (1) --------MDKEVIAVSAMDAFEKLEKVGEGTYGKVYRAREKATGKIVALKKTRLHEDEEGVPSTTLREISILRMLARDPHIVKLMDVKQGLSKDGKTVLYLVFEYMDTD
                  XP_018446996.1-[Raphanus-sativus]-SSL> (1) --------MDKEVIAVSAMEAFEKLEKVGEGTYGKVYRAREKSTGKIVALKKTRLHEDEEGVPSTTLREISILRMLARDPHIVKLMDVKQGLSKDGKTVLYLVFEYIDTD
                  NP_177780.1-[Arabidopsis-thaliana]-SSL> (1) --------MDEGVIAVSAMDAFEKLEKVGEGTYGKVYRAREKATGKIVALKKTRLHEDEEGVPSTTLREISILRMLARDPHVVRLMDVKQGLSKEGKTVLYLVFEYMDTD
                  XP_006301092.1-[Capsella-rubella]-SSL> (1) --------MDEGVIAVSAMDAFEKLEKVGEGTYGKVYRAREKATGTIVALKKTRLHEDEEGVPSTTLREISILRMLARDPHIVRLMDVKQGLSKEGKTVLYLVFEYMDTD
               GAV65814.1-[Cephalotus-follicularis]-ASL> (1) -----------MEKTVSAMDAFEKLEKVGEGTYGKVYRAREKATGKIVALKKTRLHEDDEGVPPTTLREVSILRMLSRDPHVVRLMDVKQGQNKEGKTVLYLVFEYMDTD
                        GAY49342.1-[Citrus-unshiu]-TRL> (1) ------------------MDAFEKLEKVGEGTYGKVYRAREKATGKIVALKKTRLHEDDEGVPPTTLREVSILRMLSRDPHVVRLMDVKQGQNKEGRTVLYLVFEYMDTD
                   XP_006473866.1-[Citrus-sinensis]-TRL> (1) ----------------MVMDAFEKLEKVGEGTYGKVYRAREKATGKIVALKKTRLHEDDEGVPPTTLREVSILRMLSRDPHVVRLMDVKQGQNKEGRTVLYLVFEYMDTD
     PQQ03509.1-[Prunus-yedoensis-var.-nudiflora]-ASL> (1) -----MESKAANTTTVSAMDAFEKLEKVGEGTYGKVYRAREKATGKIVALKKTRLHEDEEGVPPTTLREVSILRMLSRDPHIVRLMDVKQGQNKEGKTVLYLVFEYMDTD
```

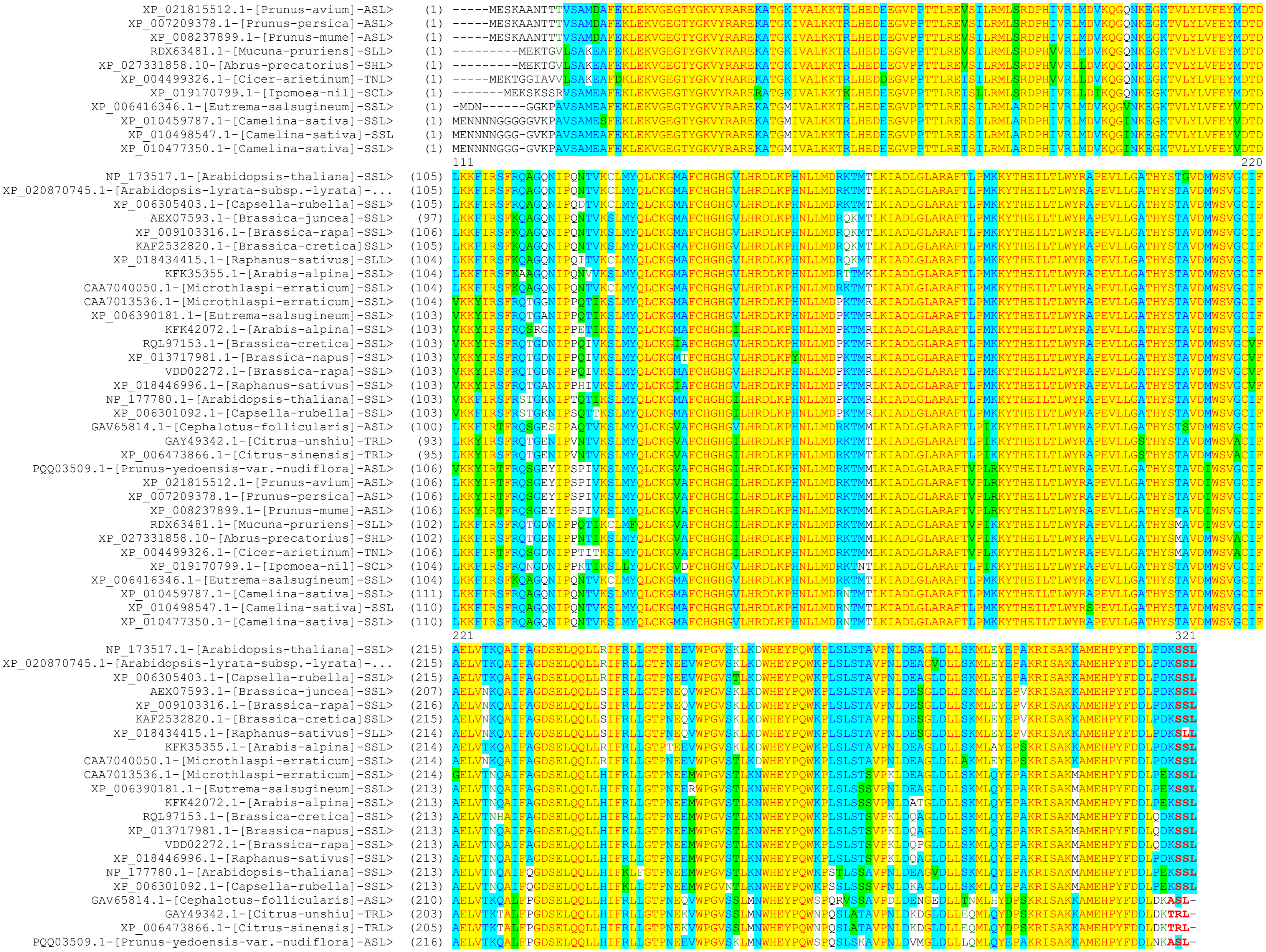

```
XP_021815512.1-[Prunus-avium]-ASL>  (216) AELVTKQALFPGDSELQQLLHIFRLLGTPNEEVWPGVSKLMNWHEYPQWSPQSLSKAVPNLDVMGLDLLLQMLQYEPAKRISAKKAMEHPYFDDLNKASL-
XP_007209378.1-[Prunus-persica]-ASL>  (216) AELVTKQALFPGDSELQQLLHIFRLLGTPNEEVWPGVSKLMNWHEYPQWNPQSLSKAVPNLDAMGLDLLLQMLQYEPAKRISAKKAMEHPYFDDLNKASL-
XP_008237899.1-[Prunus-mume]-ASL>  (216) AELVTKQALFPGDSELQQLLHIFRLLGTPNEEVWPGVSKLMNWHEYPQWSPQSLSKAVPNLDAMGLDLLLQMLQYEPAKRISAKKAMEHPYFDDLNKASL-
RDX63481.1-[Mucuna-pruriens]-SLL>  (212) AELVTKQALFPGDSELQQLLHIFRLLGTPNEEMWPGVSKLMNWHEYPQWNPQSLTTAVPSLDVLGLDLLSQMLKYEPSKRISAKKAMEHPYFDDLDKSLL-
XP_027331858.10-[Abrus-precatorius]-SHL>  (212) AELVTKQALFPGDSELQQLLHIFRLLGTPNEEMWPGVSKLMNWHEYPQWSPQRLSKAVPNLDELGLDLLSQMLKYEPSKRISAKKAMEHPYFDDLDKSHL-
XP_004499326.1-[Cicer-arietinum]-TNL>  (216) AELVTKQALFPGDSELQQLLHIFRLLGTPNEEMWPGVSKLMNWHEYPQWSPQSLSKAVPNLEEAGLDLLSQMLQYEPSKRISAKKAMEHPYFDGLDKTNL-
XP_019170799.1-[Ipomoea-nil]-SCL>  (214) AELVTNQALFPGDSELQQLLQIFRLLGTPNEEMWPGVSKLVNWHEYPQWRPKPLSSAVPNLDGDGLNLLGEMLQYEPSKRISAKKAMEHPYFDDLDKSCL-
XP_006416346.1-[Eutrema-salsugineum]-SSL>  (214) AELVTKQAIFAGDSELQQLLRIFRLLGTPNEEVWPGVSTLKDWHEYPQWKPLSLSTAVPNLDEAGLDLLSKMLVYEPAKRISAKKAMEHPYFDDLPDKSSL
XP_010459787.1-[Camelina-sativa]-SSL>  (221) AELVTKQAIFAGDSELQQLLRIFRLLGTPNEEVWPGVSTLKDWHEYPQWKPLSLSTAVPNLDEAGLDLLSKMLEYEPAKRISAKKAMEHPYFDDLPDKSSL
XP_010498547.1-[Camelina-sativa]-SSL  (220) AELVTKQAIFAGDSELQQLLRIFRLLGTPNEEVWPGVSTLKDWHEYPQWKPLSLSTAVPNLDEAGLDLLSKMLEYEPAKRISAKKAMEHPYFDDLPDKSSL
XP_010477350.1-[Camelina-sativa]-SSL>  (220) AELVTKQAIFAGDSELQQLLRIFRLLGTPNEEVWPGVSKLKDWHEYPQWKPLSLSTAVPNLDEAGLDLLSKMLEYEPAKRISAKKAMEHPYFDDLPDKSSL
```

PTD24_ AT1G66880.1 (Appeared in this study in Cytosol)

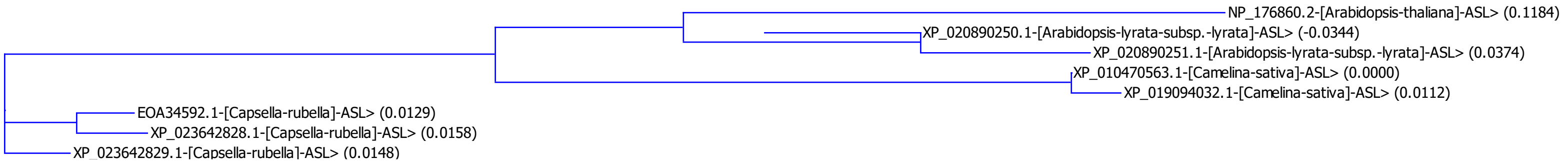


```
                                                         1                                                                                                          110
       NP_176860.2-[Arabidopsis-thaliana]-ASL>     (1) --------------------------------------------------------------------------------------------------------------
XP_020890250.1-[Arabidopsis-lyrata-subsp.-lyrata]-...     (1) --------------------------------------------------------------------------------------------------------------
XP_020890251.1-[Arabidopsis-lyrata-subsp.-lyrata]-...     (1) --------------------------------------------------------------------------------------------------------------
       XP_010470563.1-[Camelina-sativa]-ASL>     (1) --------------------------------------------------------------------------------------------------------------
       XP_019094032.1-[Camelina-sativa]-ASL>     (1) --------------------------------------------------------------------------------------------------------------
          EOA34592.1-[Capsella-rubella]-ASL>     (1) --------------------------------------------------------------------------------------------------------------
      XP_023642828.1-[Capsella-rubella]-ASL>     (1) MRKMENTNVNDLSQILPRVLVVSRRTLRKNKFVDFVGEYHLDLIVEYGAVPVIVPRVAGVHKLLESFKPIHGVLLCEGEDIDPSLYESELSSLSPQELDEIRKTHASDTA
      XP_023642829.1-[Capsella-rubella]-ASL>     (1) --------------------------------------------------------------------------------------------------------------
                                                         111                                                                                                        220
       NP_176860.2-[Arabidopsis-thaliana]-ASL>     (1) --------------------------------------------------------------------------------------------------------------
XP_020890250.1-[Arabidopsis-lyrata-subsp.-lyrata]-...     (1) --------------------------------------------------------------------------------------------------------------
XP_020890251.1-[Arabidopsis-lyrata-subsp.-lyrata]-...     (1) --------------------------------------------------------------------------------------------------------------
       XP_010470563.1-[Camelina-sativa]-ASL>     (1) --------------------------------------------------------------------------------------------------------------
       XP_019094032.1-[Camelina-sativa]-ASL>     (1) --------------------------------------------------------------------------------------------------------------
          EOA34592.1-[Capsella-rubella]-ASL>     (1) --------------------------------------------------------------------------------------------------------------
      XP_023642828.1-[Capsella-rubella]-ASL>   (111) IDKEKDSIEFALAKLCLEQNIPYLGICRGSQVLNVACGGSLYQDLETEVTNYVSEENRRNHIDYDDYDGYRHEVKIVKNSPLHKWFKDSLDEEKMEIFVNSYHHQGVKRL
      XP_023642829.1-[Capsella-rubella]-ASL>     (1) --------------------------------------------------------------------------------------------------------------
                                                         221                                                                                                        330
       NP_176860.2-[Arabidopsis-thaliana]-ASL>     (1) --------------------------------------------------------------------------------------------------------------
XP_020890250.1-[Arabidopsis-lyrata-subsp.-lyrata]-...     (1) --------------------------------------------------------------------------------------------------------------
XP_020890251.1-[Arabidopsis-lyrata-subsp.-lyrata]-...     (1) --------------------------------------------------------------------------------------------------------------
       XP_010470563.1-[Camelina-sativa]-ASL>     (1) --------------------------------------------------------------------------------------------------------------
       XP_019094032.1-[Camelina-sativa]-ASL>     (1) --------------------------------------------------------------------------------------------------------------
          EOA34592.1-[Capsella-rubella]-ASL>     (1) --------------------------------------------------------------------------------------------------------------
      XP_023642828.1-[Capsella-rubella]-ASL>   (221) AQRFVPMAFAPDGLIEGFYDPDMYNPEQGKFLVGLQFHPERMRKNGLEEFDFPGCPVAYQEFAKAVIACQKKLNSSLSVPKKLELNQEMENKRKILVRSFSLARSMYTRS
      XP_023642829.1-[Capsella-rubella]-ASL>     (1) --------------------------------------------------------------------------------------------------------------
                                                         331                                                                                                        440
       NP_176860.2-[Arabidopsis-thaliana]-ASL>     (1) -----------------------------------------------------------------MKNMNFERLSELMAFYHLMGKISSECGNITAGFPFWGGNRLKHCG
XP_020890250.1-[Arabidopsis-lyrata-subsp.-lyrata]-...     (1) --------------------------------------------------------------------------------------------------------------
XP_020890251.1-[Arabidopsis-lyrata-subsp.-lyrata]-...     (1) --------------------------------------------------------------------------------------------------------------
       XP_010470563.1-[Camelina-sativa]-ASL>     (1) --------------------------------------------------------------------------------------------------------------
       XP_019094032.1-[Camelina-sativa]-ASL>     (1) --------------------------------------------------------------------------------------------------------------
          EOA34592.1-[Capsella-rubella]-ASL>     (1) ----------------------------------------------------MNYLLTSCLFFFFFLLSLFHPLPCASGKQELGLCQDKFQCGNITAGFPFWGGNRHKDCG
      XP_023642828.1-[Capsella-rubella]-ASL>   (331) YSLNNQSMESELEVGAEFLESNTALSVKQETRLKEMGATMRNGGSFTEKLKLDEEKQRKAMNIMKKMNVERLSELITFYQLMGKISTEFQCGNITAGFPFWGGNRHKDCG
      XP_023642829.1-[Capsella-rubella]-ASL>     (1) --------------------------------------------------------------------------------------------------------------
                                                         441                                                                                                        550
       NP_176860.2-[Arabidopsis-thaliana]-ASL>    (46) LPSLELHCSK--NITSLFISSQEFYVLHLNQTSKTLKLARTDLLGSICNSTFTTITLPPNIFELSPTYKRLTVFYYCFLLTHYVSSYKCPMRGFIFVSENHEYHKICGDT
XP_020890250.1-[Arabidopsis-lyrata-subsp.-lyrata]-...     (1) --------------------------------------------------------------------------------------------------------------
XP_020890251.1-[Arabidopsis-lyrata-subsp.-lyrata]-...     (1) --------------------------------------------------------------------------------------------------------------
       XP_010470563.1-[Camelina-sativa]-ASL>     (1) --------------------------------------------------------------------------------------------------------------
       XP_019094032.1-[Camelina-sativa]-ASL>     (1) --------------------------------------------------------------------------------------------------------------
          EOA34592.1-[Capsella-rubella]-ASL>    (59) HPLLELHCDRENNNTSLIISKEKYSVFQLNQESNTLTLAKTKLLSSFCSSVTSETYLPKETFELSPTYKTIPVFYLCISSTHNLYTHTCPQMGSISASENPQYHKTCLQS
      XP_023642828.1-[Capsella-rubella]-ASL>   (441) HPLLELHCDRENNNTSLIISKEKYSVFQLNQESNTLTLAKTKLLSSFCSSVTSETYLPKETFELSPTYKTIPVFYLCISSTHNLYTHTCPQMGSISASENPQYHKTCLQS
      XP_023642829.1-[Capsella-rubella]-ASL>     (1) --------------------------------------------------------------------------------------------------------------
                                                         551                                                                                                        660
       NP_176860.2-[Arabidopsis-thaliana]-ASL>   (154) FTVIVPTRFGAGEKELNMTNLESVLSKGFEVKVKIDDTSCQECLSSHGSCGFNETLPVGGKCSPLNPTTQPPPQPGDRHKHCGHPLLELRCDQNKSTSLFISDQEFFVLH
XP_020890250.1-[Arabidopsis-lyrata-subsp.-lyrata]-...     (1) --------------------------------------------------------------------------------------------------------------
```

```
XP_020890251.1-[Arabidopsis-lyrata-subsp.-lyrata]-...    (1) --------------------------------------------------------------------------------------------------------------
          XP_010470563.1-[Camelina-sativa]-ASL>    (1) --------------------------------------------------------------------------------------------------------------
          XP_019094032.1-[Camelina-sativa]-ASL>    (1) --------------------------------------------------------------------------------------------------------------
              EOA34592.1-[Capsella-rubella]-ASL>  (169) FNVYVPTSFVTKEKELNMTNLKRVLQKGFEMKVKINKNACQDCVSSHGTCSFDETYPYEFNCIPLHPLHHLTGN----RPKDCGNSLLELHCDEDVTSLSISDQDFNILQ
          XP_023642828.1-[Capsella-rubella]-ASL>  (551) FNVYVPTSFVTKEKELNMTNLKRVLQKGFEMKVKINKNACQDCVSSHGTCSFDETYPYEFNCIPLHPLHHLTGN----RPKDCGNSLLELHCDEDVTSLSISDQDFNILQ
          XP_023642829.1-[Capsella-rubella]-ASL>    (1) --------------------------------------------------------------------------------------------------------------
                                                       661                                                                                                        770
          NP_176860.2-[Arabidopsis-thaliana]-ASL>  (264) VDQTSYSLTLARPDLLHSFCSLTFTNTTLPPEIFELSPAYKSVTFYH-CYPVLPDLSNYTCPVIGPISVSGNPEDHETCFPNFAANVPTSFVTKEKKLNIANLESVLEKG
XP_020890250.1-[Arabidopsis-lyrata-subsp.-lyrata]-...    (1) --------------------------------------------------------------------------------------------------------------
XP_020890251.1-[Arabidopsis-lyrata-subsp.-lyrata]-...    (1) --------------------------------------------------------------------------------------------------------------
          XP_010470563.1-[Camelina-sativa]-ASL>    (1) --------------------------------------------------------------------------------------------------------------
          XP_019094032.1-[Camelina-sativa]-ASL>    (1) --------------------------------------------------------------------------------------------------------------
              EOA34592.1-[Capsella-rubella]-ASL>  (275) INQTSNTLTLARTDHLGSFCSSTFTNTALPPELFELSPNYQSLTVYYNCICHDPYLSKNTCPRLGNYSVSQIPKYNEFCHDKFTVNVPKSFVPGEKEFSWTSLENVLCKG
          XP_023642828.1-[Capsella-rubella]-ASL>  (657) INQTSNTLTLARTDHLGSFCSSTFTNTALPPELFELSPNYQSLTVYYNCICHDPYLSKNTCPRLGNYSVSQIPKYNEFCHDKFTVNVPKSFVPGEKEFSWTSLENVLCKG
          XP_023642829.1-[Capsella-rubella]-ASL>    (1) --------------------------------------------------------------------------------------------------------------
                                                       771                                                                                                        880
          NP_176860.2-[Arabidopsis-thaliana]-ASL>  (373) FEVNMNVIMKACQACSYSNESCGFDENFPFEVKCKPHHSPTAKFQCGNITAGFPFSGGNRPQICGHPSLELHCYNNMASIIISDHFYNVLHIDQTSNTLRLARAELEGSF
XP_020890250.1-[Arabidopsis-lyrata-subsp.-lyrata]-...    (1) -------MYYLPTSCLFLFFLFSSFHPLPYASSKQELGWCEAQFQCGDITAGFPFSGGSRPQICGHPSLVLHCFNNKTSIIISDHFYNVLHIDQTSNTLRLARAELEGSF
XP_020890251.1-[Arabidopsis-lyrata-subsp.-lyrata]-...    (1) -------MYYLPTSCLFLFFLFSSFHPLPYASSKQELGWCEAQFQCGDITAGFPFSGGSRPQICGHPSLVLHCFNNKTSIIISDHFYNVLHIDQTSNTLRLARAELEGSF
          XP_010470563.1-[Camelina-sativa]-ASL>    (1) --------------------------------------------------------------------------------------------------------------
          XP_019094032.1-[Camelina-sativa]-ASL>    (1) --------------------------------------------------------------------------------------------------------------
              EOA34592.1-[Capsella-rubella]-ASL>  (385) FEVKVKIDAKACQDCSSSLEHCEFNKALPLGAKCSPLHQPTAMFQCGNITAGFPFSGANRPQICGHPSLELHCFNNKTSIIISDHLYNVLHIDEKSHTLRLARAELEGSF
          XP_023642828.1-[Capsella-rubella]-ASL>  (767) FEVKVKIDAKACQDCSSSLEHCEFNKALPLGAKCSPLHQPTAMFQCGNITAGFPFSGANRPQICGHPSLELHCFNNKTSIIISDHLYNVLHIDEKSHTLRLARAELEGSF
          XP_023642829.1-[Capsella-rubella]-ASL>    (1) --------------------------------------------------------------------------------------------------------------
                                                       881                                                                                                        990
          NP_176860.2-[Arabidopsis-thaliana]-ASL>  (483) CNATYTATTLPSKIFEISSTYKSLTVFYLCDPKVSYRSSYTCPGRGLVSVSQNSDYHNSCQDSFTINVPKSFVPEEKELDVTNLESALREGFEVKVKVDEKTCQKCTSSG
XP_020890250.1-[Arabidopsis-lyrata-subsp.-lyrata]-...  (104) CNATYTATTLPSDIFELSSTYTSLTVFYLCDPKLSYRSSYTCPGRGLISVSQNLDYKYSCQDSFTINVPKSFVPEEKELNLRKLEKAFQEGFEVKVKIDEKACQKCSSSG
XP_020890251.1-[Arabidopsis-lyrata-subsp.-lyrata]-...  (104) CNATYTATTLPSDIFELSSTYTSLTVFYLCDPKLSYRSSYTCPGRGLISVSQNLDYKYSCQDSFTINVPKSFVPEEKELNLRKLEKAFQEGFEVKVKIDEKACQKCSSSG
          XP_010470563.1-[Camelina-sativa]-ASL>    (1) --------------------------------------------------------------------------------------------------------------
          XP_019094032.1-[Camelina-sativa]-ASL>    (1) --------------------------------------------------------------------------------------------------------------
              EOA34592.1-[Capsella-rubella]-ASL>  (495) CEATFTSTTLPSELFQLSSTYKSLTVFYLCDSNLSYSSSYTCPGRGLVSVSETLDYQNSCQASFPVNVPKNFLLEEKELNLTHLEKALREGFEVKVKIDEKACQECSSSG
          XP_023642828.1-[Capsella-rubella]-ASL>  (877) CEATFTSTTLPSELFQLSSTYKSLTVFYLCDSNLSYSSSYTCPGRGLVSVSETLDYQNSCQASFPVNVPKNFLLEEKELNLTHLEKALREGFEVKVKIDEKACQECSSSG
          XP_023642829.1-[Capsella-rubella]-ASL>    (1) --------------------------------------------------------------------------------------------------------------
                                                       991                                                                                                       1100
          NP_176860.2-[Arabidopsis-thaliana]-ASL>  (593) GTCGFQNSTQICCKEASSLGCNKVHPLPDATEL--ELHRRCNGTFSCGDQRELFYPFWTS-GREDCGHPDFKLDDCSGRFAELSISSVKFRILASVYGSNIIRLGRSEYI
XP_020890250.1-[Arabidopsis-lyrata-subsp.-lyrata]-...  (214) GICGFENSTQICCKQASSSGCNKLHPLLDDK------------------------------G------------------------------------------------
XP_020890251.1-[Arabidopsis-lyrata-subsp.-lyrata]-...  (214) GICGFENSTQICCKQASSSGCNKLHPLLDAD----ELHRLCSPSFTCGNQSGFLYPFWIA-GRKECGHPDFELECGTG-FPELSISSVNFRILAANYDSGIIRLARSDKI
          XP_010470563.1-[Camelina-sativa]-ASL>    (1) -MINLSLSLSKSIAYSFIWMLFVILPSYVFSA--EQHHVLCSPSFSCGNQSGLLYPFWIP-GRKDCGHPDFELD-CSGGFPELSISSVKFQILAANYDSGIIKLARSDYS
          XP_019094032.1-[Camelina-sativa]-ASL>    (1) -MINLSLSLSKSIAYSFIWMLFVILPSYVFSA--EQHHVLCSPSFSCGNQSGLLYPFWIP-GRKDCGHPDFELD-CSGGFPELSISSVKFQILAANYDSGIIKLARSDYS
              EOA34592.1-[Capsella-rubella]-ASL>  (605) GTCGFQNSTQICCKESSPSSCYKLHSLNANNS---QIHKRCSGSFSCGNQTELFYPFWKSGGREDCGHPEFKLDDCSAGFPEFSLSSVKFRILEADYITGIIRLARLDYT
          XP_023642828.1-[Capsella-rubella]-ASL>  (987) GTCGFQNSTQICCKESSPSSCYKLHSLNANNS---QIHKRCSGSFSCGNQTELFYPFWKSGGREDCGHPEFKLDDCSAGFPEFSLSSVKFRILEADYITGIIRLARLDYT
          XP_023642829.1-[Capsella-rubella]-ASL>    (1) -MIHLSLSVTKSMSYSFLLWMFFLIPSCVFSANNSQIHKRCSGSFSCGNQTELFYPFWKSGGREDCGHPEFKLDDCSAGFPEFSLSSVKFRILEADYITGIIRLARLDYT
                                                       1101                                                                                                      1210
          NP_176860.2-[Arabidopsis-thaliana]-ASL>  (700) GDLCPQDPINAPFSESVLPFAPNTELLTIFYNCSRDFPQQVTNFG-DFACGDDSDDDR-SYYVTRNLSFPPLSEINDLLYDFSQSCDRNVSIPASGSTLNILQSTPSNDN
XP_020890250.1-[Arabidopsis-lyrata-subsp.-lyrata]-...  (246) --------------------------------------------------------------------------------------------------------------
XP_020890251.1-[Arabidopsis-lyrata-subsp.-lyrata]-...  (318) DDPCLQGSLTTSFNETILPLAPTTDLLTIYYDCSRDFSSFVSTFVKDLDCPDDETDDRGNYYVTRNLLSPVLQGISNALNDFRRFCSRNVSIPASITALSTLESSPSTDN
          XP_010470563.1-[Camelina-sativa]-ASL>  (106) DDPCSQDSLKISFNETVLPIAPTTDWLTVYYDCNQDFSAFVSTYVKELDCG---NDDSKNYYVTRNLVSSLLEGYSPVFIDFRRSCSINVNTPASRTALNTLESSPSRDN
          XP_019094032.1-[Camelina-sativa]-ASL>  (106) DDPCSQDSLKISFNETVLPIAPTTDWLTVYYDCNQDFSAFVSTYVKELDCG---NDDSKNYYVTRNLVSSLLEGYSPVFIDFRRSCSINVNTPASRTALNTLESSPSRDN
              EOA34592.1-[Capsella-rubella]-ASL>  (712) EDICPDHPIDAPFNGSILPFASNTKLLTIYSHCSNDLPYPVPTNVKELACG-DETDDEIRYYVTRDLSFSPLDQISTLLNEFKRYCDRNVSIPVSGPTLNTLQLTWSRDN
          XP_023642828.1-[Capsella-rubella]-ASL> (1094) EDICPDHPIDAPFNGSILPFASNTKLLTIYSHCSNDLPYPVPTNVKELACG-DETDDEIRYYVTRDLSFSPLDQISTLLNEFKRYCDRNVSIPVSGPTLNTLQLTWSRDN
          XP_023642829.1-[Capsella-rubella]-ASL>  (110) EDICPDHPIDAPFNGSILPFASNTKLLTIYSHCSNDLPYPVPTNVKELACG-DETDDEIRYYVTRDLSFSPLDQISTLLNEFKRYCDRNVSIPVSGPTLNTLQLTWSRDN
                                                       1211                                                                                                      1320
          NP_176860.2-[Arabidopsis-thaliana]-ASL>  (808) LKKALEYGFELELNQDCRTCIDSKGACGYSQTSSR--FVCYSIEE-PQTPTPPNPTRNKDTSLSIGAKAGIAVASVSGLAILLLAGLFLCIRRRRKTQDAQYTSKSLPIT
XP_020890250.1-[Arabidopsis-lyrata-subsp.-lyrata]-...  (246) --------------------------------------------------------------LSNGAKAGIAVASVSGLAILILAGVFLCIRRRRKTQDAEYTNKSLPIT
XP_020890251.1-[Arabidopsis-lyrata-subsp.-lyrata]-...  (428) LKKALEDRFELQVNSDCRTCIDSKGACGFSQTSSK--FECYYIEK-PQTPQVDSSARNKDKGLSNGAKAGIAVASVSGLAILILAGVFLCIRRRRKTQDAEYTNKSLPIT
          XP_010470563.1-[Camelina-sativa]-ASL>  (213) LNKAIEESFEVGVNLDCKKCRDSGGACGYNHTLSTSGFVCYCIDDQPGNGVCGSTNKDGKKSTKANTGIIAAAVSISGVLLLVVACVFLLIRRRRKTQEAQYTSKDLPTT
          XP_019094032.1-[Camelina-sativa]-ASL>  (213) LNKAIEESFEVGVNLDCKKCRDSGGACGYNHTLSTSGFVCYCIDDQPGNGVCGSTNKDGKKSTKANTGIIAAAVSISGVLLLVVACVFLLIRRRRKTQEAQYTSKDLPTT
              EOA34592.1-[Capsella-rubella]-ASL>  (821) LKKAFEDGFQLGPNQECPKCIESGGACGYNQSSSS--FTCYYMEE-P-------------------HKG-IVAGSIAGLAILTLACLVCFIRRRRKTQEAQYTSKDLPIT
          XP_023642828.1-[Capsella-rubella]-ASL> (1203) LKKAFEDGFQLGPNQECPKCIESGGACGYNQSSSS--FTCYYMEE-PHKG------------LSTGAIAGIVAGSIAGLAILTLACLVCFIRRRRKTQEAQYTSKDLPIT
          XP_023642829.1-[Capsella-rubella]-ASL>  (219) LKKAFEDGFQLGPNQECPKCIESGGACGYNQSSSS--FTCYYMEE-PHKGKISDILTMKIVLLFS--SPGIVAGSIAGLAILTLACLVCFIRRRRKTQEAQYTSKDLPIT
                                                       1321                                                                                                      1430
          NP_176860.2-[Arabidopsis-thaliana]-ASL>  (915) SYSSRDTSRNPTSTTISSSSNHSLLP--SISNLANRSDYCGVQVFSYEELEEATENFSRELGDGGFGTVYYGVLKDGRAVAVKRLYERSLKRVEQFKNEIEILKSLKHPN
XP_020890250.1-[Arabidopsis-lyrata-subsp.-lyrata]-...  (294) SYSSRETSRNPTSTTISYSSNHSFLP--SISNLANGSDYYGVQVFSYEELEEATENFSRELGDGGFGTVYYGVLKDGRAVAVKRLYERSLKRVEQFKNEIEILKSLKHPN
XP_020890251.1-[Arabidopsis-lyrata-subsp.-lyrata]-...  (535) SYSSRETSRNPTSTTISYSSNHSFLP--SISNLANGSDYYGVQVFSYEELEEATENFSRELGDGGFGTVYYGVLKDGRAVAVKRLYERSLKRVEQFKNEIEILKSLKHPN
          XP_010470563.1-[Camelina-sativa]-ASL>  (323) SYSSRETSRNPTSTTISSSSNHSYLPSMSISNLPNGSDYYGVQVFSYEELEEATENFSRELGDGGFGTVYYGMLKDGRAVAVKRLYERSLKRVEQFKNEIEILKRIKHQN
          XP_019094032.1-[Camelina-sativa]-ASL>  (323) SYSSRETSRNPTSTTISSSSNHSYLPSMSISNLPNGSDYYGVQVFSYEELEEATENFSRELGDGGFGTVYYGMLKDGRAVAVKRLYERSLKRVEQFKNEIEILKRIKHQN
              EOA34592.1-[Capsella-rubella]-ASL>  (908) SYSSRETSRNPTSTTISSSSNHSLLP--SLSNIPNGSDYYGVQVFSYEELDEATGNFSRELGDGGFGTVYYGILKDGRAVAVKRLYERSLKRVEQFKNEIEILKSLKHPN
          XP_023642828.1-[Capsella-rubella]-ASL> (1298) SYSSRETSRNPTSTTISSSSNHSLLP--SLSNIPNGSDYYGVQVFSYEELDEATGNFSRELGDGGFGTVYYGILKDGRAVAVKRLYERSLKRVEQFKNEIEILKSLKHPN
```

```
XP_023642829.1-[Capsella-rubella]-ASL>   (324) SYSSRETSRNPTSTTISSSSNHSLLP--SLSNIPNGSDYYGVQVFSYEELDEATGNFSRELGDGGFGTVYYGILKDGRAVAVKRLYERSLKRVEQFKNEIEILKSLKHPN
                                                1431                                                                                                      1540
NP_176860.2-[Arabidopsis-thaliana]-ASL>  (1023) LVILYGCTSRHSRELLLVYEYISNGTLAEHLHGNRAEARPLCWSTRLNIAIETASALSFLHIKGIIHRDIKTTNILLDDNYQVKVADFGLSRLFPMDQTHISTAPQGTPG
XP_020890250.1-[Arabidopsis-lyrata-subsp.-lyrata]-...   (402) LVILYGCTSRHSRELLLVYEYISNGTLAEHLHGNRAETRPLCWSTRLNIAIQTASALSYLHKSEVIHRDVKTTNILLDDNYQVKVADFGLSRLFPMDQTHISTAPQGTPG
XP_020890251.1-[Arabidopsis-lyrata-subsp.-lyrata]-...   (643) LVILYGCTSRHSRELLLVYEYISNGTLAEHLHGNRAETRPLCWSTRLNIAIQTASALSYLHKSEVIHRDVKTTNILLDDNYQVKVADFGLSRLFPMDQTHISTAPQGTPG
XP_010470563.1-[Camelina-sativa]-ASL>   (433) LVILYGCTSRHSRELLLVYEYISNGTLAEHLHGDRAEARPLSWSTRLNVAIETANALSFLHKSEIIHRDVKTTNILLEDNYQVKVADFGLSRLFPMDQTHISTAPQGTPG
XP_019094032.1-[Camelina-sativa]-ASL>   (433) LVILYGCTSRHSRELLLVYEYISNGTLAEHLHGDRAEARPLSWSTRLNVAIETANALSFLHKSEIIHRDVKTTNILLEDNYQVKVADFGLSRLFPMDQTHISTAPQGTPG
EOA34592.1-[Capsella-rubella]-ASL>  (1016) LVILYGCTSGRSRELLLVYEYISNGTLAEHLHGDRAEARPLSWSTRLNVAIETANALSYLHKSGIIHRDVKTTNILLDDNHQVKVADFGLSRLFPMDQTHISTAPQGTPG
XP_023642828.1-[Capsella-rubella]-ASL>  (1406) LVILYGCTSGRSRELLLVYEYISNGTLAEHLHGDRAEARPLSWSTRLNVAIETANALSYLHKSGIIHRDVKTTNILLDDNHQVKVADFGLSRLFPMDQTHISTAPQGTPG
XP_023642829.1-[Capsella-rubella]-ASL>   (432) LVILYGCTSGRSRELLLVYEYISNGTLAEHLHGDRAEARPLSWSTRLNVAIETANALSYLHKSGIIHRDVKTTNILLDDNHQVKVADFGLSRLFPMDQTHISTAPQGTPG
                                                1541                                                                                                      1650
NP_176860.2-[Arabidopsis-thaliana]-ASL>  (1133) YVDPEYYQCYQLNEKSDVYSFGVVLTELISSKEAVDITRHRHDINLANMAVSKIQNNALHELVDSSLGYDNDPEVRRKMMAVAELAFRCLQQERDVRPAMDEIVEILRGI
XP_020890250.1-[Arabidopsis-lyrata-subsp.-lyrata]-...   (512) YVDPEYYQCYSLNEKSDVYSFGVVLSELISSKEAVDITRHRHDINLANMAVSKIQNNALHELVDSSLGFDNDPEVRRKMMSVAELAFRCLQQEREVRPSMDEIVEILRGV
XP_020890251.1-[Arabidopsis-lyrata-subsp.-lyrata]-...   (753) YVDPEYYQCYSLNEKSDVYSFGVVLSELISSKEAVDITRHRHDINLANMAVSKIQNNALHELVDSSLGFDNDPEVRRKMMSVAELAFRCLQQEREVRPSMDEIVEILRGV
XP_010470563.1-[Camelina-sativa]-ASL>   (543) YVDPEYYQCYRLNEKSDVYSFGVVLTELISSKEAVDITRHRHDINLANMAVSKIQNNALHELVDPSLGFEKDLDVRRKMMSVAELAFRCLQQEREVRPSMDEIVEILKGI
XP_019094032.1-[Camelina-sativa]-ASL>   (543) YVDPEYYQCYRLNEKSDVYSFGVVLTELISSKEAVDITRHRHDINLANMAVSKIQNNALHELVDPSLGFEKDLDVRRKMMSVAELAFRCLQQEREVRPSMDEIVEILKGI
EOA34592.1-[Capsella-rubella]-ASL>  (1126) YVDPEYYQCYRLNEKSDVYSFGVVLTELISSKEAVDITRHRHDINLANMAVSKIQNNALHELVDPSLGFDKDPEIRRKMMSVAELAFRCLQQEREVRPAMDEIVEILKGI
XP_023642828.1-[Capsella-rubella]-ASL>  (1516) YVDPEYYQCYRLNEKSDVYSFGVVLTELISSKEAVDITRHRHDINLANMAVSKIQNNALHELVDPSLGFDKDPEIRRKMMSVAELAFRCLQQEREVRPAMDEIVEILKGI
XP_023642829.1-[Capsella-rubella]-ASL>   (542) YVDPEYYQCYRLNEKSDVYSFGVVLTELISSKEAVDITRHRHDINLANMAVSKIQNNALHELVDPSLGFDKDPEIRRKMMSVAELAFRCLQQEREVRPAMDEIVEILKGI
                                                1651                                                 1707
NP_176860.2-[Arabidopsis-thaliana]-ASL>  (1243) KDDEKKRVLVKSPDVVDIECGGG---DDVGLLRNSVPPPISPETDKWTSSSDTAASL
XP_020890250.1-[Arabidopsis-lyrata-subsp.-lyrata]-...   (622) KEEEEKRVLVKSPDVVDIECGGG---DDVGLLRNSVPPPISPETDKWTSSSDTAASL
XP_020890251.1-[Arabidopsis-lyrata-subsp.-lyrata]-...   (863) KEEEEKRVLVKSPDVVDIECGGG---DDVGLLRNSVPPPISPETDKWTSSSDTAASL
XP_010470563.1-[Camelina-sativa]-ASL>   (653) KDGEKRVGVVESPDVVDIEHGGGG--DDVGLLRNSVPPPISPETDKWTSSSDTAASL
XP_019094032.1-[Camelina-sativa]-ASL>   (653) KDGEKRVGVVESTPDVVDIERGG---DDVGLLRNSVPPPISPETDKWTSSSDTAASL
EOA34592.1-[Capsella-rubella]-ASL>  (1236) KDEEKRVVVVESPDVVDIERGGGGGGDDVGLLRNSIPPPISPDTDKWTSSSDTAASL
XP_023642828.1-[Capsella-rubella]-ASL>  (1626) KDEEKRVVVVESPDVVDIERGGGGGGDDVGLLRNSIPPPISPDTDKWTSSSDTAASL
XP_023642829.1-[Capsella-rubella]-ASL>   (652) KDEEKRVVVVESPDVVDIERGGGGGGDDVGLLRNSIPPPISPDTDKWTSSSDTAASL
```

PTD26_ AT4G18950.1 (Appeared in this study in Cytosol)

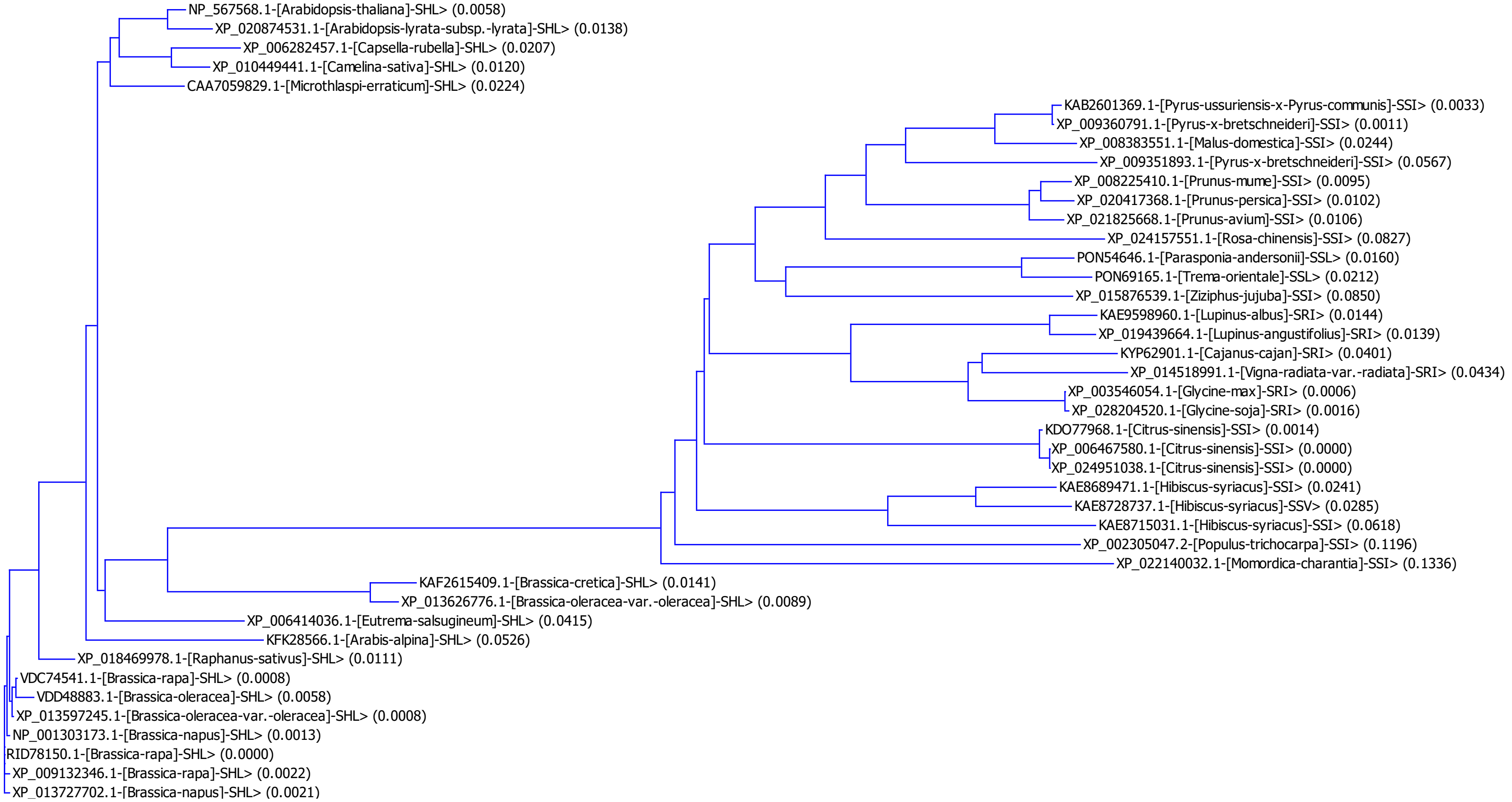


```
                                                          1                                                                                                              110
                   NP_567568.1-[Arabidopsis-thaliana]-SHL>  (1) ---------MEEDYQQPRFTIGRQSSMAPEK---IPEPSVHSEEEVFEDGEEIDGGVRLMYLANEGDIEGIKELIDSGIDANYRDIDDRTALHVAACQGLKDVVELLLDR
XP_020874531.1-[Arabidopsis-lyrata-subsp.-lyrata]-...  (1) ---------MEEDYLQPRFTIGRQSSLAPEK---IPEPSVHSEEEVFEDGEEIDGGVRLMYLANEGDIEGIKELLDSGIDANYRDIDDRTALHVAACQGLKDVVELLLDR
              XP_006282457.1-[Capsella-rubella]-SHL>  (1) ---------MEEEYLQPRFTIGRQSSMAPDK---VPEPSIHSEEEVLEEGDEIDGGVRLMYLSNEGDIEGIRELLDSGIDANYRDIDDRTALHVAACQGLKDVVELLLDR
               XP_010449441.1-[Camelina-sativa]-SHL>  (1) ---------MEEEYLVPRFTIGRQSSMAPDK---VPEPSIHSEEEVLEDGEEIDGGVRLMYLSNEGDIEGIKELLDSGIDANYRDIDDRTALHVAACQGLKDVVELLLDR
         CAA7059829.1-[Microthlaspi-erraticum]-SHL>  (1) MSFVGFSRDTEEDYQTPRWTIGRQSSMAPDK---NPEPSIHSEEEVLEDGEEIDGGVRLMYLSNEGDIEGIKELLDSGIDANYRDIDDRTALHVAACQGLKDVVELLLDR
KAB2601369.1-[Pyrus-ussuriensis-x-Pyrus-communis]-...  (1) ---------MDVKATPLRFPLGRQSSLAPER-----ELEERVGEGLEDDPEGIDPGVKLMYLANEADLDGIKELLDSGVDVNFRDIDDRTALHVAACQGLTDVVSLLLDR
        XP_009360791.1-[Pyrus-x-bretschneideri]-SSI>  (1) ---------MDLKATPLRFPLGRQSSLAPER-----ELEERVGEGLEDDPEGIDPGVKLMYLANEADLDGIKELLDSGVDVNFRDIDDRTALHVAACQGLTDVVSLLLDR
              XP_008383551.1-[Malus-domestica]-SSI>  (1) ---------MDLKATPLRFTLGRQSSLAPELE-----REERVGEGQEDDPEGIDPRVKLMYLANEADLDGIKELLDSGVDVNFRDIDDRTALHVAACQGLTDVVSLLLDR
        XP_009351893.1-[Pyrus-x-bretschneideri]-SSI>  (1) ---------MDLKTQPLRFTLGKQSSLAPER-------EWEKRVGEEDDQEGIDPRVKLMYLANEADLDGIKEVLDSGLDVNFRDIDDRTALHVAACQGLTNVVSLLLDR
                  XP_008225410.1-[Prunus-mume]-SSI>  (1) ---------MDVKTTPLRFTLGKQSSLAPEC------EWEELVGEGEDDPEGIDPRVKLMYLANEADLDGVKELLDSGIDVNFKDIDDRTALHVAACQGLTDVVSLLLER
               XP_020417368.1-[Prunus-persica]-SSI>  (1) ---------MDVKTTPLRFTLGKQSSLAPEC-----EWEKLVVGEGEDDPEGIDPRVKLMYLANEADLDGVKELLDSGIDVNFKDIDDRTALHVAACQGLTDVVSLLLER
                 XP_021825668.1-[Prunus-avium]-SSI>  (1) ---------MDVKTTPLRFTLGKQSSLAPEG-----EWEELVAGEGEDYPEGIDPRVKLMYLANEADLDGVKELLDSGIDVNFRDIDDRTALHVAACQGLTDVVSLLLER
               XP_024157551.1-[Rosa-chinensis]-SSI>  (1) ---------MDLKTTPLRFTLGKQSSLAPER-------ESDAGDAEEDEVEVIDPRVRLMYLANEADLDGIKELLDSGIDVNFRDIDNRTALHVAACQGYSDVVSLLLER
           PON54646.1-[Parasponia-andersonii]-SSL>  (1) --------METAKTTALRFTLGKQSSMAPER--------DDSEPRKEXESVRIDPRVRLMYLANEGDLDGIRELLESGIDVNFRDIDDRTALHVAACQGFYDVIQLLLER
                 PON69165.1-[Trema-orientale]-SSL>  (1) --------METAKTTALRFTLGKQSSMAPEPDDSEPRKREDGDGDGDDESVRIDPRVRLMYLANEGDLDGIRELLESGIDVNFRDIDDRTALHVAACQGFYDVVQLLLER
              XP_015876539.1-[Ziziphus-jujuba]-SSI>  (1) ---------MEMRTTPLRFALGKQSSLAPARG------SDRRNKPEVDSGGEVNPGVRLMYLANEADLDGIRELLDSGIDVNFRDIDNRTALHIAACQGLTDVVALLLQR
                KAE9598960.1-[Lupinus-albus]-SRI>  (1) ----------MESKTPARFTLGKQSSLAPERHG-----EESGSHKDGESVEGVDSGVRLMYSANEGDLDGIREVLESGVSVNFRDIDYRTPLHIAACQGLTDVVALLLEK
```

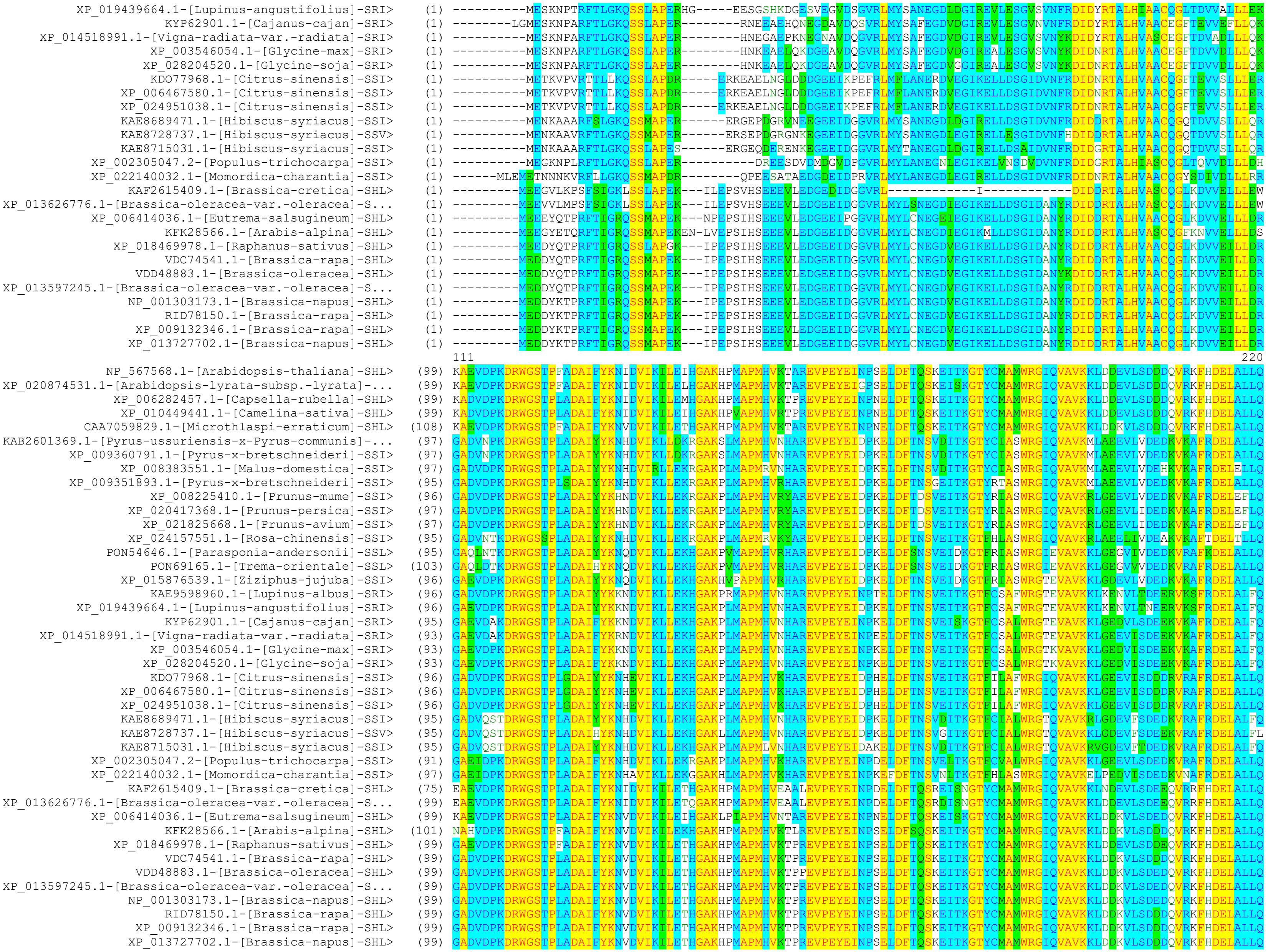
XP_019439664.1-[Lupinus-angustifolius]-SRI> (1)
KYP62901.1-[Cajanus-cajan]-SRI> (1)
XP_014518991.1-[Vigna-radiata-var.-radiata]-SRI> (1)
XP_003546054.1-[Glycine-max]-SRI> (1)
XP_028204520.1-[Glycine-soja]-SRI> (1)
KDO77968.1-[Citrus-sinensis]-SSI> (1)
XP_006467580.1-[Citrus-sinensis]-SSI> (1)
XP_024951038.1-[Citrus-sinensis]-SSI> (1)
KAE8689471.1-[Hibiscus-syriacus]-SSI> (1)
KAE8728737.1-[Hibiscus-syriacus]-SSV> (1)
KAE8715031.1-[Hibiscus-syriacus]-SSI> (1)
XP_002305047.2-[Populus-trichocarpa]-SSI> (1)
XP_022140032.1-[Momordica-charantia]-SSI> (1)
KAF2615409.1-[Brassica-cretica]-SHL> (1)
XP_013626776.1-[Brassica-oleracea-var.-oleracea]-S... (1)
XP_006414036.1-[Eutrema-salsugineum]-SHL> (1)
KFK28566.1-[Arabis-alpina]-SHL> (1)
XP_018469978.1-[Raphanus-sativus]-SHL> (1)
VDC74541.1-[Brassica-rapa]-SHL> (1)
VDD48883.1-[Brassica-oleracea]-SHL> (1)
XP_013597245.1-[Brassica-oleracea-var.-oleracea]-S... (1)
NP_001303173.1-[Brassica-napus]-SHL> (1)
RID78150.1-[Brassica-rapa]-SHL> (1)
XP_009132346.1-[Brassica-rapa]-SHL> (1)
XP_013727702.1-[Brassica-napus]-SHL> (1)
111
220
NP_567568.1-[Arabidopsis-thaliana]-SHL> (99)
XP_020874531.1-[Arabidopsis-lyrata-subsp.-lyrata]-... (99)
XP_006282457.1-[Capsella-rubella]-SHL> (99)
XP_010449441.1-[Camelina-sativa]-SHL> (99)
CAA7059829.1-[Microthlaspi-erraticum]-SHL> (108)
KAB2601369.1-[Pyrus-ussuriensis-x-Pyrus-communis]-... (97)
XP_009360791.1-[Pyrus-x-bretschneideri]-SSI> (97)
XP_008383551.1-[Malus-domestica]-SSI> (97)
XP_009351893.1-[Pyrus-x-bretschneideri]-SSI> (95)
XP_008225410.1-[Prunus-mume]-SSI> (96)
XP_020417368.1-[Prunus-persica]-SSI> (97)
XP_021825668.1-[Prunus-avium]-SSI> (97)
XP_024157551.1-[Rosa-chinensis]-SSI> (95)
PON54646.1-[Parasponia-andersonii]-SSL> (95)
PON69165.1-[Trema-orientale]-SSL> (103)
XP_015876539.1-[Ziziphus-jujuba]-SSI> (96)
KAE9598960.1-[Lupinus-albus]-SRI> (96)
XP_019439664.1-[Lupinus-angustifolius]-SRI> (96)
KYP62901.1-[Cajanus-cajan]-SRI> (95)
XP_014518991.1-[Vigna-radiata-var.-radiata]-SRI> (93)
XP_003546054.1-[Glycine-max]-SRI> (93)
XP_028204520.1-[Glycine-soja]-SRI> (93)
KDO77968.1-[Citrus-sinensis]-SSI> (96)
XP_006467580.1-[Citrus-sinensis]-SSI> (96)
XP_024951038.1-[Citrus-sinensis]-SSI> (96)
KAE8689471.1-[Hibiscus-syriacus]-SSI> (95)
KAE8728737.1-[Hibiscus-syriacus]-SSV> (95)
KAE8715031.1-[Hibiscus-syriacus]-SSI> (95)
XP_002305047.2-[Populus-trichocarpa]-SSI> (91)
XP_022140032.1-[Momordica-charantia]-SSI> (97)
KAF2615409.1-[Brassica-cretica]-SHL> (75)
XP_013626776.1-[Brassica-oleracea-var.-oleracea]-S... (99)
XP_006414036.1-[Eutrema-salsugineum]-SHL> (99)
KFK28566.1-[Arabis-alpina]-SHL> (101)
XP_018469978.1-[Raphanus-sativus]-SHL> (99)
VDC74541.1-[Brassica-rapa]-SHL> (99)
VDD48883.1-[Brassica-oleracea]-SHL> (99)
XP_013597245.1-[Brassica-oleracea-var.-oleracea]-S... (99)
NP_001303173.1-[Brassica-napus]-SHL> (99)
RID78150.1-[Brassica-rapa]-SHL> (99)
XP_009132346.1-[Brassica-rapa]-SHL> (99)
XP_013727702.1-[Brassica-napus]-SHL> (99)

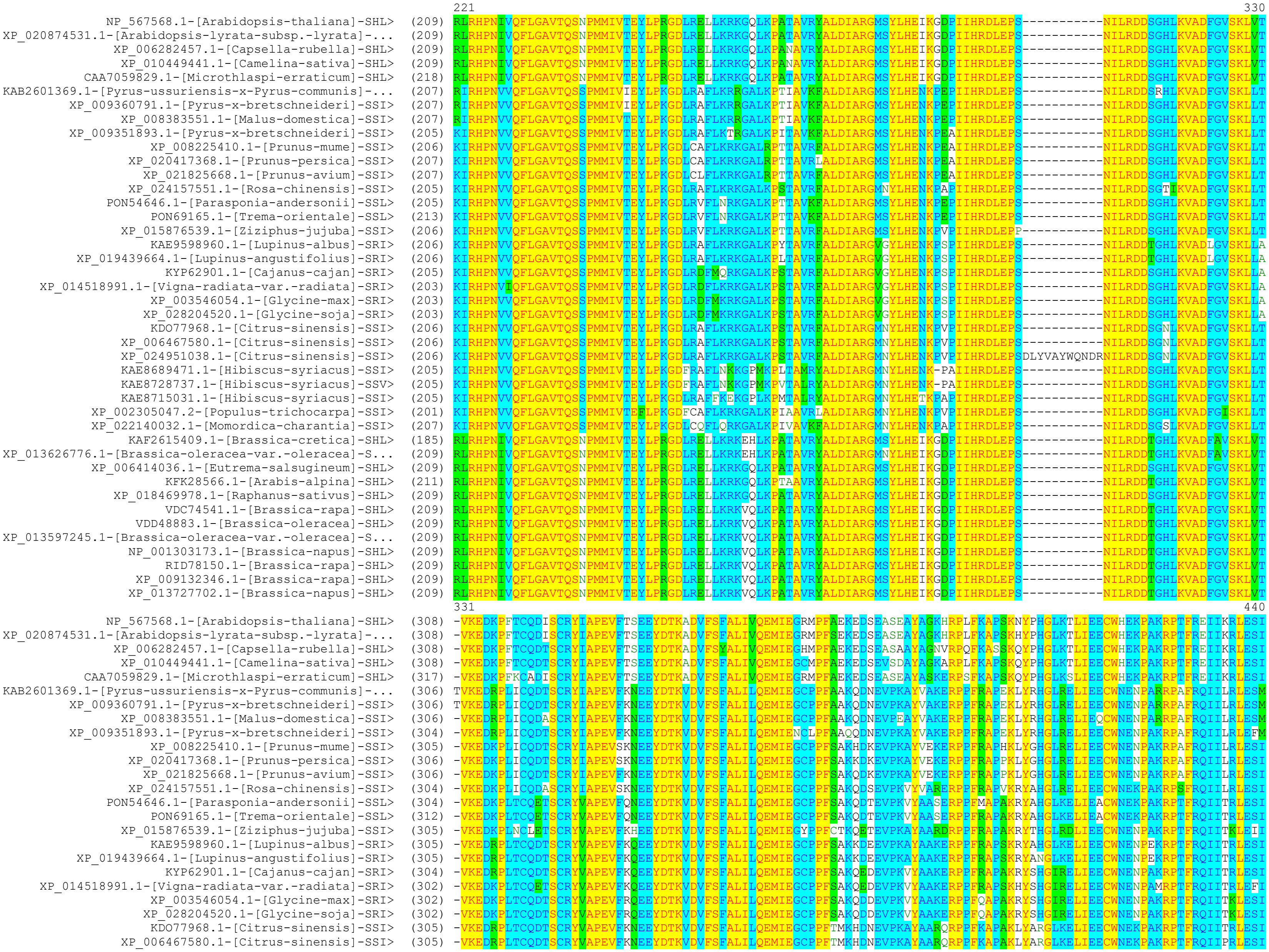

```
           XP_024951038.1-[Citrus-sinensis]-SSI>  (316) -VKEDRPLTCQDTSCRYVAPEVFKNEEYDTKVDVFSFALILQEMIEGCPPFTMKHDNEVPKAYAARQRPPFKAPAKLYARGLKELIEECWNEKPAKRPTFRQIIPRLESI
           KAE8689471.1-[Hibiscus-syriacus]-SSI>  (303) -VKEDKPLTCLDTSCRYVAPEVFSNHGYDTKVDVFSFALILQEMIEGCPPFSAKQDNEVPKAYEASERPPFKASTKNYAHGLKELIEDCWNEKPAKRPTFRQILTKLESI
           KAE8728737.1-[Hibiscus-syriacus]-SSV>  (303) -VKEDKPLTCLDTSCRYVAPEVFSNHDYDTKVDVFSFALILQEMIEGCPPFSAKQDNEVPKAYEARERPPFKASTKNYAHGLKELIEDCWNEKPVKRPTFRQIITKLESI
           KAE8715031.1-[Hibiscus-syriacus]-SSI>  (304) -VKEDKPLTFLEASCRYVAPEVVSSHDYDTKVDVFSFALILQEMIEGCPPFSAKQENDVPKAYAARERPPFKAPAKNYSHGLKELIEDCWNEKPAKRPTFGQIITRLESI
      XP_002305047.2-[Populus-trichocarpa]-SSI>  (300) -VKEEKPLISLDNSWRYVAPEVFKNEEYDTKVDIFSFALILQEMIEGCPPFSAKQEHEVPSAYAAKERPPFRAPTKSYAHGLKELIQECWHENPAKRPTFRQILTRLDAI
      XP_022140032.1-[Momordica-charantia]-SSI>  (306) -VKEDKPLTCQDTSCRYVAPEVFKNNGYDTKVDVFSFALILQEMIEGQPPFSTKKENAIPKEYAAGVRPPFKAPAKCYAHGIKELIEDCWNEKPSKRPNFRQIITRLETI
            KAF2615409.1-[Brassica-cretica]-SHL>  (284) -VKEDKPLTFLDTSCRYIAPEVFTRDEYDTKADVFSFALIVQEMIEGRIPFAEKEDSEASEAYACKERPSFKAPSKHYPHGLKSLIEECWLDKPAKRPTFRAIIKRLESI
XP_013626776.1-[Brassica-oleracea-var.-oleracea]-S...  (308) -VKEDKPLTFLDTSCRYIAPEVFTSDEYDTKADVFSFALIVQEMIEGRIPFAEKEDSEASEAYACKERPSFKAPSKHYPHGLKSLIEECWLDKPAKRPTFRAIIKRLESI
      XP_006414036.1-[Eutrema-salsugineum]-SHL>  (308) -VKEDKPFTCPDTSRRYIAPEVFTSEEYDTKADVFSFALIVQEMIEGRIPFAEKEDNEASDAYARKDRPLFKAPSKHYPHGLKTLIEECWLDKPSKRPTFSEIIKRLESI
               KFK28566.1-[Arabis-alpina]-SHL>  (310) -VKEDKPFTCVDTSCRYIAPEVFTSEEYDTKADVFSFALIVQEMIEGRMPFAEKEDSEASKAYACKARPTFKAPSKLYPHGLKTLIEECWQEKPAKRPTFREVIKKLESI
        XP_018469978.1-[Raphanus-sativus]-SHL>  (308) -VKEDKPFTCLDTSCRYIAPEVFTSEEYDTKADVFSFALIVQEMIEGRMPFAEKEDSEASEAYASKERPLFKAPSKLYPHGLKTLIEECWQDKPAKRPTFREIIKRLESI
               VDC74541.1-[Brassica-rapa]-SHL>  (308) -VKEDKPFTCLDTSCKYIAPEVFTSEEYDTKADVFSFALIVQEMIEGRMPFAEKEDSEASEAYASKERPLFKAPSKLYPHGLKTLIEECWQDKPAKRPTFREIIKRLESI
           VDD48883.1-[Brassica-oleracea]-SHL>  (308) -VKEDKPFTCLDTSCKYIAPEVFTSEEYDTKADVFSFALIVQEMIEGRMPFAEKEDSEASEAYASKERPLFKAPSKLYPHGLKTLIEECWQDKPAKRPTFREIIKRLESI
XP_013597245.1-[Brassica-oleracea-var.-oleracea]-S...  (308) -VKEDKPFTCLDTSCRYIAPEVFTSEEYDTKADVFSFALIVQEMIEGRMPFAEKEDSEASEAYASKERPLFKAPSKLYPHGLKTLIEECWQDKPAKRPTFREIIKRLESI
            NP_001303173.1-[Brassica-napus]-SHL>  (308) -VKEDKPFTCLDTSCRYIAPEVFTSEEYDTKADVFSFALIVQEMIEGRMPFAEKEDSEASEAYASKERPLFKAPSKLYPHGLKTLIEECWQDKPAKRPTFREIIKRLESI
               RID78150.1-[Brassica-rapa]-SHL>  (308) -VKEDKPFTCLDTSCRYIAPEVFTSEEYDTKADVFSFALIVQEMIEGRMPFAEKEDSEASEAYASKERPLFKAPSKLYPHGLKTLIEECWQDKPAKRPTFREIIKRLESI
           XP_009132346.1-[Brassica-rapa]-SHL>  (308) -VKEDKPFTCLDTSCRYIAPEVFTSEEYDTKADVFSFALIVQEMIEGRMPFAEKEDSEASEAYASKERPLFKAPSKLYPDGLKTLIEECWQDKPAKRPTFREIIKRLESI
          XP_013727702.1-[Brassica-napus]-SHL>  (308) -VKEDKPFTCLDTSCRYIAPEVFTSEEYDTKADVFSFALIVQEMIEGRMPFAEKEDSEASEAYASKDRPLFKAPSKLYPHGLKTLIEECWQDKPAKRPTFREIIKRLESI
                                                      441                                          487
      NP_567568.1-[Arabidopsis-thaliana]-SHL>  (417) LHHMGHKRQWRMRP-LTCFQNFEHKKKHNWDLSSHDGSSSGSHL---
XP_020874531.1-[Arabidopsis-lyrata-subsp.-lyrata]-...  (417) LHHMGHKRQWRMRP-LTCFQNFEHKKKQNWDLSSHDGSSSGSHL---
        XP_006282457.1-[Capsella-rubella]-SHL>  (417) LHHMGHKRQWRMRP-LTCFQNFEHKKKHNWDLSSHDGSSSGSHL---
        XP_010449441.1-[Camelina-sativa]-SHL>  (417) LHHMGHKRQWRMRP-LTCFQNFEHKKKHNWDLSSHDGSSSGSHL---
    CAA7059829.1-[Microthlaspi-erraticum]-SHL>  (426) LHHMGHKRQWRMRP-LTCFQNFEHKKKHNWDLSSHDGSSSGSHL---
KAB2601369.1-[Pyrus-ussuriensis-x-Pyrus-communis]-...  (416) YNTLGHKRRWKVRP-LKCFQNLEAMIRKDNSGRSGRSRSSRSTSSI-
     XP_009360791.1-[Pyrus-x-bretschneideri]-SSI>  (416) YNTLGHKRRWKVRP-LKCFQNLEAMIRKDNSGRSGRSRSSRSTSSI-
        XP_008383551.1-[Malus-domestica]-SSI>  (415) YNTLGYKRRWKVRP-LKCFQNLEAMLRKDNSRRSGRSRSSRSTSSI-
     XP_009351893.1-[Pyrus-x-bretschneideri]-SSI>  (413) YNTLGQKSQWKVRP-LQCFQNFEAMFRKNRSGQGSRSRSSRSTSSI-
          XP_008225410.1-[Prunus-mume]-SSI>  (414) HNSLGHKRRWKVRP-LKCFQNIEAMLRKDRSGRGSRSRSSRSTSSI-
        XP_020417368.1-[Prunus-persica]-SSI>  (415) HNSLGHKRRWKVRP-LKCFQNIEAMLRKDRSGRGSRSRSSRSTSSI-
         XP_021825668.1-[Prunus-avium]-SSI>  (415) HNSLGHKRRWKVRP-LKCFQNIEAMLRKDRSGRGSRSRSSRSTSSI-
        XP_024157551.1-[Rosa-chinensis]-SSI>  (413) YNTIGHKRRWKVRP-LKCFPNLEAMLRKDHISLSSRSRSSRSSASSI
     PON54646.1-[Parasponia-andersonii]-SSL>  (413) HNTLGHKRRWKVRPPLKCFQNLEAMLRKDHSNPSSRSSRSSTSSL--
          PON69165.1-[Trema-orientale]-SSL>  (421) HNTLGHKGRWKVRPPMKCFQNLEAMLRKDHSNPSSRSSRSSTSSL--
        XP_015876539.1-[Ziziphus-jujuba]-SSI>  (414) HNSLSHKRRWKVRP-LKCFQNIEAMLRKDRSNPSSRSGSSRSSRSSI
          KAE9598960.1-[Lupinus-albus]-SRI>  (414) NNTIGYRRRWKVRS-FKCFQNLEAFLKRDHSNLSSRGSSSRSASSRI
    XP_019439664.1-[Lupinus-angustifolius]-SRI>  (414) NNTIGYKRRWKVRS-FKCFQNLEALLKRDHSKLSSRGSSSRSASSRI
            KYP62901.1-[Cajanus-cajan]-SRI>  (413) YNAIGQKRRWKVRP-MRCFQNLEALLKRDHSNASSRGGSSRSTSSRI
XP_014518991.1-[Vigna-radiata-var.-radiata]-SRI>  (411) YNAIGQKRRWKVRP-LKCFQNLEALLKRDHSNGSSRGSSRSKSSRI-
          XP_003546054.1-[Glycine-max]-SRI>  (411) YNTIGHKRHWKVRP-LKCFQNLEALLKRDHSNGSSRGGSSRSTSSRI
         XP_028204520.1-[Glycine-soja]-SRI>  (411) YNTIGHKRHWKVRP-LKCFQNLEALLKRDHSNGSSRGGSSRSTSSRI
          KDO77968.1-[Citrus-sinensis]-SSI>  (414) NNSINHKRRWKIRT-MKCFHNLEAMLKKDHSSPSSRSKSSCSTSSI-
        XP_006467580.1-[Citrus-sinensis]-SSI>  (414) NNSINHKRRWKIRT-MKCFHNLEAMLKKDHSSPSSRRKSSCSTSSI-
        XP_024951038.1-[Citrus-sinensis]-SSI>  (425) NNSINHKRRWKIRT-MKCFHNLEAMLKKDHSSPSSRRKSSCSTSSI-
        KAE8689471.1-[Hibiscus-syriacus]-SSI>  (412) HYSFSHKKHWKIRP-LKWFQNLEAKLKRNHSSPSSRSSSSRSTSSI-
        KAE8728737.1-[Hibiscus-syriacus]-SSV>  (412) HNSFSHKKHWKIRP-LKCFQNLEAKLKRNYSSPSSRSSSSRSTSSV-
        KAE8715031.1-[Hibiscus-syriacus]-SSI>  (413) LNSFSHKKRWKIRP-LKCFQNLEAMLKKDHSSPTSCSSSSRSTSSI-
     XP_002305047.2-[Populus-trichocarpa]-SSI>  (409) QNSIGHKRRWKVRP-LKCFQNLEALLKKDRSLSHRSSRSYSSI----
     XP_022140032.1-[Momordica-charantia]-SSI>  (415) HHGLSHRRRWKLPT-LRCFQNVEAKMRRDHISSSRSLSSRSASSI--
          KAF2615409.1-[Brassica-cretica]-SHL>  (393) LHHMGHKRQWRIKP-LTCFQKFEHKKKHNWDVSSPDGSSSGSHL---
XP_013626776.1-[Brassica-oleracea-var.-oleracea]-S...  (417) LHHMGHKRQWRIKP-LTCFQKFEHKKKHNWDVSSADGSSSGSHL---
     XP_006414036.1-[Eutrema-salsugineum]-SHL>  (417) LHHMGHKRQWRMRP-LTCFQNFEHKKKRNWDLGSHDGSSSGSHL---
             KFK28566.1-[Arabis-alpina]-SHL>  (419) LNHMGHKRQWRMKP-LTCFQSPEHKKKHNWDLSSHDGSSSGSHL---
       XP_018469978.1-[Raphanus-sativus]-SHL>  (417) LHHMGHKRQWRMRP-LTCFQNFEHKKKHNWDMSSHDGSSSGSHL---
             VDC74541.1-[Brassica-rapa]-SHL>  (417) LHHMGHKRQWRMRP-LTCFQNFEHKKKHNWDMSSHDGSSSGSHL---
         VDD48883.1-[Brassica-oleracea]-SHL>  (417) LHHMGHKRQWRMRP-LTCFQNFEHKKKHNWDMSSHDGSSSGSHL---
XP_013597245.1-[Brassica-oleracea-var.-oleracea]-S...  (417) LHHMGHKRQWRMRP-LTCFQNFEHKKKHNWDMSSHDGSSSGSHL---
          NP_001303173.1-[Brassica-napus]-SHL>  (417) LHHMGHKRQWRMRP-LTCFQNFEHKKKHNWDMSSHDGSSSGSHL---
             RID78150.1-[Brassica-rapa]-SHL>  (417) LHHMGHKRQWRMRP-LTCFQNFEHKKKHNWDMSSHDGSSSGSHL---
         XP_009132346.1-[Brassica-rapa]-SHL>  (417) LHHMGHKRQWRMRP-LTCFQNFEHKKKHNWDMSSHDGSSSGSHL---
        XP_013727702.1-[Brassica-napus]-SHL>  (417) LHHMGHKRQWRMRP-LTCFQNFEHKKKHNWDMSSHDGSSSGSHL---
```
